# Application of whole genome re-sequencing data in the development of diagnostic DNA markers tightly linked to a disease-resistance locus for marker-assisted selection in lupin (*Lupinus angustifolius*)

**DOI:** 10.1186/s12864-015-1878-5

**Published:** 2015-09-02

**Authors:** Huaan Yang, Jianbo Jian, Xuan Li, Daniel Renshaw, Jonathan Clements, Mark W. Sweetingham, Cong Tan, Chengdao Li

**Affiliations:** Department of Agriculture and Food Western Australia, 3 Baron-Hay Court, South Perth, 6151 Australia; Beijing Genome Institute – Shenzhen, Beishan Industrial Zone, Yantian District, Shenzhen, 518083 China; State Agricultural Biotechnology Centre, Murdoch University, Murdoch, 6150 Australia

**Keywords:** Genome sequencing, Re-sequencing, Next-generation sequencing (NGS), Marker-assisted selection (MAS), Diagnostic markers, Precision breeding

## Abstract

**Background:**

Molecular marker-assisted breeding provides an efficient tool to develop improved crop varieties. A major challenge for the broad application of markers in marker-assisted selection is that the marker phenotypes must match plant phenotypes in a wide range of breeding germplasm. In this study, we used the legume crop species *Lupinus angustifolius* (lupin) to demonstrate the utility of whole genome sequencing and re-sequencing on the development of diagnostic markers for molecular plant breeding.

**Results:**

Nine lupin cultivars released in Australia from 1973 to 2007 were subjected to whole genome re-sequencing. The re-sequencing data together with the reference genome sequence data were used in marker development, which revealed 180,596 to 795,735 SNP markers from pairwise comparisons among the cultivars. A total of 207,887 markers were anchored on the lupin genetic linkage map. Marker mining obtained an average of 387 SNP markers and 87 InDel markers for each of the 24 genome sequence assembly scaffolds bearing markers linked to 11 genes of agronomic interest. Using the R gene *PhtjR* conferring resistance to phomopsis stem blight disease as a test case, we discovered 17 candidate diagnostic markers by genotyping and selecting markers on a genetic linkage map. A further 243 candidate diagnostic markers were discovered by marker mining on a scaffold bearing non-diagnostic markers linked to the *PhtjR* gene. Nine out from the ten tested candidate diagnostic markers were confirmed as truly diagnostic on a broad range of commercial cultivars. Markers developed using these strategies meet the requirements for broad application in molecular plant breeding.

**Conclusions:**

We demonstrated that low-cost genome sequencing and re-sequencing data were sufficient and very effective in the development of diagnostic markers for marker-assisted selection. The strategies used in this study may be applied to any trait or plant species. Whole genome sequencing and re-sequencing provides a powerful tool to overcome current limitations in molecular plant breeding, which will enable plant breeders to precisely pyramid favourable genes to develop super crop varieties to meet future food demands.

**Electronic supplementary material:**

The online version of this article (doi:10.1186/s12864-015-1878-5) contains supplementary material, which is available to authorized users.

## Background

Over thousands of years, the success of plant breeding and selection has relied on phenotypic measurements and breeder experience. The Green Revolution has greatly boosted the world grain production from the 1940s to 1960s. The advent of molecular biotechnology has progressively provided improved tools for precision plant breeding for genetic improvement. The concept of marker-assisted selection (MAS) in plant breeding was proposed in the 1980s [1], and has the potential to vastly enhance the efficiency of genetic improvement [2, 3]. In the last 30 years, molecular markers have been gradually applied to assist plant breeding of agricultural crops. A small number of commercial varieties obtained through marker-assisted breeding were released in rice, soybean, maize, barley, wheat and potato [3]. However, the gap between the expectations and actual impact of MAS is well recognised. Most of the thousands of publications with the terms “marker-assisted selection”, “quantitative trait loci (QTLs)” or “molecular markers” have failed to show any impact in plant breeding [3–5].

There are two major challenges in developing molecular markers for MAS. Firstly, markers must be closely linked to genes of agronomic traits of interest to enable the accurate prediction of desired plant phenotypes [3]. The most desirable markers for MAS are “co-segregating”, where marker genotypes are completely consistent with plant phenotypes in segregating breeding populations. Co-segregating markers offer maximum accuracy on MAS [6, 7]. Secondly, the genotypes of the markers should match plant phenotypes in a wide range of breeding germplasm, allowing broad application in a breeding program. Unfortunately, most of the molecular markers developed over the last 30 years through DNA fingerprinting and genetic mapping are not on target gene sequences; and some genetic distances exist between markers and genes. As a result, genetic recombination may occur in the region between the marker and the gene on the chromosome during evolution and in the plant breeding process. In MAS practice, it is a common problem that cultivars exhibiting desirable marker genotypes may not necessarily have the targeted genes and *vice versa*, which is known as “false positives” [8, 9]. When a cultivar containing a desirable gene is crossed with a breeding line with a false positive marker genotype, the F_2_ progeny plants will show the same marker allele, even though the gene of interest is segregating; therefore, the marker cannot be used for MAS. In order to deal with the prevalence of the false positives, molecular biologists have to undertake “marker validation” work to determine which markers fit which crosses in plant breeding programs [8, 9]. The marker validation step not only increases the overall cost, but also greatly slows down the pace of MAS [8–11]. The best solution for this plight is to develop “diagnostic markers” [12]; that is, markers which have marker genotypes consistent with plant trait phenotypes in all of the breeding germplasm in a breeding program. Diagnostic markers can be used in MAS without the marker validation step [12]. It is now well recognised that the development of diagnostic markers is the key for successful, large-scale and broad application of MAS in plant breeding [10–12].

Functional markers designed on target gene sequences are diagnostic [12], but their development requires identifying, cloning and understanding the genes and their functions. Non-genic diagnostic markers can be developed on random sequences without knowledge of the causal genes by DNA fingerprinting and genetic mapping to select markers with genotypes matched to plant phenotypes in breeding germplasm [13–15]. Traditional methods of developing functional markers and diagnostic markers are tedious and time consuming [16]. The advancements in next-generation sequencing (NGS) and whole genome sequencing have vastly improved the capacity for marker discovery in plants. For example, more than 55 million SNPs were discovered in maize by genome sequencing and re-sequencing [17, 18] and 18.9 million SNPs were obtained by re-sequencing a core collection of rice accessions [19]. Although genome sequencing has been increasingly applied to a wide range of plant species in recent years, there is no report on how to use whole genome sequencing and re-sequencing data to overcome the key challenges and to develop markers widely applicable for plant breeding programs.

Narrow-leafed lupin (*Lupinus angustifolius* L.) was fully domesticated by the early 1970s in Australia and is currently cultivated in Australia, Europe, America and Africa. Over the last 15 years, the DNA fingerprinting method microsatellite-anchored fragment length polymorphism (MFLP) [20] has been used to develop PCR-based markers linked to major genes of industry importance in lupin [16, 21–30]. A genetic linkage map was published in 2005 based on a F_8_ recombinant inbred line (RIL) population originating from a wild × domesticated cross [31]. Three updated versions of the map from the same mapping population followed [32–34]. Most of the markers on these maps were anonymous without sequence information. The application of NGS technology in the last four years has accelerated molecular research on this legume species. NGS has been used to end-sequence a small portion of a bacterial artificial chromosome (BAC) library [35] and in a transcriptome study [36]. NGS was applied as a DNA fingerprinting method to rapidly develop markers for MAS [37], and to construct a sequence-defined, dense genetic map in lupin [38]. More significantly, a draft genome sequence has been established, providing first insight into the lupin genome [38].

Phomopsis stem blight (PSB) caused by the fungal pathogen *Diaporthe toxica* is a major disease in lupin. It infects young stems, remaining as a latent subcuticular coralloid hyphal structure in green plants [39]. Upon plant senescence, the fungus colonizes the stems and develops large lesions. During saprophytic colonization, the fungus produces mycotoxins which can kill animals that graze on lupin stubble [40]. Selection for PSB disease resistance is a key objective in lupin breeding programs. Conventional methods of screening for PSB resistance are difficult and time consuming [41, 42]. Genetic analysis has indicated at least three major genes (*Phr1*, *Phr1* and *PhtjR*) among Australian domesticated lupin lines, each independently conferring resistance to PSB [43, 44]. The R gene *PhtjR* is present in cultivar Tanjil, which has been extensively used as a parental line in the Australian lupin breeding program since its release in 1998. Seven sequence-specific, simple PCR-based markers were developed which flank the R gene *PhtjR* [44]; unfortunately, none have both the key characters of co-segregating and diagnostic desired for MAS. The R gene *PhtjR* has been integrated in the dense genetic map [38]. The objectives of this study were: (1) to undertake genome sequencing and re-sequencing on representative commercial lupin cultivars to discover molecular markers at the whole genome level, and (2) to examine the use of whole genome sequencing and re-sequencing to rapidly develop diagnostic markers closely linked to genes of agronomic interest for large scale application of MAS in molecular lupin breeding without the knowledge of functional genes.

## Results

### Whole genome re-sequencing in nine cultivars

The sequenced commercial cultivars were selected to represent a subset of the lupin breeding history released from 1973 to 2007. For each of the nine re-sequenced lupin cultivars, approximately 10 to 16 Gb of high quality clean sequencing data was obtained (Table 1), which represents 9-15X coverage of the lupin genome size at 1.1 Gb [38]. The sequence reads for each cultivar were assembled into scaffolds using the software program SOAPdenovo [45], and the N50 of assembled scaffolds for each cultivar ranged from 7,633 bp to 10,864 bp (Table 1). The total length of scaffold span for each cultivar ranged from 485 Mbp to 513 Mbp, approximately 90 % of the length of the reference genome assembly based on cultivar Tanjil [38]. The genome GC content of all re-sequenced cultivars was around 32 % (Table 1), which was consistent with the GC content of the reference genome [38].. The re-sequencing data of the nine lupin cultivars have been deposited at Genbank (NCBI accession number: “PRJNA290411”; website address: http://www.ncbi.nlm.nih.gov/bioproject/PRJNA290411).

**Table 1:** Statistics of *denovo* genome sequence assembly of re-sequenced nine cultivars of *Lupinus angustifolius*

	Unicrop	Yorrel	Merrit	Kalya	Tallerack	Quilinock	Mandelup	Coromup	Jenabilup
Raw data (Mbp)	13,334	14,322	15,958	15,760	11,043	17,275	17,727	15,242	14,588
Clean data (Mbp)	12,714	13,642	15,275	15,069	10,524	16,471	16,936	14,605	14,003
Q20 base rate (%)	95.3	96.6	96.9	97.0	95.9	96.8	97.0	97.0	97.1
Number of scaffolds	208,181	277,622	309,904	371,733	256,387	279,705	383,911	268,036	363,979
Total scaffold span (Mbp)	485	497	501	513	488	500	512	504	498
Scaffold N50 (bp)	10,864	9,463	8,814	9,307	9,070	9,835	9,423	10,487	7,633
Average scaffold length (bp)	2,332	1,789	1,617	1,380	1,905	1,789	1,332	1,882	1,369
Longest scaffold (bp)	305,995	183,544	191,423	156,385	229,074	228,256	147,382	211,945	125,123
GC content (%)	32.96	32.70	32.87	32.87	32.62	32.72	32.90	32.65	32.89

### Marker discovery by genome sequencing and re-sequencing

Pairwise comparison of whole genome sequencing data among the reference genome (cultivar Tanjil) and nine re-sequenced lupin cultivars revealed 180,596—795,735 SNP markers (Table 2). The number of insertion/deletion (InDel) markers between cultivars ranged from 33,094 to 122,513. In general, the number of InDels was positively correlated with the number of SNPs detected for each cultivar (Table 2).

**Table 2:** Numbers of SNP markers and InDel markers discovered by pairwise comparison of whole genome sequencing and re-sequencing data among 10 cultivars of Lupinus angustifolius*

Lupin cultivars		Unicrop	Yorrel	Merrit	Kalya	Tallerack	Quilinock	Mandelup	Coromup	Jenabillup
Yorrel	SNP	361,783								
	InDel	74,074								
Merrit	SNP	387,619	379,884							
	InDel	42,670	53,825							
Kalya	SNP	231,674	363,644	399,442						
	InDel	50,771	70,606	40,572						
Tallerack	SNP	457,861	516,424	581,288	466,314					
	InDel	84,239	100,668	71,220	80,863					
Quilinock	SNP	358,425	402,839	386,350	370,174	521,485				
	InDel	60,592	74,470	39,501	56,952	86,272				
Mandelup	SNP	383,509	333,375	363,518	405,193	525,458	399,216			
	InDel	59,611	62,906	34,838	57,048	83,158	57,406			
Coromup	SNP	358,729	318,466	338,840	377,613	509,809	365,480	210,394		
	InDel	59,469	61,381	35,666	57,069	84,167	55,839	39,077		
Jenabillup	SNP	325,324	360,401	312,064	330,028	452,170	180,596	287,423	266,773	
	InDel	52,035	65,258	27,406	48,075	75,939	33,094	42,073	41,398	
Tanjil (Reference)	SNP	644,901	510,722	432,717	564,221	795,735	609,359	601,497	543,048	467,465
	InDel	93,730	105,235	59,780	90,986	122,513	93,675	88,261	88,910	79,623

* SNP markers are presented in black; InDel markers are in green

**Fig. 1:**
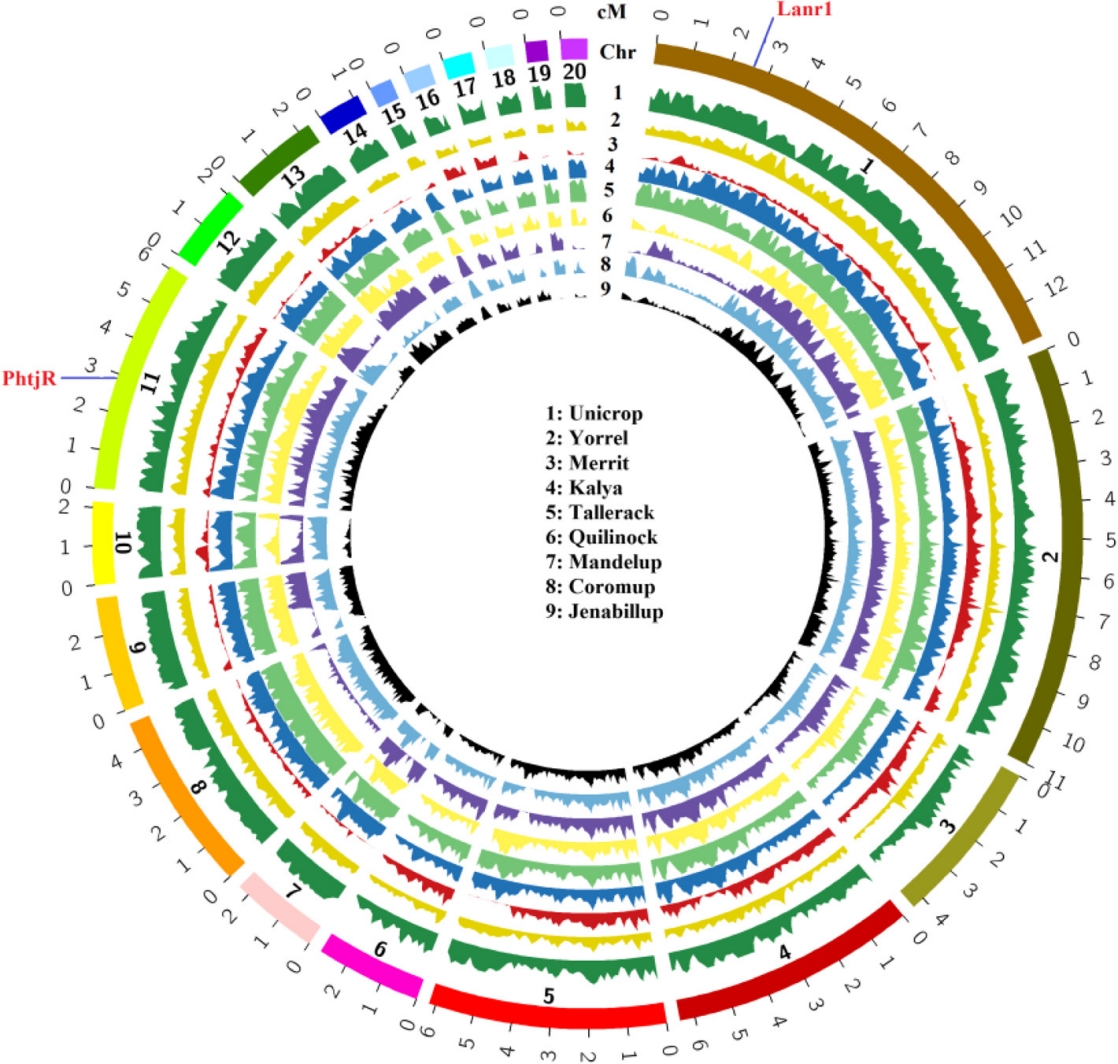
Genome-wide genetic diversity as measured by SNP abundance along each linkage group between reference cultivar Tanjil and nine re-sequenced cultivars of *Lupinus angustifolius*. Twenty linkage groups (SLG) were displayed in a circle. The inner number was SLG index and the outer was physical position (Mb). The circular histograms from circular 1 to 9 with different filling colour were SNP frequency distributions of nine cultivars in whole genome and the response relationship was given in the core area. Higher peaks indicated larger number of SNPs in the interval and lower troughs meant low abundance of SNP. The SNP frequency was counted in non-overlapping 100 kb intervals along each chromosome

Sequence comparison between the reference genome sequence cultivar Tanjil and each of the nine re-sequenced cultivars revealed significant genetic diversity variation at the genome level and at chromosome level (Fig. 1). Cultivar Unicrop, which was the earliest fully domesticated cultivar in this species with most distant pedigree kinship from later released cultivars, showed the greatest level of diversity. In comparison, cultivar Merrit, which has the closest pedigree kinship which reference genome cultivar Tanjil [46], exhibited the least diversity among the nine sequenced cultivars (Fig. 1). At chromosome level, the sequences in sequence-defined linkage group [38] SLG-1, SLG-2, SLG-8 and SLG-11 were highly diverse; while SLG-3 was more conserved, particularly in the second half of this linkage group (Fig. 1).

### Genotyping sequence-defined DNA markers on a genetic linkage map

The genome sequencing and re-sequencing data were successfully applied to genotype markers in the sequence-defined lupin genetic linkage map [38]. A total of 3,277 DNA markers from the 20 linkage groups were characterized for the 10 sequenced cultivars, including 2,902 SNP markers and 375 InDel markers (Additional file 1). By using the DNA sequences bearing the marker variation sites to Blast search of the genome sequencing data, the genotypes of these 3,277 markers on the reference cultivars Tanjil and on the nine re-sequenced cultivars were obtained and recorded (Additional file 1). For completeness, Additional file 1 contains all the 20 SLGs, the list of mapped SNP markers and InDel markers, the sequences bearing the marker sites, and the positions of nucleotides of the mapped markers in their corresponding scaffolds in the reference genome sequence assembly [38].

### Enrichment of molecular markers for the lupin genetic map

Sequence alignments on the 4,214 scaffolds anchored on the sequence-defined lupin genetic linkage map between the two cultivars Tanjil and Unicrop, the two parental lines of the F_8_ RIL mapping population used to establish the dense genetic linkage map [38], identified 207,887 markers, which included 174,639 SNP markers and 33,248 InDel markers (Additional file 2). The average marker density of the enriched genetic linkage map was 127 markers per CentiMorgan. The distribution of these markers in each linkage group is summarized in Table 3. The average length of the 4,214 scaffolds anchored on the genetic linkage map was 17,035 bp. The average numbers of SNP markers and InDel markers per scaffold were 41.4 and 7.9, respectively. Detailed numbers of markers detected on each anchored scaffold, and their corresponding positions in the genetic linkage map are presented in Additional file 2.

**Table 3:** Summary of SNP markers and InDel markers integrated into the sequence-defined genetic linkage map through sequence comparison on scaffolds in *Lupinus angustifolius* ^a^

Linkage groups	Genetic length (cM)	Number of anchored scaffolds^b^	Number of SNP markers detected	Number of InDel markers detected
SLG-1	234.3	763	35,605	5,036
SLG-2	156.7	724	24,158	5,190
SLG-3	149	236	8,071	2,027
SLG-4	144.2	400	14,160	3,202
SLG-5	101.9	365	13,028	2,654
SLG-6	89	129	4,830	1,437
SLG-7	86.5	114	6,959	1,512
SLG-8	85	289	13,761	1,688
SLG-9	83.5	155	8,772	1,578
SLG-10	82.6	138	6,230	1,132
SLG-11	82.2	344	13,869	2,164
SLG-12	64.9	143	5,778	1,094
SLG-13	52.2	155	6,566	1,022
SLG-14	51.1	57	2,806	735
SLG-15	34.5	32	1,676	430
SLG-16	33.3	47	1,468	443
SLG-17	32.4	40	1,612	549
SLG-18	26.6	28	1,616	478
SLG-19	20.6	13	1,499	416
SLG-20	19.4	42	2,175	461
Sub total	1629.9	4,214	174,639	33,248

^a^The sequence-defined genetic linkage map has been published previously [38]

^b^Full list of scaffolds anchored on the genetic linkage map, and the number of markers detected from each scaffold are presented in Additional file 2

### Marker mining on scaffolds linked to genes of agronomic traits of interest

The 24 previously-developed DNA markers linked to 11 genes of agronomic traits of interest were located on 23 scaffolds in the draft genome sequence assembly [38]. Marker MoA [23] and MoLI [30] were on the same scaffold. Each of the other 22 markers was on a separate specific scaffold (Table 4). The length of these 23 scaffolds ranged from 8,191 bp to 64,039 bp, and the average length was 27,687 bp (Table 4).

**Table 4:** Marker mining on 23 genome sequence assembly scaffolds bearing 24 markers linked to 11 key genes of agronomic traits of interest by sequence alignments among 10 sequenced cultivars of *Lupinus angustifolius* ^*a*^

Agronomic traits	Name of markers	Distance between marker and target gene (cM)	Reference	Scaffold identified	Scaffold size (bp)	Number of SNP markers from scaffold sequence alignment	Number of InDel markers from scaffold sequence alignment
Disease resistance gene *PhtjR*	DAFWA6895	0	[38]	Scaffold84773	33,448	489	101
Disease resistance gene *PhtjR*	PhtjM1	1.3	[44]	scaffold70674	11,068	102	39
Disease resistance gene *PhtjR*	PhtjM4	1.1	[44]	scaffold16849	40,716	526	259
Disease resistance gene *PhtjR*	PhtjM6	1.9	[44]	scaffold2572	55,753	808	263
Disease resistance gene *PhtjR*	PhtjM7	1.1	[44]	scaffold57606	13,893	188	62
Disease resistance gene *Lanr1*	DAFWA5820	0	[38]	scaffold 31581	15,706	225	33
Disease resistance gene *Lanr1*	AntjM1	3.5	[22]	scaffold83350	11,407	74	35
Disease resistance gene *Lanr1*	AntjM2	2.3	[14]	scaffold2992	33,979	341	188
Disease resistance gene *Lanr1*	AnSeq3	0.9	[37]	Scaffold33942	64,039	716	138
Disease resistance gene *Lanr1*	AnSeq4	0.9	[37]	Scaffold31346	33,727	221	158
Seed coat colour	DAFWA6428	0	[38]	scaffold11676	22,481	588	154
Seed coat colour	DAFWA4544	0	[38]	scaffold13708	44,176	821	81
Disease resistance gene *AnMan*	AnManM1	5.0	[16]	scaffold36514	50,220	311	213
Disease resistance gene *Phr1*	Ph258M1	5.7	[21]	scaffold84752	21,471	292	94
Disease resistance gene *Phr1*	Ph258M2	2.1	[21]	scaffold16252	15,559	212	25
Resistance gene against lupin rust disease	RustM1	Unknown	Unpublished	scaffold15347	42,210	578	25
Early flowering gene *Ku*	KuH	0	[25]	scaffold21489	30,923	676	23
Soft-seed coat gene *mollis*	MoA, MoLi	0	[23, 30]	scaffold75616	14,783	63	16
Pod-non-shattering *le*	LeLi	6.0	[29]	scaffold87978	9,909	59	17
Pod-non-shattering gene *le*	LeM2	1.3	[24]	scaffold79908	20,738	103	22
Pod-non-shattering gene *tardus*	TaM1	2.1	[26]	scaffold15347	21,529	578	25
Pod-non-shattering gene *tardus*	TaLi	1.4	[27]	scaffold36274	8,191	62	4
Low alkaloid gene *iucundus*	IucLi	0.9	[28]	scaffold30160	20,677	667	22
Average scaffold size and marker numbers					27,687	378	87

^*a*^The list of 10 sequenced cultivars is presented in Tables 2 and 5

Sequence alignments on the 23 scaffolds among 10 sequenced cultivars discovered a total of 8,700 SNP markers and 1,997 InDel markers (Table 4). The average numbers of SNP and InDel markers for each scaffold were 378 and 87, respectively. Generally, scaffolds in longer length contained more markers than shorter scaffolds. For example, scaffold2572 (55,753 bp in length) contained 1,071 markers; while scaffold36247 (8,191 bp in length) had 66 markers (Table 4).

### Development of diagnostic markers linked to the R gene *PhtjR* by genotyping markers from the genetic linkage map

The R gene *PhtjR* conferring resistance to PSB disease was mapped in the SLG-11 of the sequence-defined genetic linkage map of lupin (Additional file 1). Of the 3,277 genotyped markers, 343 were on SLG-11 (Additional file 1). Thirty-three genotyped markers were distributed within 5 centiMorgans (cM) of the R gene *PhtjR* (highlighted in green in Additional file 1; also presented in Table 5). The comparison between the *PhtjR* gene phenotypes and the marker genotypes among the 10 sequenced cultivars identified 17 markers where the marker genotypes completely matched the PSB disease phenotypes (Table 5); these 17 markers were considered “candidate diagnostic markers” for the *PhtjR* gene. The other 18 markers showed the R-allele marker genotype on one or more cultivars without the R gene, which is the linkage disequilibrium decay [47], and is also called “false positive” [11, 48, 49] (Table 5).

**Table 5:**
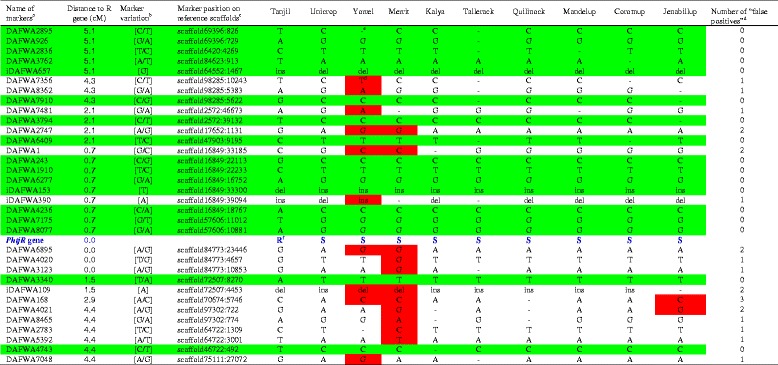
Identification of candidate diagnostic markers through genotyping sequence-defined markers with whole genome sequencing data from 10 cultivars on genetic linkage map flanking the R gene *PhtjR* conferring resistance to phomopsis in *Lupinus angustifolius*

^a^Markers showing genotypes completely consistent with PSB disease phenotypes on all 10 cultivars are considered candidate diagnostic markers and are highlighted in green

^b^Two nucleotides separated by a stroke line in brackets are SNP markers; nucleotides in brackets without a stroke line are InDel markers

^c^Marker positions are the nucleotide positions on the reference genome sequence assembly from cultivar Tanjil (Genbank BioProject number PRJNA179231)

^d^Markers showing R-allele genotype on cultivars without the R gene *Phtj* (false positives) are highlighted in red

^e^Marker sequences missing in genome re-sequencing were recorded as missing data “-”

^f^Genotypes of R gene *PhtjR* on sequenced cultivars presented in blue: R = presence of *PhtjR* gene; S = absence of *PhtjR* gene [44]

**Fig. 2:**
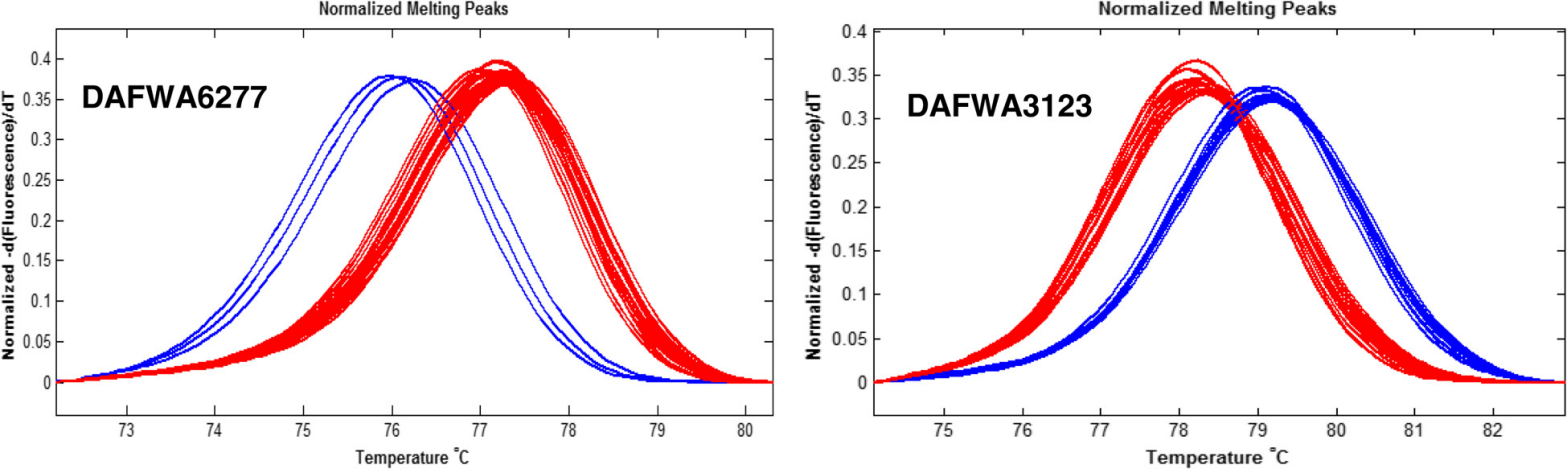
Validation of simple PCR-based SNP markers linked to the R gene *PhtjR* conferring phomopsis stem blight disease resistance on all 27 historical and current cultivars of *Lupinus angustifolius* released in Australia by high resolution melting (HRM) on LightScanner. SNP marker DAFWA6277 (left) was confirmed as diagnostic for the *PhtjR* gene, as the three cultivars (Wonga, Tanjil and Barlock) showed the resistance marker allele (melting curves in blue), while all the other 23 cultivar not possessing the R gene has the susceptible marker allele (melting curves in red). In contrast, SNP marker DAFWA3123 (right) was confirmed as non-diagnostic, since six cultivars (Table 7) without the R gene had the resistance marker allele (melting curves in blue). Detailed records of genotypes for 27 cultivars of these two markers are presented in Table 7

Five candidate diagnostic markers, together with five non-diagnostic markers as controls, were converted into sequence-specific simple PCR markers by designing a pair of sequence-specific primers flanking each SNP site (Table 6). Validation tests confirmed that the five candidate diagnostic markers, DAFWA926, DAFWA2836, DAFWA3794, DAFWA6277 and DAFWA8077, were truly diagnostic on the 27 historical and current commercial cultivars released in Australia (Table 7). The three SNP markers most closely linked to the R gene (co-segregating), DAFWA3123, DAFWA4020 and DAFWA6895, had six to eight false positives (Table 7). SNP markers DAFWA2747 and DAFWA4021 have seven and eight false positives, respectively (Table 7). The genotypes of SNP markers were easily differentiated by high resolution melting (HRM) on LightScanner (Fig. 2).

**Table 6:** Conversion of SNP markers identified from genotyping markers on genetic linkage map flanking the R gene *PhtjR* into sequence-specific PCR markers suitable for genotyping by high resolution melting (HRM) with LightScanner

Marker	Primers	Primer sequence (5′-3′)
DAFWA926	DAFWA926F	GGTTGGGTTAACTTTTATGTCTAAAATC
	DAFWA926R	GGTAAGTTTATTTTTCTAAAGTTGAAC
DAFWA2836	DAFWA2836F	CACATAAGAATATGGAAATGGAGA
	DAFWA2836R	CTGTAAACTGAAGGTGGGCATT
DAFWA3794	DAFWA3794F	GAAAGGAGAAAACTAATCAACATAAG
	DAFWA3794R	ATTAGGGTTTGAGATAGAGTAACAT
DAFWA2747	DAFWA2747F	CCTAACTTCCGATCCAGTAAGC
	DAFWA2747R	CTTTGATCGCTTGGGTTTC
DAFWA6277	DAFWA6277F	TTCGGGAATTTGTATGAGCT
	DAFWA6277R	GGATGGATTCAAAGGTTCAAG
DAFWA8077	DAFWA8077F	GAGATTATTTTCACAAGCTTCCTC
	DAFWA8077R	CCTTTTAGCTTATTCAATTAGCTTG
DAFWA6895	DAFWA6895F	TGAAGGTCCAATACCAGCAAG
	DAFWA6895R	CAACTTCCCTGGAGCAAAA
DAFWA4020	DAFWA4020F	CTAGATAGTTTCGTTTTATCATAC
	DAFWA4020R	GACATAAAGCTTATATATTTGCA
DAFWA3123	DAFWA3123F	CCCTGGACTCTCTCCCTGTATT
	DAFWA3123R	GAATGAAAGTTTGATATGCATAATAA
DAFWA4021	DAFWA4021F	GCTCAGAAACGGTGTCGTT
	DAFWA4021R	GAAGACCTCCAAAACCAAAGC

**Table 7:**
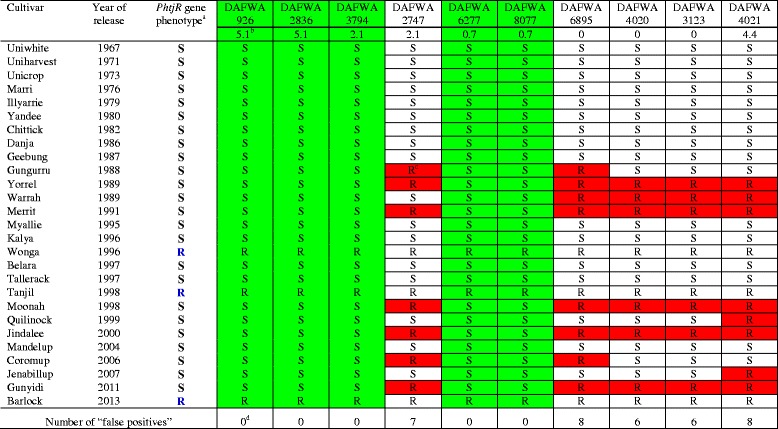
Validation of sequence-specific SNP markers identified from genotyping markers on a genetic linkage map flanking the R gene *PhtjR* conferring resistance to phomopsis stem blight disease on all historical and current commercial cultivars of *Lupinus angustifolius* released in Australia

^a^Genotypes of R gene *PhtjR* on commercial cultivars are presented as: R = presence of *PhtjR* gene; S = absence of *PhtjR* gene [44]

^b^Genetic distance of the marker to the R gene *PhtjR* in centiMorgans (cM) was adapted from the mapping studies [38]

^c^Markers showing R-allele genotype on cultivars without the R gene (false positives) are in highlighted in red

^d^SNP markers showing marker genotypes completely consistent with the *PhtjR* gene phenotypes in all 27 commercial cultivars (no false positive) are diagnostic markers, and are highlighted in green

### Development of diagnostic markers linked to the R gene *PhtjR* by marker mining on a genome sequence assembly scaffold

The three SNP markers most-tightly linked to the R gene *PhtjR* (co-segregating, genetic distance 0 cM) on the genetic linkage map were DAFWA3132, DAFWA4020 and DAFWA6895 (Additional file 1), which were confirmed as non-diagnostic (Table 7). These three SNP markers on the same scaffold84773 in the lupin genome sequence assembly (Additional file 1). Scaffold84773 was used as a test case to investigate the feasibility of developing diagnostic markers by marker mining on genome sequencing assembly scaffolds.

The length of scaffold84773 on the reference genome sequence assembly based on cultivar Tanjil (Genbank accession number “gi 448398638”, AOCW01145302) was 33,448 bp. DNA sequence alignment of the 10 sequenced cultivars on scaffold84773 revealed 489 SNP markers and 101 InDel markers (Additional file 3). Of the 489 SNP markers, 187 had marker genotypes completely matching with *PhtjR* gene phenotypes on all 10 lupin cultivars, and were considered candidate diagnostic markers (highlighted in green in Additional file 3). The other 302 SNP markers were non-diagnostic, evidenced by one or more false positives in the 10 sequenced cultivars. Similarly, 56 InDel markers were identified as candidate diagnostic markers (highlighted in blue in Additional file 3); the other 45 InDel markers were non-diagnostic (Additional file 3).

**Fig. 3:**
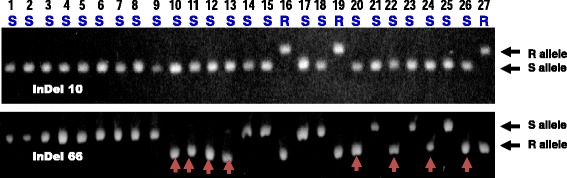
Validation of InDel markers arising from marker mining on genome sequence assembly scaffold84773 linked to the R gene *PhtjR* conferring phomopsis stem blight disease resistance on all 27 historical and current cultivars of *Lupinus angustifolius* by polyacrylamide electrophoresis gels. The 27 cultivars are: Uniwhite (Lane 1), Uniharvest (Lane 2), Unicrop (Lane 3), Marri (Lane 4), Illyarrie (Lane 5), Yandee (Lane 6), Chittick (Lane 7), Danja (Lane 8), Geebung (Lane 9), Gungurru (Lane 10), Yorrel (Lane 11), Warrah (Lane 12), Merrit (Lane 13), Myallie (Lane 14), Kalya (Lane 15), Wonga (Lane 16), Belara (Lane 17), Tallerack (Lane 18), Tanjil (Lane 19), Moonah (Lane 20), Quilinock (Lane 21), Jindalee (Lane 22), Mandelup (Lane 23), Coromup (Lane 24), Jenabillup (Lane 25), Gunyidi (Lane 26) and Barlock (Lane 27). Disease phenotypes of the cultivars are presented as “S” (susceptible) or “R” (resistant) in blue letters. Marker “InDel10” was confirmed as diagnostic for the *PhtjR* gene, since it showed the marker genotypes consistent with PSB phenotypes on all cultivars. In comparison, marker “InDel 66” was confirmed non-diagnostic, since eight cultivars (arrowed in red) without the R gene had the resistance marker allele (“false positives”)

A small subset of 10 SNP markers and four InDel markers arising from sequence alignment on scaffold84773 were selected for further investigation (Table 8). These 14 markers exhibited a wide range of variation in marker genotypes among 10 sequenced lupin cultivars. Markers SNP20, SNP25, SNP263, SNP271, InDel2 and InDel10 showed marker genotypes consistent with R gene *PhtjR* phenotypes of all 10 sequenced cultivars, and were identified as candidate diagnostic markers. On the 10 sequenced cultivars, false positives were discovered in InDel28 (1), SNP250, SNP268 and InDel66 (2), SNP264 (7), and SNP267 and SNP272 (8) (Table 8). Six SNP markers and four InDel markers were converted to sequence-specific PCR markers by designing a pair of sequence-specific primers flanking the marker variation sites (Table 9). Validation tests on the 27 Australian historical and commercial cultivars confirmed three SNP markers, SNP20, SNP25 and SNP263, had genotypes consistent with PSB phenotypes, and were diagnostic for the R gene *PhtjR* (Table 10). On these 27 cultivars, false positives were discovered on SNP271 (1), SNP250 (6) and SNP264 (17) (Table 10). Two InDel markers, InDel2 and InDel10, were diagnostic on all 27 cultivars, while InDel28 and InDel66 had four and eight false positives, respectively (Fig. 3).

**Table 8:**
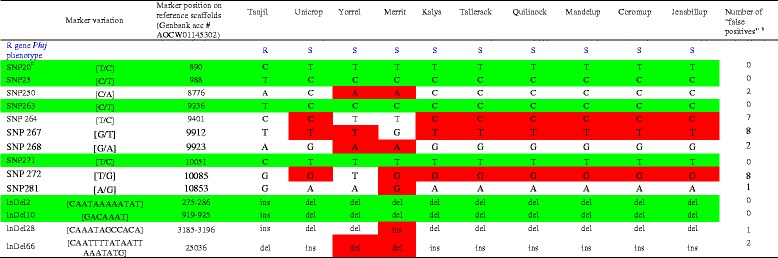
List of a small portion of SNP markers and InDel markers discovered by marker mining on scaffold84773 (Genbank accession # AOCW01145302) showing large variation in marker genotypes among 10 sequenced cultivars and identification of candidate diagnostic markers for the R gene PhtjR of *Lupinus angustifolius* ^a^

^a^The full lists of the 489 SNP markers and 101InDel markers discovered from sequence alignment on scaffold84773 are markers in Additional file 3. Names of identified markers are consistent with the names labelled numerically in Additional file 3

^b^Markers showing R-allele genotypes on cultivars without the R gene *PhtjR* (false positives) are in highlighted in red

^c^Markers showing genotypes consistent with disease resistance phenotypes on all 10 sequenced cultivars are considered as candidate diagnostic markers, and are highlighted in green

**Table 9:** Conversion of SNP markers and InDel markers arising from marker mining on scaffold84773 into sequence-specific PCR markers in *Lupinus angustifolius*

Marker name	Primers	Primer sequence (5′-3′)
SNP 20	SNP20F	GTCCCTGCCATTATTAATAGTTACT
	SNP20R	CATCATGAGTCAATTTACCACTTA
SNP 25	SNP25F	GTCACTAATTTTATCTTTGCAAGA
	SNP25R	GATCATAAGAATAATAATAATAATTTGGT
SNP 250	SNP250F	GACTTAGTAATGTGCAACAAGAG
	SNP250R	CTGACACTACAGGTTCGCCT
SNP 263	SNP263F	GGAACATTGTGATTCAGTCACC
	SNP263R	GATAGGTTTGTTGCAATAAGCG
SNP264	SNP264F	GTTTCTTAGTTGCATAGTTGCAA
	SNP264R	CAAAACATTCATAAGTAACAAGG
SNP271	SNP271F	CGACACCATCTGATATATGAAAATAA
	SNP271R	ACCGGAAATCTGTGTTTTTC
InDel2	InDel2F	GATAAAGTATATCTAAATTATGTTTGC
	InDel2R	CTATATTTTGTATCAATTATAACAAATT
InDel10	InDel10F	GTTAAGTGGTAAATTGACTCATG
	InDel10R	GTTTTRCATTCTTGCAAAGATAAAATTAG
InDel28	InDel28F	CTACAATAGCCACACAAATAG
	InDel28R	GTTTAGATGGCCMTGTGC
InDel66	InDel66F	CTTCTGAGTTGGACCATAAAC
	InDel66R	ACTCACATTTACAGAACTTTAACT

**Table 10:**
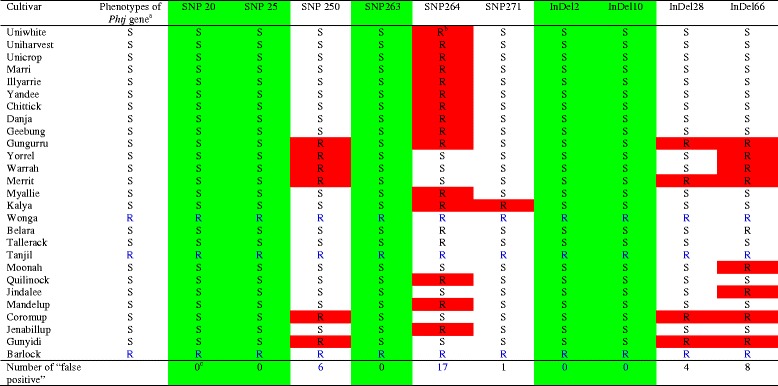
Validation of sequence-specific SNP and InDel markers arising from marker mining on scaffold84773 linked the R gene *PhtjR* conferring resistance to PSB disease on all historical and current commercial cultivars of *Lupinus angustifolius* released in Australia

^a^Genotypes of R gene *PhtjR* on commercial cultivars: R = presence of *PhtjR* gene; S = absence of *PhtjR* gene [44]

^b^Markers showing R-allele genotype on cultivars without the R gene (false positives) are highlighted in red

^c^Markers showing genotypes completely consistent with *PhtjR* gene phenotypes in all 27 commercial cultivars are diagnostic markers, and are highlighted in green

### Linkage confirmation, validation, and application of established markers

The two sequence-specific, PCR-based SNP markers developed from genotyping markers from the genetic linkage map, DAFWA6277 and DAFWA8077, were successfully genotyped on the F_8_ population containing 186 RILs segregating for the R gene *PhtjR* [44]. Linkage analysis using the software program MapManager [50] based on marker genotypes and PSB disease phenotypes confirmed that these two markers are linked to the R gene *PhtjR* with a genetic distance of 1.1 cM, which would be approximately 99 % accurate for selecting lupin progeny with the R gene for MAS.

Three of the sequence-specific, PCR-based markers arising from marker mining on scaffold87443 developed this study—SNP20, SNP25 and InDel10—were genotyped on the F_8_ RIL population derived from the Unicrop × Tanjil cross which was segregating for the *PhtjR* gene [44]. All three markers had marker genotypes completely consistent with PSB disease phenotypes on all 186 RILs (co-segregating). Further validation identified marker genotypes consistent with PSB disease phenotypes on all 69 advanced breeding lines and 163 parental lines used for crossing in the Australian lupin breeding program.

The genetic linkage analysis and validation tests confirmed that markers developed through the two different approaches in this study were all superior to previously developed markers [44] both in accuracy and in wide applicability. The two SNP markers, SNP20 and SNP25, which fit well with the cost-effective, high-throughput SNP genotyping platform LightScanner, have been applied for MAS in the Australian lupin breeding program.

## Discussion

Genome sequence is a fundamental knowledge in understanding the genomics, genetic and biology in plants. Thanks to the advancements in parallel sequencing technologies in recent years, tens of thousands of genomes are in the process of being sequenced [51]. At current time, “close-to-complete genome sequences” have only been achieved on a few model plant species, such as *Arabidopsis*, rice, *Brachypodium*, and *Medicago* [51, 52] where DNA sequences are available almost continuously from the beginning to the end of each chromosome in the genomes. The lengths of sequence span of “complete” genome sequences are equal to the plant genome sizes. However, the majority of other published plant genomes are still at “draft” stage, where genome sequences are presented as large pieces of scaffold sequences. The scaffolds sequences can be aligned into each chromosome through the help of dense genetic linkage maps [53, 54], but many gaps exist between scaffolds on each chromosome. The sequence spans of “draft” genome sequences are smaller than the genome sizes. For examples, the length span of recently released high-depth (358X) genome sequence (1.34Gb) reached to 89.3 % coverage of the oak tree genome size (1.5Gb) [55]; the length of the genome sequence reported on Setaria (396.7 Mbp) was 77.8 % of the genome size (510 Mbp) [54]; the length of the cucumber genome sequence published (243.5 Mbp) was approximately 66 % of the genome size (367 Mbp) [56]. The two major challenges for obtaining complete genome sequences in plant genome sequencing projects are the large genome sizes and the repetitive sequences [52]. The lupin draft genome sequence has a relatively low genome coverage at 51.9 % [38], which was duo to three factors: the lupin genome size is pretty large (at 1.153 Gb) [38]; the genome is rich in repetitive sequences [34]; and the draft sequence was generated from a low costing sequencing project (equivalent to US$5,000) originated from two sequencing libraries with sequencing depth only at 27X [38]. In this study, the genome sequencing and re-sequencing data were used in the identification and selection of candidate diagnostic markers linked to a gene conferring disease resistance. The final selected candidate markers then went through the genetic linkage confirmation step and validation step in the same way as in other standard marker development methods [16, 21, 37]. The linkage confirmation and validation steps ensured that the final markers recommended for MAS were single copy in the genome, were closely linked to gene of interest, were applicable to wide range of breeding germplasm, and were desirable for marker-assisted plant breeding. There are lively discussions among plant scientists about what more can be gained from an in-depth, time-consuming and costly effort to generate high-quality complete sequences than from low coverage draft genome sequences [52]. The results in this study have demonstrated that low coverage genome sequencing and re-sequencing data were sufficient and very effective on marker development in molecular plant breeding. The same low coverage lupin genome sequence was also very successful in the discovery of a candidate gene based diagnostic markers linked to anthracnose disease resistance [38], and in the conversion of previously established gel-based InDel markers into SNP markers to suit modern SNP genotyping platforms for marker implementation in lupin breeding [51].

This study was the first attempt at whole genome re-sequencing of the legume crop species *L. angustifolius* following a 2013 report on its draft genome sequence [38]. Comparing the genome sequences of 10 sequenced cultivars identified 0.3 to 0.6 million molecular markers, which demonstrated the power of whole genome sequencing and re-sequencing for marker discovery. These markers provide lupin breeders and molecular geneticists with a broader suite of options for a wide range of breeding and research purposes. Lupin is a relatively new agricultural crop, domesticated in the early 1970s from its wild relatives. The abundance of SNP and InDel markers among commercial cultivars reflects the rich genetic diversity of the wild parental lines used in the domestication and breeding efforts over the last 40 years. It is evident that the selection pressure for certain desirable agronomic traits of interest in the lupin breeding program had a major impact on genetic diversity at chromosome level. For example, anthracnose disease caused a serious epidemic in Australia in 1996. A major R gene, *Lanr1*, had been exclusively utilized by the lupin breeding program to combat the disease since 1996 [22]; which resulted in the lower genetic diversity in SLG-1 where the *Lanr1* gene was mapped among the recently released commercial cultivars. In contrast, there are at least three major R genes each independently conferring resistance to phomopsis stem blight disease applied in the Australian lupin breeding program [44]; the lack of selection pressure for *PhtjR* gene has helped to preserve the genetic diversity in SLG-11 where the *PhtjR* gene was mapped.

Genetic mapping is a commonly-used approach for marker-trait association discovery in plant molecular studies. In the last three decades, genetic linkage maps have been constructed for most cultivated grain crops. The application of NGS and genome sequencing in recent years has enhanced the power of plant genetic mapping. For example, a genotyping by sequencing (GBS) study discovered and mapped 416,856 markers in wheat [57]; a whole genome sequencing study on a F_8_ RIL population in rice mapped 1,226,791 SNP markers [58]; and sequencing and physical mapping identified 1,013,161–2,053,580 SNP markers in each of four mapping populations in barley [59]. In this study, we anchored 207,887 markers on the lupin genetic linkage map. In theory, all markers with known DNA sequences on genetic linkage maps can be genotyped by whole genome sequencing and re-sequencing data. With so many markers available on genetic linkage maps, the genes of interest to breeders are usually flanked by a large number of markers, which provides ample choice for identifying diagnostic markers desirable for MAS. Yet with traditional methods, identifying diagnostic markers through conversion and validation tests on a large number of markers is tedious and time consuming. Whole genome sequencing and re-sequencing has been demonstrated in this study to be a powerful approached to select diagnostic markers from genetic maps. The 10 lupin cultivars used in the genome sequencing and re-sequencing in this study were carefully selected based on their pedigree kinship to represent genetic diversity in commercial cultivars released in Australia. Therefore, most of the candidate diagnostic markers identified from genotyping these cultivars were validated as truly diagnostic on a wide range of historical and current commercial cultivars. Two of the sequence-specific, simple PCR-based SNP markers developed in this study, DAFWA6277 and DAFWA8077, meet the two key requirements for MAS of being “diagnostic” and “closely linked (1.1 cM) to the target gene of interest”.

In molecular plant breeding, it is common that markers identified from DNA fingerprinting and genetic mapping may not be diagnostic even though they are closely linked to genes of interest, which limited their application for MAS in plant breeding [8–11]. In this study, we demonstrated that whole genome sequencing and re-sequencing can be applied to develop diagnostic markers for MAS through marker mining on scaffolds bearing non-diagnostic markers. All of the 24 previously-established markers linked to the 11 genes of agronomic interest in lupin were successfully located on their specific scaffolds in the genome sequence assembly. Marker mining through scaffold sequence alignments obtained, on average, 378 SNP markers and 87 InDel markers for each of 23 scaffolds bearing markers linked to lupin genes of breeder interest. In the example of PSB disease resistance, none of the three SNP markers most-tightly linked (co-segregating, or 0 cM) to the R gene *PhtjR* on the genetic map were diagnostic. These three non-diagnostic markers were located on the same scaffold87443. Of the 590 DNA markers obtained from marker mining from scaffold87443, a staggering 243 markers showed a diagnostic nature in the 10 sequenced cultivars, which illustrates the effectiveness of this marker development strategy. Three markers developed by marker mining on the scaffold (two SNPs and one InDel marker) were confirmed as truly diagnostic on all of the commercial cultivars, breeding lines and parental lines, and co-segregated with the R gene which is highly desirable for MAS.

Development of diagnostic markers closely linked to genes of agronomic interest is the key to the successful broad application of MAS in routine plant breeding. Functional markers, also called genic markers, are clearly the best type of marker for MAS because there is no risk of genetic recombination to cause false positives. Functional markers have broad application for MAS in a breeding program without the need for a marker validation step. In major crops, functional markers have been successfully developed and applied in plant breeding, such as functional markers for the *Pm3* gene conferring resistance against powdery mildew disease [60], the *Cre3* gene conferring nematode resistance [12] in wheat, the fragrance gene in soybean [61] and the bacterial leaf blight disease resistance genes *xa5* [62] and *Xa21* [63] in rice. However, a plant genome may contain tens of thousands of genes [53, 64], and the development of functional markers requires identifying, cloning and determining the functions of target genes, all of which requires considerable research effort. The principle of the methods in developing non-genic diagnostic markers through whole genome sequencing and re-sequencing seen in this study is the same as that for DNA fingerprinting and genetic mapping in other crops, such as the SSR marker *Xgwm382* for yellow rust disease resistance [13, 65] and a sequence-tagged microsatellite marker stem rust disease resistance gene *Sr2* [66, 67] in wheat. The marker development strategies illustrated here do not require tedious gene cloning. In MAS, markers linked to target genes within 1 cM genetic distance provide >99 % accuracy for predicting and selecting desired genes, which satisfies the needs of most plant breeding applications. In lupin, 1 cM genetic distance is equivalent to approximately 0.6 Mbp in the lupin genome [38]. Such a large piece of DNA in a chromosome would cover thousands of closely-linked DNA markers, offering ample choice for identifying diagnostic markers for MAS through marker mining by genome sequencing and re-sequencing. The methods demonstrated in this study provide a solution to develop diagnostic markers for plant breeding. Further investigations such as sequencing the pathogen genome [68] and studying the plant-pathogen interactions [69] could lead to the identification of the R gene for the development of functional markers.

The lupin genome size is 1.1 Gb [38], which is slightly larger than the soybean genome at 950 Mbp [53]. Currently, the cost of re-sequencing the whole genomes of nine lupin cultivars to a depth of 10–15 X including bioinformatics analysis is approximately US$15,000 at the Beijing Genome Institute (BGI-Shenzhen). The cost of genome sequencing and re-sequencing in a breeding program is a one-off cost. Once the reference genome sequence and re-sequencing data are available, they can be used for genotyping and selecting diagnostic markers for any agronomic traits of interest within this species. Therefore, whole genome sequencing and re-sequencing provides a cost-effective approach for marker discovery and development for plant breeding programs. Once the marker development work is completed, it enters the marker implementation stage. Molecular markers have been applied to large-scale MAS in the Australian national lupin breeding program since 2002. Leaf samples were taken in breeder’s field plots commencing from three weeks after sowing early in June when plants were in the juvenile stage. Tens of thousands of breeding plants were screened and selected with molecular markers annually [51]. The MAS work was usually completed in the end of August at flowering. The application of MAS has made a major impact on lupin breeding. For example, MAS with markers linked to anthracnose disease resistance has replaced the tedious glasshouse and field disease screening trials, which not only saved the cost, but also increased the genetic improvement efficiency in lupin breeding [51]. The development of diagnostic markers reported in this study provides lupin breeders with new tools for MAS to select phomopsis stem blight resistance in lupin breeding.

## Conclusions

Genome sequencing and re-sequencing revealed large genetic variations among commercial cultivars in *Lupinus angustifolius*. We demonstrated two approaches for rapid development of diagnostic markers for MAS by utilizing genome sequencing and re-sequencing data: (1) by genotyping and selecting markers from genetic linkage maps closely linked to genes of breeder interest, and (2) by marker mining from scaffolds bearing non-diagnostic markers. Whole genome sequencing and re-sequencing provides an efficient and cost-effective way to develop diagnostic markers which has broad application in marker-assisted selection. This approach does not require the gene identification and cloning that is needed to develop functional markers. The marker development strategies illustrated in this study may overcome the bottleneck in developing markers with wide applicability in molecular plant breeding. Whole genome sequencing and re-sequencing will facilitate diagnostic tests and selection without limitation of specific breeding parents or population structures. Plant breeders will be able to precisely pyramid favourable genes and alleles to develop super crop varieties to meet the future food demand.

## Methods

### Plant materials

Cultivars of *L. angustifolius* employed for genome re-sequencing and marker validation tests were grown from single-seed-descent derived self-pollinated lines to minimize heterogeneity. The marker population for genetic linkage analysis was the F_8_ RILs derived from a Unicrop (susceptible to PSB disease) × Tanjil (resistant) cross. Details on this F_8_ population have been described previously [44]. Advanced breeding lines and parental lines used for marker validation were from the Australian national lupin breeding program. All plant materials are kept at the Department of Agriculture and Food Western Australia, and are available for scientific research purpose on request.

### Genome re-sequencing on nine cultivars

The nine re-sequenced cultivars were Unicrop (the first fully domesticated cultivar in this species which was release in 1973), Yorrel (released in 1989), Merrit (1991), Kalya (1996), Tallerack (1997), Quilinock (1999), Mandelup (1994), Coromup (2006), and Jenabillup (2007). Re-sequencing of the nine cultivars was performed by the whole genome shotgun (WGS) approach [70]. DNA was extracted from three-week-old seedlings grown in a glasshouse. DNA was randomly sheared by nebulization, end-repaired with T4 DNA polymerase, and size-selected by gel electrophoresis on 1 % low-melting-point agarose. A sequencing library of insert-size 500 bp was constructed for each cultivar according to the Illumina Inc. manufacturer instructions. Pair-end sequencing of the sequencing libraries was performed on NGS platform Hiseq2000 at Beijing Genome Institutes (BGI-Shenzhen). The sequencing data for each cultivar were assembled by SOAP *de novo* [71]. The assembled sequences were aligned into corresponding scaffolds based on the reference draft genome sequence of Tanjil by Short Oligonucleotide Alignment Program (SOAP 2.20) [72].

### Marker discovery among sequenced cultivars

Genome sequence data of the nine re-sequencing cultivars were mapped onto the reference sequences originated from cultivar Tanjil [38]. Based on the mapping result by SOAP 2.20, uniquely mapped single-end and paired-end results were used in the SNP calling. The genotypes of each individual at every genomic site were calculated by SOAPsnp [66]. Polymorphic loci against the reference sequence were selected and then filtered. SNP markers were recorded if they are supported by at least 3 reads with quality value greater than 20. The InDel markers (insertions and deletions shorter than 10 bp) were identified by gap allowed alignment (additional parameter of “-g 10” was used in SOAP2). InDels supported by at least three pair reads were detected by SOAPindel pipeline (http://soap.genomics.org.cn/) as described by Zheng et al [67]. Genomewide genetic diversity between reference cultivar Tanjil and the nine re-sequenced cultivars was based on the calculation of SNP abundance along each linkage group in the genetic map [38]. SNP numbers were counted in each non-overlapping 100 kb interval and displayed in a circular histogram using the software of circus (http://circos.ca/).

### Genotyping sequence-defined DNA markers on a genetic linkage map

The sequence-defined lupin genetic linkage map and marker RAD sequence reads were reported previously [38]. The genome sequencing and re-sequencing data from each of the 10 sequenced cultivars were subjected to homology BLAST search with the RAD-seq sequence reads bearing the SNP markers and InDel markers from the genetic linkage map. The nucleotides from the SNP and InDel variation sites were recorded as marker genotypes for each cultivar. Marker sequences missing on the re-sequencing data were recorded as missing data. To maximize stringency, any RAD-seq sequences showing a sequence variation other than the target SNP/InDel site were discarded, and the corresponding genotype scored as “missing data”. Any markers with missing data on more than three of 10 sequenced cultivars were discarded.

### Enrichment of molecular markers for the lupin genetic map

The genetic linkage map of *L. angustifolius* contained 20 SLGs with 8,244 sequence-defined markers, in which 4,214 scaffolds from the draft genome sequence assembly were anchored [38]. DNA sequences of these 4,214 scaffolds were aligned by sequence similarity and compared between cultivars Tanjil and Unicrop, being the two parental lines for the F_8_ RIL population based on which map was constructed [38]. The SNP markers and InDel markers discovered from sequence alignment on each scaffold were traced to each SLG through their respective SNP markers on the map.

### Marker mining on scaffolds bearing markers linked to genes of agronomic traits of interest

In the last 15 years, 24 DNA markers have been established and linked to 11 genes of agronomic traits of interest by DNA fingerprinting methodologies at the Department of Agriculture and Food Western Australian [14, 16, 21–30, 37, 38, 44]. The marker sequences were applied to the BLAST search of the reference genome sequence [38] to identify the specific scaffold for each marker (Table 4). For each scaffold, DNA sequences from 10 sequenced cultivars were aligned to identify the SNP markers and InDel markers for each scaffold, using the principle as demonstrated in Additional file 3.

### Development of diagnostic markers through genotyping molecular markers from genetic linkage map flanking the R gene *Phtj*

The SNP markers and InDel markers with marker genotypes on 10 sequenced cultivars (Additional file 1) flanking the R gene *PhtjR* at genetic distance of 5 cM were investigated for development of diagnostic markers. The marker genotypes were compared with the *PhtjR* gene phenotypes. A marker is considered a “candidate diagnostic marker” for *PhtjR* gene if its genotypes match the *PhtjR* gene phenotypes on all 10 sequenced cultivars. To prove the concept of selection of diagnostic markers by this strategy, five candidate diagnostic markers together with five non-diagnostic markers as controls were selected for marker validation on all 27 historical and current commercial cultivars released in Australia to confirm their diagnostic nature. Each of these 10 selected SNP markers was converted into a sequence-specific, simple PCR-based marker by designing a pair of sequence-specific primers. Screening of these converted markers was conducted by HRM using LightScanner (Idaho Technology Inc., USA) according to the manufacturer’s instructions, except that EvaGreen Dye (Biotium, USA) replaced the LC Green Dye due to its lower cost and good performance.

### Development of diagnostic markers linked to R gene *PhtjR* through marker mining from genome sequence assembly scaffold

The genome sequence assembly scaffold87443, which bears markers most-tightly linked to the R genes *PhtjR* (co-segregating) on the lupin genetic map (Additional file 2) was used as a test case for marker mining to identify diagnostic markers. Genome sequencing data on scaffold87443 from 10 sequenced cultivars were aligned; all SNP markers and InDel markers from the sequence alignment were recorded (Additional file 3). Markers showing genotypes consistent with *PhtjR* gene phenotypes on all 10 sequenced cultivars were regarded as candidate diagnostic markers (Additional file 3). In order to validate their diagnostic nature on a broader range of cultivars, six SNP markers and four InDel markers were converted into sequence-specific PCR-based markers by designing a pair of sequence-specific primers for each. The screening of converted SNP markers was through HRM on LightScanner. InDel markers were screened on 6 % acrylamide gel electrophoresis using the BIO-RAD Protean II electrophoresis unit at 80 volts for 6 h. The 10 converted markers were tested on the 27 historical and current commercial cultivars to examine the correlation of marker genotypes and *PhtjR* gene phenotypes.

### Linkage confirmation and validation of established markers

The two diagnostic markers most closely linked to the *PhtjR* gene identified from genotyping markers from the lupin genetic linkage map (DAFWA6277 and DAFWA8077) and three diagnostic markers arising from marker mining from scaffold 84773 (SNP20, SNP25 and InDel10) were tested on a F_8_ population derived from the cross containing 186 RILs from a Unicrop (susceptible to PSB) × Tanjil (resistant) cross. The marker genotyping score data and PSB disease phenotyping data were merged and analysed using the software program MapManager QTX [45] to confirm the genetic linkage between these markers and the R gene *PhtjR* [44].

The two best SNP markers developed in this study (which were co-segregating with the R gene *PhtjR* and diagnostic on all released commercial cultivars), SNP20 and SNP25, were further validated on the 69 advanced breeding lines and on 163 parental lines used for crossing in the Australian lupin breeding program in 2014 to evaluate their applicability for MAS in lupin breeding.

## Additional files

Additional file 1: Table S1. Genotyping of sequence-defined SNP markers and InDel markers from the genetic linkage map [38] on 10 commercial cultivars through genome sequencing and re-sequencing in *Lupinus angustifolius*. (XLSX 475 kb) Additional file 2: Table S2. Enrichment of SNP markers and InDel markers for the genetic linkage map through sequence alignment on anchored scaffolds between two parental cultivars Tanjil and Unicrop of the mapping population in *Lupinus angustifolius*. (XLSX 260 kb) Additional file 3:
**Discovery of SNP markers and InDel markers, and identification of diagnostic markers for the R gene** ***PhtjR*** **conferring PSB disease resistance by marker mining on scaffold87443 in the genome sequence assembly of** ***Lupinus angustifolius.*** (DOCX 171 kb)

## Abbreviations

MAS: Marker-assisted selection
NGS: Next-generation sequencing
SNP: Single nucleotide polymorphism
InDel: Insertion/deletion
MFLP: Microsatellite-anchored fragment length polymorphism
RILs: Recombinant inbred lines
PSB: Phomopsis stem blight
PCR: Polymerase chain reaction
SLG: Eequence-defined linkage group
RAD-seq: Restriction-site associated DNA sequencing
HRM: High-resolution melting
