# Supplementary material for: Application of whole genome re-sequencing data in the development of diagnostic DNA markers tightly linked to a disease-resistance locus for marker-assisted selection in lupin (Lupinus angustifolius)
Source: BMC Genomics. 2015 Sep 2;16(1):660. doi: 10.1186/s12864-015-1878-5 (PMC4557927; doi:10.1186/s12864-015-1878-5)
Supplement: Additional file 3: — Discovery of SNP markers and InDel markers, and identification of diagnostic markers for the R gene PhtjR conferring PSB disease resistance by marker mining on scaffold87443 in the genome sequence assembly of Lupinus angustifolius. (DOCX 171 kb) [file 12864_2015_1878_MOESM3_ESM.docx]

**Additional file 3.** Discovery of SNP markers and InDel markers, and identification of diagnostic markers linked to the R gene *PhtjR* conferring PSB disease resistance by marker mining on scaffold87443 in the genome sequence assembly of *Lupinus angustifolius.*

* Ten sequenced commercial cutivars and their phenotypes for PSB disease resistance:

- kal = Kalya (Susceptible)
- yor = Yorrel (S)
- jen = Jenabilup(S)
- cor = Coromup (S)
- man = Mandelup (S)
- uni = Unicrop (S)
- tall = Tallerack (S)
- quil = Quilinock (S)
- meri = Merrit (S)
- ref = Tanjil (Resistance). The sequence of reference scaffold87443 was deposited at Genbank (Accession number AOCW01145302).

**SNP markers and InDel markers are labelled numerically in comments.

***SNP markers showing marker genotype matching with R gene *PhtjR* phenotypes in all 10 cultivars are considered “candidate diagnostic markers” and are highlighted in green.

****InDel markers showing marker genotype matching with R gene *PhtjR* phenotypes in all 10 cultivars are considered “candidate diagnostic InDel markers”, and are highlighted in blue in comments.

kal ........................................ 0

yor ........................................ 0

jen ........................................ 0

cor ........................................ 0

man ........................................ 0

uni ........................................ 0

tall ........................................ 0

quil ........................................ 0

meri ........................................ 0

ref ttgttaatctatcatctataatttatactttaaactctta 40

Consensus

kal ........................................ 0

yor ........................................ 0

jen ........................................ 0

cor ........................................ 0

man ........................................ 0

uni ........................................ 0

tall ........................................ 0

quil ........................................ 0

meri ........................................ 0

ref ttacttaaataatagtaatataaatggatgatagtataaa 80

Consensus

kal ........................................ 0

yor ........................................ 0

jen ........................................ 0

cor ........................................ 0

man ........................................ 0

uni ........................................ 0

tall ........................................ 0

quil ........................................ 0

meri ........................................ 0

ref aaaacttgacgaaactattttgaaatgcaaaaaagaatgt 120

Consensus

kal ...........TTAAATATTCAGTACAATAATTACTAATT 29

yor ...........TTAAATATTCAGTACAATAATTACTAATT 29

jen ...........TTAAATATTCAGTACAATAATTACTAATT 29

cor ...........TTAAATATTCAGTACAATAATTACTAATT 29

man ...........TTAAATATTCAGTACAATAATTACTAATT 29

uni ...........TTAAATATTCAGTACAATAATTACTAATT 29

tall ...........TTAAATATTCAGTACAATAATTACTAATT 29

quil ...........TTAAATATTCAGTACAATAATTACTAATT 29

meri ...........TTAAATATTCAGTACAATAATTACTAATT 29

ref attaaaaaaagTTAAATATTCAGTACAATAATTACTAATT 160

Consensus ttaaatattcagtacaataattactaatt

kal AGGAAGAGAATTTTAGATGATCGTACAAAGAGGATTTAAG 69

yor AGGAAGAGAATTTTAGATGATCGTACAAAGAGGATTTAAG 69

jen AGGAAGAGAATTTTAGATGATCGTACAAAGAGGATTTAAG 69

cor AGGAAGAGAATTTTAGATGATCGTACAAAGAGGATTTAAG 69

man AGGAAGAGAATTTTAGATGATCGTACAAAGAGGATTTAAG 69

uni AGGAAGAGAATTTTAGATGATCGTACAAAGAGGATTTAAG 69

tall AGGAAGAGAATTTTAGATGATCGTACAAAGAGGATTTAAG 69

quil AGGAAGAGAATTTTAGATGATCGTACAAAGAGGATTTAAG 69

meri AGGAAGAGAATTTTAGATGATCGTACAAAGAGGATTTAAG 69

ref AGGAAGAGAATTTTAGATGATtGTACAAAGAGGATTTAAG 200

Consensus aggaagagaattttagatgat gtacaaagaggatttaag

kal TTGATAGGTGAAACTATAGTAATAAATGAACAATGATCTA 109

yor TTGATAGGTGAAACTATAGTAATAAATGAACAATGATCTA 109

jen TTGATAGGTGAAACTATAGTAATAAATGAACAATGATCTA 109

cor TTGATAGGTGAAACTATAGTAATAAATGAACAATGATCTA 109

man TTGATAGGTGAAACTATAGTAATAAATGAACAATGATCTA 109

uni TTGATAGGTGAAACTATAGTAATAAATGAACAATGATCTA 109

tall TTGATAGGTGAAACTATAGTAATAAATGAACAATGATCTA 109

quil TTGATAGGTGAAACTATAGTAATAAATGAACAATGATCTA 109

meri TTGATAGGTGAAACTATAGTAATAAATGAACAATGATCTA 109

ref TTGATAGGTGAAACTATAGTAATAAATGAACAATGATCTA 240

Consensus ttgataggtgaaactatagtaataaatgaacaatgatcta

kal GGATTGATAAAGTATATCTAAATTATGTTTGCTAC..... 144

yor GGATTGATAAAGTATATCTAAATTATGTTTGCTAC..... 144

jen GGATTGATAAAGTATATCTAAATTATGTTTGCTAC..... 144

cor GGATTGATAAAGTATATCTAAATTATGTTTGCTAC..... 144

man GGATTGATAAAGTATATCTAAATTATGTTTGCTAC..... 144

uni GGATTGATAAAGTATATCTAAATTATGTTTGCTAC..... 144

tall GGATTGATAAAGTATATCTAAATTATGTTTGCTAC..... 144

quil GGATTGATAAAGTATATCTAAATTATGTTTGCTAC..... 144

meri GGATTGATAAAGTATATCTAAATTATGTTTGCTAC..... 144

ref ctAT.GATAAAGTATATCTAAATTATGTTTGCTACcaata 279

Consensus at gataaagtatatctaaattatgtttgctac

kal .......ATCATGTTAATTAAAAAATTATTATTTTTGTTA 177

yor .......ATCATGTTAATTAAAAAATTATTATTTTTGTTA 177

jen .......ATCATGTTAATTAAAAAATTATTATTTTTGTTA 177

cor .......ATCATGTTAATTAAAAAATTATTATTTTTGTTA 177

man .......ATCATGTTAATTAAAAAATTATTATTTTTGTTA 177

uni .......ATCATGTTAATTAAAAAATTATTATTTTTGTTA 177

tall .......ATCATGTTAATTAAAAAATTATTATTTTTGTTA 177

quil .......ATCATGTTAATTAAAAAATTATTATTTTTGTTA 177

meri .......ATCATGTTAATTAAAAAATTATTATTTTTGTTA 177

ref aaaatatATCATGTTAATTAAAAAATTATTATTTTTtcTA 319

Consensus atcatgttaattaaaaaattattattttt ta

kal TTTTT.TTTCAATATCATTGATACAAAATATAATTTGTTA 216

yor TTTTT.TTTCAATATCATTGATACAAAATATAATTTGTTA 216

jen TTTTT.TTTCAATATCATTGATACAAAATATAATTTGTTA 216

cor TTTTT.TTTCAATATCATTGATACAAAATATAATTTGTTA 216

man TTTTT.TTTCAATATCATTGATACAAAATATAATTTGTTA 216

uni TTTTT.TTTCAATATCATTGATACAAAATATAATTTGTTA 216

tall TTTTT.TTTCAATATCATTGATACAAAATATAATTTGTTA 216

quil TTTTT.TTTCAATATCATTGATACAAAATATAATTTGTTA 216

meri TTTTT.TTTCAATATCATTGATACAAAATATAATTTGTTA 216

ref TTTTTaTTTCAATATtATTGATACAAAATATAATTTGTTA 359

Consensus ttttt tttcaatat attgatacaaaatataatttgtta

kal TAATTGATACAAAATATAGTTTTAT.GTTTTTCACGTCTA 255

yor TAATTGATACAAAATATAGTTTTtT.GTTTTTCACGTCTA 255

jen TAATTGATACAAAATATAGTTTTAT.GTTTTTCACGTCTA 255

cor TAATTGATACAAAATATAGTTTTAT.GTTTTTCACGTCTA 255

man TAATTGATACAAAATATAGTTTTAT.GTTTTTCACGTCTA 255

uni TAATTGATACAAAATATAGTTTTAT.GTTTTTCACGTCTA 255

tall TAATTGATACAAAATATAGTTTTAT.GTTTTTCACGTCTA 255

quil TAATTGATACAAAATATAGTTTTAT.GTTTTTCACGTCTA 255

meri TAATTGATACAAAATATAGTTTTtT.GTTTTTCACGTCTA 255

ref TAATTGATACAAAATATAGTTTTtTtGTTTcTgACGTCTA 399

Consensus taattgatacaaaatatagtttt t gttt t acgtcta

kal TTTTCGTATCGGAAGCAATCCTGTCCGAGCTGAAAATATC 295

yor TTTTCGTATCGGAAGCAATCCTGTCCGAGCTGAgAATATC 295

jen TTTTCGTATCGGAAGCAATCCTGTCCGAGCTGAAAATATC 295

cor TTTTCGTATCGGAAGCAATCCTGTCCGAGCTGAAAATATC 295

man TTTTCGTATCGGAAGCAATCCTGTCCGAGCTGAAAATATC 295

uni TTTTCGTATCGGAAGCAATCCTGTCCGAGCTGAAAATATC 295

tall TTTTCGTATCGGAAGCAATCCTGTCCGAGCTGAAAATATC 295

quil TTTTCGTATCGGAAGCAATCCTGTCCGAGCTGAAAATATC 295

meri TTTTCGTATCGGAAGCAATCCTGTCCGAGCTGAgAATATC 295

ref TTaTCGTATCaGAgaCAATCCTGTCCGAaCTGggAAcATC 439

Consensus tt tcgtatc ga caatcctgtccga ctg aa atc

kal TACAAGTATGTGAGACTAATTTTCTC..AGCGGATATTTT 333

yor TACAAGTATGTGAGACTAATTTTCTC..AGCGGATATTTT 333

jen TACAAGTATGTGAGACTAATTTTCTC..AGCGGATATTTT 333

cor TACAAGTATGTGAGACTAATTTTCTC..AGCGGATATTTT 333

man TACAAGTATGTGAGACTAATTTTCTC..AGCGGATATTTT 333

uni TACAAGTATGTGAGACTAATTTTCTC..AGCGGATATTTT 333

tall TACAAGTATGTGAGACTAATTTTCTC..AGCGGATATTTT 333

quil TACAAGTATGTGAGACTAATTTTCTC..AGCGGATATTTT 333

meri TACAAGTATGTGAGACTAATTTTCTC..AGCGGATATTTT 333

ref TACAAGTATGTGAGACTAAcTcTCcCctAGCGGATATTTT 479

Consensus tacaagtatgtgagactaa t tc c agcggatatttt

kal TCCATCCACAAAACTCGAATTCGATACTTTACTTAAGAAA 373

yor TCCATCCACAAAACTCGAATTCGATACTTTACTTAAGAAA 373

jen TCCATCCACAAAACTCGAATTCGATACTTTACTTAAGAAA 373

cor TCCATCCACAAAACTCGAATTCGATACTTTACTTAAGAAA 373

man TCCATCCACAAAACTCGAATTCGATACTTTACTTAAGAAA 373

uni TCCATCCACAAAACTCGAATTCGATACTTTACTTAAGAAA 373

tall TCCATCCACAAAACTCGAATTCGATACTTTACTTAAGAAA 373

quil TCCATCCACAAAACTCGAATTCGATACTTTACTTAAGAAA 373

meri TCCATCCACAAAACTCaAATTCGATACTTTACTTAAGAAA 373

ref Tt.ATCCACAAgACTCGAATcCaATACTTTACTTAAGAAA 518

Consensus t atccacaa actc aat c atactttacttaagaaa

kal AATCAAATATGTAT...................TTTGATA 394

yor AATCAAATATGTAT...................TTTGATA 394

jen AATCAAATATGTAT...................TTTGATA 394

cor AATCAAATATGTAT...................TTTGATA 394

man AATCAAATATGTAT...................TTTGATA 394

uni AATCAAATATGTAT...................TTTGATA 394

tall AATCAAATATGTAT...................TTTGATA 394

quil AATCAAATATGTAT...................TTTGATA 394

meri AATCAAATATGTAT...................TTTGATA 394

ref AATCAAATATGTAcaacttgaactaacatctgaTTTGATA 558

Consensus aatcaaatatgta tttgata

kal GAACAAATCCTTGGTTAATAAATAGGCTATTTGTTGCATG 434

yor GAACAAATCCTaGGTTAATAAATAGGCTAcTTGTTGCATG 434

jen GAACAAATCCTTGGTTAATAAATAGGCTATTTGTTGCATG 434

cor GAACAAATCCTTGGTTAATAAATAGGCTATTTGTTGCATG 434

man GAACAAATCCTTGGTTAATAAATAGGCTATTTGTTGCATG 434

uni GAACAAATCCTTGGTTAATAAATAGGCTATTTGTTGCATG 434

tall GAACAAATCCTTGGTTAATAAATAGGCTATTTGTTGCATG 434

quil GAACAAATCCTTGGTTAATAAATAGGCTATTTGTTGCATG 434

meri GAACAAATCCTaGGTTAATAAATAGGCTAcTTGTTGCATG 434

ref GAACAAATtCTgGGTTAATAAATAGGCTAcTTGTTGCATG 598

Consensus gaacaaat ct ggttaataaataggcta ttgttgcatg

kal TCATTTTCATTTCATAATGGCAACAAAGAGCGTTGCCATA 474

yor TCATTTTCATTTCATAATGGCAACAAAGAGCGTTGCCATA 474

jen TCATTTTCATTTCATAATGGCAACAAAGAGCGTTGCCATA 474

cor TCATTTTCATTTCATAATGGCAACAAAGAGCGTTGCCATA 474

man TCATTTTCATTTCATAATGGCAACAAAGAGCGTTGCCATA 474

uni TCATTTTCATTTCATAATGGCAACAAAGAGCGTTGCCATA 474

tall TCATTTTCATTTCATAATGGCAACAAAGAGCGTTGCCATA 474

quil TCATTTTCATTTCATAATGGCAACAAAGAGCGTTGCCATA 474

meri TCATTTTCATTTCATAATGGCAACAAAGAGCGTTGCCATA 474

ref TCATTTTCATTTCATAATGGCAACAAAGAGCGTTGCCATA 638

Consensus tcattttcatttcataatggcaacaaagagcgttgccata

kal TATCACAGATCCAGGCAACGTGGGTAGTAACCTTGTAGGG 514

yor TATCACAGATCCAGGCAACGTGGGTAGTAACCTTGTAGGG 514

jen TATCACAGATCCAGGCAACGTGGGTAGTAACCTTGTAGGG 514

cor TATCACAGATCCAGGCAACGTGGGTAGTAACCTTGTAGGG 514

man TATCACAGATCCAGGCAACGTGGGTAGTAACCTTGTAGGG 514

uni TATCACAGATCCAGGCAACGTGGGTAGTAACCTTGTAGGG 514

tall TATCACAGATCCAGGCAACGTGGGTAGTAACCTTGTAGGG 514

quil TATCACAGATCCAGGCAACGTGGGTAGTAACCTTGTAGGG 514

meri TATCACAGATCCAGGCAACGTGGGTAGTAACCTTGTAGGG 514

ref TATCACAGATCCAGGCAACGTGGGTAGTAACCTTGTAGGG 678

Consensus tatcacagatccaggcaacgtgggtagtaaccttgtaggg

kal TATATTTTTTTAATGTCATTTTCATGCATCTCAATACACA 554

yor TATATTTTTTTAATGTCATTTTCATGCATCTCAATACACA 554

jen TATATTTTTTTAATGTCATTTTCATGCATCTCAATACACA 554

cor TATATTTTTTTAATGTCATTTTCATGCATCTCAATACACA 554

man TATATTTTTTTAATGTCATTTTCATGCATCTCAATACACA 554

uni TATATTTTTTTAATGTCATTTTCATGCATCTCAATACACA 554

tall TATATTTTTTTAATGTCATTTTCATGCATCTCAATACACA 554

quil TATATTTTTTTAATGTCATTTTCATGCATCTCAATACACA 554

meri TATATTTTTTTAATGTCATTTTCATGCATCTCAATACACA 554

ref TATATTTTTTTAATGTCATTTTCATGCATCTCAATACACA 718

Consensus tatatttttttaatgtcattttcatgcatctcaatacaca

kal TCACATATGGATCTGGTCGCACTATATTATTTTACAATTC 594

yor TCACATATGGATCTGGTCGCACTATATTATTTTACAATTC 594

jen TCACATATGGATCTGGTCGCACTATATTATTTTACAATTC 594

cor TCACATATGGATCTGGTCGCACTATATTATTTTACAATTC 594

man TCACATATGGATCTGGTCGCACTATATTATTTTACAATTC 594

uni TCACATATGGATCTGGTCGCACTATATTATTTTACAATTC 594

tall TCACATATGGATCTGGTCGCACTATATTATTTTACAATTC 594

quil TCACATATGGATCTGGTCGCACTATATTATTTTACAATTC 594

meri TCACATATGGATCTGGTCGCACTATATTATTTTACAATTC 594

ref TCACATATGGATCTGGTCGCACT................. 741

Consensus tcacatatggatctggtcgcact

kal TTTTAAATATTAATCTCCTTTTATATTCTAAAACAGATTT 634

yor TTTTAAATATTAATCTCCTTTTATATTCTAAAACAGATTT 634

jen TTTTAAATATTAATCTCCTTTTATATTCTAAAACAGATTT 634

cor TTTTAAATATTAATCTCCTTTTATATTCTAAAACAGATTT 634

man TTTTAAATATTAATCTCCTTTTATATTCTAAAACAGATTT 634

uni TTTTAAATATTAATCTCCTTTTATATTCTAAAACAGATTT 634

tall TTTTAAATATTAATCTCCTTTTATATTCTAAAACAGATTT 634

quil TTTTAAATATTAATCTCCTTTTATATTCTAAAACAGATTT 634

meri TTTTAAATATTAATCTCCTTTTATATTCTAAAACAGATTT 634

ref ..............CTCCTTTTATATcCTAAAACAGtTTT 767

Consensus ctccttttatat ctaaaacag ttt

kal AATATATTTTAAATCTA.....AGTGTTGATCTTGATTTT 669

yor AATATATTTTAAATCTA.....AGTGTTGATCTTGATTTT 669

jen AATATATTTTAAATCTA.....AGTGTTGATCTTGATTTT 669

cor AATATATTTTAAATCTA.....AGTGTTGATCTTGATTTT 669

man AATATATTTTAAATCTA.....AGTGTTGATCTTGATTTT 669

uni AATATATTTTAAATCTA.....AGTGTTGATCTTGATTTT 669

tall AATATATTTTAAATCTA.....AGTGTTGATCTTGATTTT 669

quil AATATATTTTAAATCTA.....AGTGTTGATCTTGATTTT 669

meri AATATATTTTAAATCTA.....AGTGTTGATCTTGATTTT 669

ref AATATATTTTAAATCTAtattaAGTGTTGATCTTGATTTT 807

Consensus aatatattttaaatcta agtgttgatcttgatttt

kal GGTTTTCCATTAATAATATATTATTTTTTCTTCAAAGTTT 709

yor GGTTTTCCATTAATAATATATTATTTTTTCTTCAAAGTTT 709

jen GGTTTTCCATTAATAATATATTATTTTTTCTTCAAAGTTT 709

cor GGTTTTCCATTAATAATATATTATTTTTTCTTCAAAGTTT 709

man GGTTTTCCATTAATAATATATTATTTTTTCTTCAAAGTTT 709

uni GGTTTTCCATTAATAATATATTATTTTTTCTTCAAAGTTT 709

tall GGTTTTCCATTAATAATATATTATTTTTTCTTCAAAGTTT 709

quil GGTTTTCCATTAATAATATATTATTTTTTCTTCAAAGTTT 709

meri GGTTTTCCATTAATAATATATTATTTTTTCTTCAAAGTTT 709

ref GGTTTTCCATTAATAATATATTATTTTTTCTTCAAAGTTT 847

Consensus ggttttccattaataatatattattttttcttcaaagttt

kal GAATTACATCTTTTTGTCCCTGCCATTATTAATAGTTACT 749

yor GAATTACATCTTTTTGTCCCTGCCATTATTAATAGTTACT 749

jen GAATTACATCTTTTTGTCCCTGCCATTATTAATAGTTACT 749

cor GAATTACATCTTTTTGTCCCTGCCATTATTAATAGTTACT 749

man GAATTACATCTTTTTGTCCCTGCCATTATTAATAGTTACT 749

uni GAATTACATCTTTTTGTCCCTGCCATTATTAATAGTTACT 749

tall GAATTACATCTTTTTGTCCCTGCCATTATTAATAGTTACT 749

quil GAATTACATCTTTTTGTCCCTGCCATTATTAATAGTTACT 749

meri GAATTACATCTTTTTGTCCCTGCCATTATTAATAGTTACT 749

ref GAATTACATCTTTTTGTCCCTGCCATTATTAATAGTTACT 887

Consensus gaattacatctttttgtccctgccattattaatagttact

kal TTTGTTAAGTGGTAAATTGACTCATGATGAT.......AC 782

yor TTTGTTAAGTGGTAAATTGACTCATGATGAT.......AC 782

jen TTTGTTAAGTGGTAAATTGACTCATGATGAT.......AC 782

cor TTTGTTAAGTGGTAAATTGACTCATGATGAT.......AC 782

man TTTGTTAAGTGGTAAATTGACTCATGATGAT.......AC 782

uni TTTGTTAAGTGGTAAATTGACTCATGATGAT.......AC 782

tall TTTGTTAAGTGGTAAATTGACTCATGATGAT.......AC 782

quil TTTGTTAAGTGGTAAATTGACTCATGATGAT.......AC 782

meri TTTGTTAAGTGGTAAATTGACTCATGATGAT.......AC 782

ref TTcGTTAAGTGGTAAATTGACTCATGATGATgacaaatAa 927

Consensus tt gttaagtggtaaattgactcatgatgat a

kal ATATTGTCATATAGTTCACTCATGGTATTGGTAGTCACTA 822

yor ATATTGTCATgTAGTTCACTCATGGTATTGGTAGTCACTA 822

jen ATATTGTCATATAGTTCACTCATGGTATTGGaAGTCACTA 822

cor ATATTGTCATATAGTTCACTCATGGTATTGGTAGTCACTA 822

man ATATTGTCATATAGTTCACTCATGGTATTGGTAGTCACTA 822

uni ATATTGTCATATAGTTCACTCATGGTATTGGTAGTCACTA 822

tall ATATTGTCATATAGTTCACTCATGGTATTGGTAGTCACTA 822

quil ATATTGTCATATAGTTCACTCATGGTATTGGTAGTCACTA 822

meri ATATTGTCATATAGTTCACTCATGGTATTGGTAGTCACTA 822

ref gcATTGTtATATAGTTCACTCAaGGTATTGGTAGTCACTA 967

Consensus attgt at tagttcactca ggtattgg agtcacta

kal ATTTTATCTTTGCAAGAATGCAAAACCAAATTATTATTAT 862

yor ATTTTATCTTTGCAAGAATGCAAAACCAAATTATTATTAT 862

jen ATTTTATCTTTGCAAGAATGCAAAACCAAATTATTATTAT 862

cor ATTTTATCTTTGCAAGAATGCAAAACCAAATTATTATTAT 862

man ATTTTATCTTTGCAAGAATGCAAAACCAAATTATTATTAT 862

uni ATTTTATCTTTGCAAGAATGCAAAACCAAATTATTATTAT 862

tall ATTTTATCTTTGCAAGAATGCAAAACCAAATTATTATTAT 862

quil ATTTTATCTTTGCAAGAATGCAAAACCAAATTATTATTAT 862

meri ATTTTATCTTTGCAAGAATGCAAAACCAAATTATTATTAT 862

ref ATTTTATCTTTGCAAGAATGtAAAACCAAATTATTATTAT 1007

Consensus attttatctttgcaagaatg aaaaccaaattattattat

kal TATTCTTATGATGATCAAATGAAAATTGCCCTACTTTATA 902

yor TATTCTTATGATGATCAAATGAAAATTGCCCTACTTTATA 902

jen TATTCTTATGATGATCAAATGAAAATTGCCCTACTTTATA 902

cor TATTCTTATGATGATCAAATGAAAATTGCCCTACTTTATA 902

man TATTCTTATGATGATCAAATGAAAATTGCCCTACTTTATA 902

uni TATTCTTATGATGATCAAATGAAAATTGCCCTACTTTATA 902

tall TATTCTTATGATGATCAAATGAAAATTGCCCTACTTTATA 902

quil TATTCTTATGATGATCAAATGAAAATTGCCCTACTTTATA 902

meri TATTCTTATGATGATCAAATGAAAATTGCCCTACTTTATA 902

ref TATTCTTATGATGATCAAATGAAAATTGCCCTACTTTATA 1047

Consensus tattcttatgatgatcaaatgaaaattgccctactttata

kal TGAAGGAGATTTTA.......................... 916

yor TGAAGGAGATTTTA.......................... 916

jen TGAAGGAGATTTTA.......................... 916

cor TGAAGGAGATTTTA.......................... 916

man TGAAGGAGATTTTA.......................... 916

uni TGAAGGAGATTTTA.......................... 916

tall TGAAGGAGATTTTA.......................... 916

quil TGAAGGAGATTTTA.......................... 916

meri TGAAGGAGATTTTA.......................... 916

ref TGAAGGAGATTTTAtgtccccatttttgtttctcattagt 1087

Consensus tgaaggagatttta

kal .AAAAGAAAAAAATATAAAACCAAAAGTTTATATTTTAAT 955

yor .AAAAGAAAAAAATATAAAACCAAAAGTTTATATTTTAAT 955

jen .AAAAGAAAAAAATATAAAACCAAAAGTTTATATTTTAAT 955

cor .AAAAGAAAAAAATATAAAACCAAAAGTTTATATTTTAAT 955

man .AAAAGAAAAAAATATAAAACCAAAAGTTTATATTTTAAT 955

uni .AAAAGAAAAAAATATAAAACCAAAAGTTTATATTTTAAT 955

tall .AAAAGAAAAAAATATAAAACCAAAAGTTTATATTTTAAT 955

quil .AAAAGAAAAAAATATAAAACCAAAAGTTTATATTTTAAT 955

meri .AAAAGAAAAAAATATAAAACCAAAAGTTTATATTTTAAT 955

ref tAAAAagAAAAAAaATAAAAaCAAAAGTTcATATTTTAAT 1127

Consensus aaaa aaaaaa ataaaa caaaagtt atattttaat

kal ATTATTGAATAAAGTTTGATGGATTAGGAACATGATTTTC 995

yor ATTATTGAATAAAGTTTGATGGATTAGGAACATGATTTTC 995

jen ATTATTGAATAAAGTTTGATGGATTAGGAACATGATTTTC 995

cor ATTATTGAATAAAGTTTGATGGATTAGGAACATGATTTTC 995

man ATTATTGAATAAAGTTTGATGGATTAGGAACATGATTTTC 995

uni ATTATTGAATAAAGTTTGATGGATTAGGAACATGATTTTC 995

tall ATTATTGAATAAAGTTTGATGGATTAGGAACATGATTTTC 995

quil ATTATTGAATAAAGTTTGATGGATTAGGAACATGATTTTC 995

meri ATTATTGAATAAAGTTTGATGGATTAGGAACATGATTTTC 995

ref ATTATTGAATAAAGTTTGActGATTAGGAACATGATTTTg 1167

Consensus attattgaataaagtttga gattaggaacatgatttt

kal TTAGGACAAATTGATCCTTGTTCAAAACATATTAAAACAT 1035

yor TTAGGACAAATTGATCCTTGTTCAAAACATATTAAAACAT 1035

jen TTAGGACAAATTGATCCTTGTTCAAAACATATTAAAACAT 1035

cor TTAGGACAAATTGATCCTTGTTCAAAACATATTAAAACAT 1035

man TTAGGACAAATTGATCCTTGTTCAAAACATATTAAAACAT 1035

uni TTAGGACAAATTGATCCTTGTTCAAAACATATTAAAACAT 1035

tall TTAGGACAAATTGATCCTTGTTCAAAACATATTAAAACAT 1035

quil TTAGGACAAATTGATCCTTGTTCAAAACATATTAAAACAT 1035

meri TTAGGACAAATTGATCCTTGTTCAAAACATATTAAAACAT 1035

ref TTAGGACAAAT.aATCCTTGcTCAAAACATATTAAAACAT 1206

Consensus ttaggacaaat atccttg tcaaaacatattaaaacat

kal TTATTTAACTCTGAAGTAGGACATGGATATTAGATTTCTC 1075

yor TTATTTAACTCTGAAGTAGGACATGGATATTAGATTTCTC 1075

jen TTATTTAACTCTGAAGTAGGACATGGATATTAGATTTCTC 1075

cor TTATTTAACTCTGAAGTAGGACATGGATATTAGATTTCTC 1075

man TTATTTAACTCTGAAGTAGGACATGGATATTAGATTTCTC 1075

uni TTATTTAACTCTGAAGTAGGACATGGATATTAGATTTCTC 1075

tall TTATTTAACTCTGAAGTAGGACATGGATATTAGATTTCTC 1075

quil TTATTTAACTCTGAAGTAGGACATGGATATTAGATTTCTC 1075

meri TTATTTAACTCTGAAGTAGGACATGGATATTAGATTTCTC 1075

ref TTATTTAACTCTcAAGTAGGACATGGATATTAGATTTCTC 1246

Consensus ttatttaactct aagtaggacatggatattagatttctc

kal TCTCAACAATCACAATCTTGAACAAAAAGAAAAGATGGAA 1115

yor TCTCAACAATCACAATCTTGAACAAAAAGAAAAGATGGAA 1115

jen TCTCAACAATCACAATCTTGAACAAAAAGAAAAGATGGAA 1115

cor TCTCAACAATCACAATCTTGAACAAAAAGAAAAGATGGAA 1115

man TCTCAACAATCACAATCTTGAACAAAAAGAAAAGATGGAA 1115

uni TCTCAACAATCACAATCTTGAACAAAAAGAAAAGATGGAA 1115

tall TCTCAACAATCACAATCTTGAACAAAAAGAAAAGATGGAA 1115

quil TCTCAACAATCACAATCTTGAACAAAAAGAAAAGATGGAA 1115

meri TCTCAACAATCACAATCTTGAACAAAAAGAAAAGATGGAA 1115

ref TCTCAACAATCACAATCTTGAACAAAAAGAAAAGATGGAA 1286

Consensus tctcaacaatcacaatcttgaacaaaaagaaaagatggaa

kal GAGTCAAAAAGCTAAGCAAGTTTCAGAATCATAACCAGGA 1155

yor GAGTCAAAAAGCTAAGCAAGTTTCAGAATCATAACCAGGA 1155

jen GAGTCAAAAAGCTAAGCAAGTTTCAGAATCATAACCAGGA 1155

cor GAGTCAAAAAGCTAAGCAAGTTTCAGAATCATAACCAGGA 1155

man GAGTCAAAAAGCTAAGCAAGTTTCAGAATCATAACCAGGA 1155

uni GAGTCAAAAAGCTAAGCAAGTTTCAGAATCATAACCAGGA 1155

tall GAGTCAAAAAGCTAAGCAAGTTTCAGAATCATAACCAGGA 1155

quil GAGTCAAAAAGCTAAGCAAGTTTCAGAATCATAACCAGGA 1155

meri GAGTCAAAAAGCTAAGCAAGTTTCAGAATCATAACCAGGA 1155

ref GAGTCAAAAAGCTAAGCAAGTTTCAGAATCATAACCAGGA 1326

Consensus gagtcaaaaagctaagcaagtttcagaatcataaccagga

kal GATATATAATGTCGAGAGGGTATTTTTAATAAGTTCAACT 1195

yor GATATATAATGTCGAGAGGGTATTTTTAATAAGTTCAACT 1195

jen GATATATAATGTCGAGAGGGTATTTTTAATAAGTTCAACT 1195

cor GATATATAATGTCGAGAGGGTATTTTTAATAAGTTCAACT 1195

man GATATATAATGTCGAGAGGGTATTTTTAATAAGTTCAACT 1195

uni GATATATAATGTCGAGAGGGTATTTTTAATAAGTTCAACT 1195

tall GATATATAATGTCGAGAGGGTATTTTTAATAAGTTCAACT 1195

quil GATATATAATGTCGAGAGGGTATTTTTAATAAGTTCAACT 1195

meri GATATATAATGTCGAGAGGGTATTTTTAATAAGTTCAACT 1195

ref GATATATAgTGTCGAGAGGGTATTTTcAATAAGTTCAACT 1366

Consensus gatatata tgtcgagagggtatttt aataagttcaact

kal TCTTGTTTATATGCCTCATTTAAACCCAAAAAATGACATA 1235

yor TCTTGTTTATATGCCTCATTTAAACCCAAAAAATGACATA 1235

jen TCTTGTTTATATGCCTCATTTAAACCCAAAAAATGACATA 1235

cor TCTTGTTTATATGCCTCATTTAAACCCAAAAAATGACATA 1235

man TCTnnnnnnnnnnnnnnnnnnnnnnnnnnAAAATGACATA 1235

uni TCTTGTTTATATGCCTCATTTAAACCCAAAAAATGACATA 1235

tall TCTTGTTTATATGCCTCATTTAAACCCAAAAAATGACATA 1235

quil TCTTGTTTATATGCCTCATTTAAACCCAAAAAATGACATA 1235

meri TCTTGTTTATATGCCTCATTTAAACCCAAAAAATGACATA 1235

ref TCTTGTTTATATGCCTCATTTAAACCCAAAAAATGACATA 1406

Consensus tct aaaatgacata

kal AAAATGACACCTTCTTCATCTCTAGTGATGCTGCACCACC 1275

yor AAAATGACACCTTCTTCATCTCTAGTGATGCTGCACCACC 1275

jen AAAATGACACCTTCTTCATCTCTAGTGATGCTGCACCACC 1275

cor AAAATGACACCTTCTTCATCTCTAGTGATGCTGCACCACC 1275

man AAAATGACACCTTCTTCATCTCTAGTGATGCTGCACCACC 1275

uni AAAATGACACCTTCTTCATCTCTAGTGATGCTGCACCACC 1275

tall AAAATGACACCTTCTTCATCTCTAGTGATGCTGCACCACC 1275

quil AAAATGACACCTTCTTCATCTCTAGTGATGCTGCACCACC 1275

meri AAAATGACACCTTCTTCATCTCTAGTGATGCTGCACCACC 1275

ref AAAATGACACCTTCTTCATCTCTAGTGATG.......... 1436

Consensus aaaatgacaccttcttcatctctagtgatg

kal TTCTTGATTGTCACGCCGACTATGTTGTAAAGATGTAAAC 1315

yor TTCTTGATTGTCACGCCGACTATGTTGTAAAGATGTAAAC 1315

jen TTCTTGATTGTCACGCCGACTATGTTGTAAAGATGTAAAC 1315

cor TTCTTGATTGTCACGCCGACTATGTTGTAAAGATGTAAAC 1315

man TTCTTGATTGTCACGCCGACTATGTTGTAAAGATGTAAAC 1315

uni TTCTTGATTGTCACGCCGACTATGTTGTAAAGATGTAAAC 1315

tall TTCTTGATTGTCACGCCGACTATGTTGTAAAGATGTAAAC 1315

quil TTCTTGATTGTCACGCCGACTATGTTGTAAAGATGTAAAC 1315

meri TTCTTGATTGTCACGCCGACTATGTTGTAAAGATGTAAAC 1315

ref ...................................TAAAC 1441

Consensus taaac

kal ACATGAGGAGAGTCTAATTCAAAAAACTAGTTAATTATGT 1355

yor ACATGAGGAGAGTCTAATTCAAAAAACTAGTTAATTATGT 1355

jen ACATGAGGAGAGTCTAATTCAAAAAACTAGTTAATTATGT 1355

cor ACATGAGGAGAGTCTAATTCAAAAAACTAGTTAATTATGT 1355

man ACATGAGGAGAGTCTAATTCAAAAAACTAGTTAATTATGT 1355

uni ACATGAGGAGAGTCTAATTCAAAAAACTAGTTAATTATGT 1355

tall ACATGAGGAGAGTCTAATTCAAAAAACTAGTTAATTATGT 1355

quil ACATGAGGAGAGTCTAATTCAAAAAACTAGTTAATTATGT 1355

meri ACATGAGGAGAGTCTAATTCAAAAAACTAGTTAATTATGT 1355

ref AtATGAGGAGAGcCTAATTtAAAAAAtTAGTTAATgATGT 1481

Consensus a atgaggagag ctaatt aaaaaa tagttaat atgt

kal TAGAAAATATTATAACTTAAGTATCACATTAAATTTATTA 1395

yor TAGAAAATATcATAACTTAAGTATCACATTAAATTTATTA 1395

jen TAGAAAATATTATAACTTAAGTATCACATTAAATTTATTA 1395

cor TAGAAAATATTATAACTTAAGTATCACATTAAATTTATTA 1395

man TAGAAAATATTATAACTTAAGTATCACATTAAATTTATTA 1395

uni TAGAAAATATTATAACTTAAGTATCACATTAAATTTATTA 1395

tall TAGAAAATATTATAACTTAAGTATCACATTAAATTTATTA 1395

quil TAGAAAATATTATAACTTAAGTATCACATTAAATTTATTA 1395

meri TAGAAAATATcATAACTTAAGTATCACATTAAATTTATTA 1395

ref TAGAAAAcATgATAACTTAAGTATCACATTAAATTTATTA 1521

Consensus tagaaaa at ataacttaagtatcacattaaatttatta

kal TATTATCTAATTGAGTTGAAAATATATTTTATATTTCAAA 1435

yor TATTATCTAATTGAGTTGAAAATATATTTTATATTTCAAA 1435

jen TATTATCTAATTGAGTTGAAAATATATTTTATATTTCAAA 1435

cor TATTATCTAATTGAGTTGAAAATATATTTTATATTTCAAA 1435

man TATTATCTAATTGAGTTGAAAATATATTTTATATTTCAAA 1435

uni TATTATCTAATTGAGTTGAAAATATATTTTATATTTCAAA 1435

tall TATTATCTAATTGAGTTGAAAATATATTTTATATTTCAAA 1435

quil TATTATCTAATTGAGTTGAAAATATATTTTATATTTCAAA 1435

meri TATTATCTAATTGAGTTGAAAATATATTTTATATTTCAAA 1435

ref TATTATtTgATTGAGTTGAAAATATATTTTATATTTCAAA 1561

Consensus tattat t attgagttgaaaatatattttatatttcaaa

kal ATGTATATTACTTCTTATATTCATATTATTCTTATTGATT 1475

yor ATGTATATTACTTCTTATATTCATATTATTCTTATTGATT 1475

jen ATGTATATTACTTCTTATATTCATATTATTCTTATTGATT 1475

cor ATGTATATTACTTCTTATATTCATATTATTCTTATTGATT 1475

man ATGTATATTACTTCTTATATTCATATTATTCTTATTGATT 1475

uni ATGTATATTACTTCTTATATTCATATTATTCTTATTGATT 1475

tall ATGTATATTACTTCTTATATTCATATTATTCTTATTGATT 1475

quil ATGTATATTACTTCTTATATTCATATTATTCTTATTGATT 1475

meri ATGTATATTACTTCTTATATTCATATTATTCTTATTGATT 1475

ref ATGTATATTACTTCTTATATTCATATTATTCTTATTGATT 1601

Consensus atgtatattacttcttatattcatattattcttattgatt

kal AATTAGAAAATATAGGTAAATGTATATTTTATTTTCCTTA 1515

yor AATTAGAAAATATAGGTAAATGTATATTTTATTTTCCTTA 1515

jen AATTAGAAAATATAGGTAAATGTATATTTTATTTTCCTTA 1515

cor AATTAGAAAATATAGGTAAATGTATATTTTATTTTCCTTA 1515

man AATTAGAAAATATAGGTAAATGTATATTTTATTTTCCTTA 1515

uni AATTAGAAAATATAGGTAAATGTATATTTTATTTTCCTTA 1515

tall AATTAGAAAATATAGGTAAATGTATATTTTATTTTCCTTA 1515

quil AATTAGAAAATATAGGTAAATGTATATTTTATTTTCCTTA 1515

meri AATTAGAAAATATAGGTAAATGTATATTTTATTTTCCTTA 1515

ref AATTAGAAAATATAGGTAAATGTATATTTTATTTTCCTTA 1641

Consensus aattagaaaatataggtaaatgtatattttattttcctta

kal GCTTACAAAAAAAAATGAGTGACACGTGAAGTATTATAGG 1555

yor GCTTACAAAAAAAAATGAGTGACACGTGAAGTATTATAGG 1555

jen GCTTACAAAAAAAAATGAGTGACACGTGAAGTATTATAGG 1555

cor GCTTACAAAAAAAAATGAGTGACACGTGAAGTATTATAGG 1555

man GCTTACAAAAAAAAATGAGTGACACGTGAAGTATTATAGG 1555

uni GCTTACAAAAAAAAATGAGTGACACGTGAAGTATTATAGG 1555

tall GCTTACAAAAAAAAATGAGTGACACGTGAAGTATTATAGG 1555

quil GCTTACAAAAAAAAATGAGTGACACGTGAAGTATTATAGG 1555

meri GCTTACAAAAAAAAATGAGTGACACGTGAAGTATTATAGG 1555

ref tCTTACAAAAAAA...GAGTGACACGTGAAGTATTATAGG 1678

Consensus cttacaaaaaaa gagtgacacgtgaagtattatagg

kal ACATCAAATATATCTCCAAGTGAGTTAGGTAAGTTAGGTA 1595

yor ACATCAAATATATCTCCAAGTGAGTTAGGTAAGTTAGGTA 1595

jen ACATCAAATATATCTCCAAGTGAGTTAGGTAAGTTAGGTA 1595

cor ACATCAAATATATCTCCAAGTGAGTTAGGTAAGTTAGGTA 1595

man ACATCAAATATATCTCCAAGTGAGTTAGGTAAGTTAGGTA 1595

uni ACATCAAATATATCTCCAAGTGAGTTAGGTAAGTTAGGTA 1595

tall ACATCAAATATATCTCCAAGTGAGTTAGGTAAGTTAGGTA 1595

quil ACATCAAATATATCTCCAAGTGAGTTAGGTAAGTTAGGTA 1595

meri ACATCAAATATATCTCCAAGTGAGTTAGGTAAGTTAGGTA 1595

ref ACATCAAATATATCTCCAAGTGAGTTAGGTAAGTTAGGTA 1718

Consensus acatcaaatatatctccaagtgagttaggtaagttaggta

kal TGAAGCAACACATTAATATCAACAAACTATTTTTTAAGTT 1635

yor TGAAGCAACACATTAATATCAACAAACTATTTTTTAAGTT 1635

jen TGAAGCAACACATTAATATCAACAAACTATTTTTTAAGTT 1635

cor TGAAGCAACACATTAATATCAACAAACTATTTTTTAAGTT 1635

man TGAAGCAACACATTAATATCAACAAACTATTTTTTAAGTT 1635

uni TGAAGCAACACATTAATATCAACAAACTATTTTTTAAGTT 1635

tall TGAAGCAACACATTAATATCAACAAACTATTTTTTAAGTT 1635

quil TGAAGCAACACATTAATATCAACAAACTATTTTTTAAGTT 1635

meri TGAAGCAACACATTAATATCAACAAACTATTTTTTAAGTT 1635

ref TGAAGCAACACATTAATATCAACAAgCTATTTTTTAAGTT 1758

Consensus tgaagcaacacattaatatcaacaa ctattttttaagtt

kal TACTTCGGTAATTTTCTTTAATAAAAAATATGTAAATGAG 1675

yor TACTTCGGTAATTTTCTTTAATAAAAAATATGTAAATGAG 1675

jen TACTTCGGTAATTTTCTTTAATAAAAAATATGTAAATGAG 1675

cor TACTTCGGTAATTTTCTTTAATAAAAAATATGTAAATGAG 1675

man TACTTCGGTAATTTTCTTTAATAAAAAATATGTAAATGAG 1675

uni TACTTCGGTAATTTTCTTTAATAAAAAATATGTAAATGAG 1675

tall TACTTCGGTAATTTTCTTTAATAAAAAATATGTAAATGAG 1675

quil TACTTCGGTAATTTTCTTTAATAAAAAATATGTAAATGAG 1675

meri TACTTCGGTAATTTTCTTTAATAAAAAATATGTAAATGAG 1675

ref TACTTCGGTAcTTTTCTTTAATAAAAAATATGTAAAcGAG 1798

Consensus tacttcggta ttttctttaataaaaaatatgtaaa gag

kal AGTTTAAAAATAAAAAA.TTGCTAATCGTATATATTAAGA 1714

yor AGTTTAAAAATAAAAAA.TTGCTAATCGTATATATTAAGA 1714

jen AGTTTAAAAATAAAAAA.TTGCTAATCGTATATATTAAGA 1714

cor AGTTTAAAAATAAAAAA.TTGCTAATCGTATATATTAAGA 1714

man AGTTTAAAAATAAAAAA.TTGCTAATCGTATATATTAAGA 1714

uni AGTTTAAAAATAAAAAA.TTGCTAATCGTATATATTAAGA 1714

tall AGTTTAAAAATAAAAAA.TTGCTAATCGTATATATTAAGA 1714

quil AGTTTAAAAATAAAAAA.TTGCTAATCGTATATATTAAGA 1714

meri AGTTTAAAAATAAAAAA.TTGCTAATCGTATATATTAAGA 1714

ref AGTTTAAAAATAAAAAAaTTGCTAATCGTATATATTAAGA 1838

Consensus agtttaaaaataaaaaa ttgctaatcgtatatattaaga

kal AGGAGTTATTACAAACTTATTCACATTCATGGGTAAGATA 1754

yor AGGAGTTATTACAAACTTATTCACATTCATGGGTAAGATA 1754

jen AGGAGTTATTACAAACTTATTCACATTCATGGGTAAGATA 1754

cor AGGAGTTATTACAAACTTATTCACATTCATGGGTAAGATA 1754

man AGGAGTTATTACAAACTTATTCACATTCATGGGTAAGATA 1754

uni AGGAGTTATTACAAACTTATTCACATTCATGGGTAAGATA 1754

tall AGGAGTTATTACAAACTTATTCACATTCATGGGTAAGATA 1754

quil AGGAGTTATTACAAACTTATTCACATTCATGGGTAAGATA 1754

meri AGGAGTTATTACAAACTTATTCACATTCATGGGTAAGATA 1754

ref AGGAGTTATTACAAACTTATTCACATTCATGGGTAAGATA 1878

Consensus aggagttattacaaacttattcacattcatgggtaagata

kal CAATCATTGTTATTCACCTAAAAGACCGTATTAATCTCAA 1794

yor CAATCATTGTTATTCACCTAAAAGACCGTATTAATCTCAA 1794

jen CAATCATTGTTATTCACCTAAAAGACCGTATTAATCTCAA 1794

cor CAATCATTGTTATTCACCTAAAAGACCGTATTAATCTCAA 1794

man CAATCATTGTTATTCACCTAAAAGACCGTATTAATCTCAA 1794

uni CAATCATTGTTATTCACCTAAAAGACCGTATTAATCTCAA 1794

tall CAATCATTGTTATTCACCTAAAAGACCGTATTAATCTCAA 1794

quil CAATCATTGTTATTCACCTAAAAGACCGTATTAATCTCAA 1794

meri CAATCATTGTTATTCACCTAAAAGACCGTATTAATCTCAA 1794

ref CAATCATTGTTATTCACCTAAAAGACCGTATTAATCTCAA 1918

Consensus caatcattgttattcacctaaaagaccgtattaatctcaa

kal AGTTAATGTTGAACTATTTTATTATATTTTGCTTTTTATA 1834

yor AGTTAATGTTGAACTATTTTATTATATTTTGCTTTTTATA 1834

jen AGTTAATGTTGAACTATTTTATTATATTTTGCTTTTTATA 1834

cor AGTTAATGTTGAACTATTTTATTATATTTTGCTTTTTATA 1834

man AGTTAATGTTGAACTATTTTATTATATTTTGCTTTTTATA 1834

uni AGTTAATGTTGAACTATTTTATTATATTTTGCTTTTTATA 1834

tall AGTTAATGTTGAACTATTTTATTATATTTTGCTTTTTATA 1834

quil AGTTAATGTTGAACTATTTTATTATATTTTGCTTTTTATA 1834

meri AGTTAATGTTGAACTATTTTATTATATTTTGCTTTTTATA 1834

ref AGTTAATGTTGAACTATTTTATTATATTTTGCTTTTTATA 1958

Consensus agttaatgttgaactattttattatattttgctttttata

kal AGAATATTTTGGACCATATTTTTAAGTTAATCGAATAATT 1874

yor AGAATATTTTGGACCATATTTTTAAGTTAATCGAATAATT 1874

jen AGAATATTTTGGACCATATTTTTAAGTTAATCGAATAATT 1874

cor AGAATATTTTGGACCATATTTTTAAGTTAATCGAATAATT 1874

man AGAATATTTTGGACCATATTTTTAAGTTAATCGAATAATT 1874

uni AGAATATTTTGGACCATATTTTTAAGTTAATCGAATAATT 1874

tall AGAATATTTTGGACCATATTTTTAAGTTAATCGAATAATT 1874

quil AGAATATTTTGGACCATATTTTTAAGTTAATCGAATAATT 1874

meri AGAATATTTTGGACCATATTTTTAAGTTAATCGAATAATT 1874

ref AGAATATTTTGGACCATATTTTTAAGTTAATCGAATAATT 1998

Consensus agaatattttggaccatatttttaagttaatcgaataatt

kal TGAATAAATTTTGTCTTACTAGATAAACGTGCGTTGCTTT 1914

yor TGAATAAATTTTGTCTTACTAGATAAACGTGCGTTGCTTT 1914

jen TGAATAAATTTTGTCTTACTAGATAAACGTGCGTTGCTTT 1914

cor TGAATAAATTTTGTCTTACTAGATAAACGTGCGTTGCTTT 1914

man TGAATAAATTTTGTCTTACTAGATAAACGTGCGTTGCTTT 1914

uni TGAATAAATTTTGTCTTACTAGATAAACGTGCGTTGCTTT 1914

tall TGAATAAATTTTGTCTTACTAGATAAACGTGCGTTGCTTT 1914

quil TGAATAAATTTTGTCTTACTAGATAAACGTGCGTTGCTTT 1914

meri TGAATAAATTTTaTCTTgCTAGATAAACGTGCGTTGCTTT 1914

ref TGAATAAATTTTaTCTTACTAGATgAACGTGCGTTGCTTT 2038

Consensus tgaataaatttt tctt ctagat aacgtgcgttgcttt

kal GCGCACATGAAAACCGACAGAAATATAAAATAACATGCAT 1954

yor GCGCACATGAAAACCGACAGAAATATAAAATAACATGCAT 1954

jen GCGCACATGAAAACCGACAGAAATATAAAATAACATGCAT 1954

cor GCGCACATGAAAACCGACAGAAATATAAAATAACATGCAT 1954

man GCGCACATGAAAACCGACAGAAATATAAAATAACATGCAT 1954

uni GCGCACATGAAAACCGACAGAAATATAAAATAACATGCAT 1954

tall GCGCACATGAAAACCGACAGAAATATAAAATAACATGCAT 1954

quil GCGCACATGAAAACCGACAGAAATATAAAATAACATGCAT 1954

meri GCGCACATGAAAACCGACAGAAATATAAAATAACATGCAT 1954

ref GCGCACATGgAAACCGACAGAAATATAAAATAACATGCAT 2078

Consensus gcgcacatg aaaccgacagaaatataaaataacatgcat

kal GATATAGAACTGGTAGAAGTTCCCATCAATCATAAATAAT 1994

yor GATATAGAACTGGTAGAAGTTCCCATCAATCATAAATAAT 1994

jen GATATAGAACTGGTAGAAGTTCCCATCAATCATAAATAAT 1994

cor GATATAGAACTGGTAGAAGTTCCCATCAATCATAAATAAT 1994

man GATATAGAACTGGTAGAAGTTCCCATCAATCATAAATAAT 1994

uni GATATAGAACTGGTAGAAGTTCCCATCAATCATAAATAAT 1994

tall GATATAGAACTGGTAGAAGTTCCCATCAATCATAAATAAT 1994

quil GATATAGAACTGGTAGAAGTTCCCATCAATCATAAATAAT 1994

meri GATATAGAACTGGTAGAAGTTCCCATCAATCATAAATAAT 1994

ref GATATAGAACTGGTAGAAGTTCCCATCAATCATAAATAAT 2118

Consensus gatatagaactggtagaagttcccatcaatcataaataat

kal GTACTTAATTTTTTTTGTGT..TACTAATAGTATTTCTTC 2032

yor GTACTTAATTTTTTTTGTGT..TACTAATAGTATTTCTTC 2032

jen GTACTTAATTTTTTTTGTGT..TACTAATAGTATTTCTTC 2032

cor GTACTTAATTTTTTTTGTGT..TACTAATAGTATTTCTTC 2032

man GTACTTAATTTTTTTTGTGT..TACTAATAGTATTTCTTC 2032

uni GTACTTAATTTTTTTTGTGT..TACTAATAGTATTTCTTC 2032

tall GTACTTAATTTTTTTTGTGTgtTACTAATAGTATTTCTTC 2034

quil GTACTTAATTTTTTTTGTGT..TACTAATAGTATTTCTTC 2032

meri GTACTTAATTTTTTTTtgtgt.TACTAATAGTATTTCTTC 2033

ref GTAtTTAATTTTTTTT........................ 2134

Consensus gta ttaatttttttt

kal ACCTTTTGGGTAAAGAACTAATCTCTTTTGGAATATGTAA 2072

yor ACCTTTTGGGTAAAGAACTAATCTCTTTTGGAATATGTAA 2072

jen ACCTTTTGGGTAAAGAACTAATCTCTTTTGGAATATGTAA 2072

cor ACCTTTTGGGTAAAGAACTAATCTCTTTTGGAATATGTAA 2072

man ACCTTTTGGGTAAAGAACTAATCTCTTTTGGAATATGTAA 2072

uni ACCTTTTGGGTAAAGAACTAATCTCTTTTGGAATATGTAA 2072

tall ACCTTTTGGGTAAAGAACTAATCTCTTTTGGAATATGTAA 2074

quil ACCTTTTGGGTAAAGAACTAATCTCTTTTGGAATATGTAA 2072

meri ACCTTTTGGGTAAAGAACTAATCTCTTTTGGAATATGTAA 2073

ref .........GTAAAGA.CTAATCTCTTTTaGAgTgTaTAA 2164

Consensus gtaaaga ctaatctctttt ga t t taa

kal TTGTTCCTGACAACAAATATCGCTCTTAAAAATCAAACAT 2112

yor TTGTTCCTGACAACAAATATCGCTCTTAAAAATCAAACAT 2112

jen TTGTTCCTGACAACAAATATCGCTCTTAAAAATCAAACAT 2112

cor TTGTTCCTGACAACAAATATCGCTCTTAAAAATCAAACAT 2112

man TTGTTCCTGACAACAAATATCGCTCTTAAAAATCAAACAT 2112

uni TTGTTCCTGACAACAAATATCGCTCTTAAAAATCAAACAT 2112

tall TTGTTCCTGACAACAAATATCGCTCTTAAAAATCAAACAT 2114

quil TTGTTCCTGACAACAAATATCGCTCTTAAAAATCAAACAT 2112

meri TTGTTCCTGACAACAAATATCGCTCTTAAAAATCAAACAT 2113

ref TTGTTCaTGACAACAgATATtatTCTTAAAAATCAAACAT 2204

Consensus ttgttc tgacaaca atat tcttaaaaatcaaacat

kal TTGACATTTTAGTCATCTTATAAATCTAGTATAATTTTAT 2152

yor TTGACATTTTAGTCATCTTATAAATCTAGTATAATTTTAT 2152

jen TTGACATTTTAGTCATCTTATAAATCTAGTATAATTTTAT 2152

cor TTGACATTTTAGTCATCTTATAAATCTAGTATAATTTTAT 2152

man TTGACATTTTAGTCATCTTATAAATCTAGTATAATTTTAT 2152

uni TTGACATTTTAGTCATCTTATAAATCTAGTATAATTTTAT 2152

tall TTGACATTTTAGTCATCTTATAAATCTAGTATAATTTTAT 2154

quil TTGACATTTTAGTCATCTTATAAATCTAGTATAATTTTAT 2152

meri TTGACATTTTAGTCATCTTATAAATCTAGTATAATTTTAT 2153

ref TTGAtATTTTAGTCATCTTATAAATtTAGTATAATTTTAT 2244

Consensus ttga attttagtcatcttataaat tagtataattttat

kal ACCACTAGA.................AATCTTGTATTTAA 2175

yor ACCACTAGA.................AATCTTGTATTTAA 2175

jen ACCACTAGA.................AATCTTGTATTTAA 2175

cor ACCACTAGA.................AATCTTGTATTTAA 2175

man ACCACTAGA.................AATCTTGTATTTAA 2175

uni ACCACTAGA.................AATCTTGTATTTAA 2175

tall ACCACTAGA.................AATCTTGTATTTAA 2177

quil ACCACTAGA.................AATCTTGTATTTAA 2175

meri ACCACTAGA.................AATCTTGTATTTAA 2176

ref AgCACTAGActaacagtttgttggtaAATCTTGTATTTAA 2284

Consensus a cactaga aatcttgtatttaa

kal TTATGTCACAACCAATATATTTTTCTAAAATACAAGAAAG 2215

yor TTATGTCACAACCAATATATTTTTCTAAAATACAAGAAAG 2215

jen TTATGTCACAACCAATATATTTTTCTAAAATACAAGAAAG 2215

cor TTATGTCACAACCAATATATTTTTCTAAAATACAAGAAAG 2215

man TTATGTCACAACCAATATATTTTTCTAAAATACAAGAAAG 2215

uni TTATGTCACAACCAATATATTTTTCTAAAATACAAGAAAG 2215

tall TTATGTCACAACCAATATATTTTTCTAAAATACAAGAAAG 2217

quil TTATGTCACAACCAATATATTTTTCTAAAATACAAGAAAG 2215

meri TTATGTCACAACCAATATATTTTTCTAAAATACAAGAAAG 2216

ref TTATGTCAtcACCAATATATTTTTCTAAAATACAAGAAAG 2324

Consensus ttatgtca accaatatatttttctaaaatacaagaaag

kal AGAGAGAGGGAGGGGGAGAAAATCAGTTTTCAAAATGTAG 2255

yor AGAGAGAGGGAGGGGGAGAAAATCAGTTTTCAAAATGTAG 2255

jen AGAGAGAGGGAGGGGGAGAAAATCAGTTTTCAAAATGTAG 2255

cor AGAGAGAGGGAGGGGGAGAAAATCAGTTTTCAAAATGTAG 2255

man AGAGAGAGGGAGGGGGAGAAAATCAGTTTTCAAAATGTAG 2255

uni AGAGAGAGGGAGGGGGAGAAAATCAGTTTTCAAAATGTAG 2255

tall AGAGAGAGGGAGGGGGAGAAAATCAGTTTTCAAAATGTAG 2257

quil AGAGAGAGGGAGGGGGAGAAAATCAGTTTTCAAAATGTAG 2255

meri AGAGAGAGGGAGGGGGAGAAAATCAGTTTTCAAAATGTAG 2256

ref AGAGAGAGGGAGGGGGAGAAAATCAGTTTTCAAAATtTAG 2364

Consensus agagagagggagggggagaaaatcagttttcaaaat tag

kal AAGCTATTATAAAGTTTAAATTTACAACTTGGTTATTCAT 2295

yor AAGCTATTATAAAGTTTAAATTTACAACTTGGTTATTCAT 2295

jen AAGCTATTATAAAGTTTAAATTTACAACTTGGTTATTCAT 2295

cor AAGCTATTATAAAGTTTAAATTTACAACTTGGTTATTCAT 2295

man AAGCTATTATAAAGTTTAAATTTACAACTTGGTTATTCAT 2295

uni AAGCTATTATAAAGTTTAAATTTACAACTTGGTTATTCAT 2295

tall AAGCTATTATAAAGTTTAAATTTACAACTTGGTTATTCAT 2297

quil AAGCTATTATAAAGTTTAAATTTACAACTTGGTTATTCAT 2295

meri AAGCTATTATAAAGTTTAAATTTACAACTTGGTTATTCAT 2296

ref AAGCTATTATAAAGTTTAAATTTACAACTTGcTTATTtAT 2404

Consensus aagctattataaagtttaaatttacaacttg ttatt at

kal GCAACAAATTTCGAGATTGTATAATT.TATATACATTTAT 2334

yor GCAACAAATTTCGAGATTGTATAATT.TATATACATTTAT 2334

jen GCAACAAATTTCGAGATTGTATAATT.TATATACATTTAT 2334

cor GCAACAAATTTCGAGATTGTATAATT.TATATACATTTAT 2334

man GCAACAAATTTCGAGATTGTATAATT.TATATACATTTAT 2334

uni GCAACAAATTTCGAGATTGTATAATT.TATATACATTTAT 2334

tall GCAACAAATTTCGAGATTGTATAATT.TATATACATTTAT 2336

quil GCAACAAATTTCGAGATTGTATAATT.TATATACATTTAT 2334

meri GCAACAAATTTCGAGATTGTATAATT.TATATACATTTAT 2335

ref GCAACAAATTTCGAGATTGTATAATTgTATATACATTTAT 2444

Consensus gcaacaaatttcgagattgtataatt tatatacatttat

kal CTCAAACTCACACCAAACATTATATATCTCTTCTTTATGC 2374

yor CTCAAACTCACACCAAACATTATATATCTCTTCTTTATGC 2374

jen CTCAAACTCACACCAAACATTATATATCTCTTCTTTATGC 2374

cor CTCAAACTCACACCAAACATTATATATCTCTTCTTTATGC 2374

man CTCAAACTCACACCAAACATTATATATCTCTTCTTTATGC 2374

uni CTCAAACTCACACCAAACATTATATATCTCTTCTTTATGC 2374

tall CTCAAACTCACACCAAACATTATATATCTCTTCTTTATGC 2376

quil CTCAAACTCACACCAAACATTATATATCTCTTCTTTATGC 2374

meri CTCAAACTCACACCAAACATTATATATCTCTTCTTTATGC 2375

ref CTCAAACTCACAtCAAACATTtTATATCTCTTCTTTATat 2484

Consensus ctcaaactcaca caaacatt tatatctcttctttat

kal ..TTTCTCCTTAAATCTTAGTGGCAGTGAATAGATGAAAT 2412

yor ..TTTCTCCTTAAATCTTAGTGGCAGTGAATAGATGAAAT 2412

jen ..TTTCTCCTTAAATCTTAGTGGCAGTGAATAGATGAAAT 2412

cor ..TTTCTCCTTAAATCTTAGTGGCAGTGAATAGATGAAAT 2412

man ..TTTCTCCTTAAATCTTAGTGGCAGTGAATAGATGAAAT 2412

uni ..TTTCTCCTTAAATCTTAGTGGCAGTGAATAGATGAAAT 2412

tall ..TTTCTCCTTAAATCTTAGTGGCAGTGAATAGATGAAAT 2414

quil ..TTTCTCCTTAAATCTTAGTGGCAGTGAATAGATGAAAT 2412

meri ..TTTCTCCTTAAATCTTAGTGGCAGTGAATAGATGAAAT 2413

ref gaTTTCTCCTTAAATCTTAGTGGCAGTGAATAGATGAAAT 2524

Consensus tttctccttaaatcttagtggcagtgaatagatgaaat

kal ATTTAGTTATAAATGTAAGAGATTATGATTTCAAGTCATG 2452

yor ATTTtGTTATAAATGTAAGAGATTATGATTTCAAGTaATG 2452

jen ATTTAGTTATAAATGTAAGAGATTATGATTTCAAGTCATG 2452

cor ATTTAGTTATAAATGTAAGAGATTATGATTTCAAGTCATG 2452

man ATTTAGTTATAAATGTAAGAGATTATGATTTCAAGTCATG 2452

uni ATTTAGTTATAAATGTAAGAGATTATGATTTCAAGTCATG 2452

tall ATTTAGTTATAAATGTAAGAGATTATGATTTCAAGTCATG 2454

quil ATTTAGTTATAAATGTAAGAGATTATGATTTCAAGTCATG 2452

meri ATTTtGTTATAAATGTAAGAGATTATGATTTCAAGTCATG 2453

ref ATTTtGTTATAAATGTAAGAGATTATGAgTTCAAGTaATG 2564

Consensus attt gttataaatgtaagagattatga ttcaagt atg

kal GGGAATCTCCTTGTAGAGAATAAATATTATATGAGTACTT 2492

yor GGGAATCTCCTTaTAGAGAATAAATATTATATGAGTACTT 2492

jen GGGAATCTCCTTGTAGAGAATAAATATTATATGAGTACTT 2492

cor GGGAATCTCCTTGTAGAGAATAAATATTATATGAGTACTT 2492

man GGGAATCTCCTTGTAGAGAATAAATATTATATGAGTACTT 2492

uni GGGAATCTCCTTGTAGAGAATAAATATTATATGAGTACTT 2492

tall GGGAATCTCCTTGTAGAGAATAAATATTATATGAGTACTT 2494

quil GGGAATCTCCTTGTAGAGAATAAATATTATATGAGTACTT 2492

meri GGGAATCTCCTTaTAGAGAATAAATATTATATGAGTACTT 2493

ref tGGAATCTCCTTaTAGgGAATAAATATTATATaAGTACTT 2604

Consensus ggaatctcctt tag gaataaatattatat agtactt

kal AAACTTAAAAGAGTGATACATTGAGAGTCGTATGTC.AAA 2531

yor AAACTTAAAAGAGTGATACATTGAGAGTCGTATGTC.AAA 2531

jen AAACTTAAAAGAGTGATACATTGAGAGTCGTATGTC.AAA 2531

cor AAACTTAAAAGAGTGATACATTGAGAGTCGTATGTC.AAA 2531

man AAACTTAAAAGAGTGATACATTGAGAGTCGTATGTC.AAA 2531

uni AAACTTAAAAGAGTGATACATTGAGAGTCGTATGTC.AAA 2531

tall AAACTTAAAAGAGTGATACATTGAGAGTCGTATGTC.AAA 2533

quil AAACTTAAAAGAGTGATACATTGAGAGTCGTATGTC.AAA 2531

meri AAACTTAAAAGAGTGATACATTGAGA.TCGTATGTC.AAA 2531

ref AAACTTgAA.GAGTGtTACA.......TCGTATGTCtAAg 2636

Consensus aaactt aa gagtg taca tcgtatgtc aa

kal ACTAGGGTATTACAATCAAATTTTATATCATTGTTTATTC 2571

yor ACTAGGGTATTACAATCAAATTTTATATCATTGTTTATTC 2571

jen ACTAGGGTATTACAATCAAATTTTATATCATTGTTTATTC 2571

cor ACTAGGGTATTACAATCAAATTTTATATCATTGTTTATTC 2571

man ACTAGGGTATTACAATCAAATTTTATATCATTGTTTATTC 2571

uni ACTAGGGTATTACAATCAAATTTTATATCATTGTTTATTC 2571

tall ACTAGGGTATTACAATCAAATTTTATATCATTGTTTATTC 2573

quil ACTAGGGTATTACAATCAAATTTTATATCATTGTTTATTC 2571

meri ACTAGGGTATTACAATCAAATTTTATATCATTGTTTATcC 2571

ref ACTAGGGTAT.ACAATtAtATTTTATATCATTGTTcATTt 2675

Consensus actagggtat acaat a attttatatcattgtt at

kal TGTCTCAAAACAAAAATATATAT..TGATAGTATATGAGT 2609

yor TGTCTCAAAACAAAAATATATATatTGATAGTATATGAGT 2611

jen TGTCTCAAAACAAAAATATATAT..TGATAGTATATGAGT 2609

cor TGTCTCAAAACAAAAATATATAT..TGATAGTATATGAGT 2609

man TGTCTCAAAACAAAAATATATAT..TGATAGTATATGAGT 2609

uni TGTCTCAAAACAAAAATATATAT..TGATAGTATATGAGT 2609

tall TGTCTCAAAACAAAAATATATAT..TGATAGTATATGAGT 2611

quil TGTCTCAAAACAAAAATATATAT..TGATAGTATATGAGT 2609

meri TGTCTCAAAACAAAAATATATAT..TGATAGTATATGAGT 2609

ref TGTCTtAAAAtAAAAATATATAT..cGATAaTgTATGAGT 2713

Consensus tgtct aaaa aaaaatatatat gata t tatgagt

kal TTTATATATTGTAAGTAAGAATCATCCATTATTATTTTCA 2649

yor TTTATATATTGTgAGTAAGAATCATCCATTATTATTTTCA 2651

jen TTTATATATTGTAAGTAAGAATCATCCATTATTATTTTCA 2649

cor TTTATATATTGTAAGTAAGAATCATCCATTATTATTTTCA 2649

man TTTATATATTGTAAGTAAGAATCATCCATTATTATTTTCA 2649

uni TTTATATATTGTAAGTAAGAATCATCCATTATTATTTTCA 2649

tall TTTATATATTGTAAGTAAGAATCATCCATTATTATTTTCA 2651

quil TTTATATATTGTAAGTAAGAATCATCCATTATTATTTTCA 2649

meri TTTATATATTGTgAGTAAGAATCgTCCATTATTATTTTCA 2649

ref TTTATATATTGTgAGTA..tATCATttATTATTATTTTCA 2751

Consensus tttatatattgt agta atc t attattattttca

kal ATCATCATTGACTCTTCAAAAAATTAATTAAGATGAGATG 2689

yor ATCATCATTGACTCTTCAAAAAATTAATTAAGATGAGATG 2691

jen ATCATCATTGACTCTTCAAAAAATTAATTAAGATGAGATG 2689

cor ATCATCATTGACTCTTCAAAAAATTAATTAAGATGAGATG 2689

man ATCATCATTGACTCTTCAAAAAATTAATTAAGATGAGATG 2689

uni ATCATCATTGACTCTTCAAAAAATTAATTAAGATGAGATG 2689

tall ATCATCATTGACTCTTCAAAAAATTAATTAAGATGAGATG 2691

quil ATCATCATTGACTCTTCAAAAAATTAATTAAGATGAGATG 2689

meri ATCATCATTGACTCTTCAAAAAATTAATTAAGATGAGATG 2689

ref ATCATCATTGACTCTTCAAAAAATTAATTAAGATGAGATG 2791

Consensus atcatcattgactcttcaaaaaattaattaagatgagatg

kal TGAATAATCCACTAACTATATATAAATCTTTATGGTATTG 2729

yor TGAATAATCCACTAACTATATATAAATCTTTATGcTATTG 2731

jen TGAATAATCCACTAACTATATATAAATCTTTATGGTATTG 2729

cor TGAATAATCCACTAACTATATATAAATCTTTATGGTATTG 2729

man TGAATAATCCACTAACTATATATAAATCTTTATGGTATTG 2729

uni TGAATAATCCACTAACTATATATAAATCTTTATGGTATTG 2729

tall TGAATAATCCACTAACTATATATAAATCTTTATGGTATTG 2731

quil TGAATAATCCACTAACTATATATAAATCTTTATGGTATTG 2729

meri TGAATAATCCACTAACTATATATAtAaaTcTtT....... 2722

ref TGAATAATCCACTAACTATATATAAATCTTTATGcTATcG 2831

Consensus tgaataatccactaactatatata a t t t

kal ATGCATAAATATCGCACTATTCTAAAAAACATTGAACACT 2769

yor ATGCATAAATATCtCACTATTCTAAAAAACATTGAACACT 2771

jen ATGCATAAATATCGCACTATTCTAAAAAACATTGAACACT 2769

cor ATGCATAAATATCGCACTATTCTAAAAAACATTGAACACT 2769

man ATGCATAAATATCGCACTATTCTAAAAAACATTGAACACT 2769

uni ATGCATAAATATCGCACTATTCTAAAAAACATTGAACACT 2769

tall ATGCATAAATATCGCACTATTCTAAAAAACATTGAACACT 2771

quil ATGCATAAATATCGCACTATTCTAAAAAACATTGAACACT 2769

meri ATGCATAAATATCGCACTATTCTAAAAAACATTGAACACT 2762

ref ATGCAaAAATATCtCACTATTCTAAAAAACATTGAACACT 2871

Consensus atgca aaatatc cactattctaaaaaacattgaacact

kal ATTGATGTTCATTTTTGATATTTTATGCTAATTGCTCTCT 2809

yor ATTGATGTTCATTTTTGATATTTTATGCTAATTGCTCTCT 2811

jen ATTGATGTTCATTTTTGATATTTTATGCTAATTGCTCTCT 2809

cor ATTGATGTTCATTTTTGATATTTTATGCTAATTGCTCTCT 2809

man ATTGATGTTCATTTTTGATATTTTATGCTAATTGCTCTCT 2809

uni ATTGATGTTCATTTTTGATATTTTATGCTAATTGCTCTCT 2809

tall ATTGATGTTCATTTTTGATATTTTATGCTAATTGCTCTCT 2811

quil ATTGATGTTCATTTTTGATATTTTATGCTAATTGCTCTCT 2809

meri ATTGATGTTCATTTTTGATATTTTATGCTAATTGCTCTCT 2802

ref ATTGATGTTCATTTTTGATATTTTATGCTAATTGCTCTCT 2911

Consensus attgatgttcatttttgatattttatgctaattgctctct

kal TAATAATTGACTTGGGTGGAAATGACTATCAATTAAATAT 2849

yor TAATAATTGACTTGGGTGaAAATGACTATCAATTAAATAT 2851

jen TAATAATTGACTTGGGTGGAAATGACTATCAATTAAATAT 2849

cor TAATAATTGACTTGGGTGGAAATGACTATCAATTAAATAT 2849

man TAATAATTGACTTGGGTGGAAATGACTATCAATTAAATAT 2849

uni TAATAATTGACTTGGGTGGAAATGACTATCAATTAAATAT 2849

tall TAATAATTGACTTGGGTGGAAATGACTATCAATTAAATAT 2851

quil TAATAATTGACTTGGGTGGAAATGACTATCAATTAAATAT 2849

meri TAATAATTGACTTGGGTGaAAATGACTATCAATTAAATAT 2842

ref TAATAATTGACTTGGGTGaAAATGACTATCAATTAAATAT 2951

Consensus taataattgacttgggtg aaatgactatcaattaaatat

kal TCACAATAATTCAACATTACAAAATTATTTAATATAATAG 2889

yor TCACAATAATTCAACATTACAAAATTATTTAATATAATAG 2891

jen TCACAATAATTCAACATTACAAAATTATTTAATATAATAG 2889

cor TCACAATAATTCAACATTACAAAATTATTTAATATAATAG 2889

man TCACAATAATTCAACATTACAAAATTATTTAATATAATAG 2889

uni TCACAATAATTCAACATTACAAAATTATTTAATATAATAG 2889

tall TCACAATAATTCAACATTACAAAATTATTTAATATAATAG 2891

quil TCACAATAATTCAACATTACAAAATTATTTAATATAATAG 2889

meri TCACAATAATTCAACATTACAAAATTATTTAATATAATAG 2882

ref TCACAATAATTCAACATTACAAAATTATTTAATATAATAG 2991

Consensus tcacaataattcaacattacaaaattatttaatataatag

kal TTAGCAATATTTAAGGATCAAAATGGAATCCAGCTAGATA 2929

yor TTAGCAATATTTAAGGATCAAAATGGAATCCAGCTAGATA 2931

jen TTAGCAATATTTAAGGATCAAAATGGAATCCAGCTAGATA 2929

cor TTAGCAATATTTAAGGATCAAAATGGAATCCAGCTAGATA 2929

man TTAGCAATATTTAAGGATCAAAATGGAATCCAGCTAGATA 2929

uni TTAGCAATATTTAAGGATCAAAATGGAATCCAGCTAGATA 2929

tall TTAGCAATATTTAAGGATCAAAATGGAATCCAGCTAGATA 2931

quil TTAGCAATATTTAAGGATCAAAATGGAATCCAGCTAGATA 2929

meri TTAGCAATATTTAAGGATCAAAATGGAATCCAGCTAGATA 2922

ref TTAGCAATATTTAAGGATCAAAATGGAATCCAGCTAGATA 3031

Consensus ttagcaatatttaaggatcaaaatggaatccagctagata

kal CCCGGTTCTATAAATAAGGATCATACCTACCCATTGGCAC 2969

yor CCCGGTTCTATAAATAAGGATCATACCTACCCATTGGCAC 2971

jen CCCGGTTCTATAAATAAGGATCATACCTACCCATTGGCAC 2969

cor CCCGGTTCTATAAATAAGGATCATACCTACCCATTGGCAC 2969

man CCCGGTTCTATAAATAAGGATCATACCTACCCATTGGCAC 2969

uni CCCGGTTCTATAAATAAGGATCATACCTACCCATTGGCAC 2969

tall CCCGGTTCTATAAATAAGGATCATACCTACCCATTGGCAC 2971

quil CCCGGTTCTATAAATAAGGATCATACCTACCCATTGGCAC 2969

meri CCCGGTTCTATAAATAAGGATCATACCTACCCATTGGCAC 2962

ref CCCGGTTCTATAAATAAcGATCATACCTACCCATTGGCAC 3071

Consensus cccggttctataaataa gatcatacctacccattggcac

kal AATCCAAAACACATTGTCACCTTAACACTCGTGGTGCTAA 3009

yor AATCCAAAACACATTGTCACCTTAACACTCGTGGTGCTAA 3011

jen AATCCAAAACACATTGTCACCTTAACACTCGTGGTGCTAA 3009

cor AATCCAAAACACATTGTCACCTTAACACTCGTGGTGCTAA 3009

man AATCCAAAACACATTGTCACCTTAACACTCGTGGTGCTAA 3009

uni AATCCAAAACACATTGTCACCTTAACACTCGTGGTGCTAA 3009

tall AATCCAAAACACATTGTCACCTTAACACTCGTGGTGCTAA 3011

quil AATCCAAAACACATTGTCACCTTAACACTCGTGGTGCTAA 3009

meri AATCCAAAACACATTGTCACCTTAACACTCGTGGTGCTAA 3002

ref AATCCAAAACACATTGTCACCTTAACACTCGTGGTGCTAA 3111

Consensus aatccaaaacacattgtcaccttaacactcgtggtgctaa

kal AGTTCTACAATGACTTTTAGCTACGAAAATTCAACAATGG 3049

yor AGTTCTACAATGACTTTTAGCTACGAAAATTCAACAATGG 3051

jen AGTTCTACAATGACTTTTAGCTACGAAAATTCAACAATGG 3049

cor AGTTCTACAATGACTTTTAGCTACGAAAATTCAACAATGG 3049

man AGTTCTACAATGACTTTTAGCTACGAAAATTCAACAATGG 3049

uni AGTTCTACAATGACnnnnnnnnnnnnnnnnnnnnnnnnnn 3049

tall AGTTCTACAATGACTTTTAGCTACGAAAATTCAACAATGG 3051

quil AGTTCTACAATGACTTTTAGCTACGAAAATTCAACAATGG 3049

meri AGTTCTACAATGACTTTTAGCTACGAAAATTCAACAATGG 3042

ref AGTTCTACAATGACTTTTAGCTACGAAAATTCAACAATGG 3151

Consensus agttctacaatgac

kal CCTCAAACTACAATAGCCACACAAATAGCCACA....... 3082

yor CCTCAAACTACAATAGCCACACAAATAGCCACA....... 3084

jen CCTCAAACTACAATAGCCACACAAATAGCCACA....... 3082

cor CCTCAAACTACAATAGCCACACAAATAGCCACA....... 3082

man CCTCAAACTACAATAGCCACACAAATAGCCACA....... 3082

uni nnnCAAACTACAATAGCCACACAAATAGCCACA....... 3082

tall CCTCAAACTACAATAGCCACACAAATAGCCACA....... 3084

quil CCTCAAACTACAATAGCCACACAAATAGCCACA....... 3082

meri CCTCAAACTACAATAGCCACACAAATAGCCACAcaaatag 3082

ref CCTCAAACTACAATAGCCACACAAATAGCCACAcaaatag 3191

Consensus caaactacaatagccacacaaatagccaca

kal .....ACAATGGTACCAAAGCCTTTAATCAAACACAAGTA 3117

yor .....ACAATGGcACCAAAGCCTTTAATCAAACACAAGTA 3119

jen .....ACAATGGTACCAAAGCCTTTAATCAAACACAAGTA 3117

cor .....ACAATGGTACCAAAGCCTTTAATCAAACACAAGTA 3117

man .....ACAATGGTACCAAAGCCTTTAATCAAACACAAGTA 3117

uni .....ACAATGGTACCAAAGCCTTTAATCAAACACAAGTA 3117

tall .....ACAATGGTACCAAAGCCTTTAATCAAACACAAGTA 3119

quil .....ACAATGGTACCAAAGCCTTTAATCAAACACAAGTA 3117

meri ccacaACAATGGcACCAAAGgCTTTAATCAAACACAAGTA 3122

ref ccacaACAATGGcACCAAAGgCTTTAATCAAACACAAGTA 3231

Consensus acaatgg accaaag ctttaatcaaacacaagta

kal GTTGTAGTTATGGTTCCTTTTCCAGCACAAGGCCATCTAA 3157

yor GTTGTAGTTATGGTTCCTTTTCCAGCACAAGGCCATCTAA 3159

jen GTTGTAGTTATGGTTCCTTTTCCAGCACAAGGCCATCTAA 3157

cor GTTGTAGTTATGGTTCCTTTTCCAGCACAAGGCCATCTAA 3157

man GTTGTAGTTATGGTTCCTTTTCCAGCACAAGGCCATCTAA 3157

uni GTTGTAGTTATGGTTCCTTTTCCAGCACAAGGCCATCTAA 3157

tall GTTGTAGTTATGGTTCCTTTTCCAGCACAAGGCCATCTAA 3159

quil GTTGTAGTTATGGTTCCTTTTCCAGCACAAGGCCATCTAA 3157

meri GTTGTAGTTATGGTTCCTTTTCCAGCACAAGGCCATCTAA 3162

ref GTTGTAGTTATGGTTCCTTTTCCAGCACAAGGCCATCTAA 3271

Consensus gttgtagttatggttccttttccagcacaaggccatctaa

kal ACCAACTTCTAAACTTCTCACGCTTAATCTTATCTCACAA 3197

yor ACCAACTTCTAcACTTCTCACGCTTAATCTTATCTCACAA 3199

jen ACCAACTTCTAAACTTCTCACGCTTAATCTTATCTCACAA 3197

cor ACCAACTTCTAAACTTCTCACGCTTAATCTTATCTCACAA 3197

man ACCAACTTCTAAACTTCTCACGCTTAATCTTATCTCACAA 3197

uni ACCAACTTCTAAACTTCTCACGCTTAATCTTATCTCACAA 3197

tall ACCAACTTCTAAACTTCTCACGCTTAATCTTATCTCACAA 3199

quil ACCAACTTCTAAACTTCTCACGCTTAATCTTATCTCACAA 3197

meri ACCAACTTCTAcACTTCTCACGCTTAATCTTATCTCACAA 3202

ref ACCAACTTCTAcACTTCTCACGCTTAATCTTATCTCACAA 3311

Consensus accaacttcta acttctcacgcttaatcttatctcacaa

kal CATTCATGTTCATTTTGTTGGCACTCCCACACACAACAAA 3237

yor CATTCATGTTCATTTTGTTGGCACTCCCAtACACAACAAA 3239

jen CATTCATGTTCATTTTGTTGGCACTCCCACACACAACAAA 3237

cor CATTCATGTTCATTTTGTTGGCACTCCCACACACAACAAA 3237

man CATTCATGTTCATTTTGTTGGCACTCCCACACACAACAAA 3237

uni CATTCATGTTCATTTTGTTGGCACTCCCACACACAACAAA 3237

tall CATTCATGTTCATTTTGTTGGCACTCCCACACACAACAAA 3239

quil CATTCATGTTCATTTTGTTGGCACTCCCACACACAACAAA 3237

meri CATTCATGTTCATTTTGTTGGCACTCCCACACACAACAAA 3242

ref CATTCATGTTCATTTTGTTGGCACTCCCACACACAACAAA 3351

Consensus cattcatgttcattttgttggcactccca acacaacaaa

kal CAAGCCATATTTCGTGCCCAAGGTTGGAACCCAAAATCCG 3277

yor CAAGCCATATTTCGTGCCCAAGGTTGGAACCCAAAATCCG 3279

jen CAAGCCATATTTCGTGCCCAAGGTTGGAACCCAAAATCCG 3277

cor CAAGCCATATTTCGTGCCCAAGGTTGGAACCCAAAATCCG 3277

man CAAGCCATATTTCGTGCCCAAGGTTGGAACCCAAAATCCG 3277

uni CAAGCCATATTTCGTGCCCAAGGTTGGAACCCAAAATCCG 3277

tall CAAGCCATATTTCGTGCCCAAGGTTGGAACCCAAAATCCG 3279

quil CAAGCCATATTTCGTGCCCAAGGTTGGAACCCAAAATCCG 3277

meri CAAGCCATATTTCGTGCCCAAGGTTGGAACCCAAAATCCG 3282

ref CAAGCCATATTTCGTGCCCAAGGTTGGAACCCAAAATCCG 3391

Consensus caagccatatttcgtgcccaaggttggaacccaaaatccg

kal TCGCTAATATTCATATTCATGATTTTGAGGTTCCTAGTTT 3317

yor TCGCTAATATTCATATTCATGATTTTGAGGTTCCTAGTTT 3319

jen TCGCTAATATTCATATTCATGATTTTGAGGTTCCTAGTTT 3317

cor TCGCTAATATTCATATTCATGATTTTGAGGTTCCTAGTTT 3317

man TCGCTAATATTCATATTCATGATTTTGAGGTTCCTAGTTT 3317

uni TCGCTAATATTCATATTCATGATTTTGAGGTTCCTAGTTT 3317

tall TCGCTAATATTCATATTCATGATTTTGAGGTTCCTAGTTT 3319

quil TCGCTAATATTCATATTCATGATTTTGAGGTTCCTAGTTT 3317

meri TCGCTAATATTCATATTCATGATTTTGAGGTTCCTAGTTT 3322

ref TCGCTAATATTCATATTCATGATTTTGAGGTTCCTAGTTT 3431

Consensus tcgctaatattcatattcatgattttgaggttcctagttt

kal TGCTTCACCTCTTCCCAATCCCAATAATGCAAAAACAAAA 3357

yor TGCTTCACCTCTTCCCAATCCCAATAATGCAAAAACAAAA 3359

jen TGCTTCACCTCTTCCCAATCCCAATAATGCAAAAACAAAA 3357

cor TGCTTCACCTCTTCCCAATCCCAATAATGCAAAAACAAAA 3357

man TGCTTCACCTCTTCCCAATCCCAATAATGCAAAAACAAAA 3357

uni TGCTTCACCTCTTCCCAATCCCAATAATGCAAAAACAAAA 3357

tall TGCTTCACCTCTTCCCAATCCCAATAATGCAAAAACAAAA 3359

quil TGCTTCACCTCTTCCCAATCCCAATAATGCAAAAACAAAA 3357

meri TGCTTCACCTCTTCCCAATCCCAATAATGCAAAAACAAAA 3362

ref TGCTTCACCTCTTCCCAATCCCAATAATGCAAAAACAAAA 3471

Consensus tgcttcacctcttcccaatcccaataatgcaaaaacaaaa

kal TTCCCATCTCATCTTTTACCTTCCTTTGAAGCTTCCTCAA 3397

yor TTCCCATCTCATCTTTTACCTTCCTTTGAAGCTTCCTCAA 3399

jen TTCCCATCTCATCTTTTACCTTCCTTTGAAGCTTCCTCAA 3397

cor TTCCCATCTCATCTTTTACCTTCCTTTGAAGCTTCCTCAA 3397

man TTCCCATCTCATCTTTTACCTTCCTTTGAAGCTTCCTCAA 3397

uni TTCCCATCTCATCTTTTACCTTCCTTTGAAGCTTCCTCAA 3397

tall TTCCCATCTCATnnnnnnnnnnnCTTTGAAGCTTCCTCAA 3399

quil TTCCCATCTCATCTTTTACCTTCCTTTGAAGCTTCCTCAA 3397

meri TTCCCATCTCATCTTTTACCTTCCTTTGAAGCTTCCTCAA 3402

ref TTCCCATCTCATCTTTTACCTTCCTTTGAAGCTTCCTCAA 3511

Consensus ttcccatctcat ctttgaagcttcctcaa

kal AGCTTAGGGAACCCGTGGCTAAACTCTTACTATCCCTTTC 3437

yor AGCTTAGGGAACCCGTGGCTAAACTCTTACTATCCCTTTC 3439

jen AGCTTAGGGAACCCGTGGCTAAACTCTTACTATCCCTTTC 3437

cor AGCTTAGGGAACCCGTGGCTAAACTCTTACTATCCCTTTC 3437

man AGCTTAGGGAACCCGTGGCTAAACTCTTACTATCCCTTTC 3437

uni AGCTTAGGGAACCCGTGGCTAAACTCTTACTATCCCTTTC 3437

tall AGCTTAGGGAACCCGTGGCTAAACTCTTACTATCCCTTTC 3439

quil AGCTTAGGGAACCCGTGGCTAAACTCTTACTATCCCTTTC 3437

meri AGCTTAGGGAACCCGTGGCTAAACTCTTACaATCCCTTTC 3442

ref AGCTTAGGGAACCCGTGGCTAAACTCTTACaATCCCTTTC 3551

Consensus agcttagggaacccgtggctaaactcttac atccctttc

kal ATCCGTGGCTAAAAGGGTCGTGGTTATTCATGACTCTCTC 3477

yor ATCCGTGGCTAAAAGGGTCGTGGTTATTCATGACTCcCTC 3479

jen ATCCGTGGCTAAAAGGGTCGTGGTTATTCATGACTCTCTC 3477

cor ATCCGTGGCTAAAAGGGTCGTGGTTATTCATGACTCTCTC 3477

man ATCCGTGGCTAAAAGGGTCGTGGTTATTCATGACTCTCTC 3477

uni ATCCGTGGCTAAAAGGGTCGTGGTTATTCATGACTCTCTC 3477

tall ATCCGTGGCTAAAAGGGTCGTGGTTATTCATGACTCTCTC 3479

quil ATCCGTGGCTAAAAGGGTCGTGGTTATTCATGACTCTCTC 3477

meri ATCCGTGGCTAAAAGGGTCGTGGTTATTCATGACTCcCTC 3482

ref ATCCGTGGCTAAAAGGGTCGTGGTTATTCATGACTCcCTC 3591

Consensus atccgtggctaaaagggtcgtggttattcatgactc ctc

kal ATGGCATGTGTTGTACAAGATGCTATTCATATAGCAAATT 3517

yor ATGGCATGTGTTGTACAAGATGCTATTCATATAGCAAATT 3519

jen ATGGCATGTGTTGTACAAGATGCTATTCATATAGCAAATT 3517

cor ATGGCATGTGTTGTACAAGATGCTATTCATATAGCAAATT 3517

man ATGGCATGTGTTGTACAAGATGCTATTCATATAGCAAATT 3517

uni ATGGCATGTGTTGTACAAGATGCTATTCATATAGCAAATT 3517

tall ATGGCATGTGTTGTACAAGATGCTATTCATATAGCAAATT 3519

quil ATGGCATGTGTTGTACAAGATGCTATTCATATAGCAAATT 3517

meri ATGGCATGTGTTGTACAAGATGCTATTCATATAGCAAATT 3522

ref ATGGCATGTGTTGTACAAGATGCTATTCATATAGCAAATT 3631

Consensus atggcatgtgttgtacaagatgctattcatatagcaaatt

kal GTGAGAGCTACACTTTTCATAGTGTCTCTGCCTTTACCAT 3557

yor GTGAGAGCTACACTTTTCATAGTGTCTCTGCCTTTACCAT 3559

jen GTGAGAGCTACACTTTTCATAGTGTCTCTGCCTTTACCAT 3557

cor GTGAGAGCTACACTTTTCATAGTGTCTCTGCCTTTACCAT 3557

man GTGAGAGCTACACTTTTCATAGTGTCTCTGCCTTTACCAT 3557

uni GTGAGAGCTACACTTTTCATAGTGTCTCTGCCTTTACCAT 3557

tall GTGAGAGCTACACTTTTCATAGTGTCTCTGCCTTTACCAT 3559

quil GTGAGAGCTACACTTTTCATAGTGTCTCTGCCTTTACCAT 3557

meri GTGAGAGCTACACTTTTCATAGTGTCTCTGCCTTTACCAT 3562

ref GTGAGAGCTACACTTTTCATAGTGTCTCTGCCTTTACCAT 3671

Consensus gtgagagctacacttttcatagtgtctctgcctttaccat

kal GTTCTTGTATTTTTGGGATGCAATGGGAAAAGGAAAAGAA 3597

yor GTTCTTGTATTTTTGGGATGCAATGGGAAAAGGAAAAGAA 3599

jen GTTCTTGTATTTTTGGGATGCAATGGGAAAAGGAAAAGAA 3597

cor GTTCTTGTATTTTTGGGATGCAATGGGAAAAGGAAAAGAA 3597

man GTTCTTGTATTTTTGGGATGCAATGGGAAAAGGAAAAGAA 3597

uni GTTCTTGTATTTTTGGGATGCAATGGGAAAAGGAAAAGAA 3597

tall GTTCTTGTATTTTTGGGATGCAATGGGAAAAGGAAAAGAA 3599

quil GTTCTTGTATTTTTGGGATGCAATGGGAAAAGGAAAAGAA 3597

meri GTTCTTGTATTTTTGGGATGCAATGGGAAAAGGAAAAGAA 3602

ref GTTCTTGTATTTTTGGGATGCAATGGGAAAAGGAAAAGAA 3711

Consensus gttcttgtatttttgggatgcaatgggaaaaggaaaagaa

kal AATGAAAATGAAAAAGCTCTTAGAGGAAAAAATTCTCATG 3637

yor AATGAAAATGAAAAAGCTCTTAGAGGAAAAAATTCTCATG 3639

jen AATGAAAATGAAAAAGCTCTTAGAGGAAAAAATTCTCATG 3637

cor AATGAAAATGAAAAAGCTCTTAGAGGAAAAAATTCTCATG 3637

man AATGAAAATGAAAAAGCTCTTAGAGGAAAAAATTCTCATG 3637

uni AATGAAAATGAAAAAGCTCTTAGAGGAAAAAATTCTCATG 3637

tall AATGAAAATGAAAAAGCTCTTAGAGGAAAAAATTCTCATG 3639

quil AATGAAAATGAAAAAGCTCTTAGAGGAAAAAATTCTCATG 3637

meri AATGAAAATGAAAAAGCTgTTAGAGGAAAAAATTCTCATG 3642

ref AATGAAAATGAAAAAGCTCTTAGAGGAAAAAATTCTCATG 3751

Consensus aatgaaaatgaaaaagct ttagaggaaaaaattctcatg

kal ATCATTATAATATCATCCCAGAAGTTCCTTCTTTGGAGGG 3677

yor ATCATTATAATATCATCCCAGAAGTTCCTTCTTTGGAGGG 3679

jen ATCATTATAATATCATCCCAGAAGTTCCTTCTTTGGAGGG 3677

cor ATCATTATAATATCATCCCAGAAGTTCCTTCTTTGGAGGG 3677

man ATCATTATAATATCATCCCAGAAGTTCCTTCTTTGGAGGG 3677

uni ATCATTATAATATCATCCCAGAAGTTCCTTCTTTGGAGGG 3677

tall ATCATTATAATATCATCCCAGAAGTTCCTTCTTTGGAGGG 3679

quil ATCATTATAATATCATCCCAGAAGTTCCTTCTTTGGAGGG 3677

meri ATCATTATAATATCATCCCAGAAGTTCCTTCTTTGGAGGG 3682

ref ATCATTATAATATCATCCCAGAAGTTCCTTCTTTGGAGGG 3791

Consensus atcattataatatcatcccagaagttccttctttggaggg

kal TTGTTTCTCAACCCAATTCATTGATTTCATCACTTCACAA 3717

yor TTGTTTCTCAACCCAATTCATTGATTTCATCACTTCACAA 3719

jen TTGTTTCTCAACCCAATTCATTGATTTCATCACTTCACAA 3717

cor TTGTTTCTCAACCCAATTCATTGATTTCATCACTTCACAA 3717

man TTGTTTCTCAACCCAATTCATTGATTTCATCACTTCACAA 3717

uni TTGTTTCTCAACCCAATTCATTGATTTCATCACTTCACAA 3717

tall TTGTTTCTCAACCCAATTCATTGATTTCATCACTTCACAA 3719

quil TTGTTTCTCAACCCAATTCATTGATTTCATCACTTCACAA 3717

meri TTGTTTCTCAACCCAATTCATTGATTTCATCACTTCACAA 3722

ref TTGTTTCTCAACCCAATTCATTGATTTCATCACTTCACAA 3831

Consensus ttgtttctcaacccaattcattgatttcatcacttcacaa

kal TATGAGTTCCATAAATTTAGCAAAGGTGCAATTTACAACA 3757

yor TATGAGTTCCATAAATTTAGCAAAGGTGCAATTTACAACA 3759

jen TATGAGTTCCATAAATTTAGCAAAGGTGCAATTTACAACA 3757

cor TATGAGTTCCATAAATTTAGCAAAGGTGCAATTTACAACA 3757

man TATGAGTTCCATAAATTTAGCAAAGGTGCAATTTACAACA 3757

uni TATGAGTTCCATAAATTTAGCAAAGGTGCAATTTACAACA 3757

tall TATGAGTTCCATAAATTTAGCAAAGGTGCAATTTACAACA 3759

quil TATGAGTTCCATAAATTTAGCAAAGGTGCAATTTACAACA 3757

meri TATGAGTTCCATAAATTTAGCAAAGGTGCAATTTACAACA 3762

ref TATGAGTTCCATAAATTTAGCAAAGGTGCAATTTACAACA 3871

Consensus tatgagttccataaatttagcaaaggtgcaatttacaaca

kal CAACAAGGGCAATTGAAAGTCCTTATATGGAGTTAATAGA 3797

yor CAACAAGGGCAATTGAAAGTCCTTATATGGAGTTAATAGA 3799

jen CAACAAGGGCAATTGAAAGTCCTTATATGGAGTTAATAGA 3797

cor CAACAAGGGCAATTGAAAGTCCTTATATGGAGTTAATAGA 3797

man CAACAAGGGCAATTGAAAGTCCTTATATGGAGTTAATAGA 3797

uni CAACAAGGGCAATTGAAAGTCCTTATATGGAGTTAATAGA 3797

tall CAACAAGGGCAATTGAAAGTCCTTATATGGAGTTAATAGA 3799

quil CAACAAGGGCAATTGAAAGTCCTTATATGGAGTTAATAGA 3797

meri CAACAAGGGCAATTGAAAGTCCTTATATGGAGTTAATAGA 3802

ref CAACAAGGGCAATTGAAAGTCCTTATATGGAGTTAATAGA 3911

Consensus caacaagggcaattgaaagtccttatatggagttaataga

kal AAGTATAGTTACTACAAAATCTCATTGGGCTTTAGGCCCC 3837

yor AAGTATAGTTgCTACAAAATCTCATTGGGCTTTAGGCCCC 3839

jen AAGTATAGTTACTACAAAATCTCATTGGGCTTTAGGCCCC 3837

cor AAGTATAGTTACTACAAAATCTCATTGGGCTTTAGGCCCC 3837

man AAGTATAGTTACTACAAAATCTCATTGGGCTTTAGGCCCC 3837

uni AAGTATAGTTACTACAAAATCTCATTGGGCTTTAGGCCCC 3837

tall AAGTATAGTTACTACAAAATCTCATTGGGCTTTAGGCCCC 3839

quil AAGTATAGTTACTACAAAATCTCATTGGGCTTTAGGCCCC 3837

meri AAGTATAGTTACTACAAAATCTCATTGGGCTTTAGGCCCC 3842

ref AAGTATAGTTACTACAAAATCTCATTGGGCTTTAGGCCCC 3951

Consensus aagtatagtt ctacaaaatctcattgggctttaggcccc

kal TTCAACCCTTTATCTATAGAGCAAAAAAGCTATAAAGGTA 3877

yor TTCAACCCTTTATCTATAGAGCAAAAAAGCTATAAAGGTA 3879

jen TTCAACCCTTTATCTATAGAGCAAAAAAGCTATAAAGGTA 3877

cor TTCAACCCTTTATCTATAGAGCAAAAAAGCTATAAAGGTA 3877

man TTCAACCCTTTATCTATAGAGCAAAAAAGCTATAAAGGTA 3877

uni TTCAACCCTTTATCTATAGAGCAAAAAAGCTATAAAGGTA 3877

tall TTCAACCCTTTATCTATAGAGCAAAAAAGCTATAAAGGTA 3879

quil TTCAACCCTTTATCTATAGAGCAAAAAAGCTATAAAGGTA 3877

meri TTCAACCCTTTATCTATAGAGCAAAAAAGCTATAAAGGTA 3882

ref TTCAACCCTTTATCTATAGAGCAAAAAAGCTATAAAGGTA 3991

Consensus ttcaaccctttatctatagagcaaaaaagctataaaggta

kal AAAAGCACTTTAGCATGGAATGGCTTGACAAGAAAAGCCC 3917

yor AAAAGCACTTTAGCATGGAATGGCTTGACAAGAAAAGCCC 3919

jen AAAAGCACTTTAGCATGGAATGGCTTGACAAGAAAAGCCC 3917

cor AAAAGCACTTTAGCATGGAATGGCTTGACAAGAAAAGCCC 3917

man AAAAGCACTTTAGCATGGAATGGCTTGACAAGAAAAGCCC 3917

uni AAAAGCACTTTAGCATGGAATGGCTTGACAAGAAAAGCCC 3917

tall AAAAGCACTTTAGCATGGAATGGCTTGACAAGAAAAGCCC 3919

quil AAAAGCACTTTAGCATGGAATGGCTTGACAAGAAAAGCCC 3917

meri AAAAGCACTTTAGCATGGAATGGCTTGACAAGAAAAGCCC 3922

ref AAAAGCACTTTAGCATGGAATGGCTTGACAAGAAAAGCCC 4031

Consensus aaaagcactttagcatggaatggcttgacaagaaaagccc

kal AAAATCAGTTATATATGTGTCTTTTGGAACCACTGTAGCA 3957

yor AAAATCAGTTATATATGTGTCTTTTGGAACCACTGTAGCA 3959

jen AAAATCAGTTATATATGTGTCTTTTGGAACCACTGTAGCA 3957

cor AAAATCAGTTATATATGTGTCTTTTGGAACCACTGTAGCA 3957

man AAAATCAGTTATATATGTGTCTTTTGGAACCACTGTAGCA 3957

uni AAAATCAGTTATATATGTGTCTTTTGGAACCACTGTAGCA 3957

tall AAAATCAGTTATATATGTGTCTTTTGGAACCACTGTAGCA 3959

quil AAAATCAGTTATATATGTGTCTTTTGGAACCACTGTAGCA 3957

meri AAAATCAGTTATATATGTGTCTTTTGGAACCACTGTAGCA 3962

ref AAAATCAGTTATATATGTGTCTTTTGGAACCACTGTAGCA 4071

Consensus aaaatcagttatatatgtgtcttttggaaccactgtagca

kal TTCTTAGATGAACAAATCAAAGAGCTTGCAATTGGGTTGG 3997

yor TTCTTAGATGAACAAATCAAAGAGCTTGCAATTGGGTTGG 3999

jen TTCTTAGATGAACAAATCAAAGAGCTTGCAATTGGGTTGG 3997

cor TTCTTAGATGAACAAATCAAAGAGCTTGCAATTGGGTTGG 3997

man TTCTTAGATGAACAAATCAAAGAGCTTGCAATTGGGTTGG 3997

uni TTCTTAGATGAACAAATCAAAGAGCTTGCAATTGGGTTGG 3997

tall TTCTTAGATGAACAAATCAAAGAGCTTGCAATTGGGTTGG 3999

quil TTCTTAGATGAACAAATCAAAGAGCTTGCAATTGGGTTGG 3997

meri TTCTTAGATGAgCAAATCgAAGAGCTTGCAATTGGGTTGG 4002

ref TTCTTAGATGAACAAATCAAAGAGCTTGCAATTGGGTTGG 4111

Consensus ttcttagatga caaatc aagagcttgcaattgggttgg

kal AACAAAGCAAGCAAAATTTCATTTGGGTCATAAGAGATGC 4037

yor AACAAAGCAAGCAAAATTTCATTTGGGTCATAAGAGATGC 4039

jen AACAAAGCAAGCAAAATTTCATTTGGGTCATAAGAGATGC 4037

cor AACAAAGCAAGCAAAATTTCATTTGGGTCATAAGAGATGC 4037

man AACAAAGCAAGCAAAATTTCATTTGGGTCATAAGAGATGC 4037

uni AACAAAGCAAGCAAAATTTCATTTGGGTCATAAGAGATGC 4037

tall AACAAAGCAAGCAAAATTTCATTTGGGTCATAAGAGATGC 4039

quil AACAAAGCAAGCAAAATTTCATTTGGGTCATAAGAGATGC 4037

meri AACAAAGCAAGCAAAATTTCATTTGGGTCATAAGAGATGC 4042

ref AACAAAGCAAGCAAAATTTCATTTGGGTCATAAGAGATGC 4151

Consensus aacaaagcaagcaaaatttcatttgggtcataagagatgc

kal TGATAAAGGTGATGTTTTTGATGTTGATGAAGTGAGAAGA 4077

yor TGATAAAGGTGATGTTTTTGATGTTGATGAAGTGAGAAGA 4079

jen TGATAAAGGTGATGTTTTTGATGTTGATGAAGTGAGAAGA 4077

cor TGATAAAGGTGATGTTTTTGATGTTGATGAAGTGAGAAGA 4077

man TGATAAAGGTGATGTTTTTGATGTTGATGAAGTGAGAAGA 4077

uni TGATAAAGGTGATGTTTTTGATGTTGATGAAGTGAGAAGA 4077

tall TGATAAAGGTGATGTTTTTGATGTTGATGAAGTGAGAAGA 4079

quil TGATAAAGGTGATGTTTTTGATGTTGATGAAGTGAGAAGA 4077

meri TGATAAAGGTGATGTTTTTGATGTTGATGAAGTGAGAAGA 4082

ref TGATAAAGGTGATGTTTTTGATGTTGATGAAGTGAGAAGA 4191

Consensus tgataaaggtgatgtttttgatgttgatgaagtgagaaga

kal GTTGAACTTCCAAAAGGGTTTGAAGAGAGAATTGAGGTAG 4117

yor GTTGAACTTCCAAAAGGGTTTGAAGAGAGAATTGAGGTAG 4119

jen GTTGAACTTCCAAAAGGGTTTGAAGAGAGAATTGAGGTAG 4117

cor GTTGAACTTCCAAAAGGGTTTGAAGAGAGAATTGAGGTAG 4117

man GTTGAACTTCCAAAAGGGTTTGAAGAGAGAATTGAGGTAG 4117

uni GTTGAACTTCCAAAAGGGTTTGAAGAGAGAATTGAGGTAG 4117

tall GTTGAACTTCCAAAAGGGTTTGAAGAGAGAATTGAGGTAG 4119

quil GTTGAACTTCCAAAAGGGTTTGAAGAGAGAATTGAGGTAG 4117

meri GTTGAACTTCCAAAAGGGTTTGAAGAGAGAATTGAGGTAG 4122

ref GTTGAACTTCCAAAAGGGTTTGAAGAGAGAATTGAGGTAG 4231

Consensus gttgaacttccaaaagggtttgaagagagaattgaggtag

kal AAGGATTTGGGTTAATTCTAAGGAATTGGGCCCCACAATT 4157

yor AAGGATTTGGGTTAATTCTAAGGAATTGGGCCCCACAATT 4159

jen AAGGATTTGGGTTAATTCTAAGGAATTGGGCCCCACAATT 4157

cor AAGGATTTGGGTTAATTCTAAGGAATTGGGCCCCACAATT 4157

man AAGGATTTGGGTTAATTCTAAGGAATTGGGCCCCACAATT 4157

uni AAGGATTTGGGTTAATTCTAAGGAATTGGGCCCCACAATT 4157

tall AAGGATTTGGGTTAATTCTAAGGAATTGGGCCCCACAATT 4159

quil AAGGATTTGGGTTAATTCTAAGGAATTGGGCCCCACAATT 4157

meri AAGGATTTGGGTTAATTCTAAGGAATTGGGCCCCACAATT 4162

ref AAGGATTTGGGTTAATTCTAAGGAATTGGGCCCCACAATT 4271

Consensus aaggatttgggttaattctaaggaattgggccccacaatt

kal GGAGATTCTAAGTCACCCTTCAATAGGTGGGTTTATGAGC 4197

yor GGAGATTCTAAGTCACCCTTCAATAGGTGGGTTTATGAGC 4199

jen GGAGATTCTAAGTCACCCTTCAATAGGTGGGTTTATGAGC 4197

cor GGAGATTCTAAGTCACCCTTCAATAGGTGGGTTTATGAGC 4197

man GGAGATTCTAAGTCACCCTTCAATAGGTGGGTTTATGAGC 4197

uni GGAGATTCTAAGTCACCCTTCAATAGGTGGGTTTATGAGC 4197

tall GGAGATTCTAAGTCACCCTTCAATAGGTGGGTTTATGAGC 4199

quil GGAGATTCTAAGTCACCCTTCAATAGGTGGGTTTATGAGC 4197

meri GGAGATTCTAAGTCACCCTTCAATAGGTGGGTTTATGAGC 4202

ref GGAGATTCTAAGTCACCCTTCAATAGGTGGGTTTATGAGC 4311

Consensus ggagattctaagtcacccttcaataggtgggtttatgagc

kal CATTGTGGTTGGAATTCTTGCATGGAAAGCATCACAATGG 4237

yor CATTGTGGTTGGAATTCTTGCATGGAAAGCATCACAATGG 4239

jen CATTGTGGTTGGAATTCTTGCATGGAAAGCATCACAATGG 4237

cor CATTGTGGTTGGAATTCTTGCATGGAAAGCATCACAATGG 4237

man CATTGTGGTTGGAATTCTTGCATGGAAAGCATCACAATGG 4237

uni CATTGTGGTTGGAATTCTTGCATGGAAAGCATCACAATGG 4237

tall CATTGTGGTTGGAATTCTTGCATGGAAAGCATCACAATGG 4239

quil CATTGTGGTTGGAATTCTTGCATGGAAAGCATCACAATGG 4237

meri CATTGTGGTTGGAATTCTTGCATGGAAAGCATCACAATGG 4242

ref CATTGTGGTTGGAATTCTTGCATGGAAAGCATCACAATGG 4351

Consensus cattgtggttggaattcttgcatggaaagcatcacaatgg

kal GTGTGCCAATAGCAGCATGGCCTATGCATTCTGATCAACC 4277

yor GTGTGCCAATAGCAGCATGGCCTATGCATTCTGATCAACC 4279

jen GTGTGCCAATAGCAGCATGGCCTATGCATTCTGATCAACC 4277

cor GTGTGCCAATAGCAGCATGGCCTATGCATTCTGATCAACC 4277

man GTGTGCCAATAGCAGCATGGCCTATGCATTCTGATCAACC 4277

uni GTGTGCCAATAGCAGCATGGCCTATGCATTCTGATCAACC 4277

tall GTGTGCCAATAGCAGCATGGCCTATGCATTCTGATCAACC 4279

quil GTGTGCCAATAGCAGCATGGCCTATGCATTCTGATCAACC 4277

meri GTGTGCCAATAGCAGCATGGCCTATGCATTCTGATCAACC 4282

ref GTGTGCCAATAGCAGCATGGCCTATGCATTCTGATCAACC 4391

Consensus gtgtgccaatagcagcatggcctatgcattctgatcaacc

kal AAGGAACAGTGTTTTGGTGACAGAGTTATTGAAGGTTGGT 4317

yor AAGGAACAGTGTTTTGGTGACAGAGTTATTGAAGGTTGGT 4319

jen AAGGAACAGTGTTTTGGTGACAGAGTTATTGAAGGTTGGT 4317

cor AAGGAACAGTGTTTTGGTGACAGAGTTATTGAAGGTTGGT 4317

man AAGGAACAGTGTTTTGGTGACAGAGTTATTGAAGGTTGGT 4317

uni AAGGAACAGTGTTTTGGTGACAGAGTTATTGAAGGTTGGT 4317

tall AAGGAACAGTGTTTTGGTGACAGAGTTATTGAAGGTTGGT 4319

quil AAGGAACAGTGTTTTGGTGACAGAGTTATTGAAGGTTGGT 4317

meri AAGGAACAGTGTTTTGGTGACAGAGTTATTGAAGGTTGGT 4322

ref AAGGAACAGTGTTTTGGTGACAGAGTTATTGAAGGTTGGT 4431

Consensus aaggaacagtgttttggtgacagagttattgaaggttggt

kal TTGGTTGTTAAGGATTGGTCCCAAAGAGATGAGTTGGTGA 4357

yor TTGGTTGTTAAGGATTGGTCCCAAAGAGATGAGTTGGTGA 4359

jen TTGGTTGTTAAGGATTGGTCCCAAAGAGATGAGTTGGTGA 4357

cor TTGGTTGTTAAGGATTGGTCCCAAAGAGATGAGTTGGTGA 4357

man TTGGTTGTTAAGGATTGGTCCCAAAGAGATGAGTTGGTGA 4357

uni TTGGTTGTTAAGGATTGGTCCCAAAGAGATGAGTTGGTGA 4357

tall TTGGTTGTTAAGGATTGGTCCCAAAGAGATGAGTTGGTGA 4359

quil TTGGTTGTTAAGGATTGGTCCCAAAGAGATGAGTTGGTGA 4357

meri TTGGTTGTTAAGGATTGGTCCCAAAGAGATGAGTTGGTGA 4362

ref TTGGTTGTTAAGGATTGGTCCCAAAGAGATGAGTTGGTGA 4471

Consensus ttggttgttaaggattggtcccaaagagatgagttggtga

kal CAGCATCAAATGTTCAAAATGCTGTCAATAGATTGATGGC 4397

yor CAGCATCAAATGTTCAAAATGCTGTCAAgAGATTGATGGC 4399

jen CAGCATCAAATGTTCAAAATGCTGTCAATAGATTGATGGC 4397

cor CAGCATCAAATGTTCAAAATGCTGTCAATAGATTGATGGC 4397

man CAGCATCAAATGTTCAAAATGCTGTCAATAGATTGATGGC 4397

uni CAGCATCAAATGTTCAAAATGCTGTCAATAGATTGATGGC 4397

tall CAGCATCAAATGTTCAAAATGCTGTCAATAGATTGATGGC 4399

quil CAGCATCAAATGTTCAAAATGCTGTCAATAGATTGATGGC 4397

meri CAGCATCAAATGTTCAAAATGCTGTCAATAGATTGATGGC 4402

ref CAGCATCAAATGTTCAAAATGCTGTCAAgAGATTGATGGC 4511

Consensus cagcatcaaatgttcaaaatgctgtcaa agattgatggc

kal TACAAAAGAAGGTGATGAGATGAGAGAAAGGGTAATGAAT 4437

yor TACAAAAGAAGGTGATGAGATGAGAGAAAGGGTAATGAAT 4439

jen TACAAAAGAAGGTGATGAGATGAGAGAAAGGGTAATGAAT 4437

cor TACAAAAGAAGGTGATGAGATGAGAGAAAGGGTAATGAAT 4437

man TACAAAAGAAGGTGATGAGATGAGAGAAAGGGTAATGAAT 4437

uni TACAAAAGAAGGTGATGAGATGAGAGAAAGGGTAATGAAT 4437

tall TACAAAAGAAGGTGATGAGATGAGAGAAAGGGTAATGAAT 4439

quil TACAAAAGAAGGTGATGAGATGAGAGAAAGGGTAATGAAT 4437

meri TACAAAAGAAGGTGATGAGATGAGAGAAAGGGTAATGAAT 4442

ref TACAAAAGAAGGTGATGAGATGAGAGAAAGGGTAATGAAT 4551

Consensus tacaaaagaaggtgatgagatgagagaaagggtaatgaat

kal CTCAAAAGTGTTATTTTCAAGTCTATGGATGAAGGAGGTG 4477

yor CTCAAAAGTGTTATTTTCAAGTCTATGGATGAAGGAGGTG 4479

jen CTCAAAAGTGTTATTTTCAAGTCTATGGATGAAGGAGGTG 4477

cor CTCAAAAGTGTTATTTTCAAGTCTATGGATGAAGGAGGTG 4477

man CTCAAAAGTGTTATTTTCAAGTCTATGGATGAAGGAGGTG 4477

uni CTCAAAAGTGTTATTTTCAAGTCTATGGATGAAGGAGGTG 4477

tall CTCAAAAGTGTTATTTTCAAGTCTATGGATGAAGGAGGTG 4479

quil CTCAAAAGTGTTATTTTCAAGTCTATGGATGAAGGAGGTG 4477

meri CTCAAAAGTGTTATTTTCAAGTCTATGGATGAAGGAGGTG 4482

ref CTCAAAAGTGTTATTTTCAAGTCTATGGATGAAGGAGGTG 4591

Consensus ctcaaaagtgttattttcaagtctatggatgaaggaggtg

kal TTTCTTCTGTGGAAATGAATTCTTTCATTGCTCATATAAC 4517

yor TTTCTTCTGTGGAAATGAATTCTTTCATTGCTCATATAAC 4519

jen TTTCTTCTGTGGAAATGAATTCTTTCATTGCTCATATAAC 4517

cor TTTCTTCTGTGGAAATGAATTCTTTCATTGCTCATATAAC 4517

man TTTCTTCTGTGGAAATGAATTCTTTCATTGCTCATATAAC 4517

uni TTTCTTCTGTGGAAATGAATTCTTTCATTGCTCATATAAC 4517

tall TTTCTTCTGTGGAAATGAATTCTTTCATTGCTCATATAAC 4519

quil TTTCTTCTGTGGAAATGAATTCTTTCATTGCTCATATAAC 4517

meri TTTCTTCTGTGGAAATGAATTCTTTCATTGCTCATATAAC 4522

ref TTTCTTCTGTGGAAATGAATTCTTTCATTGCTCATATAAC 4631

Consensus tttcttctgtggaaatgaattctttcattgctcatataac

kal TAGATAGTTTCGTTTTATCATACTTTATGCAAATATATAA 4557

yor TAGATAGTTTgGTTTTATCATACTTTATGCAAATATATAA 4559

jen TAGATAGTTTCGTTTTATCATACTTTATGCAAATATATAA 4557

cor TAGATAGTTTCGTTTTATCATACTTTATGCAAATATATAA 4557

man TAGATAGTTTCGTTTTATCATACTTTATGCAAATATATAA 4557

uni TAGATAGTTTCGTTTTATCATACTTTATGCAAATATATAA 4557

tall TAGATAGTTTCGTTTTATCATACTTTATGCAAATATATAA 4559

quil TAGATAGTTTCGTTTTATCATACTTTATGCAAATATATAA 4557

meri TAGATAGTTTCGTTTTATCATACTTgATGCAAATATATAA 4562

ref TAGATAGTTTCGTTTTATCATACTTgATGCAAATATATAA 4671

Consensus tagatagttt gttttatcatactt atgcaaatatataa

kal GCTTTATGTCTAGATATAGTTTACAACTATTGTATTCTGG 4597

yor GCTTTATGTCTAGATATAGTTTACAACTATTGTATTCTGG 4599

jen GCTTTATGTCTAGATATAGTTTACAACTATTGTATTCTGG 4597

cor GCTTTATGTCTAGATATAGTTTACAACTATTGTATTCTGG 4597

man GCTTTATGTCTAGATATAGTTTACAACTATTGTATTCTGG 4597

uni GCTTTATGTCTAGATATAGTTTACAACTATTGTATTCTGG 4597

tall GCTTTATGTCTAGATATAGTTTACAACTATTGTATTCTGG 4599

quil GCTTTATGTCTAGATATAGTTTACAACTATTGTATTCTGG 4597

meri GCTTTATGTCTAGATATAGTTTACAACTATTGTATTCTGG 4602

ref GCTTTATGTCTAGATATAGTTTACAACTATTGTATTCTGG 4711

Consensus gctttatgtctagatatagtttacaactattgtattctgg

kal TTTAAACTTATTTGATGATTTTCCATTATTTTATGATCAT 4637

yor TTTAAACTTATTTGATGATTTTCCATTATTTTATGATCAT 4639

jen TTTAAACTTATTTGATGATTTTCCATTATTTTATGATCAT 4637

cor TTTAAACTTATTTGATGATTTTCCATTATTTTATGATCAT 4637

man TTTAAACTTATTTGATGATTTTCCATTATTTTATGATCAT 4637

uni TTTAAACTTATTTGATGATTTTCCATTATTTTATGATCAT 4637

tall TTTAAACTTATTTGATGATTTTCCATTATTTTATGATCAT 4639

quil TTTAAACTTATTTGATGATTTTCCATTATTTTATGATCAT 4637

meri TTTAAACTTATTTGATGATTTTCCATTATTTTATGATCAT 4642

ref TTTAAACTTATTTGATGATTTTCCATTATTTTATGATCAT 4751

Consensus tttaaacttatttgatgattttccattattttatgatcat

kal GTGAATCTTAGGGAATTATAATTCATACAATGGTTATCAA 4677

yor GTGAATCTTAGGGAATTATAATTCATACAATGGTTATCAA 4679

jen GTGAATCTTAGGGAATTATAATTCATACAATGGTTATCAA 4677

cor GTGAATCTTAGGGAATTATAATTCATACAATGGTTATCAA 4677

man GTGAATCTTAGGGAATTATAATTCATACAATGGTTATCAA 4677

uni GTGAATCTTAGGGAATTATAATTCATACAATGGTTATCAA 4677

tall GTGAATCTTAGGGAATTATAATTCATACAATGGTTATCAA 4679

quil GTGAATCTTAGGGAATTATAATTCATACAATGGTTATCAA 4677

meri GTGAATCTTAGGGAATTATAATTCATACAATGGTTATCAA 4682

ref GTGAATCTTAGGGAATTATAATTCATACAATGGTTATCAA 4791

Consensus gtgaatcttagggaattataattcatacaatggttatcaa

kal ATAAGTTTTGGAAGTTTAAAATAATCACCACAGTAATAAT 4717

yor ATAAGTTTTGGAAGTTTAAAATAATCACCAtAGTAgTAAT 4719

jen ATAAGTTTTGGAAGTTTAAAATAATCACCACAGTAATAAT 4717

cor ATAAGTTTTGGAAGTTTAAAATAATCACCACAGTAATAAT 4717

man ATAAGTTTTGGAAGTTTAAAATAATCACCACAGTAATAAT 4717

uni ATAAGTTTTGGAAGTTTAAAATAATCACCACAGTAATAAT 4717

tall ATAAGTTTTGGAAGTTTAAAATAATCACCACAGTAATAAT 4719

quil ATAAGTTTTGGAAGTTTAAAATAATCACCACAGTAATAAT 4717

meri ATAAGTTTTGGAAGTTTAAAATAATCACCACAGTAATAAT 4722

ref ATAAGTTTTGGAAGTTTAAAATAATCACCACAGTAATAAT 4831

Consensus ataagttttggaagtttaaaataatcacca agta taat

kal CATTTTAAGGACTTATTCGATGATCATGTGAATCTTTGGA 4757

yor CATTTTAAGGACTTATTtGATGATCATGTGAATCTTTGGA 4759

jen CATTTTAAGGACTTATTCGATGATCATGTGAATCTTTGGA 4757

cor CATTTTAAGGACTTATTCGATGATCATGTGAATCTTTGGA 4757

man CATTTTAAGGACTTATTCGATGATCATGTGAATCTTTGGA 4757

uni CATTTTAAGGACTTATTCGATGATCATGTGAATCTTTGGA 4757

tall CATTTTAAGGACTTATTCGATGATCATGTGAATCTTTGGA 4759

quil CATTTTAAGGACTTATTCGATGATCATGTGAATCTTTGGA 4757

meri CATTTTAAGGACTTATTCGATGATCATGTGAATCTTTGGA 4762

ref CATTTTAAGGACTTATTCGATGATCATGTGAATCTTTGGA 4871

Consensus cattttaaggacttatt gatgatcatgtgaatctttgga

kal GGAGAACACAGTTCAAACCCTCATAATAAAGAGAAATCCT 4797

yor GGAGAACACAGTTCAAACCCTCATA...AAGAGAAATCCT 4796

jen GGAGAACACAGTTCAAACCCTCATAATAAAGAGAAATCCT 4797

cor GGAGAACACAGTTCAAACCCTCATAATAAAGAGAAATCCT 4797

man GGAGAACACAGTTCAAACCCTCATAATAAAGAGAAATCCT 4797

uni GGAGAACACAGTTCAAACCCTCATAATAAAGAGAAATCCT 4797

tall GGAGAACACAGTTCAAACCCTCATAATAAAGAGAAATCCT 4799

quil GGAGAACACAGTTCAAACCCTCATAATAAAGAGAAATCCT 4797

meri GGAGAACACAGTTCAAACCCTCATAATAAAGAGAAgTCCT 4802

ref GGAGAACACAGTTCAAACCCTCATAATAAAGAGAAgTCCT 4911

Consensus ggagaacacagttcaaaccctcata aagagaa tcct

kal TTATATGAATATTGTTTGCACCTTCTTTTCTCTCGTCGGT 4837

yor TTATATGAATATTGTTTGCACCTTCTTTTCTCTCGTCGGT 4836

jen TTATATGAATATTGTTTGCACCTTCTTTTCTCTCGTCGGT 4837

cor TTATATGAATATTGTTTGCACCTTCTTTTCTCTCGTCGGT 4837

man TTATATGAATATTGTTTGCACCTTCTTTTCTCTCGTCGGT 4837

uni TTATATGAATATTGTTTGCACCTTCTTTTCTCTCGTCGGT 4837

tall TTATATGAATATTGTTTGCACCTTCTTTTCTCTCGTCGGT 4839

quil TTATATGAATATTGTTTGCACCTTCTTTTCTCTCGTCGGT 4837

meri TTATATGAATATTGTTTGCACCTTCTTTTCTCTCGTCGGT 4842

ref TTATATGAATATTGTTTGCACCTTCTTTTCTCTCGTCGGT 4951

Consensus ttatatgaatattgtttgcaccttcttttctctcgtcggt

kal ATGAACAAGAATCCTCTCCGGGGAGATATGTGATTATTTT 4877

yor ATGAACAAGAATCCTCTCCGGGGAGATATGTGATTATTTT 4876

jen ATGAACAAGAATCCTCTCCGGGGAGATATGTGATTATTTT 4877

cor ATGAACAAGAATCCTCTCCGGGGAGATATGTGATTATTTT 4877

man ATGAACAAGAATCCTCTCCGGGGAGATATGTGATTATTTT 4877

uni ATGAACAAGAATCCTCTCCGGGGAGATATGTGATTATTTT 4877

tall ATGAACAAGAATCCTCTCCGGGGAGATATGTGATTATTTT 4879

quil ATGAACAAGAATCCTCTCCGGGGAGATATGTGATTATTTT 4877

meri ATGAACAAGAATCCTCTCCGGGGAGATATGTGATTATTTT 4882

ref ATGAACAAGAATCCTCTCCGGGGAGATATGTGATTATTTT 4991

Consensus atgaacaagaatcctctccggggagatatgtgattatttt

kal TACTCATATTTTTAATGACAAAAATGTTTTCATGGTAAAC 4917

yor TACTCATATTTTTAATGACAAAAATGTTTTCATGGTAAAC 4916

jen TACTCATATTTTTAATGACAAAAATGTTTTCATGGTAAAC 4917

cor TACTCATATTTTTAATGACAAAAATGTTTTCATGGTAAAC 4917

man TACTCATATTTTTAATGACAAAAATGTTTTCATGGTAAAC 4917

uni TACTCATATTTTTAATGACAAAAATGTTTTCATGGTAAAC 4917

tall TACTCATATTTTTAATGACAAAAATGTTTTCATGGTAAAC 4919

quil TACTCATATTTTTAATGACAAAAATGTTTTCATGGTAAAC 4917

meri TACTCATATTTTTAATGACAAAAATGTTTTCATaGTAAAC 4922

ref TACTCATATTTTTAATGACAAAAATGTTTTCATaGTAAAC 5031

Consensus tactcatatttttaatgacaaaaatgttttcat gtaaac

kal AAAAGGATAAAAGTGTCATTACAGGTTGCATTTTATGTGT 4957

yor AAAAGGATAAAAGTGTCATTACAGGTTGCATTTTATGTGT 4956

jen AAAAGGATAAAAGTGTCATTACAGGTTGCATTTTATGTGT 4957

cor AAAAGGATAAAAGTGTCATTACAGGTTGCATTTTATGTGT 4957

man AAAAGGATAAAAGTGTCATTACAGGTTGCATTTTATGTGT 4957

uni AAAAGGATAAAAGTGTCATTACAGGTTGCATTTTATGTGT 4957

tall AAAAGGATAAAAGTGTCATTACAGGTTGCATTTTATGTGT 4959

quil AAAAGGATAAAAGTGTCATTACAGGTTGCATTTTATGTGT 4957

meri AAAAGGATAAAAGTGTCATTACAGGTTGCATTTTATGTGT 4962

ref AAAgGGATAAAAGTGTCATTACAaGTTGCATTTTATGTGT 5071

Consensus aaa ggataaaagtgtcattaca gttgcattttatgtgt

kal TATTGTGTGTCACTCGCCTCTCCATTAATCATTACTTCCT 4997

yor TATTGTGTGTCACTCGCCTCTCCATTAATCATTACTTCCT 4996

jen TATTGTGTGTCACTCGCCTCTCCATTAATCATTACTTCCT 4997

cor TATTGTGTGTCACTCGCCTCTCCATTAATCATTACTTCCT 4997

man TATTGTGTGTCACTCGCCTCTCCATTAATCATTACTTCCT 4997

uni TATTGTGTGTCACTCGCCTCTCCATTAATCATTACTTCCT 4997

tall TATTGTGTGTCACTCGCCTCTCCATTAATCATTACTTCCT 4999

quil TATTGTGTGTCACTCGCCTCTCCATTAATCATTACTTCCT 4997

meri TATTGTGTGTCACTCGCCTCTCCATTAATCcTTACTTCCT 5002

ref TATTGTGTGTCACTCGCCTCTCCATTAATCcTTACTTCCT 5111

Consensus tattgtgtgtcactcgcctctccattaatc ttacttcct

kal TCATATGTATAGCATTTGTTTCATACTAATCAAAATATCG 5037

yor TCATATGTATAGCATTTGTTTCATACTAATCAAAATATCG 5036

jen TCATATGTATAGCATTTGTTTCATACTAATCAAAATATCG 5037

cor TCATATGTATAGCATTTGTTTCATACTAATCAAAATATCG 5037

man TCATATGTATAGCATTTGTTTCATACTAATCAAAATATCG 5037

uni TCATATGTATAGCATTTGTTTCATACTAATCAAAATATCG 5037

tall TCATATGTATAGCATTTGTTTCATACTAATCAAAATATCG 5039

quil TCATATGTATAGCATTTGTTTCATACTAATCAAAATATCG 5037

meri TCATATGTATAGCATTTGTTTCATACTAATCAAAATATCa 5042

ref TCATATGTATAGCATTTGTTTCATACTAATCAAAATATCa 5151

Consensus tcatatgtatagcatttgtttcatactaatcaaaatatc

kal TACCAGTTAAAAAATTATTTTTTCTATTTTGACTTCATTA 5077

yor TACCAGTTAAAAAATTATTTTTTCTATTTTGACTTCATTA 5076

jen TACCAGTTAAAAAATTATTTTTTCTATTTTGACTTCATTA 5077

cor TACCAGTTAAAAAATTATTTTTTCTATTTTGACTTCATTA 5077

man TACCAGTTAAAAAATTATTTTTTCTATTTTGACTTCATTA 5077

uni TACCAGTTAAAAAATTATTTTTTCTATTTTGACTTCATTA 5077

tall TACCAGTTAAAAAATTATTTTTTCTATTTTGACTTCATTA 5079

quil TACCAGTTAAAAAATTATTTTTTCTATTTTGACTTCATTA 5077

meri TACCAGTTgAAAAATTATTTTTcta.TTTTGACTTCATTA 5081

ref TACCAGTTgAAAAATTATTTTTTC..TTTTGACTTCATTA 5189

Consensus taccagtt aaaaattattttt ttttgacttcatta

kal TAATTGGTTGAGGACAAACTTTGAGATATAATTGGGGATA 5117

yor TAATTGGTTGAGGACAAACTTTGAGATATAATTGGGGATA 5116

jen TAATTGGTTGAGGACAAACTTTGAGATATAATTGGGGATA 5117

cor TAATTGGTTGAGGACAAACTTTGAGATATAATTGGGGATA 5117

man TAATTGGTTGAGGACAAACTTTGAGATATAATTGGGGATA 5117

uni TAATTGGTTGAGGACAAACTTTGAGATATAATTGGGGATA 5117

tall TAATTGGTTGAGGACAAACTTTGAGATATAATTGGGGATA 5119

quil TAATTGGTTGAGGACAAACTTTGAGATATAATTGGGGATA 5117

meri TAATTGGTTGAGGACAAACTTTGAGATATAATTGGGGATA 5121

ref TAATTGGTTGAaGACAAACTTTGAGATATAATTGGGGATA 5229

Consensus taattggttga gacaaactttgagatataattggggata

kal GCATATCATTGTTTCATTCATATAAAGAATGATTACATGT 5157

yor GCATATCATTGTTTCATTCATATAAAGAATGATTACATGT 5156

jen GCATATCATTGTTTCATTCATATAAAGAATGATTACATGT 5157

cor GCATATCATTGTTTCATTCATATAAAGAATGATTACATGT 5157

man GCATATCATTGTTTCATTCATATAAAGAATGATTACATGT 5157

uni GCATATCATTGTTTCATTCATATAAAGAATGATTACATGT 5157

tall GCATATCATTGTTTCATTCATATAAAGAATGATTACATGT 5159

quil GCATATCATTGTTTCATTCATATAAAGAATGATTACATGT 5157

meri aCgTATCATTGTcTCATTCATATAAAaAATGATcACATGT 5161

ref aCgTATCATTGTcTCATTCATATAAAaAATGATcACATGT 5269

Consensus c tatcattgt tcattcatataaa aatgat acatgt

kal CCCAAAAAATTTAT.......................... 5171

yor CCCAAAAAATTTAT.......................... 5170

jen CCCAAAAAATTTAT.......................... 5171

cor CCCAAAAAATTTAT.......................... 5171

man CCCAAAAAATTTAT.......................... 5171

uni CCCAAAAAATTTAT.......................... 5171

tall CCCAAAAAATTTAT.......................... 5173

quil CCCAAAAAATTTAT.......................... 5171

meri CCCAAAAAATTTATatctcatgtcccatatcccaaaaaat 5201

ref CCCAAAAAATTcATatctcatgtcctatgttccaaaaaat 5309

Consensus cccaaaaaatt at

kal ....GTCTCATATCCCATATCCCAAACTATTCACATTCCA 5207

yor ....GTCTCATATCCCATATCCCAAACTATTCACATTCCA 5206

jen ....GTCTCATATCCCATATCCCAAACTATTCACATTCCA 5207

cor ....GTCTCATATCCCATATCCCAAACTATTCACATTCCA 5207

man ....GTCTCATATCCCATATCCCAAACTATTCACATTCCA 5207

uni ....GTCTCATATCCCATATCCCAAACTATTCACATTCCA 5207

tall ....GTCTCATATCCCATATCCCAAACTATTCACATTCCA 5209

quil ....GTCTCATATCCCATATCCCAAACTATTCACATTCCA 5207

meri tcatGTCTCATATCCCATATCtCAAACTATTCACATTCCA 5241

ref tcatGTCTCATATCCCATATCtCAAACTATTCACATTCCA 5349

Consensus gtctcatatcccatatc caaactattcacattcca

kal CCAA.TAACATA............TTCTATTTTTATGTTT 5234

yor CCAA.TAACATA............TTCTATTTTTATGTTT 5233

jen CCAA.TAACATA............TTCTATTTTTATGTTT 5234

cor CCAA.TAACATA............TTCTATTTTTATGTTT 5234

man CCAA.TAACATA............TTCTATTTTTATGTTT 5234

uni CCAA.TAACATA............TTCTATTTTTATGTTT 5234

tall CCAA.TAACATA............TTCTATTTTTATGTTT 5236

quil CCAA.TAACATA............TTCTATTTTTATGTTT 5234

meri CCAAgTAACATAcaaaaatttataTTCTATTTTTATGTTT 5281

ref CCAAgTAACATAcaaaaatttataTTCTATTTTTATGTTT 5389

Consensus ccaa taacata ttctatttttatgttt

kal TTTATTAATTAATTAGCATTTTTTGATTGAATAGTGAATG 5274

yor TTTATTAATTAATTAGCATTTTTTGATTGAATAGTGAATG 5273

jen TTTATTAATTAATTAGCATTTTTTGATTGAATAGTGAATG 5274

cor TTTATTAATTAATTAGCATTTTTTGATTGAATAGTGAATG 5274

man TTTATTAATTAATTAGCATTTTTTGATTGAATAGTGAATG 5274

uni TTTATTAATTAATTAGCATTTTTTGATTGAATAGTGAATG 5274

tall TTTATTAATTAATTAGCATTTTTTGATTGAATAGTGAATG 5276

quil TTTATTAATTAATTAGCATTTTTTGATTGAATAGTGAATG 5274

meri TTTATTAATTAgTTAGCATTTTTTGATTGAATAaTGAATG 5321

ref TTTATTAATTAgTTAGCATTTTTTGATTGAATAaTGAATG 5429

Consensus tttattaatta ttagcattttttgattgaata tgaatg

kal AAACACAAAATATTGGGATAGATGATCTCTATCATTCAAC 5314

yor AAACAtAAAATATTGGGATAGATGATCTCTATCATTCAAC 5313

jen AAACACAAAATATTGGGATAGATGATCTCTATCATTCAAC 5314

cor AAACACAAAATATTGGGATAGATGATCTCTATCATTCAAC 5314

man AAACACAAAATATTGGGATAGATGATCTCTATCATTCAAC 5314

uni AAACACAAAATATTGGGATAGATGATCTCTATCATTCAAC 5314

tall AAACACAAAATATTGGGATAGATGATCTCTATCATTCAAC 5316

quil AAACACAAAATATTGGGATAGATGATCTCTATCATTCAAC 5314

meri AgACACAAAATATTGGG....ATGATCTCagTCATTCAAC 5357

ref AgACACAAAATATTGGa....ATGATCTCTgTCATTCAAC 5465

Consensus a aca aaaatattgg atgatctc tcattcaac

kal CATCTCACGTCTAAAAATTGTAAAGTCTAATCTCATCAAT 5354

yor CATCTCACGTCTAAAAATTGTAAAGTCTAATCTCATCAAT 5353

jen CATCTCACGTCTAAAAATTGTAAAGTCTAATCTCATCAAT 5354

cor CATCTCACGTCTAAAAATTGTAAAGTCTAATCTCATCAAT 5354

man CATCTCACGTCTAAAAATTGTAAAGTCTAATCTCATCAAT 5354

uni CATCTCACGTCTAAAAATTGTAAAGTCTAATCTCATCAAT 5354

tall CATCTCACGTCTAAAAATTGTAAAGTCTAATCTCATCAAT 5356

quil CATCTCACGTCTAAAAATTGTAAAGTCTAATCTCATCAAT 5354

meri CATCTCACGTCTAAAAATTGTAAAGTCTAATCTCATaAAT 5397

ref CATCTCACGTCTAAAAATTGTAAAGTCTAATCTCATaAAT 5505

Consensus catctcacgtctaaaaattgtaaagtctaatctcat aat

kal ATTTATATAGACATGTTTTTTTCATTCACACAATTCTAAG 5394

yor ATTTATATAGACATGTTTTTTTCATTCACACAATTCTAAG 5393

jen ATTTATATAGACATGTTTTTTTCATTCACACAATTCTAAG 5394

cor ATTTATATAGACATGTTTTTTTCATTCACACAATTCTAAG 5394

man ATTTATATAGACATGTTTTTTTCATTCACACAATTCTAAG 5394

uni ATTTATATAGACATGTTTTTTTCATTCACACAATTCTAAG 5394

tall ATTTATATAGACATGTTTTTTTCATTCACACAATTCTAAG 5396

quil ATTTATATAGACATGTTTTTTTCATTCACACAATTCTAAG 5394

meri ATTTATATAGACATGTTTTTTTCATTCACACAAaTCTcAG 5437

ref ATTTATATAGACATGTTTTTTTCATTCACACAAaTCTcAG 5545

Consensus atttatatagacatgtttttttcattcacacaa tct ag

kal TTTGTTTTCAGAGAAAACTACATTTTCAGAATCCTGACAA 5434

yor TTTGTTTTCAGAGAAAACTACATTTTCAGAATCCTGACAA 5433

jen TTTGTTTTCAGAGAAAACTACATTTTCAGAATCCTGACAA 5434

cor TTTGTTTTCAGAGAAAACTACATTTTCAGAATCCTGACAA 5434

man TTTGTTTTCAGAGAAAACTACATTTTCAGAATCCTGACAA 5434

uni TTTGTTTTCAGAGAAAACTACATTTTCAGAATCCTGACAA 5434

tall TTTGTTTTCAGAGAAAACTACATTTTCAGAATCCTGACAA 5436

quil TTTGTTTTCAGAGAAAACTACATTTTCAGAATCCTGACAA 5434

meri TTTGTTTTtAGtGAAAACTACATTTTCAGAATCCTGACAA 5477

ref TTTGTTTTtAGtGAAAACTACATTTTCAGAATCCTGACAA 5585

Consensus tttgtttt ag gaaaactacattttcagaatcctgacaa

kal TGCAATAATTTTCCAATGGATAGGAGTGAAGTATATAAGC 5474

yor TGCAATAATTTTCCAATGGATAGGAGTGAAGTATATAAGC 5473

jen TGCAATAATTTTCCAATGGATAGGAGTGAAGTATATAAGC 5474

cor TGCAATAATTTTCCAATGGATAGGAGTGAAGTATATAAGC 5474

man TGCAATAATTTTCCAATGGATAGGAGTGAAGTATATAAGC 5474

uni TGCAATAATTTTCCAATGGATAGGAGTGAAGTATATAAGC 5474

tall TGCAATAATTTTCCAATGGATAGGAGTGAAGTATATAAGC 5476

quil TGCAATAATTTTCCAATGGATAGGAGTGAAGTATATAAGC 5474

meri TGCAATAATTTTCCAATGGATAGGAGTGAAGTATATAAGC 5517

ref TGCAATAATTTTCCAATGGATAGGAGTGAAGTATATAAGC 5625

Consensus tgcaataattttccaatggataggagtgaagtatataagc

kal TACTTTTTTCACTATAAAATAG........GAGTTCAACT 5506

yor TACTTTTTTCACTATAAAATAG........GAGTTCAACT 5505

jen TACTTTTTTCACTATAAAATAG........GAGTTCAACT 5506

cor TACTTTTTTCACTATAAAATAG........GAGTTCAACT 5506

man TACTTTTTTCACTATAAAATAG........GAGTTCAACT 5506

uni TACTTTTTTCACTATAAAATAG........GAGTTCAACT 5506

tall TACTTTTTTCACTATAAAATAG........GAGTTCAACT 5508

quil TACTTTTTTCACTATAAAATAG........GAGTTCAACT 5506

meri TACTTTTTTCACTATAAAAaAaaaaaaaa.GAGTTCAACT 5556

ref TACTTTTTTCACTATAAAAaAaaaaaaaaaGAGTTCAACT 5665

Consensus tacttttttcactataaaa a gagttcaact

kal CCTTTCCACT.............................. 5516

yor CCTTTCCACT.............................. 5515

jen CCTTTCCACT.............................. 5516

cor CCTTTCCACT.............................. 5516

man CCTTTCCACT.............................. 5516

uni CCTTTCCACT.............................. 5516

tall CCTTTCCACT.............................. 5518

quil CCTTTCCACT.............................. 5516

meri CCTcTtCACTgcccggagaaaccagtactggaatatgagg 5596

ref CCTcTtCACTacc.ggagaaaccagtactggaatatgagg 5704

Consensus cct t cact

kal ........................................ 5516

yor ........................................ 5515

jen ........................................ 5516

cor ........................................ 5516

man ........................................ 5516

uni ........................................ 5516

tall ........................................ 5518

quil ........................................ 5516

meri atgagatttttctgaagaatttttatttattttttaatag 5636

ref atgagatttttctgaagaatttttatttattttttaatag 5744

Consensus

kal .....AATATTTAATTATAACCATTTAAAGAAATTTTTTG 5551

yor .....AATATTTAATTATAACCATTTAAAGAAATTTTTTG 5550

jen .....AATATTTAATTATAACCATTTAAAGAAATTTTTTG 5551

cor .....AATATTTAATTATAACCATTTAAAGAAATTTTTTG 5551

man .....AATATTTAATTATAACCATTTAAAGAAATTTTTTG 5551

uni .....AATATTTAATTATAACCATTTAAAGAAATTTTTTG 5551

tall .....AATATTTAATTATAACCATTTAAAGAAATTTTTTG 5553

quil .....AATATTTAATTATAACCATTTAAAGAAATTTTTTG 5551

meri aaaatAAcATTTAATcATAACtgTTaAAAGAAAaaaTTTG 5676

ref aaaatAAcATTTAATcATAACtgTTaAAAGAAAaaaTTTG 5784

Consensus aa atttaat ataac tt aaagaaa tttg

kal TCATATATCTTAATCCTATCCAATACTGGAATTTCCGGTA 5591

yor TCATATATCTTAATCCTATCCAATACTGGAATcTCCGGTA 5590

jen TCATATATCTTAATCCTATCCAATACTGGAATTTCCGGTA 5591

cor TCATATATCTTAATCCTATCCAATACTGGAATTTCCGGTA 5591

man TCATATATCTTAATCCTATCCAATACTGGAATTTCCGGTA 5591

uni TCATATATCTTAATCCTATCCAATACTGGAATTTCCGGTA 5591

tall TCATATATCTTAATCCTATCCAATACTGGAATTTCCGGTA 5593

quil TCATATATCTTAATCCTATCCAATACTGGAATTTCCGGTA 5591

meri TCAcATtTCTTAATtCTATCCAgTACTGGAcTcTCCGGTA 5716

ref TCAcATtTCTTAATtCTATCCAgTACTGGAcTcTCCGGTA 5824

Consensus tca at tcttaat ctatcca tactgga t tccggta

kal CTCGAGATG.TCCCCTCATAAAAAAGGTGGTGCTATATTT 5630

yor CTCGAGATG.TCCCCTCATAAAAAAGGTGGTGCTATATTT 5629

jen CTCGAGATG.TCCCCTCATAAAAAAGGTGGTGCTATATTT 5630

cor CTCGAGATG.TCCCCTCATAAAAAAGGTGGTGCTATATTT 5630

man CTCGAGATG.TCCCCTCATAAAAAAGGTGGTGCTATATTT 5630

uni CTCGAGATG.TCCCCTCATAAAAAAGGTGGTGCTATATTT 5630

tall CTCGAGATG.TCCCCTCATAAAAAAGGTGGTGCTATATTT 5632

quil CTCGAGATG.TCCCCTCATAAAAAAGGTGGTGCTATATTT 5630

meri tTCGAGATGgTaCCCTCATAAAAAAGGTaGTGCTATATTT 5756

ref tTCGAGATGgTaCCCTCATAAAAAAGGTaGTGCTATATTT 5864

Consensus tcgagatg t ccctcataaaaaaggt gtgctatattt

kal GGTAAAGCTTATTTTTAGTTTATCTACCGCAAAATGGCAA 5670

yor GGTAAAGCTTATTTTTAGTTTATCTACCGCAAAATGGCAA 5669

jen GGTAAAGCTTATTTTTAGTTTATCTACCGCAAAATGGCAA 5670

cor GGTAAAGCTTATTTTTAGTTTATCTACCGCAAAATGGCAA 5670

man GGTAAAGCTTATTTTTAGTTTATCTACCGCAAAATGGCAA 5670

uni GGTAAAGCTTATTTTTAGTTTATCTACCGCAAAATGGCAA 5670

tall GGTAAAGCTTATTTTTAGTTTATCTACCGCAAAATGGCAA 5672

quil GGTAAAGCTTATTTTTAGTTTATCTACCGCAAAATGGCAA 5670

meri GGTAAAGCTTATTTTTAGTTTATCTACCGCAAAATGGCAA 5796

ref GGTAAAGCTTATTTTTAGTTTATCTACCGCAAAATGGCAA 5904

Consensus ggtaaagcttatttttagtttatctaccgcaaaatggcaa

kal TAACCAATAATGATGGATGAATGGTTTGGTTGTGTTTTGA 5710

yor TAACCAATAATGATGGATGAATGGTTTGGTTGTGTTTTGA 5709

jen TAACCAATAATGATGGATGAATGGTTTGGTTGTGTTTTGA 5710

cor TAACCAATAATGATGGATGAATGGTTTGGTTGTGTTTTGA 5710

man TAACCAATAATGATGGATGAATGGTTTGGTTGTGTTTTGA 5710

uni TAACCAATAATGATGGATGAATGGTTTGGTTGTGTTTTGA 5710

tall TAACCAATAATGATGGATGAATGGTTTGGTTGTGTTTTGA 5712

quil TAACCAATAATGATGGATGAATGGTTTGGTTGTGTTTTGA 5710

meri TAACtAATAATGATGGATGAATGGTTTGGTgGTGTTTTGA 5836

ref TAACtAATAATGATGGATGAATGGTTTGGTgGTGTTTTGA 5944

Consensus taac aataatgatggatgaatggtttggt gtgttttga

kal TGAGGCTTTAATGTGTACTGGTCACTATAATAAAGTTGAG 5750

yor TGAGGCTTTAATGTGTACTGGTCACTATAATAAAGTTGAG 5749

jen TGAGGCTTTAATGTGTACTGGTCACTATAATAAAGTTGAG 5750

cor TGAGGCTTTAATGTGTACTGGTCACTATAATAAAGTTGAG 5750

man TGAGGCTTTAATGTGTACTGGTCACTATAATAAAGTTGAG 5750

uni TGAGGCTTTAATGTGTACTGGTCACTATAATAAAGTTGAG 5750

tall TGAGGCTTTAATGTGTACTGGTCACTATAATAAAGTTGAG 5752

quil TGAGGCTTTAATGTGTACTGGTCACTATAATAAAGTTGAG 5750

meri TGAGGCTTTAATGTGTAC.GGTCACTATAATAAAGTTGAG 5875

ref TGAGGCTTTAATGTGTAC.GGTCACTATAATAAAGTTGAG 5983

Consensus tgaggctttaatgtgtac ggtcactataataaagttgag

kal TTGAATATTTGTGAAAACAGATTCACCAAAGCAACCATAA 5790

yor TTGAATATTTGTGAAAACAGATTCACCAAAGCAACCATAA 5789

jen TTGAATATTTGTGAAAACAGATTCACCAAAGCAACCATAA 5790

cor TTGAATATTTGTGAAAACAGATTCACCAAAGCAACCATAA 5790

man TTGAATATTTGTGAAAACAGATTCACCAAAGCAACCATAA 5790

uni TTGAATATTTGTGAAAACAGATTCACCAAAGCAACCATAA 5790

tall TTGAATATTTGTGAAAACAGATTCACCAAAGCAACCATAA 5792

quil TTGAATATTTGTGAAAACAGATTCACCAAAGCAACCATAA 5790

meri TTGAATATTTGTGAAAACAGATTCACCAAAGCAACCATAA 5915

ref TTGAATATTTGTGAAAACAGATTCACCAAAGCAACCATAA 6023

Consensus ttgaatatttgtgaaaacagattcaccaaagcaaccataa

kal CTCATATGTGACCTACATTGTGAGAAATTATTCCATGCGC 5830

yor CTCATATGTGACCTACATTGTGAGAAATTATTCCATGgGC 5829

jen CTCATATGTGACCTACATTGTGAGAAATTATTCCATGCGC 5830

cor CTCATATGTGACCTACATTGTGAGAAATTATTCCATGCGC 5830

man CTCATATGTGACCTACATTGTGAGAAATTATTCCATGCGC 5830

uni CTCATATGTGACCTACATTGTGAGAAATTATTCCATGCGC 5830

tall CTCATATGTGACCTACATTGTGAGAAATTATTCCATGCGC 5832

quil CTCATATGTGACCTACATTGTGAGAAATTATTCCATGCGC 5830

meri CTCATATGTGACCTACATTGTGAGAAATTATTCCATGgGt 5955

ref CTCATATGTGACCTACATTGTGAGAAATTATTCCATGgGt 6063

Consensus ctcatatgtgacctacattgtgagaaattattccatg g

kal ACGTACCAATGGTTGAGAACATTTCAACTCAACTCTCAAT 5870

yor ACGTACCAATGGTTGAGAACATTTCAACTCAACTCTCAAT 5869

jen ACGTACCAATGGTTGAGAACATTTCAACTCAACTCTCAAT 5870

cor ACGTACCAATGGTTGAGAACATTTCAACTCAACTCTCAAT 5870

man ACGTACCAATGGTTGAGAACATTTCAACTCAACTCTCAAT 5870

uni ACGTACCAATGGTTGAGAACATTTCAACTCAACTCTCAAT 5870

tall ACGTACCAATGGTTGAGAACATTTCAACTCAACTCTCAAT 5872

quil ACGTACCAATGGTTGAGAACATTTCAACTCAACTCTCAAT 5870

meri ACGTACCAATGGTTGAGAACATTTCAACTCAACTCTCAAT 5995

ref ACGTACCAATGGTTGAGAACATTTCAACTCAACTCTCAAT 6103

Consensus acgtaccaatggttgagaacatttcaactcaactctcaat

kal AAATACATGCGTTTTAGACTATTGTTGATAAATATCTAAA 5910

yor AAATACATGCGTTTTAGACTATTGTTGATAAATATCTAAA 5909

jen AAATACATGCGTTTTAGACTATTGTTGATAAATATCTAAA 5910

cor AAATACATGCGTTTTAGACTATTGTTGATAAATATCTAAA 5910

man AAATACATGCGTTTTAGACTATTGTTGATAAATATCTAAA 5910

uni AAATACATGCGTTTTAGACTATTGTTGATAAATATCTAAA 5910

tall AAATACATGCGTTTTAGACTATTGTTGATAAATATCTAAA 5912

quil AAATACATGCGTTTTAGACTATTGTTGATAAATATCTAAA 5910

meri AAATACATGCGTTTTAGACTATTGTTGATAAATATCTAAA 6035

ref AAATACATGCGTTTTAGACTATTGTTGATAAATATCTAAA 6143

Consensus aaatacatgcgttttagactattgttgataaatatctaaa

kal TCATAACAACTGTCTCTTCACTTGTATGAATAAAGTTACC 5950

yor TCATAACAACTGTCTCTTCACTTGTATGAATAAAGTTACC 5949

jen TCATAACAACTGTCTCTTCACTTGTATGAATAAAGTTACC 5950

cor TCATAACAACTGTCTCTTCACTTGTATGAATAAAGTTACC 5950

man TCATAACAACTGTCTCTTCACTTGTATGAATAAAGTTACC 5950

uni TCATAACAACTGTCTCTTCACTTGTATGAATAAAGTTACC 5950

tall TCATAACAACTGTCTCTTCACTTGTATGAATAAAGTTACC 5952

quil TCATAACAACTGTCTCTTCACTTGTATGAATAAAGTTACC 5950

meri TCATAACAACTGTCTCTTCgCTTaTActAATAAAGTTACC 6075

ref TCATAACAACTGTCTCTTCgCTTaTActAATAAAGTTACC 6183

Consensus tcataacaactgtctcttc ctt ta aataaagttacc

kal TATATTTATCATTTCGTTCTCATACCCTAGTGTTGGAATC 5990

yor TATATTTATCATTTCGTTCTCATACCCTAGTGTTGGAATC 5989

jen TATATTTATCATTTCGTTCTCATACCCTAGTGTTGGAATC 5990

cor TATATTTATCATTTCGTTCTCATACCCTAGTGTTGGAATC 5990

man TATATTTATCATTTCGTTCTCATACCCTAGTGTTGGAATC 5990

uni TATATTTATCATTTCGTTCTCATACCCTAGTGTTGGAATC 5990

tall TATATTTATCATTTCGTTCTCATACCCTAGTGTTGGAATC 5992

quil TATATTTATCATTTCGTTCTCATACCCTAGTGTTGGAATC 5990

meri TATATTTATCATTTCaTTCctATACCtTAGaGTTGGAATC 6115

ref TATATTTATCATTTCaTTCctATACCtTAGaGTTGGAATC 6223

Consensus tatatttatcatttc ttc atacc tag gttggaatc

kal TCGTGCACTAGGTTGCAAAAAAAAAA....CTATTATTGA 6026

yor TCGTGCACTAGGTTGCAAAAAAAAA.....CTATTATTGA 6024

jen TCGTGCACTAGGTTGCAAAAAAAAAA....CTATTATTGA 6026

cor TCGTGCACTAGGTTGCAAAAAAAAAA....CTATTATTGA 6026

man TCGTGCACTAGGTTGCAAAAAAAAAA....CTATTATTGA 6026

uni TCGTGCACTAGGTTGCAAAAAAAAAA....CTATTATTGA 6026

tall TCGTGCACTAGGTTGCAAAAAAAAAA....CTATTATTGA 6028

quil TCGTGCACTAGGTTGCAAAAAAAAAA....CTATTATTGA 6026

meri TCGTGCACTAGGTTGCAAAAAAAAAAg...CTATTATTGA 6152

ref TCGTGCACTAGGTTGCAAAAAAAAAAaaaaCTATTATTGA 6263

Consensus tcgtgcactaggttgcaaaaaaaaa ctattattga

kal TGTATATAATATCATATAAGAAATTCTCTTGCTAAGATAG 6066

yor TGTATATAATATCATATAAGAAATTCTCTTGCTAAGATAG 6064

jen TGTATATAATATCATATAAGAAATTCTCTTGCTAAGATAG 6066

cor TGTATATAATATCATATAAGAAATTCTCTTGCTAAGATAG 6066

man TGTATATAATATCATATAAGAAATTCTCTTGCTAAGATAG 6066

uni TGTATATAATATCATATAAGAAATTCTCTTGCTAAGATAG 6066

tall TGTATATAATATCATATAAGAAATTCTCTTGCTAAGATAG 6068

quil TGTATATAATATCATATAAGAAATTCTCTTGCTAAGATAG 6066

meri TGTATATAATATCATATAAGAAATTCTCTTGCTAAGATAG 6192

ref TGTATAcAATATCATATAAGAAATTCTCTTGCTAAGATAG 6303

Consensus tgtata aatatcatataagaaattctcttgctaagatag

kal TAAGATGTAAGTTTGCGTTTTTAAAATTAAATCCTTTAAA 6106

yor TAAGATGTAAGTTTGCGTTTTTAAAATTAAATCCTTTAAA 6104

jen TAAGATGTAAGTTTGCGTTTTTAAAATTAAATCCTTTAAA 6106

cor TAAGATGTAAGTTTGCGTTTTTAAAATTAAATCCTTTAAA 6106

man TAAGATGTAAGTTTGCGTTTTTAAAATTAAATCCTTTAAA 6106

uni TAAGATGTAAGTTTGCGTTTTTAAAATTAAATCCTTTAAA 6106

tall TAAGATGTAAGTTTGCGTTTTTAAAATTAAATCCTTTAAA 6108

quil TAAGATGTAAGTTTGCGTTTTTAAAATTAAATCCTTTAAA 6106

meri TAAGATGTAAGTTTGCGTTTTTAAAATTAAATCCTTTAAA 6232

ref TAAGATGTAAGTTTGCGTTTTTgAAATTAAATCCTTTAAA 6343

Consensus taagatgtaagtttgcgttttt aaattaaatcctttaaa

kal CAGAAGTTTGCAATTTCACTATACCCAAAACTCATAAGAA 6146

yor CAGAAGTTTGCAATTTCACTATACCCAAAACTCATAAGAA 6144

jen CAGAAGTTTGCAATTTCACTATACCCAAAACTCATAAGAA 6146

cor CAGAAGTTTGCAATTTCACTATACCCAAAACTCATAAGAA 6146

man CAGAAGTTTGCAATTTCACTATACCCAAAACTCATAAGAA 6146

uni CAGAAGTTTGCAATTTCACTATACCCAAAACTCATAAGAA 6146

tall CAGAAGTTTGCAATTTCACTATACCCAAAACTCATAAGAA 6148

quil CAGAAGTTTGCAATTTCACTATACCCAAAACTCATAAGAA 6146

meri CAGAAGTTTGCAATTTCACTATACCCAAAACTCATAAGAA 6272

ref CA.AAcTTTGCAATTTCACTcTACCCAAAACTCATAAGAA 6382

Consensus ca aa tttgcaatttcact tacccaaaactcataagaa

kal TAATGTCCTTCAAGGATAT.AACGAGGTGTAGAGATGACA 6185

yor TAATGTCCTTCAAGGATAT.AACGAGGTGTAGAGATGACA 6183

jen TAATGTCCTTCAAGGATAT.AACGAGGTGTAGAGATGACA 6185

cor TAATGTCCTTCAAGGATAT.AACGAGGTGTAGAGATGACA 6185

man TAATGTCCTTCAAGGATAT.AACGAGGTGTAGAGATGACA 6185

uni TAATGTCCTTCAAGGATAT.AACGAGGTGTAGAGATGACA 6185

tall TAATGTCCTTCAAGGATAT.AACGAGGTGTAGAGATGACA 6187

quil TAATGTCCTTCAAGGATAT.AACGAGGTGTAGAGATGACA 6185

meri TAATGTCCTTCAAGGATAT.AACGAGGTGTAGAGATGACA 6311

ref TAATGTCCTTCAAGGATATtAACGAGGTaTAaAaATGACA 6422

Consensus taatgtccttcaaggatat aacgaggt ta a atgaca

kal ATATGGACTGACATTTTTAGCAGGTCGGACTGAGGATTTA 6225

yor ATATGGACTGACATTTTTAGCAGGTCGGACTGAGGATTTA 6223

jen ATATGGACTGACATTTTTAGCAGGTCGGACTGAGGATTTA 6225

cor ATATGGACTGACATTTTTAGCAGGTCGGACTGAGGATTTA 6225

man ATATGGACTGACATTTTTAGCAGGTCGGACTGAGGATTTA 6225

uni ATATGGACTGACATTTTTAGCAGGTCGGACTGAGGATTTA 6225

tall ATATGGACTGACATTTTTAGCAGGTCGGACTGAGGATTTA 6227

quil ATATGGACTGACATTTTTAGCAGGTCGGACTGAGGATTTA 6225

meri ATATGaACTGACATTTTTAGCAGGTCGGACTGAGGATTTA 6351

ref ATATGGACTGACATTTTTAGCAGGTCGGAtTGgaGATTTg 6462

Consensus atatg actgacatttttagcaggtcgga tg gattt

kal AGCCCTTTTAGTA.TAAGGGTTGGGTCGGATCAGCTCTTA 6264

yor AGCCCTTTTAGTA.TAAGGGTTGGGTCGGATCAGCTCTTA 6262

jen AGCCCTTTTAGTA.TAAGGGTTGGGTCGGATCAGCTCTTA 6264

cor AGCCCTTTTAGTA.TAAGGGTTGGGTCGGATCAGCTCTTA 6264

man AGCCCTTTTAGTA.TAAGGGTTGGGTCGGATCAGCTCTTA 6264

uni AGCCCTTTTAGTA.TAAGGGTTGGGTCGGATCAGCTCTTA 6264

tall AGCCCTTTTAGTA.TAAGGGTTGGGTCGGATCAGCTCTTA 6266

quil AGCCCTTTTAGTA.TAAGGGTTGGGTCGGATCAGCTCTTA 6264

meri AGCCCTTTTAGTA.TAAGGGTTGGGTCGGATCAGCTCTTA 6390

ref AGCCCTTTTAGcAaTAAaGGTTaGGTCGGATtAGCTCTTA 6502

Consensus agcccttttag a taa ggtt ggtcggat agctctta

kal TTTTGGTGAGCGAAAGGTGGACCGGGCTGACCCGTATTAT 6304

yor TTTTGGTGAGCGAAAGGTGGACCGGGCTGACCCGTATTAT 6302

jen TTTTGGTGAGCGAAAGGTGGACCGGGCTGACCCGTATTAT 6304

cor TTTTGGTGAGCGAAAGGTGGACCGGGCTGACCCGTATTAT 6304

man TTTTGGTGAGCGAAAGGTGGACCGGGCTGACCCGTATTAT 6304

uni TTTTGGTGAGCGAAAGGTGGACCGGGCTGACCCGTATTAT 6304

tall TTTTGGTGAGCGAAAGGTGGACCGGGCTGACCCGTATTAT 6306

quil TTTTGGTGAGCGAAAGGTGGACCGGGCTGACCCGTATTAT 6304

meri TTTTGGTGAGCGAAAGGTGGACCGGGCTGACCCGTATTAT 6430

ref TTTTtGTaAGCcAAAGGTGGACCGGGCTGACCCGTATTgT 6542

Consensus tttt gt agc aaaggtggaccgggctgacccgtatt t

kal TATTTATGAGGTGTGATGATTACTTATTTTTAAAATTATG 6344

yor TATTTATGAGGTGTGATGATTACTTATTTTTAAAATTATG 6342

jen TATTTATGAGGTGTGATGATTACTTATTTTTAAAATTATG 6344

cor TATTTATGAGGTGTGATGATTACTTATTTTTAAAATTATG 6344

man TATTTATGAGGTGTGATGATTACTTATTTTTAAAATTATG 6344

uni TATTTATGAGGTGTGATGATTACTTATTTTTAAAATTATG 6344

tall TATTTATGAGGTGTGATGATTACTTATTTTTAAAATTATG 6346

quil TATTTATGAGGTGTGATGATTACTTATTTTTAAAATTATG 6344

meri TATTTATGAGGTGTGATGATTACTTATTTTTAAAATTATG 6470

ref TATTTATGAGGTGTGATGATTACTTtTTTTTgAAATTATG 6582

Consensus tatttatgaggtgtgatgattactt ttttt aaattatg

kal ATTGAGTTAATTAAAAATGTGTGTTTTAAAAAAAGGATAA 6384

yor ATTGAGTTAATTAAAAATGTGTGTTTTAAAAAAAGGATAA 6382

jen ATTGAGTTAATTAAAAATGTGTGTTTTAAAAAAAGGATAA 6384

cor ATTGAGTTAATTAAAAATGTGTGTTTTAAAAAAAGGATAA 6384

man ATTGAGTTAATTAAAAATGTGTGTTTTAAAAAAAGGATAA 6384

uni ATTGAGTTAATTAAAAATGTGTGTTTTAAAAAAAGGATAA 6384

tall ATTGAGTTAATTAAAAATGTGTGTTTTAAAAAAAGGATAA 6386

quil ATTGAGTTAATTAAAAATGTGTGTTTTAAAAAAAGGATAA 6384

meri ATTGAGTTAATTAAAAATGTGTGTTTTAAAAAAAGGATAA 6510

ref tTTGAGTTAATTAAAAATGTGTGTTTTAAAAAAgGGAcAA 6622

Consensus ttgagttaattaaaaatgtgtgttttaaaaaa gga aa

kal TCTGCTGGCCTGCCA.........AATATGGGTCGAACTG 6415

yor TtTGCTGGCCTGCCA.........AATATGGGTCGAACTG 6413

jen TCTGCTGGCCTGCCA.........AATATGGGTCGAACTG 6415

cor TCTGCTGGCCTGCCA.........AATATGGGTCGAACTG 6415

man TCTGCTGGCCTGCCA.........AATATGGGTCGAACTG 6415

uni TCTGCTGGCCTGCCA.........AATATGGGTCGAACTG 6415

tall TCTGCTGGCCTGCCA.........AATATGGGTCGAACTG 6417

quil TCTGCTGGCCTGCCA.........AATATGGGTCGAACTG 6415

meri TCTGCTGGCCTGCCA.........AATATGGGTCGAACTG 6541

ref TCTGtTGGCCTGCCAgccctaccaAATATGGGTCGgACTG 6662

Consensus t tg tggcctgcca aatatgggtcg actg

kal ATATTTTGAGTTCTTTTAACAATAAATATCAGGTCAGGCC 6455

yor ATATTTTGAGTTCTTTTAACAATAAATATCAGGTCAGGCC 6453

jen ATATTTTGAGTTCTTTTAACAATAAATATCAGGTCAGGCC 6455

cor ATATTTTGAGTTCTTTTAACAATAAATATCAGGTCAGGCC 6455

man ATATTTTGAGTTCTTTTAACAATAAATATCAGGTCAGGCC 6455

uni ATATTTTGAGTTCTTTTAACAATAAATATCAGGTCAGGCC 6455

tall ATATTTTGAGTTCTTTTAACAATAAATATCAGGTCAGGCC 6457

quil ATATTTTGAGTTCTTTTAACAATAAATATCAGGTCAGGCC 6455

meri ATATTTTGAGTTCTTTTAACAATAAATATCAGGTCAGGCC 6581

ref ATATTTTGAGTTCTTTTAttAATAAATATCAGGTCAGGtC 6702

Consensus atattttgagttctttta aataaatatcaggtcagg c

kal AACTCATATTTTGACAGATCGTACAGGACGGGACTGACTC 6495

yor AACTCATATTTTGACAGATCGTACAGGACGGGACTGACTC 6493

jen AACTCATATTTTGACAGATCGTACAGGACGGGACTGACTC 6495

cor AACTCATATTTTGACAGATCGTACAGGACGGGACTGACTC 6495

man AACTCATATTTTGACAGATCGTACAGGACGGGACTGACTC 6495

uni AACTCATATTTTGACAGATCGTACAGGACGGGACTGACTC 6495

tall AACTCATATTTTGACAGATCGTACAGGACGGGACTGACTC 6497

quil AACTCATATTTTGACAGATCGTACAGGACGGGACTGACTC 6495

meri AACTCATATTTTGACAGATCGTACAGGACGGGACTGACTC 6621

ref AACcCATATTTTGACgGAcCGagCAGGACcGGACTaACTg 6742

Consensus aac catattttgac ga cg caggac ggact act

kal TTATTGTCACACCTAGTGAACTGCCATGTAAGACCTTTGT 6535

yor TTATTGTCACACCTAGTGAACTGCCATGTAAGACCTT.GT 6532

jen TTATTGTCACACCTAGTGAACTGCCATGTAAGACCTTTGT 6535

cor TTATTGTCACACCTAGTGAACTGCCATGTAAGACCTTTGT 6535

man TTATTGTCACACCTAGTGAACTGCCATGTAAGACCTTTGT 6535

uni TTATTGTCACACCTAGTGAACTGCCATGTAAGACCTTTGT 6535

tall TTATTGTCACACCTAGTGAACTGCCATGTAAGACCTTTGT 6537

quil TTATTGTCACACCTAGTGAACTGCCATGTAAGACCTTTGT 6535

meri TTATTGTCACACCTAGTGAACTGCCATGTAAGACCTT.GT 6660

ref TTATTGTCACcCCTgGcGAACTGCCATGTAAGACCTT.GT 6781

Consensus ttattgtcac cct g gaactgccatgtaagacctt gt

kal TTTAGGAAACAAAAGCTTTAAAACATGTAACTTCTTAAAT 6575

yor TTTAGGAAACAAAAGtTTTAAAACATGTAACTTCTTAAAT 6572

jen TTTAGGAAACAAAAGCTTTAAAACATGTAACTTCTTAAAT 6575

cor TTTAGGAAACAAAAGCTTTAAAACATGTAACTTCTTAAAT 6575

man TTTAGGAAACAAAAGCTTTAAAACATGTAACTTCTTAAAT 6575

uni TTTAGGAAACAAAAGCTTTAAAACATGTAACTTCTTAAAT 6575

tall TTTAGGAAACAAAAGCTTTAAAACATGTAACTTCTTAAAT 6577

quil TTTAGGAAACAAAAGCTTTAAAACATGTAACTTCTTAAAT 6575

meri TTTAGGAAACAAAAGCTTTAAAACATGTAACTTCTTAAAT 6700

ref TTTAGGAAACAAAAGaTTTAAAACATGTAACTTCcTAAAT 6821

Consensus tttaggaaacaaaag tttaaaacatgtaacttc taaat

kal GCATCATTTTAAAAAAAAAACTTGTTTATTTATGTATCCT 6615

yor GCATCATTTTAAAAAAAAAACTTGTTTATTTATGTATCCT 6612

jen GCATCATTTTAAAAAAAAAACTTGTTTATTTATGTATCCT 6615

cor GCATCATTTTAAAAAAAAAACTTGTTTATTTATGTATCCT 6615

man GCATCATTTTAAAAAAAAAACTTGTTTATTTATGTATCCT 6615

uni GCATCATTTTAAAAAAAAAACTTGTTTATTTATGTATCCT 6615

tall GCATCATTTTAAAAAAAAAACTTGTTTATTTATGTATCCT 6617

quil GCATCATTTTAAAAAAAAAACTTGTTTATTTATGTATCCT 6615

meri GCATCATTTTAAAAAAAAAACTTGTTTATTTATGTATCCT 6740

ref GCATCATTTTcAAAAAAAAACTTGTTTATTTATGTATCCT 6861

Consensus gcatcatttt aaaaaaaaacttgtttatttatgtatcct

kal TATCCCGGAGCAAAGTTTATAAAATCTATGTCAACAGTAA 6655

yor TATCCCGGAGCAAAGTTTATAAAATCTATGTaAACAGTAA 6652

jen TATCCCGGAGCAAAGTTTATAAAATCTATGTCAACAGTAA 6655

cor TATCCCGGAGCAAAGTTTATAAAATCTATGTCAACAGTAA 6655

man TATCCCGGAGCAAAGTTTATAAAATCTATGTCAACAGTAA 6655

uni TATCCCGGAGCAAAGTTTATAAAATCTATGTCAACAGTAA 6655

tall TATCCCGGAGCAAAGTTTATAAAATCTATGTCAACAGTAA 6657

quil TATCCCGGAGCAAAGTTTATAAAATCTATGTCAACAGTAA 6655

meri TATCCCGGAGCAAAGTTTATAAAATCTATGTCAACAGTAA 6780

ref TATCCCG.AGCAAAGTTTATAAAATCTATGTaAACAGTAg 6900

Consensus tatcccg agcaaagtttataaaatctatgt aacagta

kal TAACTATTTAGTGCATAATGGAACAGGTGATCCTTGTCCT 6695

yor TAACTATTTAGTGCATAATGGAACAtGTGATCCTTGTCCT 6692

jen TAACTATTTAGTGCATAATGGAACAGGTGATCCTTGTCCT 6695

cor TAACTATTTAGTGCATAATGGAACAGGTGATCCTTGTCCT 6695

man TAACTATTTAGTGCATAATGGAACAGGTGATCCTTGTCCT 6695

uni TAACTATTTAGTGCATAATGGAACAGGTGATCCTTGTCCT 6695

tall TAACTATTTAGTGCATAATGGAACAGGTGATCCTTGTCCT 6697

quil TAACTATTTAGTGCATAATGGAACAGGTGATCCTTGTCCT 6695

meri TAACTATTTAGTGCATAATGGAACAGGTGATCCTTGTCCT 6820

ref TAAtTATTTAaTGtATAATGGgACAGGTGATCCccaTCCT 6940

Consensus taa tattta tg ataatgg aca gtgatcc tcct

kal AAAATCTAAACGTGTAGTGTTAAGTTATCTTATATCACTT 6735

yor AAAATCTAAACGTGTAGTGTTAAGTTATCTTATATCACTT 6732

jen AAAATCTAAACGTGTAGTGTTAAGTTATCTTATATCACTT 6735

cor AAAATCTAAACGTGTAGTGTTAAGTTATCTTATATCACTT 6735

man AAAATCTAAACGTGTAGTGTTAAGTTATCTTATATCACTT 6735

uni AAAATCTAAACGTGTAGTGTTAAGTTATCTTATATCACTT 6735

tall AAAATCTAAACGTGTAGTGTTAAGTTATCTTATATCACTT 6737

quil AAAATCTAAACGTGTAGTGTTAAGTTATCTTATATCACTT 6735

meri AAAATCTAAACGTGTAGTGTTAAGTTATCTTATATCACTT 6860

ref AAAATCTAAAtGTGTAGTGTTAAGTTATCTTATATCACTT 6980

Consensus aaaatctaaa gtgtagtgttaagttatcttatatcactt

kal TATTCTATATAATTCAACCATGTGATCCACTCGTTAGATA 6775

yor TATTCTATATAATTCAACCATGTGATCCACTCGTTAGATA 6772

jen TATTCTATATAATTCAACCATGTGATCCACTCGTTAGATA 6775

cor TATTCTATATAATTCAACCATGTGATCCACTCGTTAGATA 6775

man TATTCTATATAATTCAACCATGTGATCCACTCGTTAGATA 6775

uni TATTCTATATAATTCAACCATGTGATCCACTCGTTAGATA 6775

tall TATTCTATATAATTCAACCATGTGATCCACTCGTTAGATA 6777

quil TATTCTATATAATTCAACCATGTGATCCACTCGTTAGATA 6775

meri TATTCTATATAATTCAACCATGTGATCCACTCGTTAGATA 6900

ref TATTCTATATAATTCAACCATGTGATCCACTCaTTAaATA 7020

Consensus tattctatataattcaaccatgtgatccactc tta ata

kal GTGAAACAAAGTACTTTAGTAAATATGAAAATTTTCAACG 6815

yor GTGAAACAAAGTACTTTAGTAAATATGAAAATTTTCAACG 6812

jen GTGAAACAAAGTACTTTAGTAAATATGAAAATTTTCAACG 6815

cor GTGAAACAAAGTACTTTAGTAAATATGAAAATTTTCAACG 6815

man GTGAAACAAAGTACTTTAGTAAATATGAAAATTTTCAACG 6815

uni GTGAAACAAAGTACTTTAGTAAATATGAAAATTTTCAACG 6815

tall GTGAAACAAAGTACTTTAGTAAATATGAAAATTTTCAACG 6817

quil GTGAAACAAAGTACTTTAGTAAATATGAAAATTTTCAACG 6815

meri GTGAAACAAAGTACTTTAGTAAATATGAAAATTTTCAACG 6940

ref GTGAAACAAAGTACTTTAGTAAATATGAAAATTTTCAACG 7060

Consensus gtgaaacaaagtactttagtaaatatgaaaattttcaacg

kal ATTAAGTTGTAAAAATAGAGACTTTGGTTAACTCCGACTT 6855

yor ATTAAGTTGTAAAAATAGAGACTTTGGTTAACTCCGACTT 6852

jen ATTAAGTTGTAAAAATAGAGACTTTGGTTAACTCCGACTT 6855

cor ATTAAGTTGTAAAAATAGAGACTTTGGTTAACTCCGACTT 6855

man ATTAAGTTGTAAAAATAGAGACTTTGGTTAACTCCGACTT 6855

uni ATTAAGTTGTAAAAATAGAGACTTTGGTTAACTCCGACTT 6855

tall ATTAAGTTGTAAAAATAGAGACTTTGGTTAACTCCGACTT 6857

quil ATTAAGTTGTAAAAATAGAGACTTTGGTTAACTCCGACTT 6855

meri ATTAAGTTGTAAAAATAGAGACTTTGGTTAACTCCGACTT 6980

ref ATTAAGTTGTAAAAATAGAGACT..GGTTAACTCCGACTT 7098

Consensus attaagttgtaaaaatagagact ggttaactccgactt

kal GTGAAAAAGTGCAAAGCTTTAATAATGTTTCTTGCACTTT 6895

yor GTGAAtAAGTGCAAAGCTTTAATAATGTTTCTTGCACTTT 6892

jen GTGAAAAAGTGCAAAGCTTTAATAATGTTTCTTGCACTTT 6895

cor GTGAAAAAGTGCAAAGCTTTAATAATGTTTCTTGCACTTT 6895

man GTGAAAAAGTGCAAAGCTTTAATAATGTTTCTTGCACTTT 6895

uni GTGAAAAAGTGCAAAGCTTTAATAATGTTTCTTGCACTTT 6895

tall GTGAAAAAGTGCAAAGCTTTAATAATGTTTCTTGCACTTT 6897

quil GTGAAAAAGTGCAAAGCTTTAATAATGTTTCTTGCACTTT 6895

meri GTGAAAAAGTGCAAAGCTTTAATAATGTTTCTTGCACTTT 7020

ref GTGAAtAAGTGCAAAGCTTTAATAATGTTTCTTGCACTTT 7138

Consensus gtgaa aagtgcaaagctttaataatgtttcttgcacttt

kal TTGTACTTCTTGAAGGAAAGATATAATAAGTATATAGAAA 6935

yor TTGTACTTCTTGAAGGAAAGATATAATAAGTATATAGAAA 6932

jen TTGTACTTCTTGAAGGAAAGATATAATAAGTATATAGAAA 6935

cor TTGTACTTCTTGAAGGAAAGATATAATAAGTATATAGAAA 6935

man TTGTACTTCTTGAAGGAAAGATATAATAAGTATATAGAAA 6935

uni TTGTACTTCTTGAAGGAAAGATATAATAAGTATATAGAAA 6935

tall TTGTACTTCTTGAAGGAAAGATATAATAAGTATATAGAAA 6937

quil TTGTACTTCTTGAAGGAAAGATATAATAAGTATATAGAAA 6935

meri TTGTACTTCTTGAAGGAAAGATATAATAAGTATATAGAAA 7060

ref TTGTACTTCTTGAAGGAAAGATATAATAAGTATATAGAAA 7178

Consensus ttgtacttcttgaaggaaagatataataagtatatagaaa

kal GGTAAAATTATCAATG.CATTTGTTTTACTTTTATATTTT 6974

yor GGTAAAATTATCAATG.CATTTGTTTTACTTTTATATTTT 6971

jen GGTAAAATTATCAATG.CATTTGTTTTACTTTTATATTTT 6974

cor GGTAAAATTATCAATG.CATTTGTTTTACTTTTATATTTT 6974

man GGTAAAATTATCAATG.CATTTGTTTTACTTTTATATTTT 6974

uni GGTAAAATTATCAATG.CATTTGTTTTACTTTTATATTTT 6974

tall GGTAAAATTATCAATG.CATTTGTTTTACTTTTATATTTT 6976

quil GGTAAAATTATCAATG.CATTTGTTTTACTTTTATATTTT 6974

meri GGTAAAATTATCAATG.CATTTGTTTTACTTTTATATTTT 7099

ref GGTAAAATTATCAATGtCATTTGTTTTACTTTTATATTTT 7218

Consensus ggtaaaattatcaatg catttgttttacttttatatttt

kal ATGTAATAAATACTTTATTTCCCATCAAAGCTTTGTATTT 7014

yor ATGTAATAAATACTTTATTTCCCATCAAAGCTTTGTATTT 7011

jen ATGTAATAAATACTTTATTTCCCATCAAAGCTTTGTATTT 7014

cor ATGTAATAAATACTTTATTTCCCATCAAAGCTTTGTATTT 7014

man ATGTAATAAATACTTTATTTCCCATCAAAGCTTTGTATTT 7014

uni ATGTAATAAATACTTTATTTCCCATCAAAGCTTTGTATTT 7014

tall ATGTAATAAATACTTTATTTCCCATCAAAGCTTTGTATTT 7016

quil ATGTAATAAATACTTTATTTCCCATCAAAGCTTTGTATTT 7014

meri ATGTAATAAATACTTTATTTCCCATCAAAGCTTTGTATTT 7139

ref ATGTAATAAATACTTTATTTCCCATCAAAGCTTTGTATTT 7258

Consensus atgtaataaatactttatttcccatcaaagctttgtattt

kal TTGCATCACTCACAGAGTATATATTCATATTGATTCTCAA 7054

yor TTaCATCACTCACAGAGTATATATTCATATTGATTCTCAA 7051

jen TTGCATCACTCACAGAGTATATATTCATATTGATTCTCAA 7054

cor TTGCATCACTCACAGAGTATATATTCATATTGATTCTCAA 7054

man TTGCATCACTCACAGAGTATATATTCATATTGATTCTCAA 7054

uni TTGCATCACTCACAGAGTATATATTCATATTGATTCTCAA 7054

tall TTGCATCACTCACAGAGTATATATTCATATTGATTCTCAA 7056

quil TTGCATCACTCACAGAGTATATATTCATATTGATTCTCAA 7054

meri TTaCATCACTCACAGAGTATATATTCATATTGATTCTCAA 7179

ref TTGCATCACTCACAGAGTATATATTCATATTGATTCTaAA 7298

Consensus tt catcactcacagagtatatattcatattgattct aa

kal AAATGATGATGTCATTAATCATTATATTGGATGATAACTT 7094

yor AAATGATGATGTCATTAATCATTATATTGGATGATAACTT 7091

jen AAATGATGATGTCATTAATCATTATATTGGATGATAACTT 7094

cor AAATGATGATGTCATTAATCATTATATTGGATGATAACTT 7094

man AAATGATGATGTCATTAATCATTATATTGGATGATAACTT 7094

uni AAATGATGATGTCATTAATCATTATATTGGATGATAACTT 7094

tall AAATGATGATGTCATTAATCATTATATTGGATGATAACTT 7096

quil AAATGATGATGTCATTAATCATTATATTGGATGATAACTT 7094

meri AAATGATGATGTCATTAATCATTATATTGGATGATAACTT 7219

ref AAATGATGATGTCATTAATCATTATATTGGATGATAACTT 7338

Consensus aaatgatgatgtcattaatcattatattggatgataactt

kal CTCCACTATTGTCAATGTGGCCAAATGAAATGAGGAGGCG 7134

yor CTCCA..................AATGAAATGAGGAGGgG 7113

jen CTCCACTATTGTCAATGTGGCCAAATGAAATGAGGAGGCG 7134

cor CTCCACTATTGTCAATGTGGCCAAATGAAATGAGGAGGCG 7134

man CTCCACTATTGTCAATGTGGCCAAATGAAATGAGGAGGCG 7134

uni CTCCACTATTGTCAATGTGGCCAAATGAAATGAGGAGGCG 7134

tall CTCCACTATTGTCAATGTGGCCAAATGAAATGAGGAGGCG 7136

quil CTCCACTATTGTCAATGTGGCCAAATGAAATGAGGAGGCG 7134

meri CTCCACTATTGTCAATGTGGCCAAATGAAATGAGGAGGCG 7259

ref CTCCACTATTGTCAATGTGGCCAAATGAAATGAGGAGGCG 7378

Consensus ctcca aatgaaatgaggagg g

kal CAGTATACATAAACATTCAAAAATTGGTGCAGTTTCAGTT 7174

yor CAGTATACATAAACATTCAAAAATTGGTGCAGTTTCAGTT 7153

jen CAGTATACATAAACATTCAAAAATTGGTGCAGTTTCAGTT 7174

cor CAGTATACATAAACATTCAAAAATTGGTGCAGTTTCAGTT 7174

man CAGTATACATAAACATTCAAAAATTGGTGCAGTTTCAGTT 7174

uni CAGTATACATAAACATTCAAAAATTGGTGCAGTTTCAGTT 7174

tall CAGTATACATAAACATTCAAAAATTGGTGCAGTTTCAGTT 7176

quil CAGTATACATAAACATTCAAAAATTGGTGCAGTTTCAGTT 7174

meri CAGTATACATAAACATTCAAAAATTGGTGCAGTTTCAGTT 7299

ref CAGTATACATAAACATTCAAAAATTGGTGCAGTTTCAGTT 7418

Consensus cagtatacataaacattcaaaaattggtgcagtttcagtt

kal AATAGTTAATGTTGTTACTCTTATTACCAACTTTGTTTCT 7214

yor AAcAGTTAATGTTGTTgCTCTTATTACCAACTTTGTTTCT 7193

jen AATAGTTAATGTTGTTACTCTTATTACCAACTTTGTTTCT 7214

cor AATAGTTAATGTTGTTACTCTTATTACCAACTTTGTTTCT 7214

man AATAGTTAATGTTGTTACTCTTATTACCAACTTTGTTTCT 7214

uni AATAGTTAATGTTGTTACTCTTATTACCAACTTTGTTTCT 7214

tall AATAGTTAATGTTGTTACTCTTATTACCAACTTTGTTTCT 7216

quil AATAGTTAATGTTGTTACTCTTATTACCAACTTTGTTTCT 7214

meri AAcAGTTAATGTTGTTgCTCTTATTACCAACTTTGTTTCT 7339

ref AAcAGTTAATGTTGTTgCTCTTATTACCAACTTTGTTTCT 7458

Consensus aa agttaatgttgtt ctcttattaccaactttgtttct

kal GCATGCATCATTGCTTTGTTGGTTATGTAGTTTTTGTTTC 7254

yor GCATGCATCATTGCTTTGTTGGTTATtTAGTTTTTGTTTC 7233

jen GCATGCATCATTGCTTTGTTGGTTATGTAGTTTTTGTTTC 7254

cor GCATGCATCATTGCTTTGTTGGTTATGTAGTTTTTGTTTC 7254

man GCATGCATCATTGCTTTGTTGGTTATGTAGTTTTTGTTTC 7254

uni GCATGCATCATTGCTTTGTTGGTTATGTAGTTTTTGTTTC 7254

tall GCATGCATCATTGCTTTGTTGGTTATGTAGTTTTTGTTTC 7256

quil GCATGCATCATTGCTTTGTTGGTTATGTAGTTTTTGTTTC 7254

meri GCATGCATCATTGCTTTGTTGGTTATtTAGTTTTTGTTTC 7379

ref GCATGCATCATTGCTTTGTTGGTTATGTAGaTTTTGTTTC 7498

Consensus gcatgcatcattgctttgttggttat tag ttttgtttc

kal AAGGAACATGATTATAGTCAACTTCTTTACTTTCAGATGA 7294

yor AAGGAACATGATTATAGTtAACTTCTTTACTTTCAGATGA 7273

jen AAGGAACATGATTATAGTCAACTTCTTTACTTTCAGATGA 7294

cor AAGGAACATGATTATAGTCAACTTCTTTACTTTCAGATGA 7294

man AAGGAACATGATTATAGTCAACTTCTTTACTTTCAGATGA 7294

uni AAGGAACATGATTATAGTCAACTTCTTTACTTTCAGATGA 7294

tall AAGGAACATGATTATAGTCAACTTCTTTACTTTCAGATGA 7296

quil AAGGAACATGATTATAGTCAACTTCTTTACTTTCAGATGA 7294

meri AAGtAACATGATTATAGTCAACTTCTTTACTTTCAGATGA 7419

ref AAGtAACATGATTATAGTCAACTTCTTTACTTTCAGATGA 7538

Consensus aag aacatgattatagt aacttctttactttcagatga

kal TCCGACTTTTCATTAATCTGATCAAGTTCATCACGGTCCG 7334

yor TCCGAtTTTTtATTAATCTGgTCAAaTTCATCAtGGTCCG 7313

jen TCCGACTTTTCATTAATCTGATCAAGTTCATCACGGTCCG 7334

cor TCCGACTTTTCATTAATCTGATCAAGTTCATCACGGTCCG 7334

man TCCGACTTTTCATTAATCTGATCAAGTTCATCACGGTCCG 7334

uni TCCGACTTTTCATTAATCTGATCAAGTTCATCACGGTCCG 7334

tall TCCGACTTTTCATTAATCTGATCAAGTTCATCACGGTCCG 7336

quil TCCGACTTTTCATTAATCTGATCAAGTTCATCACGGTCCG 7334

meri TCCGACTTTTCATTAATCTGATCAAGTTCATCACGGTCCG 7459

ref TCtGtCTcTTCATTAATCTGgTCAAGTTCATCACGGTCCG 7578

Consensus tc g t tt attaatctg tcaa ttcatca ggtccg

kal ACTCTTCACACACTTGGAACATTCATTCACAGCTACTTAA 7374

yor ACTCTTCAggCACTTGGAAtATTtATTaACgGaTACaTAA 7353

jen ACTCTTCACACACTTGGAACATTCATTCACAGCTACTTAA 7374

cor ACTCTTCACACACTTGGAACATTCATTCACAGCTACTTAA 7374

man ACTCTTCACACACTTGGAACATTCATTCACAGCTACTTAA 7374

uni ACTCTTCACACACTTGGAACATTCATTCACAGCTACTTAA 7374

tall ACTCTTCACACACTTGGAACATTCATTCACAGCTACTTAA 7376

quil ACTCTTCACACACTTGGAACATTCATTCACAGCTACTTAA 7374

meri ACTCTTCACACACTTGGAACATTCATTCACAGCTACTTAA 7499

ref ACTCTTCACACACTTGGAACATTtATTCACAGCTACTTAA 7618

Consensus actcttca cacttggaa att att ac g tac taa

kal ACATGACTCATTCTTGTTAAGTATTTAGGGTGATTATCAA 7414

yor ACATGACTCATTCTTGTTAAGTATTTAGGGTGATTATCAA 7393

jen ACATGACTCATTCTTGTTAAGTATTTAGGGTGATTATCAA 7414

cor ACATGACTCATTCTTGTTAAGTATTTAGGGTGATTATCAA 7414

man ACATGACTCATTCTTGTTAAGTATTTAGGGTGATTATCAA 7414

uni ACATGACTCATTCTTGTTAAGTATTTAGGGTGATTATCAA 7414

tall ACATGACTCATTCTTGTTAAGTATTTAGGGTGATTATCAA 7416

quil ACATGACTCATTCTTGTTAAGTATTTAGGGTGATTATCAA 7414

meri ACATGACTCATTCTTGTTAAGTATTTAGGGTGATTATCAA 7539

ref ACATGgCTCATTCTTGTTAAGTATTTAGGGTGATTATCAA 7658

Consensus acatg ctcattcttgttaagtatttagggtgattatcaa

kal ACATATATTACTATTTTCATGAAAT.....CTATTGATAT 7449

yor ACATATATTACTATTTTCATGAAATaaaatCTATTGATAT 7433

jen ACATATATTACTATTTTCATGAAAT.....CTATTGATAT 7449

cor ACATATATTACTATTTTCATGAAAT.....CTATTGATAT 7449

man ACATATATTACTATTTTCATGAAAT.....CTATTGATAT 7449

uni ACATATATTACTATTTTCATGAAAT.....CTATTGATAT 7449

tall ACATATATTACTATTTTCATGAAAT.....CTATTGATAT 7451

quil ACATATATTACTATTTTCATGAAAT.....CTATTGATAT 7449

meri ACATATATTACTATTTTCATGAAAT.....CTATTGATAT 7574

ref ACATATATTACTATTTTCATGAAAT.....CTATTGATAT 7693

Consensus acatatattactattttcatgaaat ctattgatat

kal AATCAAATGCCACCAAATGTTGATAACTTTGAGTAAAGGT 7489

yor AATCAAATGCCACCAAgTGTTGATAACTTTGAGTAAAGGT 7473

jen AATCAAATGCCACCAAATGTTGATAACTTTGAGTAAAGGT 7489

cor AATCAAATGCCACCAAATGTTGATAACTTTGAGTAAAGGT 7489

man AATCAAATGCCACCAAATGTTGATAACTTTGAGTAAAGGT 7489

uni AATCAAATGCCACCAAATGTTGATAACTTTGAGTAAAGGT 7489

tall AATCAAATGCCACCAAATGTTGATAACTTTGAGTAAAGGT 7491

quil AATCAAATGCCACCAAATGTTGATAACTTTGAGTAAAGGT 7489

meri AATCAAATGCCACCAAATGTTGATAACTTTGAGTAAAGGT 7614

ref AATCAAATGtCACCAAATGTgGATAACTTTGAGTgAAGGT 7733

Consensus aatcaaatg caccaa tgt gataactttgagt aaggt

kal ACATAAAGTGAGAGATAGAAATATGCTACTCAACATGGTG 7529

yor ACATAAAGTaAGAGATAGAAATATGCTACTtAACATGGTG 7513

jen ACATAAAGTGAGAGATAGAAATATGCTACTCAACATGGTG 7529

cor ACATAAAGTGAGAGATAGAAATATGCTACTCAACATGGTG 7529

man ACATAAAGTGAGAGATAGAAATATGCTACTCAACATGGTG 7529

uni ACATAAAGTGAGAGATAGAAATATGCTACTCAACATGGTG 7529

tall ACATAAAGTGAGAGATAGAAATATGCTACTCAACATGGTG 7531

quil ACATAAAGTGAGAGATAGAAATATGCTACTCAACATGGTG 7529

meri ACATAAAGTaAGAGATAGAAATATGCTACTtAACATGGTG 7654

ref ACATAAAGTGAGAGATAGAAATAcGCTACTtAACATGGTG 7773

Consensus acataaagt agagatagaaata gctact aacatggtg

kal TGAGACATTCATCTTATATGGAAGTTATGTATCTCAGTGT 7569

yor ..AGACATTCATCTTATAcGGtAGTTATGTgTtTCAGTGT 7551

jen TGAGACATTCATCTTATATGGAAGTTATGTATCTCAGTGT 7569

cor TGAGACATTCATCTTATATGGAAGTTATGTATCTCAGTGT 7569

man TGAGACATTCATCTTATATGGAAGTTATGTATCTCAGTGT 7569

uni TGAGACATTCATCTTATATGGAAGTTATGTATCTCAGTGT 7569

tall TGAGACATTCATCTTATATGGAAGTTATGTATCTCAGTGT 7571

quil TGAGACATTCATCTTATATGGAAGTTATGTATCTCAGTGT 7569

meri TGAGACATTCATCTTATAcGGtAGTTATGTgTCTCAGTGT 7694

ref TGAGACATTCATCTTATAcGGtAGTTATGTgTCTtAGTGT 7813

Consensus agacattcatcttata gg agttatgt t t agtgt

kal TAGAACTTAAATACATGAAGAGAACCCAACCCAAAAAACT 7609

yor TAGAACTTAAATACATGAAGAGAACCCAACCCAAAAgACT 7591

jen TAGAACTTAAATACATGAAGAGAACCCAACCCAAAAAACT 7609

cor TAGAACTTAAATACATGAAGAGAACCCAACCCAAAAAACT 7609

man TAGAACTTAAATACATGAAGAGAACCCAACCCAAAAAACT 7609

uni TAGAACTTAAATACATGAAGAGAACCCAACCCAAAAAACT 7609

tall TAGAACTTAAATACATGAAGAGAACCCAACCCAAAAAACT 7611

quil TAGAACTTAAATACATGAAGAGAACCCAACCCAAAAAACT 7609

meri TAGAACTTAAATACATGAAGAGAACCCAACCCAAAAAACT 7734

ref TAGAACTTAAATACATGAAGAGAACCCAACCCAAAAgACT 7853

Consensus tagaacttaaatacatgaagagaacccaacccaaaa act

kal AGTCTATTAGGTAGTAAAACCTCGTGGCTTAAATATCATA 7649

yor AGTtTATTAGGTAGTAgAAtCTCGTGaCTTAAATATCATA 7631

jen AGTCTATTAGGTAGTAAAACCTCGTGGCTTAAATATCATA 7649

cor AGTCTATTAGGTAGTAAAACCTCGTGGCTTAAATATCATA 7649

man AGTCTATTAGGTAGTAAAACCTCGTGGCTTAAATATCATA 7649

uni AGTCTATTAGGTAGTAAAACCTCGTGGCTTAAATATCATA 7649

tall AGTCTATTAGGTAGTAAAACCTCGTGGCTTAAATATCATA 7651

quil AGTCTATTAGGTAGTAAAACCTCGTGGCTTAAATATCATA 7649

meri AGTtTATTAGGTAGTAgAAtCTCGTGaCTTAAATATCATA 7774

ref AGTtTATTAGGTAGTAgAACCTCGTGaCTTAAATATCATA 7893

Consensus agt tattaggtagta aa ctcgtg cttaaatatcata

kal TTGAGTTTGTTATGCTAATCAATGTGAGACTCCTAACACT 7689

yor TTGAGTTTGTTATGCTAATCAATGTGAGACTCCTAACACT 7671

jen TTGAGTTTGTTATGCTAATCAATGTGAGACTCCTAACACT 7689

cor TTGAGTTTGTTATGCTAATCAATGTGAGACTCCTAACACT 7689

man TTGAGTTTGTTATGCTAATCAATGTGAGACTCCTAACACT 7689

uni TTGAGTTTGTTATGCTAATCAATGTGAGACTCCTAACACT 7689

tall TTGAGTTTGTTATGCTAATCAATGTGAGACTCCTAACACT 7691

quil TTGAGTTTGTTATGCTAATCAATGTGAGACTCCTAACACT 7689

meri TTGAGTTTGTTATGCTAATCAATGTGAGACTCCTAACACT 7814

ref TTGAGTTTGTTATGCTAATCAATGTGAGACTCgTAACACT 7933

Consensus ttgagtttgttatgctaatcaatgtgagactc taacact

kal CAGCTTGTACAAAAGAGTTCGGCGGGTGCATGTAGAGTTT 7729

yor CAGCTTGTACAAAAGAGTTCGGCcGGTGCATGTAGAGTTT 7711

jen CAGCTTGTACAAAAGAGTTCGGCGGGTGCATGTAGAGTTT 7729

cor CAGCTTGTACAAAAGAGTTCGGCGGGTGCATGTAGAGTTT 7729

man CAGCTTGTACAAAAGAGTTCGGCGGGTGCATGTAGAGTTT 7729

uni CAGCTTGTACAAAAGAGTTCGGCGGGTGCATGTAGAGTTT 7729

tall CAGCTTGTACAAAAGAGTTCGGCGGGTGCATGTAGAGTTT 7731

quil CAGCTTGTACAAAAGAGTTCGGCGGGTGCATGTAGAGTTT 7729

meri CAGCTTGTACAAAAGAGTTCGGCcGGTGCATGTAGAGTTT 7854

ref CAGCTTGTACAAAAGAGTTCGGCGGGTGCATGTAGAGTTT 7973

Consensus cagcttgtacaaaagagttcggc ggtgcatgtagagttt

kal CCCTCAAGCTTATGCCTTTGAGGTCTTTCTAACTTATATG 7769

yor CCCTCAAGCTTATGCCTTTGAGGTCTTTCTAACTTATATG 7751

jen CCCTCAAGCTTATGCCTTTGAGGTCTTTCTAACTTATATG 7769

cor CCCTCAAGCTTATGCCTTTGAGGTCTTTCTAACTTATATG 7769

man CCCTCAAGCTTATGCCTTTGAGGTCTTTCTAACTTATATG 7769

uni CCCTCAAGCTTATGCCTTTGAGGTCTTTCTAACTTATATG 7769

tall CCCTCAAGCTTATGCCTTTGAGGTCTTTCTAACTTATATG 7771

quil CCCTCAAGCTTATGCCTTTGAGGTCTTTCTAACTTATATG 7769

meri CCCTCAAGCTTATGCCTTTGAGGTCTTTCTAACTTATATG 7894

ref CCCTCAAGCTTATGCCTTTGAGGTCTTTCTAACTTATATG 8013

Consensus ccctcaagcttatgcctttgaggtctttctaacttatatg

kal CTTCAACCCTCCAATCCAATGAAAAATCTAGTCACTCTTA 7809

yor CTTCAACCCTCCAATCCAATGAAAAgTCTAGTCACTCTTA 7791

jen CTTCAACCCTCCAATCCAATGAAAAATCTAGTCACTCTTA 7809

cor CTTCAACCCTCCAATCCAATGAAAAATCTAGTCACTCTTA 7809

man CTTCAACCCTCCAATCCAATGAAAAATCTAGTCACTCTTA 7809

uni CTTCAACCCTCCAATCCAATGAAAAATCTAGTCACTCTTA 7809

tall CTTCAACCCTCCAATCCAATGAAAAATCTAGTCACTCTTA 7811

quil CTTCAACCCTCCAATCCAATGAAAAATCTAGTCACTCTTA 7809

meri CTTCAACCCTCCAATCCAATGAAAAgTCTAGTCACTCTTA 7934

ref CTTCAACCCTCCAATCCAATGAAAAgTCTAGTCACTCTTA 8053

Consensus cttcaaccctccaatccaatgaaaa tctagtcactctta

kal GAACAAAATGTACTAGTATGAATACATATTTATATGTGAT 7849

yor GAACAAAATGTACTAGTATGAATACATATTTATATGTGAT 7831

jen GAACAAAATGTACTAGTATGAATACATATTTATATGTGAT 7849

cor GAACAAAATGTACTAGTATGAATACATATTTATATGTGAT 7849

man GAACAAAATGTACTAGTATGAATACATATTTATATGTGAT 7849

uni GAACAAAATGTACTAGTATGAATACATATTTATATGTGAT 7849

tall GAACAAAATGTACTAGTATGAATACATATTTATATGTGAT 7851

quil GAACAAAATGTACTAGTATGAATACATATTTATATGTGAT 7849

meri GAACAAAATGTACTAGTATGAATACATATTTATATGTGAT 7974

ref GAACAAAATGTACTAGTATGAATACATATTTATATGTGAT 8093

Consensus gaacaaaatgtactagtatgaatacatatttatatgtgat

kal AGATACAACAAGATAACATTATAGAAATACTTCCCTTGCC 7889

yor AGATACAACAAGATAACATTATAGAAATACTTCtCTTGCC 7871

jen AGATACAACAAGATAACATTATAGAAATACTTCCCTTGCC 7889

cor AGATACAACAAGATAACATTATAGAAATACTTCCCTTGCC 7889

man AGATACAACAAGATAACATTATAGAAATACTTCCCTTGCC 7889

uni AGATACAACAAGATAACATTATAGAAATACTTCCCTTGCC 7889

tall AGATACAACAAGATAACATTATAGAAATACTTCCCTTGCC 7891

quil AGATACAACAAGATAACATTATAGAAATACTTCCCTTGCC 7889

meri AGATACAACAAGATAACATTATAGAAATACTTCCCTTGCC 8014

ref AGATACAACAAGATAACATTATAGAAATACTTCCCTTGtC 8133

Consensus agatacaacaagataacattatagaaatacttc cttg c

kal CCTACTCCTAGTCACATTACAAAAAATCTTGTTCATGTGC 7929

yor CCTACTCCTAGTCACATTACAAAAAATCTTGcTCATGTGC 7911

jen CCTACTCCTAGTCACATTACAAAAAATCTTGTTCATGTGC 7929

cor CCTACTCCTAGTCACATTACAAAAAATCTTGTTCATGTGC 7929

man CCTACTCCTAGTCACATTACAAAAAATCTTGTTCATGTGC 7929

uni CCTACTCCTAGTCACATTACAAAAAATCTTGTTCATGTGC 7929

tall CCTACTCCTAGTCACATTACAAAAAATCTTGTTCATGTGC 7931

quil CCTACTCCTAGTCACATTACAAAAAATCTTGTTCATGTGC 7929

meri CCTACTCCTAGTCACATTACAAAAAATCTTGTTCATGTGC 8054

ref CCTACTCCTAGTCACATTACAAAAAATCTTGTTCATGTGC 8173

Consensus cctactcctagtcacattacaaaaaatcttg tcatgtgc

kal ATATAATAAGGTACAATAAGATACATGAATATGACAATCG 7969

yor ATATAATAAGGTACAATAAGATACATGAATATGACAATCG 7951

jen ATATAATAAGGTACAATAAGATACATGAATATGACAATCG 7969

cor ATATAATAAGGTACAATAAGATACATGAATATGACAATCG 7969

man ATATAATAAGGTACAATAAGATACATGAATATGACAATCG 7969

uni ATATAATAAGGTACAATAAGATACATGAATATGACAATCG 7969

tall ATATAATAAGGTACAATAAGATACATGAATATGACAATCG 7971

quil ATATAATAAGGTACAATAAGATACATGAATATGACAATCG 7969

meri ATATAATAAGGTACAATAAGATACATGAATATGACAATCG 8094

ref ATATAATAAGGTACAATAAGATACATGAATATGACAATCG 8213

Consensus atataataaggtacaataagatacatgaatatgacaatcg

kal GCGAATGTGAGCGGCCGTTCATCTGAACCTCCGTTTGGTT 8009

yor GCGAATGTGAGCGGCCGTTCATCTGAACCTCCGTTTGGTT 7991

jen GCGAATGTGAGCGGCCGTTCATCTGAACCTCCGTTTGGTT 8009

cor GCGAATGTGAGCGGCCGTTCATCTGAACCTCCGTTTGGTT 8009

man GCGAATGTGAGCGGCCGTTCATCTGAACCTCCGTTTGGTT 8009

uni GCGAATGTGAGCGGCCGTTCATCTGAACCTCCGTTTGGTT 8009

tall GCGAATGTGAGCGGCCGTTCATCTGAACCTCCGTTTGGTT 8011

quil GCGAATGTGAGCGGCCGTTCATCTGAACCTCCGTTTGGTT 8009

meri GCGAATGTGAGCGGCCGTTCATCTGAACCTCCGTTTGGTT 8134

ref GCGAATGTGAGCGGCCGTTCATCTGAACCTCCGTTTGGTT 8253

Consensus gcgaatgtgagcggccgttcatctgaacctccgtttggtt

kal TCTCCGTATGCGGCTACTCCGAGTACAGTTATTGAGTAGC 8049

yor TCTCCGTATGCGGCTACTCCGAGTACAGTTATTGAGTAGC 8031

jen TCTCCGTATGCGGCTACTCCGAGTACAGTTATTGAGTAGC 8049

cor TCTCCGTATGCGGCTACTCCGAGTACAGTTATTGAGTAGC 8049

man TCTCCGTATGCGGCTACTCCGAGTACAGTTATTGAGTAGC 8049

uni TCTCCGTATGCGGCTACTCCGAGTACAGTTATTGAGTAGC 8049

tall TCTCCGTATGCGGCTACTCCGAGTACAGTTATTGAGTAGC 8051

quil TCTCCGTATGCGGCTACTCCGAGTACAGTTATTGAGTAGC 8049

meri TCTCCGTATGCGGCTACTCCGAGTACAGTTATTGAGTAGC 8174

ref TCTCCGTATGCGGCTACTCCGAGTACAGTTATTGAGTAGC 8293

Consensus tctccgtatgcggctactccgagtacagttattgagtagc

kal CACCCATGCCAATAACAGTCACAGGGTTTCTGAATATGAG 8089

yor CACCCATGCCAATAACAGTCACAGGGTTTCTGAATATGAG 8071

jen CACCCATGCCAATAACAGTCACAGGGTTTCTGAATATGAG 8089

cor CACCCATGCCAATAACAGTCACAGGGTTTCTGAATATGAG 8089

man CACCCATGCCAATAACAGTCACAGGGTTTCTGAATATGAG 8089

uni CACCCATGCCAATAACAGTCACAGGGTTTCTGAATATGAG 8089

tall CACCCATGCCAATAACAGTCACAGGGTTTCTGAATATGAG 8091

quil CACCCATGCCAATAACAGTCACAGGGTTTCTGAATATGAG 8089

meri CACCCATGCCAATAACAGTCACAGGGTTTCTGAATATGAG 8214

ref CACCCATGCCAATAACAGTCACAGGGTTTCTGAATATGAG 8333

Consensus cacccatgccaataacagtcacagggtttctgaatatgag

kal TATTGAGATAACTACAGCCACAGCACCTTTTGCATTGCCT 8129

yor TATTGAGATAACTACAGCCACAGCACCTTTTGCATTGCCT 8111

jen TATTGAGATAACTACAGCCACAGCACCTTTTGCATTGCCT 8129

cor TATTGAGATAACTACAGCCACAGCACCTTTTGCATTGCCT 8129

man TATTGAGATAACTACAGCCACAGCACCTTTTGCATTGCCT 8129

uni TATTGAGATAACTACAGCCACAGCACCTTTTGCATTGCCT 8129

tall TATTGAGATAACTACAGCCACAGCACCTTTTGCATTGCCT 8131

quil TATTGAGATAACTACAGCCACAGCACCTTTTGCATTGCCT 8129

meri TATTGAGATAACTACAGCCACAGCACCTTTTGCATTGCCT 8254

ref TATTGAGATAACTACAGCCACAGCACCTTTcGCATTGCCT 8373

Consensus tattgagataactacagccacagcaccttt gcattgcct

kal AACACCTGTTAGTATGAATTGCACAAAGTCACAAAAAAAG 8169

yor AACACCTGTTAGTATGAATTGCACAAAGTCACAAAAAgAG 8151

jen AACACCTGTTAGTATGAATTGCACAAAGTCACAAAAAAAG 8169

cor AACACCTGTTAGTATGAATTGCACAAAGTCACAAAAAAAG 8169

man AACACCTGTTAGTATGAATTGCACAAAGTCACAAAAAAAG 8169

uni AACACCTGTTAGTATGAATTGCACAAAGTCACAAAAAAAG 8169

tall AACACCTGTTAGTATGAATTGCACAAAGTCACAAAAAAAG 8171

quil AACACCTGTTAGTATGAATTGCACAAAGTCACAAAAAAAG 8169

meri AACACCTGTTAGTATGAATTGCACAAAGTCACAAAAAgAG 8294

ref AACACCTGTTAGTATGAATTGCACAAAGTCACAAAAAAAG 8413

Consensus aacacctgttagtatgaattgcacaaagtcacaaaaa ag

kal CTTGATTGTTAACTTGATTTTGTGTCTGCTTATGCTATTA 8209

yor CTTGATTGTTAACTTGATTTTGTGTCTGCTTATGCTATTA 8191

jen CTTGATTGTTAACTTGATTTTGTGTCTGCTTATGCTATTA 8209

cor CTTGATTGTTAACTTGATTTTGTGTCTGCTTATGCTATTA 8209

man CTTGATTGTTAACTTGATTTTGTGTCTGCTTATGCTATTA 8209

uni CTTGATTGTTAACTTGATTTTGTGTCTGCTTATGCTATTA 8209

tall CTTGATTGTTAACTTGATTTTGTGTCTGCTTATGCTATTA 8211

quil CTTGATTGTTAACTTGATTTTGTGTCTGCTTATGCTATTA 8209

meri CTTGATTGTTAACTTGATTTTGTGTCTGCTTATGCTATTA 8334

ref CTTGATTGTTAACTTGATTTTGTGTCTGCTTATGCTATTA 8453

Consensus cttgattgttaacttgattttgtgtctgcttatgctatta

kal AAGTCCAAAGAAGCAACTTTCACAATCAAATAGTAATTAA 8249

yor AAGTCCAAAGAAGCAACTTTCACAATCAAATAGTAATTAA 8231

jen AAGTCCAAAGAAGCAACTTTCACAATCAAATAGTAATTAA 8249

cor AAGTCCAAAGAAGCAACTTTCACAATCAAATAGTAATTAA 8249

man AAGTCCAAAGAAGCAACTTTCACAATCAAATAGTAATTAA 8249

uni AAGTCCAAAGAAGCAACTTTCACAATCAAATAGTAATTAA 8249

tall AAGTCCAAAGAAGCAACTTTCACAATCAAATAGTAATTAA 8251

quil AAGTCCAAAGAAGCAACTTTCACAATCAAATAGTAATTAA 8249

meri AAGTCCAAAGAAGCAACTTTCACAATCAAATAGTAATTAA 8374

ref AAGTCCAAAGAAGCAACTTTCACAATCAAATAGTAATTAA 8493

Consensus aagtccaaagaagcaactttcacaatcaaatagtaattaa

kal AAGAACCGGATGAAATCTCGATTTTGTCAACGCCCTTTGG 8289

yor AAGAACCGGATGAAATCTCGATTTTGTCAACGCCCTTTGG 8271

jen AAGAACCGGATGAAATCTCGATTTTGTCAACGCCCTTTGG 8289

cor AAGAACCGGATGAAATCTCGATTTTGTCAACGCCCTTTGG 8289

man AAGAACCGGATGAAATCTCGATTTTGTCAACGCCCTTTGG 8289

uni AAGAACCGGATGAAATCTCGATTTTGTCAACGCCCTTTGG 8289

tall AAGAACCGGATGAAATCTCGATTTTGTCAACGCCCTTTGG 8291

quil AAGAACCGGATGAAATCTCGATTTTGTCAACGCCCTTTGG 8289

meri AAGAACCGGATGAAATCTCGATTTTGTCAACGCCCTTTGG 8414

ref AAGAACCGGATGAAATCTCGATTTcGTCAACGCCCTTTGG 8533

Consensus aagaaccggatgaaatctcgattt gtcaacgccctttgg

kal TTCTTGTTCCGAAAATCGACAACTAAAAGAGAATATTTAT 8329

yor TTCTTGTTCCGAAAATCGACAACTAAAAGAGAATATTTAT 8311

jen TTCTTGTTCCGAAAATCGACAACTAAAAGAGAATATTTAT 8329

cor TTCTTGTTCCGAAAATCGACAACTAAAAGAGAATATTTAT 8329

man TTCTTGTTCCGAAAATCGACAACTAAAAGAGAATATTTAT 8329

uni TTCTTGTTCCGAAAATCGACAACTAAAAGAGAATATTTAT 8329

tall TTCTTGTTCCGAAAATCGACAACTAAAAGAGAATATTTAT 8331

quil TTCTTGTTCCGAAAATCGACAACTAAAAGAGAATATTTAT 8329

meri TTCTTGTTCCGAAAATCGACAACTAAAAGAGAATATTTAT 8454

ref TTCTTGTTCCGAAAgTCGACAACTAAAAGAGAATATTTAT 8573

Consensus ttcttgttccgaaa tcgacaactaaaagagaatatttat

kal AAACTAAAGGAAGTTTCATCTGTAGAATAATCTGTCAACA 8369

yor AAACTAAAGGAAGTTTCATCTGTAGAATAATCTGTCAACA 8351

jen AAACTAAAGGAAGTTTCATCTGTAGAATAATCTGTCAACA 8369

cor AAACTAAAGGAAGTTTCATCTGTAGAATAATCTGTCAACA 8369

man AAACTAAAGGAAGTTTCATCTGTAGAATAATCTGTCAACA 8369

uni AAACTAAAGGAAGTTTCATCTGTAGAATAATCTGTCAACA 8369

tall AAACTAAAGGAAGTTTCATCTGTAGAATAATCTGTCAACA 8371

quil AAACTAAAGGAAGTTTCATCTGTAGAATAATCTGTCAACA 8369

meri AAACTAAAGGAAGTTTCATCTGTAGAATAATCTGTCAACA 8494

ref AAACTAAAGGAAGTTTCATCTGTAGAATAATCTGTCAACA 8613

Consensus aaactaaaggaagtttcatctgtagaataatctgtcaaca

kal TTAATGTTCTAACATCAAGTGATTTTCTTCCTTCTTCAAG 8409

yor TTAATGTTCTAACATCAAGTGATTTTCTTCCTTCTTCAAG 8391

jen TTAATGTTCTAACATCAAGTGATTTTCTTCCTTCTTCAAG 8409

cor TTAATGTTCTAACATCAAGTGATTTTCTTCCTTCTTCAAG 8409

man TTAATGTTCTAACATCAAGTGATTTTCTTCCTTCTTCAAG 8409

uni TTAATGTTCTAACATCAAGTGATTTTCTTCCTTCTTCAAG 8409

tall TTAATGTTCTAACATCAAGTGATTTTCTTCCTTCTTCAAG 8411

quil TTAATGTTCTAACATCAAGTGATTTTCTTCCTTCTTCAAG 8409

meri TTAATGTTCTAACATCAAGTGATTTTCTTCCTTCTTCAAG 8534

ref TTAATGTTCTAACATCAAGTGATTTTCTTCCTTCTTCAAG 8653

Consensus ttaatgttctaacatcaagtgattttcttccttcttcaag

kal AAACATTAGCATAAAAAATATCATGTGAATGAAGATGAAT 8449

yor AAACATTAGCATAAAAAATATCATGTGAATGAAGATGAAT 8431

jen AAACATTAGCATAAAAAATATCATGTGAATGAAGATGAAT 8449

cor AAACATTAGCATAAAAAATATCATGTGAATGAAGATGAAT 8449

man AAACATTAGCATAAAAAATATCATGTGAATGAAGATGAAT 8449

uni AAACATTAGCATAAAAAATATCATGTGAATGAAGATGAAT 8449

tall AAACATTAGCATAAAAAATATCATGTGAATGAAGATGAAT 8451

quil AAACATTAGCATAAAAAATATCATGTGAATGAAGATGAAT 8449

meri AAACATTAGCATAAAAAATATCATGTGAATGAAGATGAAT 8574

ref AAACATTAGCATAAAAAATATCATGTGAATGAAGATGAAT 8693

Consensus aaacattagcataaaaaatatcatgtgaatgaagatgaat

kal GGTATGAAATGTTTGAAGCCTATCAAGCATAACATTAAGG 8489

yor GGTATGAAATGTTTGAAGCCTATCAAGCATAACATTAAGG 8471

jen GGTATGAAATGTTTGAAGCCTATCAAGCATAACATTAAGG 8489

cor GGTATGAAATGTTTGAAGCCTATCAAGCATAACATTAAGG 8489

man GGTATGAAATGTTTGAAGCCTATCAAGCATAACATTAAGG 8489

uni GGTATGAAATGTTTGAAGCCTATCAAGCATAACATTAAGG 8489

tall GGTATGAAATGTTTGAAGCCTATCAAGCATAACATTAAGG 8491

quil GGTATGAAATGTTTGAAGCCTATCAAGCATAACATTAAGG 8489

meri GGTATGAAATGTTTGAAGCCTATCAAGCATAACATTAAGG 8614

ref GGTATGAAATaTTTGAAaCCTATCAAGCATAACATTAAGG 8733

Consensus ggtatgaaat tttgaa cctatcaagcataacattaagg

kal GAAATCATTTGAATCAAGACTTAGTAATGTGCAACAAGAG 8529

yor GAAATCATTTGAATCAAGACTTAGTAATGTGCAACAAGAG 8511

jen GAAATCATTTGAATCAAGACTTAGTAATGTGCAACAAGAG 8529

cor GAAATCATTTGAATCAAGACTTAGTAATGTGCAACAAGAG 8529

man GAAATCATTTGAATCAAGACTTAGTAATGTGCAACAAGAG 8529

uni GAAATCATTTGAATCAAGACTTAGTAATGTGCAACAAGAG 8529

tall GAAATCATTTGAATCAAGACTTAGTAATGTGCAACAAGAG 8531

quil GAAATCATTTGAATCAAGACTTAGTAATGTGCAACAAGAG 8529

meri GAAATCATTTGAATCAAGACTTAGTAATGTGCAACAAGAG 8654

ref GAAATCATTTGAATCAAGACTTAGTAATGTGCAACAAGAG 8773

Consensus gaaatcatttgaatcaagacttagtaatgtgcaacaagag

kal ACCAAGGCGAACCTGTAGTGTCAGAGCACTAGTATGTTTA 8569

yor ACaAAGGCGAACCTGTAGTGTCAGAGCACTAGTATGTTTA 8551

jen ACCAAGGCGAACCTGTAGTGTCAGAGCACTAGTATGTTTA 8569

cor ACCAAGGCGAACCTGTAGTGTCAGAGCACTAGTATGTTTA 8569

man ACCAAGGCGAACCTGTAGTGTCAGAGCACTAGTATGTTTA 8569

uni ACCAAGGCGAACCTGTAGTGTCAGAGCACTAGTATGTTTA 8569

tall ACCAAGGCGAACCTGgAGTGTgAGAGCACTtGTATGTTTA 8571

quil ACCAAGGCGAACCTGTAGTGTCAGAGCACTAGTATGTTTA 8569

meri ACaAAGGCGAACCTGTAGTGTCAGAGCACTAGTATGTTTA 8694

ref ACaAAGGCGAACCTGTAGTGTCAGAGCgCTAGTATGTTTA 8813

Consensus ac aaggcgaacctg agtgt agagc ct gtatgttta

kal GTGACCAAGAAGTTTGTTAAGTTTGCTGCATATGCTGTGA 8609

yor GTGACCAAGAAGTTTGTTAAGTTTGCTGCATATGCTGTGA 8591

jen GTGACCAAGAAGTTTGTTAAGTTTGCTGCATATGCTGTGA 8609

cor GTGACCAAGAAGTTTGTTAAGTTTGCTGCATATGCTGTGA 8609

man GTGACCAAGAAGTTTGTTAAGTTTGCTGCATATGCTGTGA 8609

uni GTGACCAAGAAGTTTGTTAAGTTTGCTGCATATGCTGTGA 8609

tall GTcACCAAGAAGTTTGTTAAGTTTGCTGCATATGCTGTaA 8611

quil GTGACCAAGAAGTTTGTTAAGTTTGCTGCATATGCTGTGA 8609

meri GTGACCAAGAAGTTTGTTAAGTTTGCTGCATATGCTGTGA 8734

ref GTGACCAAGAAGTTTGTTAAGTTTGCTGCATATGCTGTGA 8853

Consensus gt accaagaagtttgttaagtttgctgcatatgctgt a

kal CTGAGTTAAGCAAAAGTAGTAGCCACATAAATTTATTTTC 8649

yor CTGAGTTAAGCAAAAGTAGTAGCCACATAAATTTATTTTC 8631

jen CTGAGTTAAGCAAAAGTAGTAGCCACATAAATTTATTTTC 8649

cor CTGAGTTAAGCAAAAGTAGTAGCCACATAAATTTATTTTC 8649

man CTGAGTTAAGCAAAAGTAGTAGCCACATAAATTTATTTTC 8649

uni CTGAGTTAAGCAAAAGTAGTAGCCACATAAATTTATTTTC 8649

tall CTGAGTTAAGCAAAAGTAGTAGCCACATAAATTTATTTTC 8651

quil CTGAGTTAAGCAAAAGTAGTAGCCACATAAATTTATTTTC 8649

meri CTGAGTTAAGCAAAAGTAGTAGCCACATAAATTTATTTTC 8774

ref CTGAGTTAAGCAAAAGTAGTAGCCACATAAATTTATTTTC 8893

Consensus ctgagttaagcaaaagtagtagccacataaatttattttc

kal CCTTCCAAGCTCTAATGTGACATCTACAACATTAGGCTCC 8689

yor CCTTCCAAGCTCTAATGTGACATCTACAACATTAGGCTCC 8671

jen CCTTCCAAGCTCTAATGTGACATCTACAACATTAGGCTCC 8689

cor CCTTCCAAGCTCTAATGTGACATCTACAACATTAGGCTCC 8689

man CCTTCCAAGCTCTAATGTGACATCTACAACATTAGGCTCC 8689

uni CCTTCCAAGCTCTAATGTGACATCTACAACATTAGGCTCC 8689

tall CCTTCCAAGCTCTAATGTGACATCTACAACATTAGGCTCC 8691

quil CCTTCCAAGCTCTAATGTGACATCTACAACATTAGGCTCC 8689

meri CCTTCCAAGCTCTAATGTGACATCTACAACATTAGGCTCC 8814

ref CCTTCCAAGCTCTAATGTGACATCTACAACATTAGGCTCC 8933

Consensus ccttccaagctctaatgtgacatctacaacattaggctcc

kal ATGATAAGTGCGGCCGGCAACAAAAATATGACGGCGATTG 8729

yor ATGATAAGTGCGGCCGGCAACAAAAATATGACGGCGATTG 8711

jen ATGATAAGTGCGGCCGGCAACAAAAATATGACGGCGATTG 8729

cor ATGATAAGTGCaGtaGGCAACAAAAATATGACcGCaATTG 8729

man ATGATAAGTGCGGCCGGCAACAAAAATATGACGGCGATTG 8729

uni ATGATAAGTGCGGCCGGCAACAAAAATATGACGGCGATTG 8729

tall ATGATAAGTGCGGCCGGCAACAAAAATATGACGGCGATTG 8731

quil ATGATAAGTGCGGCCGGCAACAAAAATATGACGGCGATTG 8729

meri ATGATAAGTGCGGCCGGCAACAAAAATATGACGGCGATTG 8854

ref ATGATAAGTGCGGCCGGCAACAAAAATATGACGGCcATTG 8973

Consensus atgataagtgc g ggcaacaaaaatatgac gc attg

kal GAGACATATACAGGAGCAAATTCATCGAGTTCAACTTTTC 8769

yor GAGACATATACAGGAGCAAATTCATCGAGTTCAACTTTTC 8751

jen GAGACATATACAGGAGCAAATTCATCGAGTTCAACTTTTC 8769

cor GAGACATATACAGaAGCAAATTCATCGAGTTCAACTTTTC 8769

man GAGACATATACAGGAGCAAATTCATCGAGTTCAACTTTTC 8769

uni GAGACATATACAGGAGCAAATTCATCGAGTTCAACTTTTC 8769

tall GAGACATATACAGGAGCAAATTCATCGAGTTCAACTTTTC 8771

quil GAGACATATACAGGAGCAAATTCATCGAGTTCAACTTTTC 8769

meri GAGACATATACAGGAGCAAATTCATCGAGTTCAACTTTTC 8894

ref GAGACATATACAGGAGCAAATTCATCGAGTTCAACTTTTC 9013

Consensus gagacatatacag agcaaattcatcgagttcaacttttc

kal CCTGTTAAGTTGAATTAGAAGCAATGTTATAATATGTTTG 8809

yor CCTGTTAAGTTGAATTAGAAGCAATGTTATAATATGTTTG 8791

jen CCTGTTAAGTTGAATTAGAAGCAATGTTATAATATGTTTG 8809

cor CCTGTTAAGTTGAATTAGAAGCAATGTTATAATATGTTTG 8809

man CCTGTTAAGTTGAATTAGAAGCAATGTTATAATATGTTTG 8809

uni CCTGTTAAGTTGAATTAGAAGCAATGTTATAATATGTTTG 8809

tall CCTGTTAAGTTGAATTAGAAGCAATGTTATAATATGTTTG 8811

quil CCTGTTAAGTTGAATTAGAAGCAATGTTATAATATGTTTG 8809

meri CCTGTTAAGTTGAATTAGAAGCAATGTTATAATATGTTTG 8934

ref CCTGTTAAGTTGAATTAGAAGCAATGTTATAATATGTTTG 9053

Consensus cctgttaagttgaattagaagcaatgttataatatgtttg

kal TTGCCACTAAAAAAAGATTATCTGATTTTGGCATAATACT 8849

yor TTGCCACTAAAAAAAGATTATCTGATTTTGGCATAATACT 8831

jen TTGCCACTAAAAAAAGATTATCTGATTTTGGCATAATACT 8849

cor TTGCCACTAAAAAAAGATTATCTGATTTTGGCATAATACT 8849

man TTGCCACTAAAAAAAGATTATCTGATTTTGGCATAATACT 8849

uni TTGCCACTAAAAAAAGATTATCTGATTTTGGCATAATACT 8849

tall TTGCCACTAAAAAAAGATTATCTGATTTTGGCATAATACT 8851

quil TTGCCACTAAAAAAAGATTATCTGATTTTGGCATAATACT 8849

meri TTGCCACTAAAAAAAGATTATCTGATTTTGGCATAATACT 8974

ref TTGCCACTAAAAAAAGATTATCTGATTTTGGCATAATACT 9093

Consensus ttgccactaaaaaaagattatctgattttggcataatact

kal AGTCCAAGACATTAAGGGGAGTGAGGATTCCCAAGTTGTG 8889

yor AGTCCAAGACATTAAGGGGAGTGAGGATTCCCAAGTTGTG 8871

jen AGTCCAAGACATTAAGGGGAGTGAGGATTCCCAAGTTGTG 8889

cor AGTCCAAGACATTAAGGGGAGTGAGGATTCCCAAGTTGTG 8889

man AGTCCAAGACATTAAGGGGAGTGAGGATTCCCAAGTTGTG 8889

uni AGTCCAAGACATTAAGGGGAGTGAGGATTCCCAAGTTGTG 8889

tall AGTCCAAGACATTAAGGGGAGTGAGGATTCCCAAGTTGTG 8891

quil AGTCCAAGACATTAAGGGGAGTGAGGATTCCCAAGTTGTG 8889

meri AGTCCAAGACATTAAGGGGAGTGAGGATTCCCAAGTTGTG 9014

ref AGTCCAAGACATTAAGGGGAGTGAGGATTCCCAAGcTGTG 9133

Consensus agtccaagacattaaggggagtgaggattcccaag tgtg

kal ATCATAGTTAGACAATAAGATAACTCGAAGGAACATAAAA 8929

yor ATCATAGTTAGACAATAAGATAACTCGAAGGAACATAAAA 8911

jen ATCATAGTTAGACAATAAGATAACTCGAAGGAACATAAAA 8929

cor ATCATAGTTAGACAATAAGATAACTCGAAGGAACATAAAA 8929

man ATCATAGTTAGACAATAAGATAACTCGAAGGAACATAAAA 8929

uni ATCATAGTTAGACAATAAGATAACTCGAAGGAACATAAAA 8929

tall ATCATAGTTAGACAATAAGATAACTCGAAGGAACATAAAA 8931

quil ATCATAGTTAGACAATAAGATAACTCGAAGGAACATAAAA 8929

meri ATCATAGTTAGACAATAAGATAACTCGAAGGAACATAAAA 9054

ref ATCATAGTTAGACAATAAGATAACTCGAAGGAACATAAAA 9173

Consensus atcatagttagacaataagataactcgaaggaacataaaa

kal ATATCCGATATTGCGAAGAAAATAAGAAAATGTTAAAGGA 8969

yor ATATCCGATATTGCGAAGAAAATAAGAAAATGTTAAAGGA 8951

jen ATATCCGATATTGCGAAGAAAATAAGAAAATGTTAAAGGA 8969

cor ATATCCGATATTGCGAAGAAAATAAGAAAATGTTAAAGGA 8969

man ATATCCGATATTGCGAAGAAAATAAGAAAATGTTAAAGGA 8969

uni ATATCCGATATTGCGAAGAAAATAAGAAAATGTTAAAGGA 8969

tall ATATCCGATATTGCGAAGAAAATAAGAAAATGTTAAAGGA 8971

quil ATATCCGATATTGCGAAGAAAATAAGAAAATGTTAAAGGA 8969

meri ATATCCGATATTGCGAAGAAAATAAGAAAATGTTAAAGGA 9094

ref ATATCCGATATTGCGAAGAAAATAAGAAAATGTTcAAGGA 9213

Consensus atatccgatattgcgaagaaaataagaaaatgtt aagga

kal ACATTGTGATTCAGTCACCTTGCCGCTTATTGCAACAAAC 9009

yor ACATTGTGATTCAGTCACCTTGCCGCTTATTGCAACAAAC 8991

jen ACATTGTGATTCAGTCACCTTGCCGCTTATTGCAACAAAC 9009

cor ACATTGTGATTCAGTCACCTTGCCGCTTATTGCAACAAAC 9009

man ACATTGTGATTCAGTCACCTTGCCGCTTATTGCAACAAAC 9009

uni ACATTGTGATTCAGTCACCTTGCCGCTTATTGCAACAAAC 9009

tall ACATTGTGATTCAGTCACCTTGCCGCTTATTGCAACAAAC 9011

quil ACATTGTGATTCAGTCACCTTGCCGCTTATTGCAACAAAC 9009

meri ACATTGTGATTCAGTCACCTTGCCGCTTATTGCAACAAAC 9134

ref ACATTGTGATTCAGTCACCTTGtCGCTTATTGCAACAAAC 9253

Consensus acattgtgattcagtcaccttg cgcttattgcaacaaac

kal CTATCATATGAAATGAATCTATGAAAGAAACTCTGTAAAT 9049

yor CTATCATATGAAATGAATCTATGAAAGAAACTCTGTAAAT 9031

jen CTATCATATGAAATGAATCTATGAAAGAAACTCTGTAAAT 9049

cor CTATCATATGAAATGAATCTATGAAAGAAACTCTGTAAAT 9049

man CTATCATATGAAATGAATCTATGAAAGAAACTCTGTAAAT 9049

uni CTATCATATGAAATGAATCTATGAAAGAAACTCTGTAAAT 9049

tall CTATCATATGAAATGAATCTATGAAAGAAACTCTGTAAAT 9051

quil CTATCATATGAAATGAATCTATGAAAGAAACTCTGTAAAT 9049

meri CTATCATATGAAATGAATCTATGAAAGAAACTCTGTAAAT 9174

ref CTATCATATGAAATGAATCTATGAAAGAAACTCTGTAAAT 9293

Consensus ctatcatatgaaatgaatctatgaaagaaactctgtaaat

kal CTCTCTAGATAAAGAATCGACAATTCTCAACCACGATCTT 9089

yor CTCTCTAGATAAAGAATCGACAATTCTCAACCACGATCTT 9071

jen CTCTCTAGATAAAGAATCGACAATTCTCAACCACGATCTT 9089

cor CTCTCTAGATAAAGAATCGACAATTCTCAACCACGATCTT 9089

man CTCTCTAGATAAAGAATCGACAATTCTCAACCACGATCTT 9089

uni CTCTCTAGATAAAGAATCGACAATTCTCAACCACGATCTT 9089

tall CTCTCTAGATAAAGAATCGACAATTCTCAACCACGATCTT 9091

quil CTCTCTAGATAAAGAATCGACAATTCTCAACCACGATCTT 9089

meri CTCTCTAGATAAAGAATCGACAATTCTCAACCACGATCTT 9214

ref CTCTCTAGATAAAGAATCGACAATTCTCAACCACGATCTT 9333

Consensus ctctctagataaagaatcgacaattctcaaccacgatctt

kal CTATCTATGCAAGCTAATATATGCAGATTATAGCCTGGTG 9129

yor CTATCTATGCAAGCTAATATATGCAGATTATAGCCTGGTG 9111

jen CTATCTATGCAAGCTAATATATGCAGATTATAGCCTGGTG 9129

cor CTATCTATGCAAGCTAATATATGCAGATTATAGCCTGGTG 9129

man CTATCTATGCAAGCTAATATATGCAGATTATAGCCTGGTG 9129

uni CTATCTATGCAAGCTAATATATGCAGATTATAGCCTGGTG 9129

tall CTATCTATGCAAGCTAATATATGCAGATTATAGCCTGGTG 9131

quil CTATCTATGCAAGCTAATATATGCAGATTATAGCCTGGTG 9129

meri CTATCTATGCAAGCTAATATATGCAGATTATAGCCTGGTG 9254

ref CTATCTATGCAAGCTAATATATGCAGATTATAGCCTGGTG 9373

Consensus ctatctatgcaagctaatatatgcagattatagcctggtg

kal AAAGTTTCTTAGTTGCATAGTTGCAAACACCTTGTTACTT 9169

yor AAAGTTTCTTAGTTGCATAGTTGCAAAtACCTTGTTACTT 9151

jen AAAGTTTCTTAGTTGCATAGTTGCAAACACCTTGTTACTT 9169

cor AAAGTTTCTTAGTTGCATAGTTGCAAACACCTTGTTACTT 9169

man AAAGTTTCTTAGTTGCATAGTTGCAAACACCTTGTTACTT 9169

uni AAAGTTTCTTAGTTGCATAGTTGCAAACACCTTGTTACTT 9169

tall AAAGTTTCTTAGTTGCATAGTTGCAAACACCTTGTTACTT 9171

quil AAAGTTTCTTAGTTGCATAGTTGCAAACACCTTGTTACTT 9169

meri AAAGTTTCTTAGTTGCATAGTTGCAAAtACCTTGTTACTT 9294

ref AAAGTTTCTTAGTTGCATAGTTGCAAACACCTTGTTACTT 9413

Consensus aaagtttcttagttgcatagttgcaaa accttgttactt

kal ATGAATGTTTTGACCGATAACAACACCATTAAGACTTTCC 9209

yor ATGAATGTTTTGACCGATAACAACACCATTAAGACTTTCC 9191

jen ATGAATGTTTTGACCGATAACAACACCATTAAGACTTTCC 9209

cor ATGAATGTTTTGACCGATAACAACACCATTAAGACTTTCC 9209

man ATGAATGTTTTGACCGATAACAACACCATTAAGACTTTCC 9209

uni ATGAATGTTTTGACCGATAACAACACCATTAAGACTTTCC 9209

tall ATGAATGTTTTGACCGATAACAACACCATTAAGACTTTCC 9211

quil ATGAATGTTTTGACCGATAACAACACCATTAAGACTTTCC 9209

meri ATGAATGTTTTGACCGATAACAACACCATTAAGACTTTCC 9334

ref ATGAATGTTTTGACCGATAACAACACCATTAAGACTTTCC 9453

Consensus atgaatgttttgaccgataacaacaccattaagactttcc

kal ATGAAGAAATCTGTCTATTGCATACCCCATGAGTACTTTT 9249

yor ATGAAcAAATCTGTCTATTGCATACCCCATGAGTACTTTT 9231

jen ATGAAGAAATCTGTCTATTGCATACCCCATGAGTACTTTT 9249

cor ATGAAGAAATCTGTCTATTGCATACCCCATGAGTACTTTT 9249

man ATGAAGAAATCTGTCTATTGCATACCCCATGAGTACTTTT 9249

uni ATGAAGAAATCTGTCTATTGCATACCCCATGAGTACTTTT 9249

tall ATGAAGAAATCTGTCTATTGCATACCCCATGAGTACTTTT 9251

quil ATGAAGAAATCTGTCTATTGCATACCCCATGAGTACTTTT 9249

meri ATGAAcAAATCTGTCTATTGCATACCCCATGAGTACTTTT 9374

ref ATGAAGAAATCTGTCTATTGCATACCCCATGAGTACTTTT 9493

Consensus atgaa aaatctgtctattgcataccccatgagtactttt

kal AGATGGCTAGTTCCCCATCAATGCACAAGATTTAGTTTCA 9289

yor AGATGGCTAGTTCCCCATCAATGCACAAGATTTAGTTTCA 9271

jen AGATGGCTAGTTCCCCATCAATGCACAAGATTTAGTTTCA 9289

cor AGATGGCTAGTTCCCCATCAATGCACAAGATTTAGTTTCA 9289

man AGATGGCTAGTTCCCCATCAATGCACAAGATTTAGTTTCA 9289

uni AGATGGCTAGTTCCCCATCAATGCACAAGATTTAGTTTCA 9289

tall AGATGGCTAGTTCCCCATCAATGCACAAGATTTAGTTTCA 9291

quil AGATGGCTAGTTCCCCATCAATGCACAAGATTTAGTTTCA 9289

meri AGATGGCTAGTTCCCCATCAATGCACAAGATTTAGTTTCA 9414

ref AGATGGCTAGTTCCCCATCAATGCACAAGATTTAGTTTCA 9533

Consensus agatggctagttccccatcaatgcacaagatttagtttca

kal AATAAACACTAATATAACTAAAAATGTGACAGGATTCTTA 9329

yor AATAAgCACTAATATAACTAAAAATGTGACgGGATTCTTA 9311

jen AATAAACACTAATATAACTAAAAATGTGACAGGATTCTTA 9329

cor AATAAACACTAATATAACTAAAAATGTGACAGGATTCTTA 9329

man AATAAACACTAATATAACTAAAAATGTGACAGGATTCTTA 9329

uni AATAAACACTAATATAACTAAAAATGTGACAGGATTCTTA 9329

tall AATAAACACTAATATAACTAAAAATGTGACAGGATTCTTA 9331

quil AATAAACACTAATATAACTAAAAATGTGACAGGATTCTTA 9329

meri AATAAgCACTAATATAACTAAAAATGTGACgGGATTCTTA 9454

ref AATAAACACTAATATAACTAAAAATGTGACAGGATTCTTA 9573

Consensus aataa cactaatataactaaaaatgtgac ggattctta

kal TTGGATATCATTTTTCGGCGATCTTAAAATTTAGCTGGAT 9369

yor TTGGATATCATTTTTCGGCGATCTTAAAATTTAGCTGGAT 9351

jen TTGGATATCATTTTTCGGCGATCTTAAAATTTAGCTGGAT 9369

cor TTGGATATCATTTTTCGGCGATCTTAAAATTTAGCTGGAT 9369

man TTGGATATCATTTTTCGGCGATCTTAAAATTTAGCTGGAT 9369

uni TTGGATATCATTTTTCGGCGATCTTAAAATTTAGCTGGAT 9369

tall TTGGATATCATTTTTCGGCGATCTTAAAATTTAGCTGGAT 9371

quil TTGGATATCATTTTTCGGCGATCTTAAAATTTAGCTGGAT 9369

meri TTGGATATCATTTTTCGGCGATCTTAAAATTTAGCTGGAT 9494

ref TTGGATATCATTTTTCGGCGATCTTAAAATTTAGCTGGAT 9613

Consensus ttggatatcatttttcggcgatcttaaaatttagctggat

kal AATTTTTGAAGCGAGATAACAAAGAGGAAGTTAGAAATCA 9409

yor AATTTTTGAAGCGAGATAACAAAGAGGAAGTTAGAAATCA 9391

jen AATTTTTGAAGCGAGATAACAAAGAGGAAGTTAGAAATCA 9409

cor AATTTTTGAAGCGAGATAACAAAGAGGAAGTTAGAAATCA 9409

man AATTTTTGAAGCGAGATAACAAAGAGGAAGTTAGAAATCA 9409

uni AATTTTTGAAGCGAGATAACAAAGAGGAAGTTAGAAATCA 9409

tall AATTTTTGAAGCGAGATAACAAAGAGGAAGTTAGAAATCA 9411

quil AATTTTTGAAGCGAGATAACAAAGAGGAAGTTAGAAATCA 9409

meri AATTTTTGAAGCGAGATAACAAAGAGGAAGTTAGAAATCA 9534

ref AATTTTTGAAGCGAGATAACAAAGAGGAAGTTAGAAATCA 9653

Consensus aatttttgaagcgagataacaaagaggaagttagaaatca

kal TAGATATAGTGTAAAATATTTTACCCTTCAGATGAAAGCA 9449

yor TAGATATAGTGTAAAATATTTTACCCTTCAGATGAAAGCA 9431

jen TAGATATAGTGTAAAATATTTTACCCTTCAGATGAAAGCA 9449

cor TAGATATAGTGTAAAATATTTTACCCTTCAGATGAAAGCA 9449

man TAGATATAGTGTAAAATATTTTACCCTTCAGATGAAAGCA 9449

uni TAGATATAGTGTAAAATATTTTACCCTTCAGATGAAAGCA 9449

tall TAGATATAGTGTAAAATATTTTACCCTTCAGATGAAAGCA 9451

quil TAGATATAGTGTAAAATATTTTACCCTTCAGATGAAAGCA 9449

meri TAGATATAGTGTAAAATATTTTACCCTTCAGATGAAAGCA 9574

ref TAGATATAGTGTAAAATATTTTACCCTTCAGATGAAAGCA 9693

Consensus tagatatagtgtaaaatattttacccttcagatgaaagca

kal AAATGCCCTGAAGAACAGACTTGAAAGCTCTCGCAGCGGT 9489

yor AAATGCCCTGAAGAACAGACTTGAAAGCTCTCGCAGCGGT 9471

jen AAATGCCCTGAAGAACAGACTTGAAAGCTCTCGCAGCGGT 9489

cor AAATGCCCTGAAGAACAGACTTGAAAGCTCTCGCAGCGGT 9489

man AAATGCCCTGAAGAACAGACTTGAAAGCTCTCGCAGCGGT 9489

uni AAATGCCCTGAAGAACAGACTTGAAAGCTCTCGCAGCGGT 9489

tall AAATGCCCTGAAGAACAGACTTGAAAGCTCTCGCAGCGGT 9491

quil AAATGCCCTGAAGAACAGACTTGAAAGCTCTCGCAGCGGT 9489

meri AAATGCCCTGAAGAACAGACTTGAAAGCTCTCGCAGCGGT 9614

ref AAATGCCCTGAAGAACAGACTTGAAAGCTCTCGCAGCGGT 9733

Consensus aaatgccctgaagaacagacttgaaagctctcgcagcggt

kal TGCACTTAGGCACATAAGGAATCCAAATAAGTGAAAATCT 9529

yor TGCACTTAGGCACATAAGGAATCCAAATAAGTGAAAATCT 9511

jen TGCACTTAGGCACATAAGGAATCCAAATAAGTGAAAATCT 9529

cor TGCACTTAGGCACATAAGGAATCCAAATAAGTGAAAATCT 9529

man TGCACTTAGGCACATAAGGAATCCAAATAAGTGAAAATCT 9529

uni TGCACTTAGGCACATAAGGAATCCAAATAAGTGAAAATCT 9529

tall TGCACTTAGGCACATAAGGAATCCAAATAAGTGAAAATCT 9531

quil TGCACTTAGGCACATAAGGAATCCAAATAAGTGAAAATCT 9529

meri TGCACTTAGGCACATAAGGAATCCAAATAAGTGAAAATCT 9654

ref TGCACTTAGGCACATAAGGAATCCAAATAAGTGAAAATCT 9773

Consensus tgcacttaggcacataaggaatccaaataagtgaaaatct

kal GGTTCACCCTTCAAGAACAAAATATTGTAACTGAACAGAA 9569

yor GGTTCACCCTTCAAGAACAAAATATTGTAACTGAACAGAA 9551

jen GGTTCACCCTTCAAGAACAAAATATTGTAACTGAACAGAA 9569

cor GGTTCACCCTTCAAGAACAAAATATTGTAACTGAACAGAA 9569

man GGTTCACCCTTCAAGAACAAAATATTGTAACTGAACAGAA 9569

uni GGTTCACCCTTCAAGAACAAAATATTGTAACTGAACAGAA 9569

tall GGTTCACCCTTCAAGAACAAAATATTGTAACTGAACAGAA 9571

quil GGTTCACCCTTCAAGAACAAAATATTGTAACTGAACAGAA 9569

meri GGTTCACCCTTCAAGAACAAAATATTGTAACTGAACAGAA 9694

ref GGTTCACCCTTCAAGAACAAAATATTGTAACTGAACAGAA 9813

Consensus ggttcacccttcaagaacaaaatattgtaactgaacagaa

kal GAAAACATAAAAAAA.GTATGGAAATTGGAAGCAGATATG 9608

yor GAAAACATAAAAAAAaGTATGGAAATTGGAAGCAGATATG 9591

jen GAAAACATAAAAAAA.GTATGGAAATTGGAAGCAGATATG 9608

cor GAAAACATAAAAAAA.GTATGGAAATTGGAAGCAGATATG 9608

man GAAAACATAAAAAAA.GTATGGAAATTGGAAGCAGATATG 9608

uni GAAAACATAAAAAAA.GTATGGAAATTGGAAGCAGATATG 9608

tall GAAAACATAAAAAAA.GTATGGAAATTGGAAGCAGATATG 9610

quil GAAAACATAAAAAAA.GTATGGAAATTGGAAGCAGATATG 9608

meri GAAAACATAAAAAAAaGTATGGAAATTGGAAGCAGATATG 9734

ref GAAAACATAAAAAAAaGTATGGAAATTGGAAGCAGATATG 9853

Consensus gaaaacataaaaaaa gtatggaaattggaagcagatatg

kal CACCATGCACATCAAAAGGCACTACTATTCCTTGCCAGAC 9648

yor CACCATGCACATCAAAAGGCACTACTATTCCTTGCCAGAC 9631

jen CACCATGCACATCAAAAGGCACTACTATTCCTTGCCAGAC 9648

cor CACCATGCACATCAAAAGGCACTACTATTCCTTGCCAGAC 9648

man CACCATGCACATCAAAAGGCACTACTATTCCTTGCCAGAC 9648

uni CACCATGCACATCAAAAGGCACTACTATTCCTTGCCAGAC 9648

tall CACCATGCACATCAAAAGGCACTACTATTCCTTGCCAGAC 9650

quil CACCATGCACATCAAAAGGCACTACTATTCCTTGCCAGAC 9648

meri CACCATGCACATCAAAAGGCACTACTATTCCTTGCCAGAC 9774

ref CACCATGCACATCAAAAGGCACTACTATTCCTTGCCAGAC 9893

Consensus caccatgcacatcaaaaggcactactattccttgccagac

kal ATATAATCGATTATTAAATTAGGCCATCTGATATAACAAC 9688

yor ATATAATCGATTATTAAATTAGGCCATCTaATATAACAAC 9671

jen ATATAATCGATTATTAAATTAGGCCATCTGATATAACAAC 9688

cor ATATAATCGATTATTAAATTAGGCCATCTGATATAACAAC 9688

man ATATAATCGATTATTAAATTAGGCCATCTGATATAACAAC 9688

uni ATATAATCGATTATTAAATTAGGCCATCTGATATAACAAC 9688

tall ATATAATCGATTATTAAATTAGGCCATCTGATATAACAAC 9690

quil ATATAATCGATTATTAAATTAGGCCATCTGATATAACAAC 9688

meri ATATAATCGATTATTAAAgTAGGCCATCTaATATAACAAC 9814

ref ATATAATCGATTATTAAATTAGGCCATCTaATATAACAAC 9933

Consensus atataatcgattattaaa taggccatct atataacaac

kal AATAACCAAGCCTTTATCCCGTGGAGTTGGCTACATGGAT 9728

yor AATAACCAAGCCTTTATCCCGTGGgaTTGGCTACATGGAT 9711

jen AATAACCAAGCCTTTATCCCGTGGAGTTGGCTACATGGAT 9728

cor AATAACCAAGCCTTTATCCCGTGGAGTTGGCTACATGGAT 9728

man AATAACCAAGCCTTTATCCCGTGGAGTTGGCTACATGGAT 9728

uni AATAACCAAGCCTTTATCCCGTGGAGTTGGCTACATGGAT 9728

tall AATAACCAAGCCTTTATCCCGTGGAGTTGGCTACATGGAT 9730

quil AATAACCAAGCCTTTATCCCGTGGAGTTGGCTACATGGAT 9728

meri AATAACCAAGCCTTTATCCCGTGGgGTTGGCTACATGGAT 9854

ref AATAACCAAGCCTTTATCCCGTGGgGTTGGCTACATGGAT 9973

Consensus aataaccaagcctttatcccgtgg ttggctacatggat

kal TGAACAATGTCATAATGTTCTATCATAAATAAGAAAATTA 9768

yor TGAACAATGcCATAATGTTCTATCATAAATAAGAAAATTA 9751

jen TGAACAATGTCATAATGTTCTATCATAAATAAGAAAATTA 9768

cor TGAACAATGTCATAATGTTCTATCATAAATAAGAAAATTA 9768

man TGAACAATGTCATAATGTTCTATCATAAATAAGAAAATTA 9768

uni TGAACAATGTCATAATGTTCTATCATAAATAAGAAAATTA 9768

tall TGAACAATGTCATAATGTTCTATCATAAATAAGAAAATTA 9770

quil TGAACAATGTCATAATGTTCTATCATAAATAAGAAAATTA 9768

meri TGAACAATGTCATAATGTTCTATCATAAATAAGAAAATTA 9894

ref TGAACAATGcCATAATGTTCTATCATAAATAAGAAAATTA 10013

Consensus tgaacaatg cataatgttctatcataaataagaaaatta

kal GAAAAAAAACGACACCATCTGATATATGAAAATAAATTAG 9808

yor GAAAAgAAACGACACCATCTGATATATGAAAATAAATTAG 9791

jen GAAAAAAAACGACACCATCTGATATATGAAAATAAATTAG 9808

cor GAAAAAAAACGACACCATCTGATATATGAAAATAAATTAG 9808

man GAAAAAAAACGACACCATCTGATATATGAAAATAAATTAG 9808

uni GAAAAAAAACGACACCATCTGATATATGAAAATAAATTAG 9808

tall GAAAAAAAACGACACCATCTGATATATGAAAATAAATTAG 9810

quil GAAAAAAAACGACACCATCTGATATATGAAAATAAATTAG 9808

meri GAAAAAAAACGACACCATCTGATATATGAAAATAAATTAG 9934

ref GAAAAgAAACGACACCATCTGATATATGAAAATAAATcAG 10053

Consensus gaaaa aaacgacaccatctgatatatgaaaataaat ag

kal AAAAACACAGATTTCCGGTTCTTAAAAGATTGAGATCACA 9848

yor AAAAACACAGATTTCCGGTTCTTAAAAGATTtAGATCACA 9831

jen AAAAACACAGATTTCCGGTTCTTAAAAGATTGAGATCACA 9848

cor AAAAACACAGATTTCCGGTTCTTAAAAGATTGAGATCACA 9848

man AAAAACACAGATTTCCGGTTCTTAAAAGATTGAGATCACA 9848

uni AAAAACACAGATTTCCGGTTCTTAAAAGATTGAGATCACA 9848

tall AAAAACACAGATTTCCGGTTCTTAAAAGATTGAGATCACA 9850

quil AAAAACACAGATTTCCGGTTCTTAAAAGATTGAGATCACA 9848

meri AAAAACACAGATTTCCGGTTCTTAAAAGATTGAGATCACA 9974

ref AAAAACACAGATTTCCGGTTCTTAAAAGATTGAGATCACA 10093

Consensus aaaaacacagatttccggttcttaaaagatt agatcaca

kal TTAACTTTCAAGCAAGTTGCATTTCTTAAATAAGGTCATC 9888

yor TTAACTTTCAAGCAAGTTGCATTTgTTAAATAAGGTCATC 9871

jen TTAACTTTCAAGCAAGTTGCATTTCTTAAATAAGGTCATC 9888

cor TTAACTTTCAAGCAAGTTGCATTTCTTAAATAAGGTCATC 9888

man TTAACTTTCAAGCAAGTTGCATTTCTTAAATAAGGTCATC 9888

uni TTAACTTTCAAGCAAGTTGCATTTCTTAAATAAGGTCATC 9888

tall TTAACTTTCAAGCAAGTTGCATTTCTTAAATAAGGTCATC 9890

quil TTAACTTTCAAGCAAGTTGCATTTCTTAAATAAGGTCATC 9888

meri TTAACTTTCAAGCAAGTTGCATTTgTTAAATAAGGTCATC 10014

ref TTAACTTTCAAGCAAGTTGCATTTCTTAAATAAGGTCATC 10133

Consensus ttaactttcaagcaagttgcattt ttaaataaggtcatc

kal AGAAATAAAATTAGGCTATCTGGTATATGCAAACAATCTA 9928

yor AGAAATAAAATTAGGCTATCTGGTATATGCAAACAATCTA 9911

jen AGAAATAAAATTAGGCTATCTGGTATATGCAAACAATCTA 9928

cor AGAAATAAAATTAGGCTATCTGGTATATGCAAACAATCTA 9928

man AGAAATAAAATTAGGCTATCTGGTATATGCAAACAATCTA 9928

uni AGAAATAAAATTAGGCTATCTGGTATATGCAAACAATCTA 9928

tall AGAAATAAAATTAGGCTATCTGGTATATGCAAACAATCTA 9930

quil AGAAATAAAATTAGGCTATCTGGTATATGCAAACAATCTA 9928

meri AGAAATAAAATTAGGCTATCTGGTATATGCAAACAATCTA 10054

ref AGAAATAAAATTAGGCTATCTGGTATATGCAAACAATCTA 10173

Consensus agaaataaaattaggctatctggtatatgcaaacaatcta

kal ATCTGTTCAAAGGATT..CCAGATCAGTATTATCATTTGA 9966

yor ATCTGTTCAAAtGATTttCCAGATCAGTATTATCATTTGA 9951

jen ATCTGTTCAAAGGATT..CCAGATCAGTATTATCATTTGA 9966

cor ATCTGTTCAAAGGATT..CCAGATCAGTATTATCATTTGA 9966

man ATCTGTTCAAAGGATT..CCAGATCAGTATTATCATTTGA 9966

uni ATCTGTTCAAAGGATT..CCAGATCAGTATTATCATTTGA 9966

tall ATCTGTTCAAAGGATT..CCAGATCAGTATTATCATTTGA 9968

quil ATCTGTTCAAAGGATT..CCAGATCAGTATTATCATTTGA 9966

meri ATCTGTTCAAAGGATT..CCAGATCAGTATTATCATTTGA 10092

ref ATCTGTTCAAAGGATT..CCAGATCAGTATTATCATTTGA 10211

Consensus atctgttcaaa gatt ccagatcagtattatcatttga

kal AATGTGATGTTGCAATGACATGTTAACCAAAACTATATAT 10006

yor AATGTGATGTTGCAATGACATGTTAACCAAAACTATATAT 9991

jen AATGTGATGTTGCAATGACATGTTAACCAAAACTATATAT 10006

cor AATGTGATGTTGCAATGACATGTTAACCAAAACTATATAT 10006

man AATGTGATGTTGCAATGACATGTTAACCAAAACTATATAT 10006

uni AATGTGATGTTGCAATGACATGTTAACCAAAACTATATAT 10006

tall AATGTGATGTTGCAATGACATGTTAACCAAAACTATATAT 10008

quil AATGTGATGTTGCAATGACATGTTAACCAAAACTATATAT 10006

meri AATGTGATGTTGCAATGACATGTTAACCAAAACTATATAT 10132

ref AATGTGATGTTGCAATGACATGTTAACCAAAACTATATAT 10251

Consensus aatgtgatgttgcaatgacatgttaaccaaaactatatat

kal GTTCATATCATCATAAAAACAGACACAAACGTCATTCAGA 10046

yor GTTCATATCATCATAAAAACAGACACAAAaGTCATTCAGA 10031

jen GTTCATATCATCATAAAAACAGACACAAACGTCATTCAGA 10046

cor GTTCATATCATCATAAAAACAGACACAAACGTCATTCAGA 10046

man GTTCATATCATCATAAAAACAGACACAAACGTCATTCAGA 10046

uni GTTCATATCATCATAAAAACAGACACAAACGTCATTCAGA 10046

tall GTTCATATCATCATAAAAACAGACACAAACGTCATTCAGA 10048

quil GTTCATATCATCATAAAAACAGACACAAACGTCATTCAGA 10046

meri GTTCATATCATCATAAAAACAGACACAAAaGTCATTCAGA 10172

ref GTTCATATgATCATAAAAACAGACACAAACGTCATTCAGA 10291

Consensus gttcatat atcataaaaacagacacaaa gtcattcaga

kal ACCATTCCACAAAAGCATAGTAACACAAAATTATAAATCA 10086

yor ACCATTCCACAAAAGCATAGTAACACAAAATTATAAATCA 10071

jen ACCATTCCACAAAAGCATAGTAACACAAAATTATAAATCA 10086

cor ACCATTCCACAAAAGCATAGTAACACAAAATTATAAATCA 10086

man ACCATTCCACAAAAGCATAGTAACACAAAATTATAAATCA 10086

uni ACCATTCCACAAAAGCATAGTAACACAAAATTATAAATCA 10086

tall ACCATTCCACAAAAGCATAGTAACACAAAATTATAAATCA 10088

quil ACCATTCCACAAAAGCATAGTAACACAAAATTATAAATCA 10086

meri ACCATTCCACAAAAGCATAGTAACACAAAATTATAAATCA 10212

ref ACCATTCCACAAAAGCATAGTAACACAAAATTATAAATCA 10331

Consensus accattccacaaaagcatagtaacacaaaattataaatca

kal AATGAATGCAAAAATTAGATAAATAGATAAACATACCCCA 10126

yor AATGAATGCAAAAATTAGATAAATAGATAAACATACCCCA 10111

jen AATGAATGCAAAAATTAGATAAATAGATAAACATACCCCA 10126

cor AATGAATGCAAAAATTAGATAAATAGATAAACATACCCCA 10126

man AATGAATGCAAAAATTAGATAAATAGATAAACATACCCCA 10126

uni AATGAATGCAAAAATTAGATAAATAGATAAACATACCCCA 10126

tall AATGAATGCAAAAATTAGATAAATAGATAAACATACCCCA 10128

quil AATGAATGCAAAAATTAGATAAATAGATAAACATACCCCA 10126

meri AATGAATGCAAAAATTAGATAAATAGATAAACATACCCCA 10252

ref AATGAATGCAAAAATTAGATAAATAGATAAACATACCCCA 10371

Consensus aatgaatgcaaaaattagataaatagataaacatacccca

kal CTTGCAATGACAACTCCAGTAACAACAGGAACAAGAGCAG 10166

yor CTTGCAATGACAACTCCAGTAACAACAGGAACAAGAGCAG 10151

jen CTTGCAATGACAACTCCAGTAACAACAGGAACAAGAGCAG 10166

cor CTTGCAATGACAACTCCAGTAACAACAGGAACAAGAGCAG 10166

man CTTGCAATGACAACTCCAGTAACAACAGGAACAAGAGCAG 10166

uni CTTGCAATGACAACTCCAGTAACAACAGGAACAAGAGCAG 10166

tall CTTGCAATGACAACTCCnnnnnnnnnnnnnnnnnnnnnnn 10168

quil CTTGCAATGACAACTCCAGTAACAACAGGAACAAGAGCAG 10166

meri CTTGCAATGACAACTCCAGTAACAACAGGAACAAGAGCAG 10292

ref CTTGCAATGACAACTCCAGTAACAACAGGAACAAGAGCAG 10411

Consensus cttgcaatgacaactcc

kal CATATGTGACCCAAGCCTCTCTTTTAAGGGTAGCCAAATA 10206

yor CATATGTGACCCAAGCCTCTCTTTTAAGGGTAGCCAAATA 10191

jen CATATGTGACCCAAGCCTCTCTTTTAAGGGTAGCCAAATA 10206

cor CATATGTGACCCAAGCCTCTCTTTTAAGGGTAGCCAAATA 10206

man CATATGTGACCCAAGCCTCTCTTTTAAGGGTAGCCAAATA 10206

uni CATATGTGACCCAAGCCTCTCTTTTAAGGGTAGCCAAATA 10206

tall nnnnTGTGACCCAAGCCTCTCTTTTAAGGGTAGCCAAATA 10208

quil CATATGTGACCCAAGCCTCTCTTTTAAGGGTAGCCAAATA 10206

meri CATATGTGACCCAAGCCTCTCTTTTAAGGGTAGCCAAATA 10332

ref CATATGTGACCCAAGCCTCTCTTTTAAGGGTAGCCAAATA 10451

Consensus tgtgacccaagcctctcttttaagggtagccaaata

kal AGCAAACACAGCAGTGAAAAATGGTGTAGTTGCACCCACA 10246

yor AGCAAACACAGCAGTGAAAAATGGTGTAGTTGCACCCACA 10231

jen AGCAAACACAGCAGTGAAAAATGGTGTAGTTGCACCCACA 10246

cor AGCAAACACAGCAGTGAAAAATGGTGTAGTTGCACCCACA 10246

man AGCAAACACAGCAGTGAAAAATGGTGTAGTTGCACCCACA 10246

uni AGCAAACACAGCAGTGAAAAATGGTGTAGTTGCACCCACA 10246

tall AGCAAACACAGCAGTGAAAAATGGTGTAGTTGCACCCACA 10248

quil AGCAAACn................................ 10214

meri AGCAAACACAGCAGTGAAAAATGGTGTAGTTGCACCCACA 10372

ref AGCAAACACAGCAGTGAAAAATGGTGTAGTTGCACCCACA 10491

Consensus agcaaac

kal GCTTGATTAAATGACACAGCCAAATACCTAAGTGAAATGT 10286

yor GCTTGATTAAATGACACAGCCAAATACCTAAGTGAAATGT 10271

jen GCTTGATTAAATGACACAGCCAAATACCTAAGTGAAATGT 10286

cor GCTTGATTAAATGACACAGCCAAATACCTAAGTGAAATGT 10286

man GCTTGATTAAATGACACAGCCAAATACCTAAGTGAAATGT 10286

uni GCTTGATTAAATGACACAGCCAAATACCTAAGTGAAATGT 10286

tall GCTTGATTAAATGACACAGCCAAATACCTAAGTGAAATGT 10288

quil ..TTGATTAAATGACACAGCCAAATACCTAAGTGAAATGT 10252

meri GCTTGATTAAATGACACAGCCAAATACCTAAGTGAAATGT 10412

ref GCTTGATTAAATGACACAGCCAAATACCTAAGTGAAATGT 10531

Consensus ttgattaaatgacacagccaaatacctaagtgaaatgt

kal TGCCACCAACCACAGACCCACAAAACACAATGCTCAAAGT 10326

yor TGCCACCAACCACAGACCCACAAAACACAATGCTCAAAGT 10311

jen TGCCACCAACCACAGACCCACAAAACACAATGCTCAAAGT 10326

cor TGCCACCAACCACAGACCCACAAAACACAATGCTCAAAGT 10326

man TGCCACCAACCACAGACCCACAAAACACAATGCTCAAAGT 10326

uni TGCCACCAACCACAGACCCACAAAACACAATGCTCAAAGT 10326

tall TGCCACCAACCACAGACCCACAAAACACAATGCTCAAAGT 10328

quil TGnnnnnnnnnnnnnnnnnnnnnnnnnnnnnnCTCAAAGT 10292

meri TGCCACCAACCACAGACCCACAAAACACAATGCTCAAAGT 10452

ref TGCCACCAACCACAGACCCACAAAACACAATGCTCAAAGT 10571

Consensus tg ctcaaagt

kal TGCAATCTTTATGAACTGAAACCTTGATTTGATAACTTGC 10366

yor TGCAATCTTTATGAACTGAAACCTTGATTTGATAACTTGC 10351

jen TGCAATCTTTATGAACTGAAACCTTGATTTGATAACTTGC 10366

cor TGCAATCTTTATGAACTGAAACCTTGATTTGATAACTTGC 10366

man TGCAATCTTTATGAACTGAAACCTTGATTTGATAACTTGC 10366

uni TGCAATCTTTATGAACTGAAACCTTGATTTGATAACTTGC 10366

tall TGCAATCTTTATGAACTGAAACCTTGATTTGATAACTTGC 10368

quil TGCAATCTTTATGAACTGAAACCTTGATTTGATAACTTGC 10332

meri TGCAATCTTTATGAACTGAAACCTTGATTTGATAACTTGC 10492

ref TGCAATCTTTATGAACTGAAACCTTGATTTGATAACTTGC 10611

Consensus tgcaatctttatgaactgaaaccttgatttgataacttgc

kal CTAGGCACCACCTTAAAGAATGCAATGGAAACATAGCTAA 10406

yor CTAGGCACCACCTTAAAGAATGCAATGGAAACATAGCTAA 10391

jen CTAGGCACCACCTTAAAGAATGCAATGGAAACATAGCTAA 10406

cor CTAGGCACCACCTTAAAGAATGCAATGGAAACATAGCTAA 10406

man CTAGGCACCACCTTAAAGAATGCAATGGAAACATAGCTAA 10406

uni CTAGGCACCACCTTAAAGAATGCAATGGAAACATAGCTAA 10406

tall CTAGGCACCACCTTAAAGAATGCAATGGAAACATAGCTAA 10408

quil CTAGGCACCACCTTAAAGAATGCAATGGAAACATAGCTAA 10372

meri CTAGGCACCACCTTAAAGAATGCAATGGAAACATAGCTAA 10532

ref CTAGGCACCACCTTAAAGAATGCAATGGAAACATAGCTAA 10651

Consensus ctaggcaccaccttaaagaatgcaatggaaacatagctaa

kal GAACAGAACATGCTGACATGTGACACATTGTGAGGAAGAT 10446

yor GAACAGAACATGCTGACATGTGACACATTGTGAGGAAGAT 10431

jen GAACAGAACATGCTGACATGTGACACATTGTGAGGAAGAT 10446

cor GAACAGAACATGCTGACATGTGACACATTGTGAGGAAGAT 10446

man GAACAGAACATGCTGACATGTGACACATTGTGAGGAAGAT 10446

uni GAACAGAACATGCTGACATGTGACACATTGTGAGGAAGAT 10446

tall GAACAGAACATGCTGACATGTGACACATTGTGAGGAAGAT 10448

quil GAACAGAACATGCTGACATGTGACACATTGTGAGGAAGAT 10412

meri GAACAGAACATGCTGACATGTGACACATTGTGAGGAAGAT 10572

ref GAACAGAACATGCTGACATGTGACACATTGTGAGGAAGAT 10691

Consensus gaacagaacatgctgacatgtgacacattgtgaggaagat

kal TGGGAACTTGAAACCATAGTTTGATAACAAATATTTGTTT 10486

yor TGGGAACTTGAAACCATAGTTTGATAACAAATATTTGTTT 10471

jen TGGGAACTTGAAACCATAGTTTGATAACAAATATTTGTTT 10486

cor TGGGAACTTGAAACCATAGTTTGATAACAAATATTTGTTT 10486

man TGGGAACTTGAAACCATAGTTTGATAACAAATATTTGTTT 10486

uni TGGGAACTTGAAACCATAGTTTGATAgCAAATATTTGTTc 10486

tall TGGGAACTTGAAACCATAGTTTGATAgCAAATATTTGTTc 10488

quil TGGGAACTTGAAACCATAGTTTGATAACAAATATTTGTTT 10452

meri TGGGAACTTGAAACCATAGTTTGATAACAAATATTTGTTT 10612

ref TGGGAACTTGAAACCATAGTTTGATAACAAATATTTGTTT 10731

Consensus tgggaacttgaaaccatagtttgata caaatatttgtt

kal AGAAGAATCACACCAATGTTTGATGAGTACCAAAGGGTTA 10526

yor AGAAGAATCACACCAATGTTTGATGAGTACCAAAGGGTTA 10511

jen AGAAGAATCACACCAATGTTTGATGAGTACCAAAGGGTTA 10526

cor AGAAGAATCACACCAATGTTTGATGAGTACCAAAGGGTTA 10526

man AGAAGAATCACACCAATGTTTGATGAGTACCAAAGGGTTA 10526

uni AGAAGAATCACACCAATGTTTGATGAGTACCAAAGGGTTA 10526

tall AGAAGAATtACACCAATGTTTGATGAGTACCAAAGGGTTA 10528

quil AGAAGAATCACACCAATGTTTGATGAGTACCAAAGGGTTA 10492

meri AGAAGAATCACACCAATGTTTGATGAGTACCAAAGGGTTA 10652

ref AGAAGAATCACACCAATGTTTGATGAGTACCAAAGGGTTA 10771

Consensus agaagaat acaccaatgtttgatgagtaccaaagggtta

kal CAAGGAAACATATGAACAAGAATTCCTTGCTGCTTGGTGA 10566

yor CAAGGAAACATATGAACAAGAATTCCTTGCTGCTTGGTGA 10551

jen CAAGGAAACATATGAACAAGAATTCCTTGCTGCTTGGTGA 10566

cor CAAGGAAACATATGAACAAGAAcTCCTTGCTGCTTGGTGA 10566

man CAAGGAAACATATGAACAAGAATTCCTTGCTGCTTGGTGA 10566

uni CAAGGAAACATATGAACAAGAATTCCTTGCTGCTTGGTGA 10566

tall CAAGGAAACATATGAACAAGAATTCCTTGCTGCTTGGTGA 10568

quil CAAGGAAACATATGAACAAGAATTCCTTGCTGCTTGGTGA 10532

meri CAAGGAAACATATGAACAAGAATTCCTTGCTGCTTGGTGA 10692

ref CAAGGAAACATATGAACAAGAATTCCTTGCTGCTTGGTGA 10811

Consensus caaggaaacatatgaacaagaa tccttgctgcttggtga

kal CACCATCTTTCCAAGCTTCCCTGGACTCTCTCCCTGTATT 10606

yor CACCATCTTTCCAAGCTTCCCTGGACTCTCTCCCTGTATT 10591

jen CACCATCTTTCCAAGCTTCCCTGGACTCTCTCCCTGTATT 10606

cor CACCATCTTTCCAAGCTTCCCTGGACTCTCTCCCTGTATT 10606

man CACCATCTTTCCAAGCTTCCCTGGACTCTCTCCCTGTATT 10606

uni CACCATCTTTCCAAGCTTCCCTGGACTCTCTCCCTGTATT 10606

tall CACCATCTTTCCAAGCTTCCCTGGACTCTCTCCCTGTATT 10608

quil CACCATCTTTCCAAGCTTCCCTGGACTCTCTCCCTGTATT 10572

meri CACCATCTTTCCAAGCTTCCCTGGACTCTCTCCCTGTATT 10732

ref CACCATCTTTCCAAGCTTCCCTGGACTCTCTCCCTGTATT 10851

Consensus caccatctttccaagcttccctggactctctccctgtatt

kal TATTATTATGCATATCAAACTTTCATTCTTTTCAAACTAT 10646

yor TATTATTATGCATATCAAACTTTCATTCTTTTCAAACTAT 10631

jen TATTATTATGCATATCAAACTTTCATTCTTTTCAAACTAT 10646

cor TATTATTATGCATATCAAACTTTCATTCTTTTCAAACTAT 10646

man TATTATTATGCATATCAAACTTTCATTCTTTTCAAACTAT 10646

uni TATTATTATGCATATCAAACTTTCATTCTTTTCAAACTAT 10646

tall TATTATTATGCATATCAAACTTTCATTCTTTTCAAACTAT 10648

quil TATTATTATGCATATCAAACTTTCATTCTTTTCAAACTAT 10612

meri TgTTATTATGCATATCAAACTTTCATTCaTTTCAAACTAT 10772

ref TgTTATTATGCATATCAAACTTTCATTCTTTTCAAACTAT 10891

Consensus t ttattatgcatatcaaactttcattc tttcaaactat

kal ACGATATAATATATCACTCTCGATTTCTGAAAGCGACACT 10686

yor ACaATATAAcATATCACTCTCGATTTCTGAAAatGACACT 10671

jen ACGATATAATATATCACTCTCGATTTCTGAAAGCGACACT 10686

cor ACGATATAATATATCACTCTCGATTTCTGAAAGCGACACT 10686

man ACGATATAATATATCACTCTCGATTTCTGAAAGCGACACT 10686

uni ACGATATAATATATCACTCTCGATTTCTGAAAGCGACACT 10686

tall ACGATATAATATATCACTCTCGATTTCTGAAAGCGACACT 10688

quil ACGATATAATATATCACTCTCGATTTCTGAAAGCGACACT 10652

meri ACaATATAAcATATCACTCTCGATTTCTGAAAatGACACT 10812

ref ACaAcATAAcATATCACTCTCGATTTCTGAAAGCGACACT 10931

Consensus ac a ataa atatcactctcgatttctgaaa gacact

kal TATTAAATAAGACAACTGTAAATTCATTATTCATACATAG 10726

yor TAT.gAgTAAGACAACTaTAAATTCATTATTCATACATAG 10710

jen TATTAAATAAGACAACTGTAAATTCATTATTCATACATAG 10726

cor TATTAAATAAGACAACTGTAAATTCATTATTCATACATAG 10726

man TATTAAATAAGACAACTGTAAATTCATTATTCATACATAG 10726

uni TATTAAATAAGACAACTGTAAATTCATTATTCATACATAG 10726

tall TATTAAATAAGACAACTGTAAATTCATTATTCATACATAG 10728

quil TATTAAATAAGACAACTGTAAATTCATTATTCATACATAG 10692

meri TAT.gAgTAAGACAACTaTAAATTCATTATTCATACATAG 10851

ref TATTgAATAAaACAACTaTAAATTCATTATTCATACATAG 10971

Consensus tat a taa acaact taaattcattattcatacatag

kal ATACATTTGGTTATACCAAAAAACTATACGATAAAAATAA 10766

yor ATACATTTtGTTATACCAAAAAACTATACGATAAAAATAA 10750

jen ATACATTTGGTTATACCAAAAAACTATACGATAAAAATAA 10766

cor ATACATTTGGTTATACCAAAAAACTATACGATAAAAATAA 10766

man ATACATTTGGTTATACCAAAAAACTATACGATAAAAATAA 10766

uni ATACATTTGGTTATACCAAAAAACTATACGATAAAAATAA 10766

tall ATACATTTGGTTATACCAAAAAACTATACGATAAAAATAA 10768

quil ATACATTTGGTTATACCAAAAAACTATACGATAAAAATAA 10732

meri ATACATTTtGTTATACCAAAAAACTATACGATAAAAATAA 10891

ref ATACATTTGGTTcTACCAAAAAACTATACGATAAAAATAA 11011

Consensus atacattt gtt taccaaaaaactatacgataaaaataa

kal AAGTACAAAGTATCAATTTTTT.TATTTGTTTCATTACTT 10805

yor AAGTACAAAGTATCAtTTTcTTaTATTTGTTTCATTACTT 10790

jen AAGTACAAAGTATCAATTTTTT.TATTTGTTTCATTACTT 10805

cor AAGTACAAAGTATCAATTTTTT.TATTTGTTTCATTACTT 10805

man AAGTACAAAGTATCAATTTTTT.TATTTGTTTCATTACTT 10805

uni AAGTACAAAGTATCAATTTTTT.TATTTGTTTCATTACTT 10805

tall AAGTACAAAGTATCAATTTTTT.TATTTGTTTCATTACTT 10807

quil AAGTACAAAGTATCAATTTTTT.TATTTGTTTCATTACTT 10771

meri AAGTACAAAGTATCAtTTTcTTaTATTTGTTTCATTACTT 10931

ref AAGTACAAAGTATCAtTTTcTTaTATTTGTTTCATTACTT 11051

Consensus aagtacaaagtatca ttt tt tatttgtttcattactt

kal ACCCCACTTTCTTTAACTGTGTCTTTGTTGTTTTCACTTT 10845

yor ACCCCACTTTCTTTAACTGTGTCTTTGTTGTTTTCACTTT 10830

jen ACCCCACTTTCTTTAACTGTGTCTTTGTTGTTTTCACTTT 10845

cor ACCCCACTTTCTTTAACTGTGTCTTTGTTGTTTTCACTTT 10845

man ACCCCACTTTCTTTAACTGTGTCTTTGTTGTTTTCACTTT 10845

uni ACCCCACTTTCTTTAACTGTGTCTTTGTTGTTTTCACTTT 10845

tall ACCCCACTTTCTTTAACTGTGTCTTTGTTGTTTTCACTTT 10847

quil ACCCCACTTTCTTTAACTGTGTCTTTGTTGTTTTCACTTT 10811

meri ACCCCACTTTCTTTAACTGTGTCTTTGTTGTTTTCACTTT 10971

ref ACCCCACTTTCTTTAACTGTGTCTTTGTTGTTTTCACTTT 11091

Consensus accccactttctttaactgtgtctttgttgttttcacttt

kal CATATGATGATTTGGATCTCATGTTATATGAAGCATGTGA 10885

yor CATATGATGATTTGGATCTCATGTTATATGAAGCATGTGA 10870

jen CATATGATGATTTGGATCTCATGTTATATGAAGCATGTGA 10885

cor CATATGATGATTTGGATCTCATGTTATATGAAGCATGTGA 10885

man CATATGATGATTTGGATCTCATGTTATATGAAGCATGTGA 10885

uni CATATGATGATTTGGATCTCATGTTATATGAAGCATGTGA 10885

tall CATATGATGATTTGGATCTCATGTTATATGAAGCATGTGA 10887

quil CATATGATGATTTGGATCTCATGTTATATGAAGCATGTGA 10851

meri CATATGATGATTTGGATCTCATGTTATATGAAGCATGTGA 11011

ref CATATGATGATTTGGATCTCATGTTATATGAAGCATGTGA 11131

Consensus catatgatgatttggatctcatgttatatgaagcatgtga

kal ACTCATTGTAACTTTTGTTGTTGTTGGTAAAGTGATCCTT 10925

yor ACTCATTGTAACTTTTGTTGTTGTTGGTAAAGTGATCCTT 10910

jen ACTCATTGTAACTTTTGTTGTTGTTGGTAAAGTGATCCTT 10925

cor ACTCATTGTAACTTTTGTTGTTGTTGGTAAAGTGATCCTT 10925

man ACTCATTGTAACTTTTGTTGTTGTTGGTAAAGTGATCCTT 10925

uni ACTCATTGTAACTTTTGTTGTTGTTGGTAAAGTGATCCTT 10925

tall ACTCATTGTAACTTTTGTTGTTGTTGGTAAAGTGATCCTT 10927

quil ACTCATTGTAACTTTTGTTGTTGTTGGTAAAGTGATCCTT 10891

meri ACTCATTGTAACTTTTGTTGTTGTTGGTAAAGTGATCCTT 11051

ref ACTCATTGTAACTTTTGTTGTTGTTGGTAAAGTGATCCTT 11171

Consensus actcattgtaacttttgttgttgttggtaaagtgatcctt

kal GTTTTTTTGTTTGTGCAGTTGTTTTTGTAACAATTAAGGT 10965

yor GTTTTTTTGTTTGTGCAGTTGTTTTTGTAACAATTAAGGT 10950

jen GTTTTTTTGTTTGTGCAGTTGTTTTTGTAACAATTAAGGT 10965

cor GTTTTTTTGTTTGTGCAGTTGTTTTTGTAACAATTAAGGT 10965

man GTTTTTTTGTTTGTGCAGTTGTTTTTGTAACAATTAAGGT 10965

uni GTTTTTTTGTTTGTGCAGTTGTTTTTGTAACAATTAAGGT 10965

tall GTTTTTTTGTTTGTGCAGTTGTTTTTGTAACAATTAAGGT 10967

quil GTTTTTTTGTTTGTGCAGTTGTTTTTGTAACAATTAAGGT 10931

meri GTTTTTTTGTTTGTGCAGTTGTTTTTGTAACAATTAAGGT 11091

ref GTTTTTTTGTTTGTGCAGTTGTTTTTGTAACAATTAAGGT 11211

Consensus gtttttttgtttgtgcagttgtttttgtaacaattaaggt

kal TCTAAACTTTTTGATTGTTTATCATTCAATGCAATTCAAG 11005

yor TCTAAACTTTTTGATTGTTTATCATTCAATGCAATTCAAG 10990

jen TCTAAACTTTTTGATTGTTTATCATTCAATGCAATTCAAG 11005

cor TCTAAACTTTTTGATTGTTTATCATTCAATGCAATTCAAG 11005

man TCTAAACTTTTTGATTGTTTATCATTCAATGCAATTCAAG 11005

uni TCTAAACTTTTTGATTGTTTATCATTCAATGCAATTCAAG 11005

tall TCTAAACTTTTTGATTGTTTATCATTCAATGCAATTCAAG 11007

quil TCTAAACTTTTTGATTGTTTATCATTCAATGCAATTCAAG 10971

meri TCTAAACTTTTTGATTGTTTATCATTCAATGCAATTCAAG 11131

ref TCTAAACTTTTTGATTGTTTATCATTCAATGCAATTCAAG 11251

Consensus tctaaactttttgattgtttatcattcaatgcaattcaag

kal AACAATGGTGAATATCATTTGAAAGCTTGTGGAATAGAAT 11045

yor AACAATGGTGAATATCATTTGAAAGCTTGTGGAATAGAAT 11030

jen AACAATGGTGAATATCATTTGAAAGCTTGTGGAATAGAAT 11045

cor AACAATGGTGAATATCATTTGAAAGCTTGTGGAATAGAAT 11045

man AACAATGGTGAATATCATTTGAAAGCTTGTGGAATAGAAT 11045

uni AACAATGGTGAATATCATTTGAAAGCTTGTGGAATAGAAT 11045

tall AACAATGGTGAATATCATTTGAAAGCTTGTGGAATAGAAT 11047

quil AACAATGGTGAATATCATTTGAAAGCTTGTGGAATAGAAT 11011

meri AACAATGGTGAATATCATTTGAAAGCTTGTGGAATAGAAT 11171

ref AACAATGGTGAATATCATTTGAAAGCTTGTGGAATAGAAT 11291

Consensus aacaatggtgaatatcatttgaaagcttgtggaatagaat

kal TAAGTTAGAAAATTGTTGATGTTTATTGCTAATGAATTTT 11085

yor TAAGTTAGAAAATTGTTGATGTTTATTGCTAATGAATTTT 11070

jen TAAGTTAGAAAATTGTTGATGTTTATTGCTAATGAATTTT 11085

cor TAAGTTAGAAAATTGTTGATGTTTATTGCTAATGAATTTT 11085

man TAAGTTAGAAAATTGTTGATGTTTATTGCTAATGAATTTT 11085

uni TAAGTTAGAAAATTGTTGATGTTTATTGCTAATGAATTTT 11085

tall TAAGTTAGAAAATTGTTGATGTTTATTGCTAATGAATTTT 11087

quil TAAGTTAGAAAATTGTTGATGTTTATTGCTAATGAATTTT 11051

meri TAAGTTAGAAAATTGTTGATGTTTATTGCTAATGAATTTT 11211

ref TAAGTTAGAAAATTGTTGATGTTTATTGCTAATGAATTTT 11331

Consensus taagttagaaaattgttgatgtttattgctaatgaatttt

kal GACTAAATTAGATAAAGGACTATATATAGAAAAACAATTT 11125

yor GACTAAATTAGATAAAGGACTATATATAGAAAAACAATTT 11110

jen GACTAAATTAGATAAAGGACTATATATAGAAAAACAATTT 11125

cor GACTAAATTAGATAAAGGACTATATATAGAAAAACAATTT 11125

man GACTAAATTAGATAAAGGACTATATATAGAAAAACAATTT 11125

uni GACTAAATTAGATAAAGGACTATATATAGAAAAACAATTT 11125

tall GACTAAATTAGATAAAGGACTATATATAGAAAAACAATTT 11127

quil GACTAAATTAGATAAAGGACTATATATAGAAAAACAATTT 11091

meri GACTAAATTAGATAAAGGACTATATATAGAAAAACAATTT 11251

ref GACTAAATTAGATAAAGGACTATtTATAGAAAAACAATTT 11371

Consensus gactaaattagataaaggactat tatagaaaaacaattt

kal GTTTGACCCATTCAAAGGAGCCTATAAAAATTTCTAACAC 11165

yor GTTTGACCCATTCAAAGGAGCCTATAAAAATTTCTAACAC 11150

jen GTTTGACCCATTCAAAGGAGCCTATAAAAATTTCTAACAC 11165

cor GTTTGACCCATTCAAAGGAGCCTATAAAAATTTCTAACAC 11165

man GTTTGACCCATTCAAAGGAGCCTATAAAAATTTCTAACAC 11165

uni GTTTGACCCATTCAAAGGAGCCTATAAAAATTTCTAACAC 11165

tall GTTTGACCCATTCAAAGGAGCCTATAAAAATTTCTAACAC 11167

quil GTTTGACCCATTCAAAGGAGCCTATAAAAATTTCTAACAC 11131

meri GTTTGACCCATTCAAAGGAGCCTATAAAAATTTCTAACAC 11291

ref GTTTGACCCATTCAAAGGAGCCTATAAAAATTTCTAACAC 11411

Consensus gtttgacccattcaaaggagcctataaaaatttctaacac

kal TTTTGAGATTTTAATTTGCAATTAATCCAAAATTAAAAAT 11205

yor TTTTGAGATTTTAATTTGCAATTAATCCAAAATTAAAAAT 11190

jen TTTTGAGATTTTAATTTGCAATTAATCCAAAATTAAAAAT 11205

cor TTTTGAGATTTTAATTTGCAATTAATCCAAAATTAAAAAT 11205

man TTTTGAGATTTTAATTTGCAATTAATCCAAAATTAAAAAT 11205

uni TTTTGAGATTTTAATTTGCAATTAATCCAAAATTAAAAAT 11205

tall TTTTGAGATTTTAATTTGCAATTAATCCAAAATTAAAAAT 11207

quil TTTTGAGATTTTAATTTGCAATTAATCCAAAATTAAAAAT 11171

meri TTTTGAGATTTTAATTTGCAATTAATCCAAAATTAAAAAT 11331

ref TTTTGAGATTTTAATTTGCAATTAATCCAAAATTAAAAAT 11451

Consensus ttttgagattttaatttgcaattaatccaaaattaaaaat

kal TGGTGAAATCTTTTAACCAGTAAGTTATAAAAAAATGTTC 11245

yor TGGTGAAATCTTTTAACCAGTAAGTTATAAAAAAATGTTC 11230

jen TGGTGAAATCTTTTAACCAGTAAGTTATAAAAAAATGTTC 11245

cor TGGTGAAATCTTTTAACCAGTAAGTTATAAAAAAATGTTC 11245

man TGGTGAAATCTTTTAACCAGTAAGTTATAAAAAAATGTTC 11245

uni TGGTGAAATCTTTTAACCAGTAAGTTATAAAAAAATGTTC 11245

tall TGGTGAAATCTTTTAACCAGTAAGTTATAAAAAAATGTTC 11247

quil TGGTGAAATCTTTTAACCAGTAAGTTATAAAAAAATGTTC 11211

meri TGGTGAAATCTTTTAACCAGTAAGTTATAAAAAAATGTTC 11371

ref TGGTGAAATCTTTTAACCAGTAAGTTATAAAAAAATGTTC 11491

Consensus tggtgaaatcttttaaccagtaagttataaaaaaatgttc

kal CAAATAGTTAGCATTGATTAAAATATAGCAATAGTTTTAA 11285

yor CAAATAGTTAGCATTGATTAAAATATAGCAATAGTTTTAA 11270

jen CAAATAGTTAGCATTGATTAAAATATAGCAATAGTTTTAA 11285

cor CAAATAGTTAGCATTGATTAAAATATAGCAATAGTTTTAA 11285

man CAAATAGTTAGCATTGATTAAAATATAGCAATAGTTTTAA 11285

uni CAAATAGTTAGCATTGATTAAAATATAGCAATAGTTTTAA 11285

tall CAAATAGTTAGCATTGATTAAAATATAGCAATAGTTTTAA 11287

quil CAAATAGTTAGCATTGATTAAAATATAGCAATAGTTTTAA 11251

meri CAAATAGTTAGCATTGATTAAAATATAGCAATAGTTTTAA 11411

ref CAAATAGTTAGCATTGATTAAAATATAGCAATAGTTTTAA 11531

Consensus caaatagttagcattgattaaaatatagcaatagttttaa

kal GGTTTAACAATTAATTAAAATAAAAAAGTAGGAAGTTCTC 11325

yor GGTTTAACAATTAATTAAAATAAAAAAGTAGGAAGTTCTC 11310

jen GGTTTAACAATTAATTAAAATAAAAAAGTAGGAAGTTCTC 11325

cor GGTTTAACAATTAATTAAAATAAAAAAGTAGGAAGTTCTC 11325

man GGTTTAACAATTAATTAAAATAAAAAAGTAGGAAGTTCTC 11325

uni GGTTTAACAATTAATTAAAATAAAAAAGTAGGAAGTTCTC 11325

tall GGTTTAACAATTAATTAAAATAAAAAAGTAGGAAGTTCTC 11327

quil GGTTTAACAATTAATTAAAATAAAAAAGTAGGAAGTTCTC 11291

meri GGTTTAACAATTAATTAAAATAAAAAAGTAGGAAGTTCTC 11451

ref GGTTTAACAATTAATTAAAATAAAAAAGTAGGAAGTTCTC 11571

Consensus ggtttaacaattaattaaaataaaaaagtaggaagttctc

kal TAGACCAATAATAAATTATGAAAAAAATGTTAGAGATATA 11365

yor TAGACCAATAATAAATTATGAAAAAAATGTTAGAGATATA 11350

jen TAGACCAATAATAAATTATGAAAAAAATGTTAGAGATATA 11365

cor TAGACCAATAATAAATTATGAAAAAAATGTTAGAGATATA 11365

man TAGACCAATAATAAATTATGAAAAAAATGTTAGAGATATA 11365

uni TAGACCAATAATAAATTATGAAAAAAATGTTAGAGATATA 11365

tall TAGACCAATAATAAATTATGAAAAAAATGTTAGAGATATA 11367

quil TAGACCAATAATAAATTATGAAAAAAATGTTAGAGATATA 11331

meri TAGACCAATAATAAATTATGAAAAAAATGTTAGAGATATA 11491

ref TAGACCAATAATAAATTATGAAAAAAATGTTAGAGATATA 11611

Consensus tagaccaataataaattatgaaaaaaatgttagagatata

kal TGTGTCTCTCATATTTATGCATACAGTAAAAGTAGAACCA 11405

yor TGTGTCTCTCATATTTATGCATACAGTAAAAGTAGAACCA 11390

jen TGTGTCTCTCATATTTATGCATACAGTAAAAGTAGAACCA 11405

cor TGTGTCTCTCATATTTATGCATACAGTAAAAGTAGAACCA 11405

man TGTGTCTCTCATATTTATGCATACAGTAAAAGTAGAACCA 11405

uni TGTGTCTCTCATATTTATGCATACAGTAAAAGTAGAACCA 11405

tall TGTGTCTCTCATATTTATGCATACAGTAAAAGTAGAACCA 11407

quil TGTGTCTCTCATATTTATGCATACAGTAAAAGTAGAACCA 11371

meri TGTGTCTCTCATATTTATGCATACAGTAAAAGTAGAACCA 11531

ref TGTGTCTCTCATATTTATGCATACAGTAAAAGTAGAACCA 11651

Consensus tgtgtctctcatatttatgcatacagtaaaagtagaacca

kal ACAAGGAGTTAGTCTAGTTGTGTAAGGTCTTATTGGATCC 11445

yor ACAAGGAGTTAGTCTAGTTGTGTAAGGTCTTATTGGATCC 11430

jen ACAAGGAGTTAGTCTAGTTGTGTAAGGTCTTATTGGATCC 11445

cor ACAAGGAGTTAGTCTAGTTGTGTAAGGTCTTATTGGATCC 11445

man ACAAGGAGTTAGTCTAGTTGTGTAAGGTCTTATTGGATCC 11445

uni ACAAGGAGTTAGTCTAGTTGTGTAAGGTCTTATTGGATCC 11445

tall ACAAGGAGTTAGTCTAGTTGTGTAAGGTCTTATTGGATCC 11447

quil ACAAGGAGTTAGTCTAGTTGTGTAAGGTCTTATTGGATCC 11411

meri ACAAGGAGTTAGTCTAGTTGTGTAAGGTCTTATTGGATCC 11571

ref ACAAGGAGTTAGTCTAGTTGTGTAAGGTCTTATTGGATCC 11691

Consensus acaaggagttagtctagttgtgtaaggtcttattggatcc

kal TCAAAGTTAAGATTTTGATTTTTGGGAGGAACAGTCACAA 11485

yor TCAAAGTTAAGATTTTGATTTTTGGGAGGAACAGTCACAA 11470

jen TCAAAGTTAAGATTTTGATTTTTGGGAGGAACAGTCACAA 11485

cor TCAAAGTTAAGATTTTGATTTTTGGGAGGAACAGTCACAA 11485

man TCAAAGTTAAGATTTTGATTTTTGGGAGGAACAGTCACAA 11485

uni TCAAAGTTAAGATTTTGATTTTTGGGAGGAACAGTCACAA 11485

tall TCAAAGTTAAGATTTTGATTTTTGGGAGGAACAGTCACAA 11487

quil TCAAAGTTAAGATTTTGATTTTTGGGAGGAACAGTCACAA 11451

meri TCAAAGTTAAGATTTTGATTTTTGGGAGGAACAGTCACAA 11611

ref TCAAAGTTAAGATTTTGATTTTTGGGAGGAACAGTCACAA 11731

Consensus tcaaagttaagattttgatttttgggaggaacagtcacaa

kal ACTCCGAAAATGATCACTTCCTTACTTAAAAAGGTGGGGA 11525

yor ACTCCGAAAATGATCACTTCCTTACTTAAAAAGGTGGGGA 11510

jen ACTCCGAAAATGATCACTTCCTTACTTAAAAAGGTGGGGA 11525

cor ACTCCGAAAATGATCACTTCCTTACTTAAAAAGGTGGGGA 11525

man ACTCCGAAAATGATCACTTCCTTACTTAAAAAGGTGGGGA 11525

uni ACTCCGAAAATGATCACTTCCTTACTTAAAAAGGTGGGGA 11525

tall ACTCCGAAAATGATCACTTCCTTACTTAAAAAGGTGGGGA 11527

quil ACTCCGAAAATGATCACTTCCTTACTTAAAAAGGTGGGGA 11491

meri ACTCCGAAAATGATCACTTCCTTACTTAAAAAGGTGGGGA 11651

ref ACTCCGAAAATGATCACTTCCTTACTTAAAAAGGTGGGGA 11771

Consensus actccgaaaatgatcacttccttacttaaaaaggtgggga

kal GAGTTGATATTACCAAAAAAGTATATAATAAAAGTAAAAT 11565

yor GAGTTGATATTACCAAAAAAGTATATAATAAAAGTAAAAT 11550

jen GAGTTGATATTACCAAAAAAGTATATAATAAAAGTAAAAT 11565

cor GAGTTGATATTACCAAAAAAGTATATAATAAAAGTAAAAT 11565

man GAGTTGATATTACCAAAAAAGTATATAATAAAAGTAAAAT 11565

uni GAGTTGATATTACCAAAAAAGTATATAATAAAAGTAAAAT 11565

tall GAGTTGATATTACCAAAAAAGTATATAATAAAAGTAAAAT 11567

quil GAGTTGATATTACCAAAAAAGTATATAATAAAAGTAAAAT 11531

meri GAGTTGATATTACCAAAAAAGTATATAATAAAAGTAAAAT 11691

ref GAGTTGATATTACCAAAAAAGTATATAATAAAAGTAAAAT 11811

Consensus gagttgatattaccaaaaaagtatataataaaagtaaaat

kal ATAAGATAGTAGAATTCATAAATAAAAAAATCAGACAATG 11605

yor ATAAGATAGTAGAATTCATAAATAAAAAAATCAGACAATG 11590

jen ATAAGATAGTAGAATTCATAAATAAAAAAATCAGACAATG 11605

cor ATAAGATAGTAGAATTCATAAATAAAAAAATCAGACAATG 11605

man ATAAGATAGTAGAATTCATAAATAAAAAAATCAGACAATG 11605

uni ATAAGATAGTAGAATTCATAAATAAAAAAATCAGACAATG 11605

tall ATAAGATAGTAGAATTCATAAATAAAAAAATCAGACAATG 11607

quil ATAAGATAGTAGAATTCATAAATAAAAAAATCAGACAATG 11571

meri ATAAGATAGTAGAATTCATAAATAAAAAAATCAGACAATG 11731

ref ATAAGATAGTAGAATTCATAAATAAAAAAATCAGACAATG 11851

Consensus ataagatagtagaattcataaataaaaaaatcagacaatg

kal AAGAATATACACAAGTACTCATAATCTAGTTATGATTTGT 11645

yor AAGAATATACACAAGTACTCATAATCTAGTTATGATTTGT 11630

jen AAGAATATACACAAGTACTCATAATCTAGTTATGATTTGT 11645

cor AAGAATATACACAAGTACTCATAATCTAGTTATGATTTGT 11645

man AAGAATATACACAAGTACTCATAATCTAGTTATGATTTGT 11645

uni AAGAATATACACAAGTACTCATAATCTAGTTATGATTTGT 11645

tall AAGAATATACACAAGTACTCATAATCTAGTTATGATTTGT 11647

quil AAGAATATACACAAGTACTCATAATCTAGTTATGATTTGT 11611

meri AAGAATATACACAAGTACTCATAATCTAGTTATGATTTGT 11771

ref AAGAATATACACAAGTACTCATAATCTAGTTATGATTTGT 11891

Consensus aagaatatacacaagtactcataatctagttatgatttgt

kal GTTAGTATTGAAAATGAAGGAAAAATTGTATGTAAGTGTT 11685

yor GTTAGTATTGAAAATGAAGGAAAAATTGTATGTAAGTGTT 11670

jen GTTAGTATTGAAAATGAAGGAAAAATTGTATGTAAGTGTT 11685

cor GTTAGTATTGAAAATGAAGGAAAAATTGTATGTAAGTGTT 11685

man GTTAGTATTGAAAATGAAGGAAAAATTGTATGTAAGTGTT 11685

uni GTTAGTATTGAAAATGAAGGAAAAATTGTATGTAAGTGTT 11685

tall GTTAGTATTGAAAATGAAGGAAAAATTGTATGTAAGTGTT 11687

quil GTTAGTATTGAAAATGAAGGAAAAATTGTATGTAAGTGTT 11651

meri GTTAGTATTGAAAATGAAGGAAAAATTGTATGTAAGTGTT 11811

ref GTTAGTATTGAAAATGAAGGAAAAATTGTATGTAAGTGTT 11931

Consensus gttagtattgaaaatgaaggaaaaattgtatgtaagtgtt

kal GGTATCATGGTTAATAAGTTGTAAGTAATTAAGTATGCTC 11725

yor GGTATCATGGTTAATAAGTTGTAAGTAATTAAGTATGCTC 11710

jen GGTATCATGGTTAATAAGTTGTAAGTAATTAAGTATGCTC 11725

cor GGTATCATGGTTAATAAGTTGTAAGTAATTAAGTATGCTC 11725

man GGTATCATGGTTAATAAGTTGTAAGTAATTAAGTATGCTC 11725

uni GGTATCATGGTTAATAAGTTGTAAGTAATTAAGTATGCTC 11725

tall GGTATCATGGTTAATAAGTTGTAAGTAATTAAGTATGCTC 11727

quil GGTATCATGGTTAATAAGTTGTAAGTAATTAAGTATGCTC 11691

meri GGTATCATGGTTAATAAGTTGTAAGTAATTAAGTATGCTC 11851

ref GGTATCATGGTTAATAAGTTGTAAGTAATTAAGTATGCTC 11971

Consensus ggtatcatggttaataagttgtaagtaattaagtatgctc

kal AAAATTTAGCAATAATAACATAATAAAAAAACATTATTTA 11765

yor AAAATTTAGCAATAATAACATAATAAAAAAACATTATTTA 11750

jen AAAATTTAGCAATAATAACATAATAAAAAAACATTATTTA 11765

cor AAAATTTAGCAATAATAACATAATAAAAAAACATTATTTA 11765

man AAAATTTAGCAATAATAACATAATAAAAAAACATTATTTA 11765

uni AAAATTTAGCAATAATAACATAATAAAAAAACATTATTTA 11765

tall AAAATTTAGCAATAATAACATAATAAAAAAACATTATTTA 11767

quil AAAATTTAGCAATAATAACATAATAAAAAAACATTATTTA 11731

meri AAAATTTAGCAATAATAACATAATAAAAAAACATTATTTA 11891

ref AAAATTTAGCAATAATAACATAATAAAAAA.CATTATTTA 12010

Consensus aaaatttagcaataataacataataaaaaa cattattta

kal TATATCAAAATAGTGAGTATGAAATTGAGTCTCAAATGAC 11805

yor TATATCAAAATAGTGAGTATGAAATTGAGTCTCAAATGAC 11790

jen TATATCAAAATAGTGAGTATGAAATTGAGTCTCAAATGAC 11805

cor TATATCAAAATAGTGAGTATGAAATTGAGTCTCAAATGAC 11805

man TATATCAAAATAGTGAGTATGAAATTGAGTCTCAAATGAC 11805

uni TATATCAAAATAGTGAGTATGAAATTGAGTCTCAAATGAC 11805

tall TATATCAAAATAGTGAGTATGAAATTGAGTCTCAAATGAC 11807

quil TATATCAAAATAGTGAGTATGAAATTGAGTCTCAAATGAC 11771

meri TATATCAAAATAGTGAGTATGAAATTGAGTCTCAAATGAC 11931

ref TATATCAAAATAGTGAGTATGAAATTGAGTCTCAAATGAC 12050

Consensus tatatcaaaatagtgagtatgaaattgagtctcaaatgac

kal TATGGCAAAAATAAGAAGGAAAAAAA.CTAAAGGTTTTTA 11844

yor TATGGCAAAAATAAGAAGGAAAAAAA.CTAAAGGTTTTTA 11829

jen TATGGCAAAAATAAGAAGGAAAAAAA.CTAAAGGTTTTTA 11844

cor TATGGCAAAAATAAGAAGGAAAAAAA.CTAAAGGTTTTTA 11844

man TATGGCAAAAATAAGAAGGAAAAAAA.CTAAAGGTTTTTA 11844

uni TATGGCAAAAATAAGAAGGAAAAAAA.CTAAAGGTTTTTA 11844

tall TATGGCAAAAATAAGAAGGAAAAAAA.CTAAAGGTTTTTA 11846

quil TATGGCAAAAATAAGAAGGAAAAAAA.CTAAAGGTTTTTA 11810

meri TATGGCAAAAATAAGAAGGAAAAAAA.CTAAAGGTTTTTA 11970

ref TATGGCAAAAATAAGAAGGAAAAAAAaCTAAAGGTTTTTA 12090

Consensus tatggcaaaaataagaaggaaaaaaa ctaaaggttttta

kal GAGCATTTTCAATCATCCATTTTTATCATATTTTTGTTGA 11884

yor GAGCATTTTCAATCATCCATTTTTATCATATTTTTGTTGA 11869

jen GAGCATTTTCAATCATCCATTTTTATCATATTTTTGTTGA 11884

cor GAGCATTTTCAATCATCCATTTTTATCATATTTTTGTTGA 11884

man GAGCATTTTCAATCATCCATTTTTATCATATTTTTGTTGA 11884

uni GAGCATTTTCAATCATCCATTTTTATCATATTTTTGTTGA 11884

tall GAGCATTTTCAATCATCCATTTTTATCATATTTTTGTTGA 11886

quil GAGCATTTTCAATCATCCATTTTTATCATATTTTTGTTGA 11850

meri GAGCATTTTCAATCATCCATTTTTATCATATTTTTGTTGA 12010

ref GAGCATTTTCAATCATCCATTTTTATCATATTTTTGTTGA 12130

Consensus gagcattttcaatcatccatttttatcatatttttgttga

kal GATGAGAATATTCCCCTTTCATTTATAGGAATGAGATTCT 11924

yor GATGAGAATATTCCCCTTTCATTTATAGGAATGAGATTCT 11909

jen GATGAGAATATTCCCCTTTCATTTATAGGAATGAGATTCT 11924

cor GATGAGAATATTCCCCTTTCATTTATAGGAATGAGATTCT 11924

man GATGAGAATATTCCCCTTTCATTTATAGGAATGAGATTCT 11924

uni GATGAGAATATTCCCCTTTCATTTATAGGAATGAGATTCT 11924

tall GATGAGAATATTCCCCTTTCATTTATAGGAATGAGATTCT 11926

quil GATGAGAATATTCCCCTTTCATTTATAGGAATGAGATTCT 11890

meri GATGAGAATATTCCCCTTTCATTTATAGGAATGAGATTCT 12050

ref GATGAGAATATTCCCCTTTCATTTATAGGAATGAGATTCT 12170

Consensus gatgagaatattcccctttcatttataggaatgagattct

kal TACTTTTTAAGGATACTATTTGTAGAAATTTTTTGTGCCT 11964

yor TACTTTTTAAGGATACTATTTGTAGAAATTTTTTGTGCCT 11949

jen TACTTTTTAAGGATACTATTTGTAGAAATTTTTTGTGCCT 11964

cor TACTTTTTAAGGATACTATTTGTAGAAATTTTTTGTGCCT 11964

man TACTTTTTAAGGATACTATTTGTAGAAATTTTTTGTGCCT 11964

uni TACTTTTTAAGGATACTATTTGTAGAAATTTTTTGTGCCT 11964

tall TACTTTTTAAGGATACTATTTGTAGAAATTTTTTGTGCCT 11966

quil TACTTTTTAAGGATACTATTTGTAGAAATTTTTTGTGCCT 11930

meri TACTTTTTAAGGATACTATTTGTAGAAATTTTTTGTGCCT 12090

ref TACTTTTTAAGGATACTATTTGTAGAAcaTTTTTGTGCCT 12210

Consensus tactttttaaggatactatttgtagaa tttttgtgcct

kal ATAA...CAAAATGTGGACTTGAAATGTAGAAAGAATGGA 12001

yor ATAA...CAAAATGTGGACTTGAAATGTAGAAAGAATGGA 11986

jen ATAA...CAAAATGTGGACTTGAAATGTAGAAAGAATGGA 12001

cor ATAA...CAAAATGTGGACTTGAAATGTAGAAAGAATGGA 12001

man ATAA...CAAAATGTGGACTTGAAATGTAGAAAGAATGGA 12001

uni ATAA...CAAAATGTGGACTTGAAATGTAGAAAGAATGGA 12001

tall ATAA...CAAAATGTGGACTTGAAATGTAGAAAGAATGGA 12003

quil ATAA...CAAAATGTGGACTTGAAATGTAGAAAGAATGGA 11967

meri ATAA...CAAAATGTGGACTTGAAATGTAGAAAGAATGGA 12127

ref ATAAtaaCAAAATGTGGACTTGAAATGTAGAAAGAATGGA 12250

Consensus ataa caaaatgtggacttgaaatgtagaaagaatgga

kal AGAGAAATGACTTTCTAATATATAAGATGATCTATTTTGC 12041

yor AGAGAAATGACTTTCTAATATATAAGATGATCTATTTTGC 12026

jen AGAGAAATGACTTTCTAATATATAAGATGATCTATTTTGC 12041

cor AGAGAAATGACTTTCTAATATATAAGATGATCTATTTTGC 12041

man AGAGAAATGACTTTCTAATATATAAGATGATCTATTTTGC 12041

uni AGAGAAATGACTTTCTAATATATAAGATGATCTATTTTGC 12041

tall AGAGAAATGACTTTCTAATATATAAGATGATCTATTTTGC 12043

quil AGAGAAATGACTTTCTAATATATAAGATGATCTATTTTGC 12007

meri AGAGAAATGACTTTCTAATATATAAGATGATCTATTTTGC 12167

ref AGAGAAATGAtTTTCTAATATATAAGATGATCTATTTTGC 12290

Consensus agagaaatga tttctaatatataagatgatctattttgc

kal .ATAGGTGTTTCTTGTATGAGTGGAAGACCCTCCAATTAA 12080

yor .ATAGGTGTTTCTTGTATGAGTGGAAGACCCTCCAATTAA 12065

jen .ATAGGTGTTTCTTGTATGAGTGGAAGACCCTCCAATTAA 12080

cor .ATAGGTGTTTCTTGTATGAGTGGAAGACCCTCCAATTAA 12080

man .ATAGGTGTTTCTTGTATGAGTGGAAGACCCTCCAATTAA 12080

uni .ATAGGTGTTTCTTGTATGAGTGGAAGACCCTCCAATTAA 12080

tall .ATAGGTGTTTCTTGTATGAGTGGAAGACCCTCCAATTAA 12082

quil .ATAGGTGTTTCTTGTATGAGTGGAAGACCCTCCAATTAA 12046

meri .ATAGGTGTTTCTTGTATGAGTGGAAGACCCTCCAATTAA 12206

ref tATAGGTGTTTCTTGTATGAGTGaAAGACCCTCCAATTAA 12330

Consensus ataggtgtttcttgtatgagtg aagaccctccaattaa

kal AGCATTGATGATTTTAGTGGTTCTTTCCTTGTTTTATTGC 12120

yor AGCATTGATGATTTTAGTGGTTCTTTCCTTGTTTTATTGC 12105

jen AGCATTGATGATTTTAGTGGTTCTTTCCTTGTTTTATTGC 12120

cor AGCATTGATGATTTTAGTGGTTCTTTCCTTGTTTTATTGC 12120

man AGCATTGATGATTTTAGTGGTTCTTTCCTTGTTTTATTGC 12120

uni AGCATTGATGATTTTAGTGGTTCTTTCCTTGTTTTATTGC 12120

tall AGCATTGATGATTTTAGTGGTTCTTTCCTTGTTTTATTGC 12122

quil AGCATTGATGATTTTAGTGGTTCTTTCCTTGTTTTATTGC 12086

meri AGCATTGATGATTTTAGTGGTTCTTTCCTTGTTTTATTGC 12246

ref AGCATTGATGATTTTAGTGGTTCTTTCCTTGTTTTATTGC 12370

Consensus agcattgatgattttagtggttctttccttgttttattgc

kal TTACTTTTTTGGGCTAAATATCATACATTAAAATTTAAAT 12160

yor TTACTTTTTTGGGCTAAATATCATACATTAAAATTTAAAT 12145

jen TTACTTTTTTGGGCTAAATATCATACATTAAAATTTAAAT 12160

cor TTACTTTTTTGGGCTAAATATCATACATTAAAATTTAAAT 12160

man TTACTTTTTTGGGCTAAATATCATACATTAAAATTTAAAT 12160

uni TTACTTTTTTGGGCTAAATATCATACATTAAAATTTAAAT 12160

tall TTACTTTTTTGGGCTAAATATCATACATTAAAATTTAAAT 12162

quil TTACTTTTTTGGGCTAAATATCATACATTAAAATTTAAAT 12126

meri TTACTTTTTTGGGCTAAATATCATACATTAAAATTTAAAT 12286

ref TTACTTTTT.GGGCTAAATATCATACATTAAAtTTTAAAT 12409

Consensus ttacttttt gggctaaatatcatacattaaa tttaaat

kal AAAGAAAATAATGGTTTATTTAAGTCAACAATCAATGTGA 12200

yor AAAGAAAATAATGGTTTATTTAAGTCAACAATCAATGTGA 12185

jen AAAGAAAATAATGGTTTATTTAAGTCAACAATCAATGTGA 12200

cor AAAGAAAATAATGGTTTATTTAAGTCAACAATCAATGTGA 12200

man AAAGAAAATAATGGTTTATTTAAGTCAACAATCAATGTGA 12200

uni AAAGAAAATAATGGTTTATTTAAGTCAACAATCAATGTGA 12200

tall AAAGAAAATAATGGTTTATTTAAGTCAACAATCAATGTGA 12202

quil AAAGAAAATAATGGTTTATTTAAGTCAACAATCAATGTGA 12166

meri AAAGAAAATAATGGTTTATTTAAGTCAACAATCAATGTGA 12326

ref AAAGAAAATAATGGTTTATTTAAGTCAACAATCAATGTGA 12449

Consensus aaagaaaataatggtttatttaagtcaacaatcaatgtga

kal AAATGGATCAATTAAATCGTAATTTATAATAGAGTACTAT 12240

yor AAATGGATCAATTAAATCGTAATTTATAATAGAGTACTAT 12225

jen AAATGGATCAATTAAATCGTAATTTATAATAGAGTACTAT 12240

cor AAATGGATCAATTAAATCGTAATTTATAATAGAGTACTAT 12240

man AAATGGATCAATTAAATCGTAATTTATAATAGAGTACTAT 12240

uni AAATGGATCAATTAAATCGTAATTTATAATAGAGTACTAT 12240

tall AAATGGATCAATTAAATCGTAATTTATAATAGAGTACTAT 12242

quil AAATGGATCAATTAAATCGTAATTTATAATAGAGTACTAT 12206

meri AAATcGATCAATTAAATCGTAATTTATAATAGAGTACTAT 12366

ref AAATGGATCAATTAAATaGTAATTTATAATAGAGTACTAT 12489

Consensus aaat gatcaattaaat gtaatttataatagagtactat

kal AGTCTTTTTATAGACTAGATAACTTCATATATATTTTAGA 12280

yor AGTCTTTTTATAGACTAGATAACTTCATATATATTTTAGA 12265

jen AGTCTTTTTATAGACTAGATAACTTCATATATATTTTAGA 12280

cor AGTCTTTTTATAGACTAGATAACTTCATATATATTTTAGA 12280

man AGTCTTTTTATAGACTAGATAACTTCATATATATTTTAGA 12280

uni AGTCTTTTTATAGACTAGATAACTTCATATATATTTTAGA 12280

tall AGTCTTTTTATAGACTAGATAACTTCATATATATTTTAGA 12282

quil AGTCTTTTTATAGACTAGATAACTTCATATATATTTTAGA 12246

meri AGTCTTTTTATAGACTAGATAACTTCATATATATTTTAGA 12406

ref AGTCTTTTTATAGACTAGATAACTTCATATATtTTTTAGA 12529

Consensus agtctttttatagactagataacttcatatat ttttaga

kal TTAAGGTCTGGTTTTTATTGTTTAAGATACATGACATTGT 12320

yor TTAAGGTCTGGTTTTTATTGTTTAAGATACATGACATTGT 12305

jen TTAAGGTCTGGTTTTTATTGTTTAAGATACATGACATTGT 12320

cor TTAAGGTCTGGTTTTTATTGTTTAAGATACATGACATTGT 12320

man TTAAGGTCTGGTTTTTATTGTTTAAGATACATGACATTGT 12320

uni TTAAGGTCTGGTTTTTATTGTTTAAGATACATGACATTGT 12320

tall TTAAGGTCTGGTTTTTATTGTTTAAGATACATGACATTGT 12322

quil TTAAGGTCTGGTTTTTATTGTTTAAGATACATGACATTGT 12286

meri TTAAGGTCTGGTTTTTATTGTTTAAGATACATGACATTGT 12446

ref TTAAGGTCTGGTTTTTATTGTTTAAGATACATGACATTGT 12569

Consensus ttaaggtctggtttttattgtttaagatacatgacattgt

kal TTTGTTTATGAATGTGAAGAATGGTTAGAACTTAGAAGTA 12360

yor TTTGTTTATGAATGTGAAGAATGGTTAGAACTTAGAAGTA 12345

jen TTTGTTTATGAATGTGAAGAATGGTTAGAACTTAGAAGTA 12360

cor TTTGTTTATGAATGTGAAGAATGGTTAGAACTTAGAAGTA 12360

man TTTGTTTATGAATGTGAAGAATGGTTAGAACTTAGAAGTA 12360

uni TTTGTTTATGAATGTGAAGAATGGTTAGAACTTAGAAGTA 12360

tall TTTGTTTATGAATGTGAAGAATGGTTAGAACTTAGAAGTA 12362

quil TTTGTTTATGAATGTGAAGAATGGTTAGAACTTAGAAGTA 12326

meri TTTGTTTATGAATGTGAAGAATGGTTAGAACTTAGAAGTA 12486

ref TTTGTTTATGAATGTGAAGAATGGTTAGAACTTAGAAGTA 12609

Consensus tttgtttatgaatgtgaagaatggttagaacttagaagta

kal ACAAGTTGACAATAAAAGACAAAGATTTAAATTTAATAGA 12400

yor ACAAGTTGACAATAAAAGACAAAGATTTAAATTTAATAGA 12385

jen ACAAGTTGACAATAAAAGACAAAGATTTAAATTTAATAGA 12400

cor ACAAGTTGACAATAAAAGACAAAGATTTAAATTTAATAGA 12400

man ACAAGTTGACAATAAAAGACAAAGATTTAAATTTAATAGA 12400

uni ACAAGTTGACAATAAAAGACAAAGATTTAAATTTAATAGA 12400

tall ACAAGTTGACAATAAAAGACAAAGATTTAAATTTAATAGA 12402

quil ACAAGTTGACAATAAAAGACAAAGATTTAAATTTAATAGA 12366

meri ACAAGTTGACAATAAAAGACAAAGATTTAAATTTAATAGA 12526

ref ACAAGTTGACAATAAAAGACAAAGATTTAAATTTAATAGA 12649

Consensus acaagttgacaataaaagacaaagatttaaatttaataga

kal GTAAGAATTACATATATTTTTAAGTTGGCATAAATGTACT 12440

yor GTAAGAATTACATATATTTTTAAGTTGGCATAAATGTACT 12425

jen GTAAGAATTACATATATTTTTAAGTTGGCATAAATGTACT 12440

cor GTAAGAATTACATATATTTTTAAGTTGGCATAAATGTACT 12440

man GTAAGAATTACATATATTTTTAAGTTGGCATAAATGTACT 12440

uni GTAAGAATTACATATATTTTTAAGTTGGCATAAATGTACT 12440

tall GTAAGAATTACATATATTTTTAAGTTGGCATAAATGTACT 12442

quil GTAAGAATTACATATATTTTTAAGTTGGCATAAATGTACT 12406

meri GTAAGAATTACATATATTTTTAAGTTGGCATAAATGTACT 12566

ref GTAAGAATTACATATATTTTTAAGTTGGCATAAATGTACT 12689

Consensus gtaagaattacatatatttttaagttggcataaatgtact

kal TTCAGTCACCTATATATTTTGTTTGACATTTTTTGAGATT 12480

yor TTCAGTCACCTATATATTTTGTTTGACATTTTTTGAGATT 12465

jen TTCAGTCACCTATATATTTTGTTTGACATTTTTTGAGATT 12480

cor TTCAGTCACCTATATATTTTGTTTGACATTTTTTGAGATT 12480

man TTCAGTCACCTATATATTTTGTTTGACATTTTTTGAGATT 12480

uni TTCAGTCACCTATATATTTTGTTTGACATTTTTTGAGATT 12480

tall TTCAGTCACCTATATATTTTGTTTGACATTTTTTGAGATT 12482

quil TTCAGTCACCTATATATTTTGTTTGACATTTTTTGAGATT 12446

meri TTCAGTCACCTATATATTTTGTTTGACATTTTTTGAGATT 12606

ref TTCAGTCACCTATATATTTTGTTTGACATTTTTTGAGATT 12729

Consensus ttcagtcacctatatattttgtttgacattttttgagatt

kal TTGGTCCCCATGTTTCTATATTTTGCAATTTTGGTCATTA 12520

yor TTGGTCCCCATGTTTCTATATTTTGCAATTTTGGTCATTA 12505

jen TTGGTCCCCATGTTTCTATATTTTGCAATTTTGGTCATTA 12520

cor TTGGTCCCCATGTTTCTATATTTTGCAATTTTGGTCATTA 12520

man TTGGTCCCCATGTTTCTATATTTTGCAATTTTGGTCATTA 12520

uni TTGGTCCCCATGTTTCTATATTTTGCAATTTTGGTCATTA 12520

tall TTGGTCCCCATGTTTCTATATTTTGCAATTTTGGTCATTA 12522

quil TTGGTCCCCATGTTTCTATATTTTGCAATTTTGGTCATTA 12486

meri TTGGTCCCCATGTTTCTATATTTTGCAATTTTGGTCATTA 12646

ref TTGGTCCCCATGTTTCTATATTTTGCAATTTTGGTCATTA 12769

Consensus ttggtccccatgtttctatattttgcaattttggtcatta

kal TATTAAAATAATAATAATTTGACGTGATAAACATGAGGAT 12560

yor TATTAAAATAATAATAATTTGACGTGATAAACATGAGGAT 12545

jen TATTAAAATAATAATAATTTGACGTGATAAACATGAGGAT 12560

cor TATTAAAATAATAATAATTTGACGTGATAAACATGAGGAT 12560

man TATTAAAATAATAATAATTTGACGTGATAAACATGAGGAT 12560

uni TATTAAAATAATAATAATTTGACGTGATAAACATGAGGAT 12560

tall TATTAAAATAATAATAATTTGACGTGATAAACATGAGGAT 12562

quil TATTAAAATAATAATAATTTGACGTGATAAACATGAGGAT 12526

meri TATTAAAATAATAATAATTTGACGTGATAAACATGAGGAT 12686

ref TATTAAAATAATAATAATTTGACGTGATAAACATGAGGAT 12809

Consensus tattaaaataataataatttgacgtgataaacatgaggat

kal AAACCAGAAAGACTAACATTACCAAATATAACAATTTGAA 12600

yor AAACCAGAAAGACTAACATTACCAAATATAACAATTTGAA 12585

jen AAACCAGAAAGACTAACATTACCAAATATAACAATTTGAA 12600

cor AAACCAGAAAGACTAACATTACCAAATATAACAATTTGAA 12600

man AAACCAGAAAGACTAACATTACCAAATATAACAATTTGAA 12600

uni AAACCAGAAAGACTAACATTACCAAATATAACAATTTGAA 12600

tall AAACCAGAAAGACTAACATTACCAAATATAACAATTTGAA 12602

quil AAACCAGAAAGACTAACATTACCAAATATAACAATTTGAA 12566

meri AAACCAGAAAGACTAACATTACCAAATATAACAATTTGAA 12726

ref AAACCAGAAAGACTAACATTACCAAATATAACAATTTGAA 12849

Consensus aaaccagaaagactaacattaccaaatataacaatttgaa

kal GGATCAAAATAAGAAATTAAAGATAAAACAAAGAAATCAA 12640

yor GGATCAAAATAAGAAATTAAAGATAAAACAAAGAAATCAA 12625

jen GGATCAAAATAAGAAATTAAAGATAAAACAAAGAAATCAA 12640

cor GGATCAAAATAAGAAATTAAAGATAAAACAAAGAAATCAA 12640

man GGATCAAAATAAGAAATTAAAGATAAAACAAAGAAATCAA 12640

uni GGATCAAAATAAGAAATTAAAGATAAAACAAAGAAATCAA 12640

tall GGATCAAAATAAGAAATTAAAGATAAAACAAAGAAATCAA 12642

quil GGATCAAAATAAGAAATTAAAGATAAAACAAAGAAATCAA 12606

meri GGATCAAAATAAGAAATTAAAGATAAAACAAAGAAATCAA 12766

ref GGATCAAAATAAGAAATTAAAGATAAAACAAAGAAATCAA 12889

Consensus ggatcaaaataagaaattaaagataaaacaaagaaatcaa

kal ATCGAAACAAAAAATTATAGATGACTAAAATTGTATTTAA 12680

yor ATCGAAACAAAAAATTATAGATGACTAAAATTGTATTTAA 12665

jen ATCGAAACAAAAAATTATAGATGACTAAAATTGTATTTAA 12680

cor ATCGAAACAAAAAATTATAGATGACTAAAATTGTATTTAA 12680

man ATCGAAACAAAAAATTATAGATGACTAAAATTGTATTTAA 12680

uni ATCGAAACAAAAAATTATAGATGACTAAAATTGTATTTAA 12680

tall ATCGAAACAAAAAATTATAGATGACTAAAATTGTATTTAA 12682

quil ATCGAAACAAAAAATTATAGATGACTAAAATTGTATTTAA 12646

meri ATCGAAACAAAAAATTATAGATGACTAAAATTGTATTTAA 12806

ref ATCGAAACAAAAAATTATAGATGACTAAAATTGTATTTAA 12929

Consensus atcgaaacaaaaaattatagatgactaaaattgtatttaa

kal CACTTTTGTTTTTTTCTCCAAAATAGCTTTAAAGCATTCT 12720

yor CACTTTTGTTTTTTTCTCCAAAATAGCTTTAAAGCATTCT 12705

jen CACTTTTGTTTTTTTCTCCAAAATAGCTTTAAAGCATTCT 12720

cor CACTTTTGTTTTTTTCTCCAAAATAGCTTTAAAGCATTCT 12720

man CACTTTTGTTTTTTTCTCCAAAATAGCTTTAAAGCATTCT 12720

uni CACTTTTGTTTTTTTCTCCAAAATAGCTTTAAAGCATTCT 12720

tall CACTTTTGTTTTTTTCTCCAAAATAGCTTTAAAGCATTCT 12722

quil CACTTTTGTTTTTTTCTCCAAAATAGCTTTAAAGCATTCT 12686

meri CACTTTTGTTTTTTTCTCCAAAATAGCTTTt...CATTCT 12843

ref CACTTTTGTTTTTTTCTCCAAAATAGCTTTAAAGCATTCT 12969

Consensus cacttttgtttttttctccaaaatagcttt cattct

kal TAAAAAAAATAGTCTCACCAACAATGTATCAGTCAAGTGG 12760

yor TAAAAAAA.TAGTCTCACCAACAATGTATCAGTCAAGTGG 12744

jen TAAAAAAAATAGTCTCACCAACAATGTATCAGTCAAGTGG 12760

cor TAAAAAAAATAGTCTCACCAACAATGTATCAGTCAAGTGG 12760

man TAAAAAAAATAGTCTCACCAACAATGTATCAGTCAAGTGG 12760

uni TAAAAAAAATAGTCTCACCAACAATGTATCAGTCAAGTGG 12760

tall TAAAAAAAATAGTCTCACCAACAATGTATCAGTCAAGTGG 12762

quil TAAAAAAAATAGTCTCACCAACAATGTATCAGTCAAGTGG 12726

meri TAAAAAAA.TAGTCTCACCAACAATGTATCAGTCAAGTGG 12882

ref TAAAAAAA.TAGTCTCACCAACAATGTATCAGTCAAGTGG 13008

Consensus taaaaaaa tagtctcaccaacaatgtatcagtcaagtgg

kal TACAAGAATTAACCATACATCACAAAATGAATTAATTTCT 12800

yor TACAAGAATTAACCATACATCACAAAATGAATTAATTTCT 12784

jen TACAAGAATTAACCATACATCACAAAATGAATTAATTTCT 12800

cor TACAAGAATTAACCATACATCACAAAATGAATTAATTTCT 12800

man TACAAGAATTAACCATACATCACAAAATGAATTAATTTCT 12800

uni TACAAGAATTAACCATACATCACAAAATGAATTAATTTCT 12800

tall TACAAGAATTAACCATACATCACAAAATGAATTAATTTCT 12802

quil TACAAGAATTAACCATACATCACAAAATGAATTAATTTCT 12766

meri TACAAGAATTAACCATACATCACAAAATGAATTAATTTCT 12922

ref TACAAGAATTAACCATACATCACAAAATGAATTAATTTCT 13048

Consensus tacaagaattaaccatacatcacaaaatgaattaatttct

kal TATATATAAGATTAAAATATATCATTGATAGCAGTGATTT 12840

yor TATATATAAGATcAAAATATATCATTGATAGCAGTGATTT 12824

jen TATATATAAGATTAAAATATATCATTGATAGCAGTGATTT 12840

cor TATATATAAGATTAAAATATATCATTGATAGCAGTGATTT 12840

man TATATATAAGATTAAAATATATCATTGATAGCAGTGATTT 12840

uni TATATATAAGATTAAAATATATCATTGATAGCAGTGATTT 12840

tall TATATATAAGATTAAAATATATCATTGATAGCAGTGATTT 12842

quil TATATATAAGATTAAAATATATCATTGATAGCAGTGATTT 12806

meri TATATATAAGATcAAAATATATCATTGATAGCAGTGATTT 12962

ref TATATATAAGATcAAAATATATCATTGATAGCAGTGATTT 13088

Consensus tatatataagat aaaatatatcattgatagcagtgattt

kal AAATAGCGGCCGCAATATTCTGGTTGTGGTGACTGCCGCA 12880

yor AAATAGCGGCCGCAATATTCTGGTTGTGGTGACTGCCGCA 12864

jen AAATAGCGGCCGCAATATTCTGGTTGTGGTGACTGCCGCA 12880

cor AAATAGCGGCCGCAATATTCTGGTTGTGGTGACTGCCGCA 12880

man AAATAGCGGCCGCAATATTCTGGTTGTGGTGACTGCCGCA 12880

uni AAATAGCGGCCGCAATATTCTGGTTGTGGTGACTGCCGCA 12880

tall AAATAGCGGCCGCAATATTCTGGTTGTGGTGACTGCCGCA 12882

quil AAATAGCGGCCGCAATATTCTGGTTGTGGTGACTGCCGCA 12846

meri AAATAGCGGCCGCAATATTCTGGTTGTGGTGACTGCCGCA 13002

ref AAATAGCGGCCGCAATATTCTGGTTGTGGTGACTGCCGCA 13128

Consensus aaatagcggccgcaatattctggttgtggtgactgccgca

kal ACCGCATTATATCGCAATTGCTATGTGATTCCGAATCACC 12920

yor ACCGCATTATATCGCAATTGCTATGTGATTCCGAATCACC 12904

jen ACCGCATTATATCGCAATTGCTATGTGATTCCGAATCACC 12920

cor ACCGCATTATATCGCAATTGCTATGTGATTCCGAATCACC 12920

man ACCGCATTATATCGCAATTGCTATGTGATTCCGAATCACC 12920

uni ACCGCATTATATCGCAATTGCTATGTGATTCCGAATCACC 12920

tall ACCGCATTATATCGCAATTGCTATGTGATTCCGAATCACC 12922

quil ACCGCATTATATCGCAATTGCTATGTGATTCCGAATCACC 12886

meri ACCGCATTATATCGCAATTGCTATGTGATTCCGAATCACC 13042

ref ACCGCATTATATCGCAATTGCTATaTGATTCCGAATCACC 13168

Consensus accgcattatatcgcaattgctat tgattccgaatcacc

kal TTGATGCGGCAACATTGAGCCGCAATGTACCCGTATCGTG 12960

yor TTGATGCGGCAACATTGAGCCGCAATGTACCCGTATCGTG 12944

jen TTGATGCGGCAACATTGAGCCGCAATGTACCCGTATCGTG 12960

cor TTGATGCGGCAACATTGAGCCGCAATGTACCCGTATCGTG 12960

man TTGATGCGGCAACATTGAGCCGCAATGTACCCGTATCGTG 12960

uni TTGATGCGGCAACATTGAGCCGCAATGTACCCGTATCGTG 12960

tall TTGATGCGGCAACATTGAGCCGCAATGTACCCGTATCGTG 12962

quil TTGATGCGGCAACATTGAGCCGCAATGTACCCGTATCGTG 12926

meri TTGATGCGGCAACATTGAGCCGCAATGTACCCGTATCGTG 13082

ref TTGATGCGGCAACATTGAGCCGCAATGTACCCGTATCGTG 13208

Consensus ttgatgcggcaacattgagccgcaatgtacccgtatcgtg

kal TTACAATGTAACCGCATTTGGATTACATCAAATTAAAAAA 13000

yor TTACAATGTAACCGCATaTGGATTACATCAAATTAAAAAA 12984

jen TTACAATGTAACCGCATTTGGATTACATCAAATTAAAAAA 13000

cor TTACAATGTAACCGCATTTGGATTACATCAAATTAAAAAA 13000

man TTACAATGTAACCGCATTTGGATTACATCAAATTAAAAAA 13000

uni TTACAATGTAACCGCATTTGGATTACATCAAATTAAAAAA 13000

tall TTACAATGTAACCGCATTTGGATTACATCAAATTAAAAAA 13002

quil TTACAATGTAACCGCATTTGGATTACATCAAATTAAAAAA 12966

meri TTACAATGTAACCGCATTTGGATTACATCAAATTAAAAAA 13122

ref TTACAATGTAACCGCATaTGGATTACATCAAATTAAAAAA 13248

Consensus ttacaatgtaaccgcat tggattacatcaaattaaaaaa

kal TAGTTTTTAATCACAAAAATAATTCACACTATAATGGACA 13040

yor TAGTTTTTAATCACAAAAATAATTCACACTATAATGGACA 13024

jen TAGTTTTTAATCACAAAAATAATTCACACTATAATGGACA 13040

cor TAGTTTTTAATCACAAAAATAATTCACACTATAATGGACA 13040

man TAGTTTTTAATCACAAAAATAATTCACACTATAATGGACA 13040

uni TAGTTTTTAATCACAAAAATAATTCACACTATAATGGACA 13040

tall TAGTTTTTAATCACAAAAATAATTCACACTATAATGGACA 13042

quil TAGTTTTTAATCACAAAAATAATTCACACTATAATGGACA 13006

meri TAGTTTTTAATCACAAAAATAATTCACACTATAATGGACA 13162

ref TAGTTTTTAATCACAAAAATAATTCACACTATAATGGACA 13288

Consensus tagtttttaatcacaaaaataattcacactataatggaca

kal CAATCCACGTCACAATAGCCACATTCACAATAACCACAAT 13080

yor CAATCCACGTCACAATAGCCACATTCACAATAACCACAAT 13064

jen CAATCCACGTCACAATAGCCACATTCACAATAACCACAAT 13080

cor CAATCCACGTCACAATAGCCACATTCACAATAACCACAAT 13080

man CAATCCACGTCACAATAGCCACATTCACAATAACCACAAT 13080

uni CAATCCACGTCACAATAGCCACATTCACAATAACCACAAT 13080

tall CAATCCACGTCACAATAGCCACATTCACAATAACCACAAT 13082

quil CAATCCACGTCACAATAGCCACATTCACAATAACCACAAT 13046

meri CAATCCACGTCACAATAGCCACATTCACAATAACCACAAT 13202

ref CAATCCACGTCACAATAGCCACATTCACAATAACCACAAT 13328

Consensus caatccacgtcacaatagccacattcacaataaccacaat

kal CGTTAGCACAATGGCGACTATGATCACAATTTAAAACAAT 13120

yor CGTTAGCACAATGGCGACTATGATCACAATTTAAAACAAT 13104

jen CGTTAGCACAATGGCGACTATGATCACAATTTAAAACAAT 13120

cor CGTTAGCACAATGGCGACTATGATCACAATTTAAAACAAT 13120

man CGTTAGCACAATGGCGACTATGATCACAATTTAAAACAAT 13120

uni CGTTAGCACAATGGCGACTATGATCACAATTTAAAACAAT 13120

tall CGTTAGCACAATGGCGACTATGATCACAATTTAAAACAAT 13122

quil CGTTAGCACAATGGCGACTATGATCACAATTTAAAACAAT 13086

meri CGTTAGCACAATGGCGACTATGATCACAATTTAAAACAAT 13242

ref CGTTAGCACAATGGCGACTATGATCACAATTTAAAACAAT 13368

Consensus cgttagcacaatggcgactatgatcacaatttaaaacaat

kal AGTATAAAAATAATAGTGCCCAGTATTAAATAGTTCTATA 13160

yor AGTATAAAAATAATAGTGgCCAGTATTAAATAGTTCTATA 13144

jen AGTATAAAAATAATAGTGCCCAGTATTAAATAGTTCTATA 13160

cor AGTATAAAAATAATAGTGCCCAGTATTAAATAGTTCTATA 13160

man AGTATAAAAATAATAGTGCCCAGTATTAAATAGTTCTATA 13160

uni AGTATAAAAATAATAGTGCCCAGTATTAAATAGTTCTATA 13160

tall AGTATAAAAATAATAGTGCCCAGTATTAAATAGTTCTATA 13162

quil AGTATAAAAATAATAGTGCCCAGTATTAAATAGTTCTATA 13126

meri AGTATAAAAATAATAGTGCCCAGTATTAAATAGTTCTATA 13282

ref AGTATAAAAATAATAGTGgCCAGTATTAAATAGTTCTATA 13408

Consensus agtataaaaataatagtg ccagtattaaatagttctata

kal AAAGCCATCAATCTACCGATAAAAAAGAATACCTGAAAAT 13200

yor AAAGCCATCAATCTACCGATAAAAAAGAATACCTGAAAAT 13184

jen AAAGCCATCAATCTACCGATAAAAAAGAATACCTGAAAAT 13200

cor AAAGCCATCAATCTACCGATAAAAAAGAATACCTGAAAAT 13200

man AAAGCCATCAATCTACCGATAAAAAAGAATACCTGAAAAT 13200

uni AAAGCCATCAATCTACCGATAAAAAAGAATACCTGAAAAT 13200

tall AAAGCCATCAATCTACCGATAAAAAAGAATACCTGAAAAT 13202

quil AAAGCCATCAATCTACCGATAAAAAAGAATACCTGAAAAT 13166

meri AAAGCCATCAATCTACCGATAAAAAAGAATACCTGAAAAT 13322

ref AAAGCCATCAATCTACCGATAAAAAAGAATACCTGAAAAT 13448

Consensus aaagccatcaatctaccgataaaaaagaatacctgaaaat

kal CAATCAACGGTTGAGAAGTTTCCAAACTGAAAAAGCCCGG 13240

yor CAATCAACGGTTGAGAAGTTTCCAAACTGAAAAAGCCCGG 13224

jen CAATCAACGGTTGAGAAGTTTCCAAACTGAAAAAGCCCGG 13240

cor CAATCAACGGTTGAGAAGTTTCCAAACTGAAAAAGCCCGG 13240

man CAATCAACGGTTGAGAAGTTTCCAAACTGAAAAAGCCCGG 13240

uni CAATCAACGGTTGAGAAGTTTCCAAACTGAAAAAGCCCGG 13240

tall CAATCAACGGTTGAGAAGTTTCCAAACTGAAAAAGCCCGG 13242

quil CAATCAACGGTTGAGAAGTTTCCAAACTGAAAAAGCCCGG 13206

meri CAATCAACGGTTGAGAAGTTTCCAAACTGAAAAAGCCCGG 13362

ref CAATCAACGGTTGAGAAGTTTCCAAACTGAAAAAGCCCGG 13488

Consensus caatcaacggttgagaagtttccaaactgaaaaagcccgg

kal TCCCACATCACAATCCAGTAAAATCCCCAAAATACCCTAA 13280

yor TCCCACATCACAATCCAGTAAAATCCCCAAAATACCCTAA 13264

jen TCCCACATCACAATCCAGTAAAATCCCCAAAATACCCTAA 13280

cor TCCCACATCACAATCCAGTAAAATCCCCAAAATACCCTAA 13280

man TCCCACATCACAATCCAGTAAAATCCCCAAAATACCCTAA 13280

uni TCCCACATCACAATCCAGTAAAATCCCCAAAATACCCTAA 13280

tall TCCCACATCACAATCCAGTAAAATCCCCAAAATACCCTAA 13282

quil TCCCACATCACAATCCAGTAAAATCCCCAAAATACCCTAA 13246

meri TCCCACATCACAATCCAGTAAAATCCCCAAAATACCCTAA 13402

ref TCCCACATCACAATCCAGTAAAATCCCCAAAATACCCTAA 13528

Consensus tcccacatcacaatccagtaaaatccccaaaataccctaa

kal GAAACCAGCTGATATTCCAAAGCAGCCACCATGGCGTGTC 13320

yor GAAACCAGCTGATATTCCAAAGCAGCCACCATGGCGTGTC 13304

jen GAAACCAGCTGATATTCCAAAGCAGCCACCATGGCGTGTC 13320

cor GAAACCAGCTGATATTCCAAAGCAGCCACCATGGCGTGTC 13320

man GAAACCAGCTGATATTCCAAAGCAGCCACCATGGCGTGTC 13320

uni GAAACCAGCTGATATTCCAAAGCAGCCACCATGGCGTGTC 13320

tall GAAACCAGCTGATATTCCAAAGCAGCCACCATGGCGTGTC 13322

quil GAAACCAGCTGATATTCCAAAGCAGCCACCATGGCGTGTC 13286

meri GAAACCAGCTGATATTCCAAAGCAGCCACCATGGCGTGTC 13442

ref GAAACCAGCTGATATTCCAAAGCAGCCACCATGGCGTGTC 13568

Consensus gaaaccagctgatattccaaagcagccaccatggcgtgtc

kal ACTTTCCAACTGCGTTCAAAGCTCGTCGCTTTCAATGGCA 13360

yor ACTTTCCAACTGCGTTCAAAGCTCGTCGCTTTCAATGGCA 13344

jen ACTTTCCAACTGCGTTCAAAGCTCGTCGCTTTCAATGGCA 13360

cor ACTTTCCAACTGCGTTCAAAGCTCGTCGCTTTCAATGGCA 13360

man ACTTTCCAACTGCGTTCAAAGCTCGTCGCTTTCAATGGCA 13360

uni ACTTTCCAACTGCGTTCAAAGCTCGTCGCTTTCAATGGCA 13360

tall ACTTTCaAACTGCGTTCAAAGCTCGTCGCTTTCAATGGCA 13362

quil ACTTTCCAACTGCGTTCAAAGCTCGTCGCTTTCAATGGCA 13326

meri ACTTTCCAACTGCGTTCAAAGCTCGTCGCTTTCAATGGCA 13482

ref ACTTTCCAACTGCGTTCAAAGCTCGTCGCTTTCAATGGCA 13608

Consensus actttc aactgcgttcaaagctcgtcgctttcaatggca

kal GAAACACACTCACTTCTTCAACATGTTCTCTCTCTGATTT 13400

yor GAAACACACTCACTTCTTCAACATGTTCTCTCTCTGATTT 13384

jen GAAACACACTCACTTCTTCAACATGTTCTCTCTCTGATTT 13400

cor GAAACACACTCACTTCTTCAACATGTTCTCTCTCTGATTT 13400

man GAAACACACTCACTTCTTCAACATGTTCTCTCTCTGATTT 13400

uni GAAACACACTCACTTCTTCAACATGTTCTCTCTCTGATTT 13400

tall GAAACtCAC..AaTTCTTCAtCATGTTCTCTCTCTGATTT 13400

quil GAAACACACTCACTTCTTCAACATGTTCTCTCTCTGATTT 13366

meri GAAACACACTCACTTCTTCAACATGTTCTCTCTCTGATTT 13522

ref GAAACACACTCACTTCTTCAACATGTTCTCTCTCTGATTT 13648

Consensus gaaac cac a ttcttca catgttctctctctgattt

kal CAGAGCCTCTTACAAACCCTAGAAACTCAAATTTCCTTAC 13440

yor CAGAGCCTCTTACAAACCCTAGAAACTCAAATTTCCTTAC 13424

jen CAGAGCCTCTTACAAACCCTAGAAACTCAAATTTCCTTAC 13440

cor CAGAGCCTCTTACAAACCCTAGAAACTCAAATTTCCTTAC 13440

man CAGAGCCTCTTACAAACCCTAGAAACTCAAATTTCCTTAC 13440

uni CAGAGCCTCTTACAAACCCTAGAAACTCAAATTTCCTTAC 13440

tall CAGAGCCTCTTACAAACCCTAGAAACTCAAATTTCCTTAC 13440

quil CAGAGCCTCTTACAAACCCTAGAAACTCAAATTTCCTTAC 13406

meri CAGAGCCTCTTACAAACCCTAGAAACTCAAATTTCCTTAC 13562

ref CAGAGCCTCTTACAAACCCTAGAAACTCAAATTTCCTTAC 13688

Consensus cagagcctcttacaaaccctagaaactcaaatttccttac

kal TCCCTTTTCTGGGTACCCTTCTTCCTCTTTCTTTCAACGC 13480

yor TCCCTTTTCTGGGTACCCTTCTTCCTCTTTCTTTCAACGC 13464

jen TCCCTTTTCTGGGTACCCTTCTTCCTCTTTCTTTCAACGC 13480

cor TCCCTTTTCTGGGTACCCTTCTTCCTCTTTCTTTCAACGC 13480

man TCCCTTTTCTGGGTACCCTTCTTCCTCTTTCTTTCAACGC 13480

uni TCCCTTTTCTGGGTACCCTTCTTCCTCTTTCTTTCAACGC 13480

tall TCCCTTTTCTGGGTACCCTTCTTCCTCTTTCTTTCAACGC 13480

quil TCCCTTTTCTGGGTACCCTTCTTCCTCTTTCTTTCAACGC 13446

meri TCCCTTTTCTGGGTACCCTTCTTCCTCTTTCTTTCAACGC 13602

ref TCCCTTTTCTGGGTACCCTTCTTCCTCTTTCTTTCAACGC 13728

Consensus tcccttttctgggtacccttcttcctctttctttcaacgc

kal CAAATGTGGGTTCTCATAAAGTTCTCAACTTTATAAAAAT 13520

yor CAAATGTGGGTTCTCATAAAGTTCTCAACTTTATAAAAAT 13504

jen CAAATGTGGGTTCTCATAAAGTTCTCAACTTTATAAAAAT 13520

cor CAAATGTGGGTTCTCATAAAGTTCTCAACTTTATAAAAAT 13520

man CAAATGTGGGTTCTCATAAAGTTCTCAACTTTATAAAAAT 13520

uni CAAATGTGGGTTCTCATAAAGTTCTCAACTTTATAAAAAT 13520

tall CAAATGTGGGTTCTCATAAAGTTCTCAACTTTATAAAAAT 13520

quil CAAATGTGGGTTCTCATAAAGTTCTCAACTTTATAAAAAT 13486

meri CAAATGTGGGTTCTCATAAAGTTCTCAACTTTATAAAAAT 13642

ref CAAATGTGGGTTCTCATAAAGTTCTCAACTTTATAAAAAT 13768

Consensus caaatgtgggttctcataaagttctcaactttataaaaat

kal ATTTTAGTTTTTTTGTTTCTATTTTTTAAATTGTTTGCAA 13560

yor ATTTTAGTTTTTTTGTTTCTATTTTTTAAATTGTTTGCAA 13544

jen ATTTTAGTTTTTTTGTTTCTATTTTTTAAATTGTTTGCAA 13560

cor ATTTTAGTTTTTTTGTTTCTATTTTTTAAATTGTTTGCAA 13560

man ATTTTAGTTTTTTTGTTTCTATTTTTTAAATTGTTTGCAA 13560

uni ATTTTAGTTTTTTTGTTTCTATTTTTTAAATTGTTTGCAA 13560

tall ATTTTAGTTTTTTTGTTTCTATTTTTTAAATTGTTTGCAA 13560

quil ATTTTAGTTTTTTTGTTTCTATTTTTTAAATTGTTTGCAA 13526

meri ATTTTAGTTTTTTTGTTTCTATTTTTTAAATTGTTTGCAA 13682

ref ATTTTAGTTTTTTTGTTTCTATTTTTTAAATTGTTTGCAA 13808

Consensus attttagtttttttgtttctattttttaaattgtttgcaa

kal AAGAAGAAAGGGTTTGAATGATAAGCATTTATTACTTCAT 13600

yor AAGAAGAAAGGGTTTGAATGATAAGCATTTATTACTTCAT 13584

jen AAGAAGAAAGGGTTTGAATGATAAGCATTTATTACTTCAT 13600

cor AAGAAGAAAGGGTTTGAATGATAAGCATTTATTACTTCAT 13600

man AAGAAGAAAGGGTTTGAATGATAAGCATTTATTACTTCAT 13600

uni AAGAAGAAAGGGTTTGAATGATAAGCATTTATTACTTCAT 13600

tall AAGAAGAAAGGGTTTGAATGATAAGCATTTATTACTTCAT 13600

quil AAGAAGAAAGGGTTTGAATGATAAGCATTTATTACTTCAT 13566

meri AAGAAGAAAGGGTTTGAATGATAAGCATTTATTACTTCAT 13722

ref AAGAAGAAAGGGTTTGAATGATAAGCATTTATTACTTCAT 13848

Consensus aagaagaaagggtttgaatgataagcatttattacttcat

kal TGTTTAATTTAATGGCTGTTGTTGAATATAATAGTGATGT 13640

yor TGTTTAATTTAATGGCTGTTGTTGAATATAATAGTGATGT 13624

jen TGTTTAATTTAATGGCTGTTGTTGAATATAATAGTGATGT 13640

cor TGTTTAATTTAATGGCTGTTGTTGAATATAATAGTGATGT 13640

man TGTTTAATTTAATGGCTGTTGTTGAATATAATAGTGATGT 13640

uni TGTTTAATTTAATGGCTGTTGTTGAATATAATAGTGATGT 13640

tall TGTTTAATTTAATGGCTGTTGTTGAATATAATAGTGATGT 13640

quil TGTTTAATTTAATGGCTGTTGTTGAATATAATAGTGATGT 13606

meri TGTTTAATTTAATGGCTGTTGTTGAATATAATAGTGATGT 13762

ref TGTTTAATTTAATGGCTGTTGTTGAATATAATAGTGATGT 13888

Consensus tgtttaatttaatggctgttgttgaatataatagtgatgt

kal TATTTAATGGGGTTGGAAGTGTAGTAGTTGCTAAGTACTA 13680

yor TATTTAATGGGGTTGGAAGTGTAGTAGTTGCTAAGTACTA 13664

jen TATTTAATGGGGTTGGAAGTGTAGTAGTTGCTAAGTACTA 13680

cor TATTTAATGGGGTTGGAAGTGTAGTAGTTGCTAAGTACTA 13680

man TATTTAATGGGGTTGGAAGTGTAGTAGTTGCTAAGTACTA 13680

uni TATTTAATGGGGTTGGAAGTGTAGTAGTTGCTAAGTACTA 13680

tall TATTTAATGGGGTTGGAAGTGTAGTAGTTGCTAAGTACTA 13680

quil TATTTAATGGGGTTGGAAGTGTAGTAGTTGCTAAGTACTA 13646

meri TATTTAATGGGGTTGGAAGTGTAGTAGTTGCTAAGTACTA 13802

ref TATTTAATGGGGTTGGAAGTGTAGTAGTTGCTAAGTACTA 13928

Consensus tatttaatggggttggaagtgtagtagttgctaagtacta

kal GTAATTGTTAAGTCAATTTAATTTAGGGTTTTAAATACTA 13720

yor GTAATTGTTAAGTCAATTTAATTTAGGGTTTTAAATACTA 13704

jen GTAATTGTTAAGTCAATTTAATTTAGGGTTTTAAATACTA 13720

cor GTAATTGTTAAGTCAATTTAATTTAGGGTTTTAAATACTA 13720

man GTAATTGTTAAGTCAATTTAATTTAGGGTTTTAAATACTA 13720

uni GTAATTGTTAAGTCAATTTAATTTAGGGTTTTAAATACTA 13720

tall GTAATTGTTAAGTCAATTTAATTTAGGGTTTTAAATACTA 13720

quil GTAATTGTTAAGTCAATTTAATTTAGGGTTTTAAATACTA 13686

meri GTAATTGTTAAGTCAATTTAATTTAGGGTTTTAAATACTA 13842

ref GTAATTGTTAAGTCAATTTAATTTAGGGTTTTAAATACTA 13968

Consensus gtaattgttaagtcaatttaatttagggttttaaatacta

kal TTCCTTTCTTATTCTTCTTATTTATTGAATGCACTTGTTG 13760

yor TTCCTTTCTTATTCTTCTTATTTATTGAATGCACTTGTTG 13744

jen TTCCTTTCTTATTCTTCTTATTTATTGAATGCACTTGTTG 13760

cor TTCCTTTCTTATTCTTCTTATTTATTGAATGCACTTGTTG 13760

man TTCCTTTCTTATTCTTCTTATTTATTGAATGCACTTGTTG 13760

uni TTCCTTTCTTATTCTTCTTATTTATTGAATGCACTTGTTG 13760

tall TTCCTTTCTTATTCTTCTTAnnnnnnnnnnnnnnnnGTTG 13760

quil TTCCTTTCTTATTCTTCTTATTTATTGAATGCACTTGTTG 13726

meri TTCCTTTCTTATTCTTCTTATTTATTGAATGCACTTGTTG 13882

ref TTCCTTTCTTATTCTTCTTATTTATTGAATGCACTTGTTG 14008

Consensus ttcctttcttattcttctta gttg

kal TTTCTGGAAGCATTATTTGAAGTTTGCAACAATTTCTTTC 13800

yor TTTCTGGAAGCATTATTTGAAGTTTGCAACAATTTCTTTC 13784

jen TTTCTGGAAGCATTATTTGAAGTTTGCAACAATTTCTTTC 13800

cor TTTCTGGAAGCATTATTTGAAGTTTGCAACAATTTCTTTC 13800

man TTTCTGGAAGCATTATTTGAAGTTTGCAACAATTTCTTTC 13800

uni TTTCTGGAAGCATTATTTGAAGTTTGCAACAATTTCTTTC 13800

tall TTTCTGGAAGCATTATTTGAAGTTTGCAACAATTTCTTTC 13800

quil TTTCTGGAAGCATTATTTGAAGTTTGCAACAATTTCTTTC 13766

meri TTTCTGGAAGCATTATTTGAAGTTTGCAACAATTTCTTTC 13922

ref TTTCTGGAAGCATTATTTGAAGTTTGCAACAATTTCTTTC 14048

Consensus tttctggaagcattatttgaagtttgcaacaatttctttc

kal AGTGGTTTTCATTTAAGATGATGGTTTATTGATTCTCCTT 13840

yor AGTGGTTTTCATTTAAGATGATGGTTTATTGATTCTCCTT 13824

jen AGTGGTTTTCATTTAAGATGATGGTTTATTGATTCTCCTT 13840

cor AGTGGTTTTCATTTAAGATGATGGTTTATTGATTCTCCTT 13840

man AGTGGTTTTCATTTAAGATGATGGTTTATTGATTCTCCTT 13840

uni AGTGGTTTTCATTTAAGATGATGGTTTATTGATTCTCCTT 13840

tall AGTGGTTTTCATTTAAGATGATGGTTTATTGATTCTCCTT 13840

quil AGTGGTTTTCATTTAAGATGATGGTTTATTGATTCTCCTT 13806

meri AGTGGTTTTCATTTAAGATGATGGTTTATTGATTCTCCTT 13962

ref AGTGGTTTTCATTTAAGATGATGGTTTATTGATTCTCCTT 14088

Consensus agtggttttcatttaagatgatggtttattgattctcctt

kal TATCTTTCTGTTGATTACATTTAATTGCTTATTTATTGTA 13880

yor TATCTTTCTGTTGATTACATTTAATTGCTTATTTATTGTA 13864

jen TATCTTTCTGTTGATTACATTTAATTGCTTATTTATTGTA 13880

cor TATCTTTCTGTTGATTACATTTAATTGCTTATTTATTGTA 13880

man TATCTTTCTGTTGATTACATTTAATTGCTTATTTATTGTA 13880

uni TATCTTTCTGTTGATTACATTTAATTGCTTATTTATTGTA 13880

tall TATCTTTCTGTTGATTACATTTAATTGCTTATTTATTGTA 13880

quil TATCTTTCTGTTGATTACATTTAATTGCTTATTTATTGTA 13846

meri TATCTTTCTGTTGATTACATTTAATTGCTTATTTATTGTA 14002

ref TATCTTTCTGTTGATTACATTTAATTGCTTATTTATTGTA 14128

Consensus tatctttctgttgattacatttaattgcttatttattgta

kal TTTTCACATTTGATTTGTAAAAGAAAGATACTTGAATGAG 13920

yor TTTTCACATTTGATTTGTAAAAGAAAGATACTTGAATGAG 13904

jen TTTTCACATTTGATTTGTAAAAGAAAGATACTTGAATGAG 13920

cor TTTTCACATTTGATTTGTAAAAGAAAGATACTTGAATGAG 13920

man TTTTCACATTTGATTTGTAAAAGAAAGATACTTGAATGAG 13920

uni TTTTCACATTTGATTTGTAAAAGAAAGATACTTGAATGAG 13920

tall TTTTCACATTTGATTTGTAAAAGAAAGATACTTGAATGAG 13920

quil TTTTCACATTTGATTTGTAAAAGAAAGATACTTGAATGAG 13886

meri TTTTCACATTTGATTTGTAAAAGAAAGATACTTGAATGAG 14042

ref TTTTCACATTTGATTTGTAAAAGAAAGATACTTGAATGAG 14168

Consensus ttttcacatttgatttgtaaaagaaagatacttgaatgag

kal TTTAGGATGGGTGTTATGTATAATACTAGCAATTTTTGCA 13960

yor TTTAGGATGGGTGTTATGTATAATACTAGCAATTTTTGCA 13944

jen TTTAGGATGGGTGTTATGTATAATACTAGCAATTTTTGCA 13960

cor TTTAGGATGGGTGTTATGTATAATACTAGCAATTTTTGCA 13960

man TTTAGGATGGGTGTTATGTATAATACTAGCAATTTTTGCA 13960

uni TTTAGGATGGGTGTTATGTATAATACTAGCAATTTTTGCA 13960

tall TTTAGGATGGGTGTTATGTATAATACTAGCAATTTTTGCA 13960

quil TTTAGGATGGGTGTTATGTATAATACTAGCAATTTTTGCA 13926

meri TTTAGGATGGGTGTTATGTATAATACTAGCAATTTTTGCA 14082

ref TTTAGGATGGGTGTTATGTATAATACTAGCAATTTTTGCA 14208

Consensus tttaggatgggtgttatgtataatactagcaatttttgca

kal TGTGTTATTTTCATAAATGGTTTATGTTTCTATTTGTTAA 14000

yor TGTGTTATTTTCATAAATGGTTTATGTTTCTATTTGTTAA 13984

jen TGTGTTATTTTCATAAATGGTTTATGTTTCTATTTGTTAA 14000

cor TGTGTTATTTTCATAAATGGTTTATGTTTCTATTTGTTAA 14000

man TGTGTTATTTTCATAAATGGTTTATGTTTCTATTTGTTAA 14000

uni TGTGTTATTTTCATAAATGGTTTATGTTTCTATTTGTTAA 14000

tall TGTGTTATTTTCATAAATGGTTTATGTTTCTATTTGTTAA 14000

quil TGTGTTATTTTCATAAATGGTTTATGTTTCTATTTGTTAA 13966

meri TGTGTTATTTTCATAAATGGTTTATGTTTCTATTTGTTAA 14122

ref TGTGTTATTTTCATAAATGGTTTATGTTTCTATTTGTTAA 14248

Consensus tgtgttattttcataaatggtttatgtttctatttgttaa

kal TTGTTAACTCAATGTTTTGTCTTCAATACACTTGATAATT 14040

yor TTGTTAACTCAATGTTTTGTCTTCAATACACTTGATAATT 14024

jen TTGTTAACTCAATGTTTTGTCTTCAATACACTTGATAATT 14040

cor TTGTTAACTCAATGTTTTGTCTTCAATACACTTGATAATT 14040

man TTGTTAACTCAATGTTTTGTCTTCAATACACTTGATAATT 14040

uni TTGTTAACTCAATGTTTTGTCTTCAATACACTTGATAATT 14040

tall TTGTTAACTCAATGTTTTGTCTTCAATACACTTGATAATT 14040

quil TTGTTAACTCAATGTTTTGTCTTCAATACACTTGATAATT 14006

meri TTGTTAACTCAATGTTTTGTCTTCAATACACTTGATAATT 14162

ref TTGTTAACTCAATGTTTTGTCTTCAATACACTTGATAATT 14288

Consensus ttgttaactcaatgttttgtcttcaatacacttgataatt

kal GTTAACTTACTTTGTTTTTGTTGTTGCTATTTTTATTTGC 14080

yor GTTAACTTACTTTGTTTTTGTTGTTGCTATTTTTATTTGC 14064

jen GTTAACTTACTTTGTTTTTGTTGTTGCTATTTTTATTTGC 14080

cor GTTAACTTACTTTGTTTTTGTTGTTGCTATTTTTATTTGC 14080

man GTTAACTTACTTTGTTTTTGTTGTTGCTATTTTTATTTGC 14080

uni GTTAACTTACTTTGTTTTTGTTGTTGCTATTTTTATTTGC 14080

tall GTTAACTTACTTTGTTTTTGTTGTTGCTATTTTTATTTGC 14080

quil GTTAACTTACTTTGTTTTTGTTGTTGCTATTTTTATTTGC 14046

meri GTTAACTTACTTTGTTTTTGTTGTTGCTATTTTTATTTGC 14202

ref GTTAACTTACTTTGTTTTTGTTGTTGCTATTTTTATTTGC 14328

Consensus gttaacttactttgtttttgttgttgctatttttatttgc

kal AGGATATAGTTGCTGTTCATCATGGCTTGGGATTTGGATC 14120

yor AGGATATAGTTGCTGTTCATCATGGCTTGGGATTTGGATC 14104

jen AGGATATAGTTGCTGTTCATCATGGCTTGGGATTTGGATC 14120

cor AGGATATAGTTGCTGTTCATCATGGCTTGGGATTTGGATC 14120

man AGGATATAGTTGCTGTTCATCATGGCTTGGGATTTGGATC 14120

uni AGGATATAGTTGCTGTTCATCATGGCTTGGGATTTGGATC 14120

tall AGGATATAGTTGCTGTTCATCATGGCTTGGGATTTGGATC 14120

quil AGGATATAGTTGCTGTTCATCATGGCTTGGGATTTGGATC 14086

meri AGGATATAGTTGCTGTTCATCATGGCTTGGGATTTGGATC 14242

ref AGGATATAGTTGCTGTTCATCATGGCTTGGGATTTGGATC 14368

Consensus aggatatagttgctgttcatcatggcttgggatttggatc

kal CTTGGAGTTCATCAATTAATGAAGGTCTCAGTTTCTCTGT 14160

yor CTTGGAGTTCATCAATTAATGAAGGTCTCAGTTTCTCTGT 14144

jen CTTGGAGTTCATCAATTAATGAAGGTCTCAGTTTCTCTGT 14160

cor CTTGGAGTTCATCAATTAATGAAGGTCTCAGTTTCTCTGT 14160

man CTTGGAGTTCATCAATTAATGAAGGTCTCAGTTTCTCTGT 14160

uni CTTGGAGTTCATCAATTAATGAAGGTCTCAGTTTCTCTGT 14160

tall CTTGGAGTTCATCAATTAATGAAGGTCTCAGTTTCTCTGT 14160

quil CTTGGAGTTCATCAATTAATGAAGGTCTCAGTTTCTCTGT 14126

meri CTTGGAGTTCATCAATTAATGAAGGTCTCAGTTTCTCTGT 14282

ref CTTGGAGTTCATCAATTAATGAAGGTCTCAGTTTCTCTGT 14408

Consensus cttggagttcatcaattaatgaaggtctcagtttctctgt

kal ACACTCTCTCTGTGATTTATCTATGAGTTGTGGTATTCTT 14200

yor ACACTCTCTCTGTGATTTATCTATGAGTTGTGGTATTCTT 14184

jen ACACTCTCTCTGTGATTTATCTATGAGTTGTGGTATTCTT 14200

cor ACACTCTCTCTGTGATTTATCTATGAGTTGTGGTATTCTT 14200

man ACACTCTCTCTGTGATTTATCTATGAGTTGTGGTATTCTT 14200

uni ACACTCTCTCTGTGATTTATCTATGAGTTGTGGTATTCTT 14200

tall ACACTCTCTCTGTGATTTATCTATGAGTTGTGGTATTCTT 14200

quil ACACTCTCTCTGTGATTTATCTATGAGTTGTGGTATTCTT 14166

meri ACACTCTCTCTGTGATTTATCTATGAGTTGTGGTATTCTT 14322

ref ACACTCTCTCTGTGATTTATCTATGAGTTGTGGTATTCTT 14448

Consensus acactctctctgtgatttatctatgagttgtggtattctt

kal TGAAATCAGTATTTACTGAAGTTGTATTGATTTTGCATGT 14240

yor TGAAATCAGTATTTACTGAAGTTGTATTGATTTTGCATGT 14224

jen TGAAATCAGTATTTACTGAAGTTGTATTGATTTTGCATGT 14240

cor TGAAATCAGTATTTACTGAAGTTGTATTGATTTTGCATGT 14240

man TGAAATCAGTATTTACTGAAGTTGTATTGATTTTGCATGT 14240

uni TGAAATCAGTATTTACTGAAGTTGTATTGATTTTGCATGT 14240

tall TGAAATCAGTATTTACTGAAGTTGTATTGATTTTGCATGT 14240

quil TGAAATCAGTATTTACTGAAGTTGTATTGATTTTGCATGT 14206

meri TGAAATCAGTATTTACTGAAGTTGTATTGATTTTGCATGT 14362

ref TGAAATCAGTATTTACTGAAGTTGTATTGATTTTGCATGT 14488

Consensus tgaaatcagtatttactgaagttgtattgattttgcatgt

kal AATTTTCGGGTAATCAGTTGTTGCTAGTGATGATAGCTAC 14280

yor AATTTTCGGGTAATCAGTTGTTGCTAGTGATGATAGCTAC 14264

jen AATTTTCGGGTAATCAGTTGTTGCTAGTGATGATAGCTAC 14280

cor AATTTTCGGGTAATCAGTTGTTGCTAGTGATGATAGCTAC 14280

man AATTTTCGGGTAATCAGTTGTTGCTAGTGATGATAGCTAC 14280

uni AATTTTCGGGTAATCAGTTGTTGCTAGTGATGATAGCTAC 14280

tall AATTTTCGGGTAATCAGTTGTTGCTAGTGATGATAGCTAC 14280

quil AATTTTCGGGTAATCAGTTGTTGCTAGTGATGATAGCTAC 14246

meri AATTTTCGGGTAATCAGTTGTTGCTAGTGATGATAGCTAC 14402

ref AATTTTCGGGTAATCAGTTGTTGCTAGTGATGATAGCTAC 14528

Consensus aattttcgggtaatcagttgttgctagtgatgatagctac

kal TATGAAAAACCTGAGTGGCAATGTAGTCTCTACTTTGGCC 14320

yor TATGAAAAACCTGAGTGGCAATGTAGTCTCTACTTTGGCC 14304

jen TATGAAAAACCTGAGTGGCAATGTAGTCTCTACTTTGGCC 14320

cor TATGAAAAACCTGAGTGGCAATGTAGTCTCTACTTTGGCC 14320

man TATGAAAAACCTGAGTGGCAATGTAGTCTCTACTTTGGCC 14320

uni TATGAAAAACCTGAGTGGCAATGTAGTCTCTACTTTGGCC 14320

tall TATGAAAAACCTGAGTGGCAATGTAGTCTCTACTTTGGCC 14320

quil TATGAAAAACCTGAGTGGCAATGTAGTCTCTACTTTGGCC 14286

meri TATGAAAAACCTGAGTGGCAATGTAGTCTCTACTTTGGCC 14442

ref TATGAAAAACCTGAGTGGCAATGTAGTCTCTACTTTGGCC 14568

Consensus tatgaaaaacctgagtggcaatgtagtctctactttggcc

kal GTGAGCGTGGTACTTTTTCTTTTACTCCCTTGCCATGCAA 14360

yor GTGAGCGTGGTACTTTTTCTTTTACTCCCTTGCCATGCAA 14344

jen GTGAGCGTGGTACTTTTTCTTTTACTCCCTTGCCATGCAA 14360

cor GTGAGCGTGGTACTTTTTCTTTTACTCCCTTGCCATGCAA 14360

man GTGAGCGTGGTACTTTTTCTTTTACTCCCTTGCCATGCAA 14360

uni GTGAGCGTGGTACTTTTTCTTTTACTCCCTTGCCATGCAA 14360

tall GTGAGCGTGGTACTTTTTCTTTTACTCCCTTGCCATGCAA 14360

quil GTGAGCGTGGTACTTTTTCTTTTACTCCCTTGCCATGCAA 14326

meri GTGAGCGTGGTACTTTTTCTTTTACTCCCTTGCCATGCAA 14482

ref GTGAGCGTGGTACTTTTTCTTTTACTCCCTTGCCATGCAA 14608

Consensus gtgagcgtggtactttttcttttactcccttgccatgcaa

kal TTTTATGGTCTTTCTTTGAGTGTATCAATGTTACTACCCT 14400

yor TTTTATGGTCTTTCTTTGAGTGTATCAATGTTACTACCCT 14384

jen TTTTATGGTCTTTCTTTGAGTGTATCAATGTTACTACCCT 14400

cor TTTTATGGTCTTTCTTTGAGTGTATCAATGTTACTACCCT 14400

man TTTTATGGTCTTTCTTTGAGTGTATCAATGTTACTACCCT 14400

uni TTTTATGGTCTTTCTTTGAGTGTATCAATGTTACTACCCT 14400

tall TTTTATGGTCTTTCTTTGAGTGTATCAATGTTACTACCCT 14400

quil TTTTATGGTCTTTCTTTGAGTGTATCAATGTTACTACCCT 14366

meri TTTTATGGTCTTTCTTTGAGTGTATCAATGTTACTACCCT 14522

ref TTTTATGGTCTTTCTTTGAGTGTATCAATGTTACTACCCT 14648

Consensus ttttatggtctttctttgagtgtatcaatgttactaccct

kal GTATTTACTCGGGCTTTCTTTTAAAATGGGTAGATGTGAT 14440

yor GTATTTACTCGGGCTTTCTTTTAAAATGGGTAGATGTGAT 14424

jen GTATTTACTCGGGCTTTCTTTTAAAATGGGTAGATGTGAT 14440

cor GTATTTACTCGGGCTTTCTTTTAAAATGGGTAGATGTGAT 14440

man GTATTTACTCGGGCTTTCTTTTAAAATGGGTAGATGTGAT 14440

uni GTATTTACTCGGGCTTTCTTTTAAAATGGGTAGATGTGAT 14440

tall GTATTTACTCGGGCTTTCTTTTAAAATGGGTAGATGTGAT 14440

quil GTATTTACTCGGGCTTTCTTTTAAAATGGGTAGATGTGAT 14406

meri GTATTTACTCGGGCTTTCTTTTAAAATGGGTAGATGTGAT 14562

ref GTATTTACTCGGGCTTTCTTTTAAAATGGGTAGATGTGAT 14688

Consensus gtatttactcgggctttcttttaaaatgggtagatgtgat

kal AGAGGAAGATATCTTGAATGAGGAATCTTGTATTCACGTC 14480

yor AGAGGAAGATATCTTGAATGAGGAATCTTGTATTCACGTC 14464

jen AGAGGAAGATATCTTGAATGAGGAATCTTGTATTCACGTC 14480

cor AGAGGAAGATATCTTGAATGAGGAATCTTGTATTCACGTC 14480

man AGAGGAAGATATCTTGAATGAGGAATCTTGTATTCACGTC 14480

uni AGAGGAAGATATCTTGAATGAGGAATCTTGTATTCACGTC 14480

tall AGAGGAAGATATCTTGAATGAGGAATCTTGTATTCACGTC 14480

quil AGAGGAAGATATCTTGAATGAGGAATCTTGTATTCACGTC 14446

meri AGAGGAAGATATCTTGAATGAGGAATCTTGTATTCACGTC 14602

ref AGAGGAAGATATCTTGAATGAGGAATCTTGTATTCACGTC 14728

Consensus agaggaagatatcttgaatgaggaatcttgtattcacgtc

kal TTGAAAATTTTGATCACCAAGGCTGATACTGAAATCGAGG 14520

yor TTGAAAATTTTGATCACCAAGGCTGATACTGAAATCGAGG 14504

jen TTGAAAATTTTGATCACCAAGGCTGATACTGAAATCGAGG 14520

cor TTGAAAATTTTGATCACCAAGGCTGATACTGAAATCGAGG 14520

man TTGAAAATTTTGATCACCAAGGCTGATACTGAAATCGAGG 14520

uni TTGAAAATTTTGATCACCAAGGCTGATACTGAAATCGAGG 14520

tall TTGAAAATTTTGATCACCAAGGCTGATACTGAAATCGAGG 14520

quil TTGAAAATTTTGATCACCAAGGCTGATACTGAAATCGAGG 14486

meri TTGAAAATTTTGATCACCAAGGCTGATACTGAAATCGAGG 14642

ref TTGAAAATTTTGATCACCAAGGCTGATACTGAAATCGAGG 14768

Consensus ttgaaaattttgatcaccaaggctgatactgaaatcgagg

kal GACTAGAGAAGGATCTCACGTCCCTTCAGAATGAATTGGC 14560

yor GACTAGAGAAGGATCTCACGTCCCTTCAGAATGAATTGGC 14544

jen GACTAGAGAAGGATCTCACGTCCCTTCAGAATGAATTGGC 14560

cor GACTAGAGAAGGATCTCACGTCCCTTCAGAATGAATTGGC 14560

man GACTAGAGAAGGATCTCACGTCCCTTCAGAATGAATTGGC 14560

uni GACTAGAGAAGGATCTCACGTCCCTTCAGAATGAATTGGC 14560

tall GACTAGAGAAGGATCTCACGTCCCTTCAGAATGAATTGGC 14560

quil GACTAGAGAAGGATCTCACGTCCCTTCAGAATGAATTGGC 14526

meri GACTAGAGAAGGATCTCACGTCCCTTCAGAATGAATTGGC 14682

ref GACTAGAGAAGGATCTCACGTCCCTTCAGAATGAATTGGC 14808

Consensus gactagagaaggatctcacgtcccttcagaatgaattggc

kal CTGGGCTGAGTATGAAAAATGGCCTGAAATATGCTGTGGT 14600

yor CTGGGCTGAGTATGAAAAATGGCCTGAAATATGCTGTGGT 14584

jen CTGGGCTGAGTATGAAAAATGGCCTGAAATATGCTGTGGT 14600

cor CTGGGCTGAGTATGAAAAATGGCCTGAAATATGCTGTGGT 14600

man CTGGGCTGAGTATGAAAAATGGCCTGAAATATGCTGTGGT 14600

uni CTGGGCTGAGTATGAAAAATGGCCTGAAATATGCTGTGGT 14600

tall CTGGGCTGAGTATGAAAAATGGCCTGAAATATGCTGTGGT 14600

quil CTGGGCTGAGTATGAAAAATGGCCTGAAATATGCTGTGGT 14566

meri CTGGGCTGAGTATGAAAAATGGCCTGAAATATGCTGTGGT 14722

ref CTGGGCTGAGTATGAAAAATGGCCTGAAATATGCTGTGGT 14848

Consensus ctgggctgagtatgaaaaatggcctgaaatatgctgtggt

kal ACTTTGACTGAAAGAATCAAATTGCTTGATGTTGCTATTA 14640

yor ACTTTGACTGAAAGAATCAAATTGCTTGATGTTGCTATTA 14624

jen ACTTTGACTGAAAGAATCAAATTGCTTGATGTTGCTATTA 14640

cor ACTTTGACTGAAAGAATCAAATTGCTTGATGTTGCTATTA 14640

man ACTTTGACTGAAAGAATCAAATTGCTTGATGTTGCTATTA 14640

uni ACTTTGACTGAAAGAATCAAATTGCTTGATGTTGCTATTA 14640

tall ACTTTGACTGAAAGAATCAAATTGCTTGATGTTGCTATTA 14640

quil ACTTTGACTGAAAGAATCAAATTGCTTGATGTTGCTATTA 14606

meri ACTTTGACTGAAAGAATCAAATTGCTTGATGTTGCTATTA 14762

ref ACTTTGACTGAAAGAATCAAATTGCTTGATGTTGCTATTA 14888

Consensus actttgactgaaagaatcaaattgcttgatgttgctatta

kal ACACCTTGAAGAAGAATGATCATGCCAATGATACAGAGGT 14680

yor ACACCTTGAAGAAGAATGATCATGCCAATGATACAGAGGT 14664

jen ACACCTTGAAGAAGAATGATCATGCCAATGATACAGAGGT 14680

cor ACACCTTGAAGAAGAATGATCATGCCAATGATACAGAGGT 14680

man ACACCTTGAAGAAGAATGATCATGCCAATGATACAGAGGT 14680

uni ACACCTTGAAGAAGAATGATCATGCCAATGATACAGAGGT 14680

tall ACACCTTGAAGAAGAATGATCATGCCAATGATACAGAGGT 14680

quil ACACCTTGAAGAAGAATGATCATGCCAATGATACAGAGGT 14646

meri ACACCTTGAAGAAGAATGATCATGCCAATGATACAGAGGT 14802

ref ACACCTTGAAGAAGAATGATCATGCCAATGATACAGAGGT 14928

Consensus acaccttgaagaagaatgatcatgccaatgatacagaggt

kal ACAGTTGCTGCTAGACAAACCTGCTGAGACAGTTCATGAA 14720

yor ACAGTTGCTGCTAGACAAACCTGCTGAGACAGTTCATGAA 14704

jen ACAGTTGCTGCTAGACAAACCTGCTGAGACAGTTCATGAA 14720

cor ACAGTTGCTGCTAGACAAACCTGCTGAGACAGTTCATGAA 14720

man ACAGTTGCTGCTAGACAAACCTGCTGAGACAGTTCATGAA 14720

uni ACAGTTGCTGCTAGACAAACCTGCTGAGACAGTTCATGAA 14720

tall ACAGTTGCTGCTAGACAAACCTGCTGAGACAGTTCATGAA 14720

quil ACAGTTGCTGCTAGACAAACCTGCTGAGACAGTTCATGAA 14686

meri ACAGTTGCTGCTAGACAAACCTGCTGAGACAGTTCATGAA 14842

ref ACAGTTGCTGCTAGACAAACCTGCTGAGACAGTTCATGAA 14968

Consensus acagttgctgctagacaaacctgctgagacagttcatgaa

kal ATTGTTGAGGCTTTGCATAGAGATCACTTTGAAGATATAC 14760

yor ATTGTTGAGGCTTTGCATAGAGATCACTTTGAAGATATAC 14744

jen ATTGTTGAGGCTTTGCATAGAGATCACTTTGAAGATATAC 14760

cor ATTGTTGAGGCTTTGCATAGAGATCACTTTGAAGATATAC 14760

man ATTGTTGAGGCTTTGCATAGAGATCACTTTGAAGATATAC 14760

uni ATTGTTGAGGCTTTGCATAGAGATCACTTTGAAGATATAC 14760

tall ATTGTTGAGGCTTTGCATAGAGATCACTTTGAAGATATAC 14760

quil ATTGTTGAGGCTTTGCATAGAGATCACTTTGAAGATATAC 14726

meri ATTGTTGAGGCTTTGCATAGAGATCACTTTGAAGATATAC 14882

ref ATTGTTGAGGCTTTGCATAGAGATCACTTTGAAGATATAC 15008

Consensus attgttgaggctttgcatagagatcactttgaagatatac

kal ATGGCCAGGTTTGACTTTTAGAATCCCTTCCTCTCCCCAA 14800

yor ATGGCCAGGTTTGACTTTTAGAATCCCTTCCTCTCCCCAA 14784

jen ATGGCCAGGTTTGACTTTTAGAATCCCTTCCTCTCCCCAA 14800

cor ATGGCCAGGTTTGACTTTTAGAATCCCTTCCTCTCCCCAA 14800

man ATGGCCAGGTTTGACTTTTAGAATCCCTTCCTCTCCCCAA 14800

uni ATGGCCAGGTTTGACTTTTAGAATCCCTTCCTCTCCCCAA 14800

tall ATGGCCAGGTTTGACTTTTAGAATCCCTTCCTCTCCCCAA 14800

quil ATGGCCAGGTTTGACTTTTAGAATCCCTTCCTCTCCCCAA 14766

meri ATGGCCAGGTTTGACTTTTAGAATCCCTTCCTCTCCCCAA 14922

ref ATGGCCAGGTTTGACTTTTAGAATCCCTTCCTCTCCCCAA 15048

Consensus atggccaggtttgacttttagaatcccttcctctccccaa

kal GTTCATGAACTTTAATTGTTTTCAGTTAATAGATTGAAAA 14840

yor GTTCATGAACTTTAATTGTTTTCAGTTAATAGATTGAAAA 14824

jen GTTCATGAACTTTAATTGTTTTCAGTTAATAGATTGAAAA 14840

cor GTTCATGAACTTTAATTGTTTTCAGTTAATAGATTGAAAA 14840

man GTTCATGAACTTTAATTGTTTTCAGTTAATAGATTGAAAA 14840

uni GTTCATGAACTTTAATTGTTTTCAGTTAATAGATTGAAAA 14840

tall GTTCATGAACTTTAATTGTTTTCAGTTAATAGATTGAAAA 14840

quil GTTCATGAACTTTAATTGTTTTCAGTTAATAGATTGAAAA 14806

meri GTTCATGAACTTTAATTGTTTTCAGTTAATAGATTGAAAA 14962

ref GTTCATGAACTTTAATTGTTTTCAGTTAATAGATTGAAAA 15088

Consensus gttcatgaactttaattgttttcagttaatagattgaaaa

kal GAAGATGCTGCATTTATCCTTTTCACCATGCAAACTCTGT 14880

yor GAAGATGCTGCATTTATCCTTTTCACCATGCAAACTCTGT 14864

jen GAAGATGCTGCATTTATCCTTTTCACCATGCAAACTCTGT 14880

cor GAAGATGCTGCATTTATCCTTTTCACCATGCAAACTCTGT 14880

man GAAGATGCTGCATTTATCCTTTTCACCATGCAAACTCTGT 14880

uni GAAGATGCTGCATTTATCCTTTTCACCATGCAAACTCTGT 14880

tall GAAGATGCTGCATTTATCCTTTTCACCATGCAAACTCTGT 14880

quil GAAGATGCTGCATTTATCCTTTTCACCATGCAAACTCTGT 14846

meri GAAGATGCTGCATTTATCCTTTTCACCATGCAAACTCTGT 15002

ref GAAGATGCTGCATTTATCCTTTTCACCATGCAAACTCTGT 15128

Consensus gaagatgctgcatttatccttttcaccatgcaaactctgt

kal ATTTGCCAACTATTCTAGAGTTTAAATTTTTTGAGACTGA 14920

yor ATTTGCCAACTATTCTAGAGTTTAAATTTTTTGAGACTGA 14904

jen ATTTGCCAACTATTCTAGAGTTTAAATTTTTTGAGACTGA 14920

cor ATTTGCCAACTATTCTAGAGTTTAAATTTTTTGAGACTGA 14920

man ATTTGCCAACTATTCTAGAGTTTAAATTTTTTGAGACTGA 14920

uni ATTTGCCAACTATTCTAGAGTTTAAATTTTTTGAGACTGA 14920

tall ATTTGCCAACTATTCTAGAGTTTAAATTTTTTGAGACTGA 14920

quil ATTTGCCAACTATTCTAGAGTTTAAATTTTTTGAGACTGA 14886

meri ATTTGCCAACTATTCTAGAGTTTAAATTTTTTGAGACTGA 15042

ref ATTTGCCAACTATTCTAGAGTTTAAATTTTTTGAGACTGA 15168

Consensus atttgccaactattctagagtttaaattttttgagactga

kal GACCTTACTTACTCTAGACTTCGACTACTTGGTAGACTCG 14960

yor GACCTTACTTACTCTAGACTTCGACTACTTGGTAGACTCG 14944

jen GACCTTACTTACTCTAGACTTCGACTACTTGGTAGACTCG 14960

cor GACCTTACTTACTCTAGACTTCGACTACTTGGTAGACTCG 14960

man GACCTTACTTACTCTAGACTTCGACTACTTGGTAGACTCG 14960

uni GACCTTACTTACTCTAGACTTCGACTACTTGGTAGACTCG 14960

tall GACCTTACTTACTCTAGACTTCGACTACTTGGTAGACTCG 14960

quil GACCTTACTTACTCTAGACTTCGACTACTTGGTAGACTCG 14926

meri GACCTTACTTACTCTAGACTTCGACTACTTGGTAGACTCG 15082

ref GACCTTACTTACTCTAGACTTCGACTACTTGGTAGACTCG 15208

Consensus gaccttacttactctagacttcgactacttggtagactcg

kal ATGAAAGGGGCAAAGCCTGCCCAATGTCTATTTTTTATAT 15000

yor ATGAAAGGGGCAAAGCCTGCCCAATGTCTATTTTTTATAT 14984

jen ATGAAAGGGGCAAAGCCTGCCCAATGTCTATTTTTTATAT 15000

cor ATGAAAGGGGCAAAGCCTGCCCAATGTCTATTTTTTATAT 15000

man ATGAAAGGGGCAAAGCCTGCCCAATGTCTATTTTTTATAT 15000

uni ATGAAAGGGGCAAAGCCTGCCCAATGTCTATTTTTTATAT 15000

tall ATGAAAGGGGCAAAGCCTGCCCAATGTCTATTTTTTATAT 15000

quil ATGAAAGGGGCAAAGCCTGCCCAATGTCTATTTTTTATAT 14966

meri ATGAAAGGGGCAAAGCCTGCCCAATGTCTATTTTTTATAT 15122

ref ATGAAAGGGGCAAAGCCTGCCCAATGTCTATTTTTTATAT 15248

Consensus atgaaaggggcaaagcctgcccaatgtctattttttatat

kal TTCACCAGAGTCGAACATGAAACCTTGCTTAAGAGGAATT 15040

yor TTCACCAGAGTCGAACATGAAACCTTGCTTAAGAGGAATT 15024

jen TTCACCAGAGTCGAACATGAAACCTTGCTTAAGAGGAATT 15040

cor TTCACCAGAGTCGAACATGAAACCTTGCTTAAGAGGAATT 15040

man TTCACCAGAGTCGAACATGAAACCTTGCTTAAGAGGAATT 15040

uni TTCACCAGAGTCGAACATGAAACCTTGCTTAAGAGGAATT 15040

tall TTCACCAGAGTCGAACATGAAACCTTGCTTAAGAGGAATT 15040

quil TTCACCAGAGTCGAACATGAAACCTTGCTTAAGAGGAATT 15006

meri TTCACCAGAGTCGAACATGAAACCTTGCTTAAGAGGAATT 15162

ref TTCACCAGAGTCGAACATGAAACCTTGCTTAAGAGGAATT 15288

Consensus ttcaccagagtcgaacatgaaaccttgcttaagaggaatt

kal TAACTTCTTACCAGTTCGGCTAACACATTGGTCCGTCCTA 15080

yor TAACTTCTTACCAGTTCGGCTAACACATTGGTCCGTCCTA 15064

jen TAACTTCTTACCAGTTCGGCTAACACATTGGTCCGTCCTA 15080

cor TAACTTCTTACCAGTTCGGCTAACACATTGGTCCGTCCTA 15080

man TAACTTCTTACCAGTTCGGCTAACACATTGGTCCGTCCTA 15080

uni TAACTTCTTACCAGTTCGGCTAACACATTGGTCCGTCCTA 15080

tall TAACTTCTTACCAGTTCGGCTAACACATTGGTCCGTCCTA 15080

quil TAACTTCTTACCAGTTCGGCTAACACATTGGTCCGTCCTA 15046

meri TAACTTCTTACCAGTTCGGCTAACACATTGGTCCGTCCTA 15202

ref TAACTTCTTACCAGTTCGGCTAACACATTGGTCCGTCCTA 15328

Consensus taacttcttaccagttcggctaacacattggtccgtccta

kal GGGCTTCCTATCTTTGATTCACAATATTTTTTTAAACCCC 15120

yor GGGCTTCCTATCTTTGATTCACAATATTTTTTTAAACCCC 15104

jen GGGCTTCCTATCTTTGATTCACAATATTTTTTTAAACCCC 15120

cor GGGCTTCCTATCTTTGATTCACAATATTTTTTTAAACCCC 15120

man GGGCTTCCTATCTTTGATTCACAATATTTTTTTAAACCCC 15120

uni GGGCTTCCTATCTTTGATTCACAATATTTTTTTAAACCCC 15120

tall GGGCTTCCTATCTTTGATTCACAATATTTTTTTAAACCCC 15120

quil GGGCTTCCTATCTTTGATTCACAATATTTTTTTAAACCCC 15086

meri GGGCTTCCTATCTTTGATTCACAATATTTTTTTAAACCCC 15242

ref GGGCTTCCTATCTTTGATTCACAATATTTTTTTAAACCCC 15368

Consensus gggcttcctatctttgattcacaatatttttttaaacccc

kal ACAAAATGATGCTGTTCCATAATTTTGTATTGCTTTGGGT 15160

yor ACAAAATGATGCTGTTCCATAATTTTGTATTGCTTTGGGT 15144

jen ACAAAATGATGCTGTTCCATAATTTTGTATTGCTTTGGGT 15160

cor ACAAAATGATGCTGTTCCATAATTTTGTATTGCTTTGGGT 15160

man ACAAAATGATGCTGTTCCATAATTTTGTATTGCTTTGGGT 15160

uni ACAAAATGATGCTGTTCCATAATTTTGTATTGCTTTGGGT 15160

tall ACAAAATGATGCTGTTCCATAATTTTGTATTGCTTTGGGT 15160

quil ACAAAATGATGCTGTTCCATAATTTTGTATTGCTTTGGGT 15126

meri ACAAAATGATGCTGTTCCATAATTTTGTATTGCTTTGGGT 15282

ref ACAAAATGATGCTGTTCCATAATTTTGTATTGCTTTGGGT 15408

Consensus acaaaatgatgctgttccataattttgtattgctttgggt

kal AGCAAAAAGCTTTATTATGACAGTTTTTCCTTTAAAATTA 15200

yor AGCAAAAAGCTTTATTATGACAGTTTTTCCTTTAAAATTA 15184

jen AGCAAAAAGCTTTATTATGACAGTTTTTCCTTTAAAATTA 15200

cor AGCAAAAAGCTTTATTATGACAGTTTTTCCTTTAAAATTA 15200

man AGCAAAAAGCTTTATTATGACAGTTTTTCCTTTAAAATTA 15200

uni AGCAAAAAGCTTTATTATGACAGTTTTTCCTTTAAAATTA 15200

tall AGCAAAAAGCTTTATTATGACAGTTTTTCCTTTAAAATTA 15200

quil AGCAAAAAGCTTTATTATGACAGTTTTTCCTTTAAAATTA 15166

meri AGCAAAAAGCTTTATTATGACAGTTTTTCCTTTAAAATTA 15322

ref AGCAAAAAGCTTTATTATGACAGTTTTTCCTTTAAAATTA 15448

Consensus agcaaaaagctttattatgacagtttttcctttaaaatta

kal TGTAGCTCAGGATGTGACGAGAGAATGCTAAAAGTGCCTT 15240

yor TGTAGCTCAGGATGTGACGAGAGAATGCTAAAAGTGCCTT 15224

jen TGTAGCTCAGGATGTGACGAGAGAATGCTAAAAGTGCCTT 15240

cor TGTAGCTCAGGATGTGACGAGAGAATGCTAAAAGTGCCTT 15240

man TGTAGCTCAGGATGTGACGAGAGAATGCTAAAAGTGCCTT 15240

uni TGTAGCTCAGGATGTGACGAGAGAATGCTAAAAGTGCCTT 15240

tall TGTAGCTCAGGATGTGACGAGAGAATGCTAAAAGTGCCTT 15240

quil TGTAGCTCAGGATGTGACGAGAGAATGCTAAAAGTGCCTT 15206

meri TGTAGCTCAGGATGTGACGAGAGAATGCTAAAAGTGCCTT 15362

ref TGTAGCTCAGGATGTGACGAGAGAATGCTAAAAGTGCCTT 15488

Consensus tgtagctcaggatgtgacgagagaatgctaaaagtgcctt

kal TGATACTAGGGCTGAACTGGACATTGTATGCTTAGCACAA 15280

yor TGATACTAGGGCTGAACTGGACATTGTATGCTTAGCACAA 15264

jen TGATACTAGGGCTGAACTGGACATTGTATGCTTAGCACAA 15280

cor TGATACTAGGGCTGAACTGGACATTGTATGCTTAGCACAA 15280

man TGATACTAGGGCTGAACTGGACATTGTATGCTTAGCACAA 15280

uni TGATACTAGGGCTGAACTGGACATTGTATGCTTAGCACAA 15280

tall TGATACTAGGGCTGAACTGGACATTGTATGCTTAGCACAA 15280

quil TGATACTAGGGCTGAACTGGACATTGTATGCTTAGCACAA 15246

meri TGATACTAGGGCTGAACTGGACATTGTATGCTTAGCACAA 15402

ref TGATACTAGGGCTGAACTGGACATTGTATGCTTAGCACAA 15528

Consensus tgatactagggctgaactggacattgtatgcttagcacaa

kal GAGGTCCCCTCTCCTACAAGTAATAAATAAAAGGCTTAAT 15320

yor GAGGTCCCCTCTCCTACAAGTAATAAATAAAAGGCTTAAT 15304

jen GAGGTCCCCTCTCCTACAAGTAATAAATAAAAGGCTTAAT 15320

cor GAGGTCCCCTCTCCTACAAGTAATAAATAAAAGGCTTAAT 15320

man GAGGTCCCCTCTCCTACAAGTAATAAATAAAAGGCTTAAT 15320

uni GAGGTCCCCTCTCCTACAAGTAATAAATAAAAGGCTTAAT 15320

tall GAGGTCCCCTCTCCTACAAGTAATAAATAAAAGGCTTAAT 15320

quil GAGGTCCCCTCTCCTACAAGTAATAAATAAAAGGCTTAAT 15286

meri GAGGTCCCCTCTCCTACAAGTAATAAATAAAAGGCTTAAT 15442

ref GAGGTCCCCTCTCCTACAAGTAATAAATAAAAGGCTTAAT 15568

Consensus gaggtcccctctcctacaagtaataaataaaaggcttaat

kal TTCACTATAGCCTCTCTGGAATCTAGATAGCTAGTGTATC 15360

yor TTCACTATAGCCTCTCTGGAATCTAGATAGCTAGTGTATC 15344

jen TTCACTATAGCCTCTCTGGAATCTAGATAGCTAGTGTATC 15360

cor TTCACTATAGCCTCTCTGGAATCTAGATAGCTAGTGTATC 15360

man TTCACTATAGCCTCTCTGGAATCTAGATAGCTAGTGTATC 15360

uni TTCACTATAGCCTCTCTGGAATCTAGATAGCTAGTGTATC 15360

tall TTCACTATAGCCTCTCTGGAATCTAGATAGCTAGTGTATC 15360

quil TTCACTATAGCCTCTCTGGAATCTAGATAGCTAGTGTATC 15326

meri TTCACTATAGCCTCTCTGGAATCTAGATAGCTAGTGTATC 15482

ref TTCACTATAGCCTCTCTGGAATCTAGATAGCTAGTGTATC 15608

Consensus ttcactatagcctctctggaatctagatagctagtgtatc

kal AGAGCAGTTTAGTGTGAAGCAACTTAGTCCCTTCACTTTA 15400

yor AGAGCAGTTTAGTGTGAAGCAACTTAGTCCCTTCACTTTA 15384

jen AGAGCAGTTTAGTGTGAAGCAACTTAGTCCCTTCACTTTA 15400

cor AGAGCAGTTTAGTGTGAAGCAACTTAGTCCCTTCACTTTA 15400

man AGAGCAGTTTAGTGTGAAGCAACTTAGTCCCTTCACTTTA 15400

uni AGAGCAGTTTAGTGTGAAGCAACTTAGTCCCTTCACTTTA 15400

tall AGAGCAGTTTAGTGTGAAGCAACTTAGTCCCTTCACTTTA 15400

quil AGAGCAGTTTAGTGTGAAGCAACTTAGTCCCTTCACTTTA 15366

meri AGAGCAGTTTAGTGTGAAGCAACTTAGTCCCTTCACTTTA 15522

ref AGAGCAGTTTAGTGTGAAGCAACTTAGTCCCTTCACTTTA 15648

Consensus agagcagtttagtgtgaagcaacttagtcccttcacttta

kal TTTTTCTAATGTTTGGAGAAATTCACTCCTTACAGATAAT 15440

yor TTTTTCTAATGTTTGGAGAAATTCACTCCTTACAGATAAT 15424

jen TTTTTCTAATGTTTGGAGAAATTCACTCCTTACAGATAAT 15440

cor TTTTTCTAATGTTTGGAGAAATTCACTCCTTACAGATAAT 15440

man TTTTTCTAATGTTTGGAGAAATTCACTCCTTACAGATAAT 15440

uni TTTTTCTAATGTTTGGAGAAATTCACTCCTTACAGATAAT 15440

tall TTTTTCTAATGTTTGGAGAAATTCACTCCTTACAGATAAT 15440

quil TTTTTCTAATGTTTGGAGAAATTCACTCCTTACAGATAAT 15406

meri TTTTTCTAATGTTTGGAGAAATTCACTCCTTACAGATAAT 15562

ref TTTTTCTAATGTTTGGAGAAATTCACTCCTTACAGATAAT 15688

Consensus tttttctaatgtttggagaaattcactccttacagataat

kal ATTTGGTTTTATCTATTCTACATGAATAAGCTTCAGCATG 15480

yor ATTTGGTTTTATCTATTCTACATGAATAAGCTTCAGCATG 15464

jen ATTTGGTTTTATCTATTCTACATGAATAAGCTTCAGCATG 15480

cor ATTTGGTTTTATCTATTCTACATGAATAAGCTTCAGCATG 15480

man ATTTGGTTTTATCTATTCTACATGAATAAGCTTCAGCATG 15480

uni ATTTGGTTTTATCTATTCTACATGAATAAGCTTCAGCATG 15480

tall ATTTGGTTTTATCTATTCTACATGAATAAGCTTCAGCATG 15480

quil ATTTGGTTTTATCTATTCTACATGAATAAGCTTCAGCATG 15446

meri ATTTGGTTTTATCTATTCTACATGAATAAGCTTCAGCATG 15602

ref ATTTGGTTTTATCTATTCTACATGAATAAGCTTCAGCATG 15728

Consensus atttggttttatctattctacatgaataagcttcagcatg

kal TTTTTCCTGTATCAGACTTTAGTTAAGTTGATGCATTAAA 15520

yor TTTTTCCTGTATCAGACTTTAGTTAAGTTGATGCATTAAA 15504

jen TTTTTCCTGTATCAGACTTTAGTTAAGTTGATGCATTAAA 15520

cor TTTTTCCTGTATCAGACTTTAGTTAAGTTGATGCATTAAA 15520

man TTTTTCCTGTATCAGACTTTAGTTAAGTTGATGCATTAAA 15520

uni TTTTTCCTGTATCAGACTTTAGTTAAGTTGATGCATTAAA 15520

tall TTTTTCCTGTATCAGACTTTAGTTAAGTTGATGCATTAAA 15520

quil TTTTTCCTGTATCAGACTTTAGTTAAGTTGATGCATTAAA 15486

meri TTTTTCCTGTATCAGACTTTAGTTAAGTTGATGCATTAAA 15642

ref TTTTTCCTGTATCAGACTTTAGTTAAGTTGATGCATTAAA 15768

Consensus tttttcctgtatcagactttagttaagttgatgcattaaa

kal TCTAATTGGTTTCTTAGAATGTAATCAATGATAGAAGCTT 15560

yor TCTAATTGGTTTCTTAGAATGTAATCAATGATAGAAGCTT 15544

jen TCTAATTGGTTTCTTAGAATGTAATCAATGATAGAAGCTT 15560

cor TCTAATTGGTTTCTTAGAATGTAATCAATGATAGAAGCTT 15560

man TCTAATTGGTTTCTTAGAATGTAATCAATGATAGAAGCTT 15560

uni TCTAATTGGTTTCTTAGAATGTAATCAATGATAGAAGCTT 15560

tall TCTAATTGGTTTCTTAGAATGTAATCAATGATAGAAGCTT 15560

quil TCTAATTGGTTTCTTAGAATGTAATCAATGATAGAAGCTT 15526

meri TCTAATTGGTTTCTTAGAATGTAATCAATGATAGAAGCTT 15682

ref TCTAATTGGTTTCTTAGAATGTAATCAATGATAGAAGCTT 15808

Consensus tctaattggtttcttagaatgtaatcaatgatagaagctt

kal CAGAGTCCTTATTAACGTGGATGAAAATGAAGAATCCATC 15600

yor CAGAGTCCTTATTAACGTGGATGAAAATGAAGAATCCATC 15584

jen CAGAGTCCTTATTAACGTGGATGAAAATGAAGAATCCATC 15600

cor CAGAGTCCTTATTAACGTGGATGAAAATGAAGAATCCATC 15600

man CAGAGTCCTTATTAACGTGGATGAAAATGAAGAATCCATC 15600

uni CAGAGTCCTTATTAACGTGGATGAAAATGAAGAATCCATC 15600

tall CAGAGTCCTTATTAACGTGGATGAAAATGAAGAATCCATC 15600

quil CAGAGTCCTTATTAACGTGGATGAAAATGAAGAATCCATC 15566

meri CAGAGTCCTTATTAACGTGGATGAAAATGAAGAATCCATC 15722

ref CAGAGTCCTTATTAACGTGGATGAAAATGAAGAATCCATC 15848

Consensus cagagtccttattaacgtggatgaaaatgaagaatccatc

kal AGTCCAGTATCAATTCAGGTGGAAAATGAACTTAACATGA 15640

yor AGTCCAGTATCAATTCAGGTGGAAAATGAACTTAACATGA 15624

jen AGTCCAGTATCAATTCAGGTGGAAAATGAACTTAACATGA 15640

cor AGTCCAGTATCAATTCAGGTGGAAAATGAACTTAACATGA 15640

man AGTCCAGTATCAATTCAGGTGGAAAATGAACTTAACATGA 15640

uni AGTCCAGTATCAATTCAGGTGGAAAATGAACTTAACATGA 15640

tall AGTCCAGTATCAATTCAGGTGGAAAATGAACTTAACATGA 15640

quil AGTCCAGTATCAATTCAGGTGGAAAATGAACTTAACATGA 15606

meri AGTCCAGTATCAATTCAGGTGGAAAATGAACTTAACATGA 15762

ref AGTCCAGTATCAATTCAGGTGGAAAATGAACTTAACATGA 15888

Consensus agtccagtatcaattcaggtggaaaatgaacttaacatga

kal ATTAGGAGCTGCAAGAGAATAAGGTAGAAGAAAGATTGGA 15680

yor ATTAGGAGCTGCAAGAGAATAAGGTAGAAGAAAGATTGGA 15664

jen ATTAGGAGCTGCAAGAGAATAAGGTAGAAGAAAGATTGGA 15680

cor ATTAGGAGCTGCAAGAGAATAAGGTAGAAGAAAGATTGGA 15680

man ATTAGGAGCTGCAAGAGAATAAGGTAGAAGAAAGATTGGA 15680

uni ATTAGGAGCTGCAAGAGAATAAGGTAGAAGAAAGATTGGA 15680

tall ATTAGGAGCTGCAAGAGAATAAGGTAGAAGAAAGATTGGA 15680

quil ATTAGGAGCTGCAAGAGAATAAGGTAGAAGAAAGATTGGA 15646

meri ATTAGGAGCTGCAAGAGAATAAGGTAGAAGAAAGATTGGA 15802

ref ATTAGGAGCTGCAAGAGAATAAGGTAGAAGAAAGATTGGA 15928

Consensus attaggagctgcaagagaataaggtagaagaaagattgga

kal AATAGTTGTCTCTTAGGAAGCACAAAGCACCAGTGTATAT 15720

yor AATAGTTGTCTCTTAGGAAGCACAAAGCACCAGTGTATAT 15704

jen AATAGTTGTCTCTTAGGAAGCACAAAGCACCAGTGTATAT 15720

cor AATAGTTGTCTCTTAGGAAGCACAAAGCACCAGTGTATAT 15720

man AATAGTTGTCTCTTAGGAAGCACAAAGCACCAGTGTATAT 15720

uni AATAGTTGTCTCTTAGGAAGCACAAAGCACCAGTGTATAT 15720

tall AATAGTTGTCTCTTAGGAAGCACAAAGCACCAGTGTATAT 15720

quil AATAGTTGTCTCTTAGGAAGCACAAAGCACCAGTGTATAT 15686

meri AATAGTTGTCTCTTAGGAAGCACAAAGCACCAGTGTATAT 15842

ref AATAGTTGTCTCTTAGGAAGCACAAAGCACCAGTGTATAT 15968

Consensus aatagttgtctcttaggaagcacaaagcaccagtgtatat

kal GTCTTAATTTGTATTACTTGATACTTGCACATCAATGTTG 15760

yor GTCTTAATTTGTATTACTTGATACTTGCACATCAATGTTG 15744

jen GTCTTAATTTGTATTACTTGATACTTGCACATCAATGTTG 15760

cor GTCTTAATTTGTATTACTTGATACTTGCACATCAATGTTG 15760

man GTCTTAATTTGTATTACTTGATACTTGCACATCAATGTTG 15760

uni GTCTTAATTTGTATTACTTGATACTTGCACATCAATGTTG 15760

tall GTCTTAATTTGTATTACTTGATACTTGCACATCAATGTTG 15760

quil GTCTTAATTTGTATTACTTGATACTTGCACATCAATGTTG 15726

meri GTCTTAATTTGTATTACTTGATACTTGCACATCAATGTTG 15882

ref GTCTTAATTTGTATTACTTGATACTTGCACATCAATGTTG 16008

Consensus gtcttaatttgtattacttgatacttgcacatcaatgttg

kal TGCATGAGGATGATGGGATGTTTTAATTTCACAAGTAATA 15800

yor TGCATGAGGATGATGGGATGTTTTAATTTCACAAGTAATA 15784

jen TGCATGAGGATGATGGGATGTTTTAATTTCACAAGTAATA 15800

cor TGCATGAGGATGATGGGATGTTTTAATTTCACAAGTAATA 15800

man TGCATGAGGATGATGGGATGTTTTAATTTCACAAGTAATA 15800

uni TGCATGAGGATGATGGGATGTTTTAATTTCACAAGTAATA 15800

tall TGCATGAGGATGATGGGATGTTTTAATTTCACAAGTAATA 15800

quil TGCATGAGGATGATGGGATGTTTTAATTTCACAAGTAATA 15766

meri TGCATGAGGATGATGGGATGTTTTAATTTCACAAGTAATA 15922

ref TGCATGAGGATGATGGGATGTTTTAATTTCACAAGTAATA 16048

Consensus tgcatgaggatgatgggatgttttaatttcacaagtaata

kal TGGGTTGTGTATAAAATGATCGCCGCAATGGTTAATATGT 15840

yor TGGGTTGTGTATAAAATGATCGCCGCAATGGTTAATATGT 15824

jen TGGGTTGTGTATAAAATGATCGCCGCAATGGTTAATATGT 15840

cor TGGGTTGTGTATAAAATGATCGCCGCAATGGTTAATATGT 15840

man TGGGTTGTGTATAAAATGATCGCCGCAATGGTTAATATGT 15840

uni TGGGTTGTGTATAAAATGATCGCCGCAATGGTTAATATGT 15840

tall TGGGTTGTGTATAAAATGATCGCCGCAATGGTTAATATGT 15840

quil TGGGTTGTGTATAAAATGATCGCCGCAATGGTTAATATGT 15806

meri TGGGTTGTGTATAAAATGATCGCCGCAATGGTTAATATGT 15962

ref TGGGTTGTGTATAAAATGATCGCCGCAATGGTTAATATGT 16088

Consensus tgggttgtgtataaaatgatcgccgcaatggttaatatgt

kal GTTTAAGTCCTGCTTATTAAAATATTTATACAGGGGCCAG 15880

yor GTTTAAGTCCTGCTTATTAAAATATTTATACAGGGGCCAG 15864

jen GTTTAAGTCCTGCTTATTAAAATATTTATACAGGGGCCAG 15880

cor GTTTAAGTCCTGCTTATTAAAATATTTATACAGGGGCCAG 15880

man GTTTAAGTCCTGCTTATTAAAATATTTATACAGGGGCCAG 15880

uni GTTTAAGTCCTGCTTATTAAAATATTTATACAGGGGCCAG 15880

tall GTTTAAGTCCTGCTTATTAAAATATTTATACAGGGGCCAG 15880

quil GTTTAAGTCCTGCTTATTAAAATATTTATACAGGGGCCAG 15846

meri GTTTAAGTCCTGCTTATTAAAATATTTATACAGGGGCCAG 16002

ref GTTTAAGTCCTGCTTATTAAAATATTTATACAGGGGCCAG 16128

Consensus gtttaagtcctgcttattaaaatatttatacaggggccag

kal TAACTTTTATGACATTAGAGTGTATAAATTATACTACCGT 15920

yor TAACTTTTATGACATTAGAGTGTATAAATTATACTACCGT 15904

jen TAACTTTTATGACATTAGAGTGTATAAATTATACTACCGT 15920

cor TAACTTTTATGACATTAGAGTGTATAAATTATACTACCGT 15920

man TAACTTTTATGACATTAGAGTGTATAAATTATACTACCGT 15920

uni TAACTTTTATGACATTAGAGTGTATAAATTATACTACCGT 15920

tall TAACTTTTATGACATTAGAGTGTATAAATTATACTACCGT 15920

quil TAACTTTTATGACATTAGAGTGTATAAATTATACTACCGT 15886

meri TAACTTTTATGACATTAGAGTGTATAAATTATACTACCGT 16042

ref TAACTTTTATGACATTAGAGTGTATAAATTATACTACCGT 16168

Consensus taacttttatgacattagagtgtataaattatactaccgt

kal ATTTTTATGTTTTACCTTTTAGTTTTAAAGGTTTAACTTA 15960

yor ATTTTTATGTTTTACCTTTTAGTTTTAAAGGTTTAACTTA 15944

jen ATTTTTATGTTTTACCTTTTAGTTTTAAAGGTTTAACTTA 15960

cor ATTTTTATGTTTTACCTTTTAGTTTTAAAGGTTTAACTTA 15960

man ATTTTTATGTTTTACCTTTTAGTTTTAAAGGTTTAACTTA 15960

uni ATTTTTATGTTTTACCTTTTAGTTTTAAAGGTTTAACTTA 15960

tall ATTTTTATGTTTTACCTTTTAGTTTTAAAGGTTTAACTTA 15960

quil ATTTTTATGTTTTACCTTTTAGTTTTAAAGGTTTAACTTA 15926

meri ATTTTTATGTTTTACCTTTTAGTTTTAAAGGTTTAACTTA 16082

ref ATTTTTATGTTTTACCTTTTAGTTTTAAAGGTTTAACTTA 16208

Consensus atttttatgttttaccttttagttttaaaggtttaactta

kal TGGATCATTTCCGTGTCTTGAAAGTGCTGATGATTGTGCA 16000

yor TGGATCATTTCCGTGTCTTGAAAGTGCTGATGATTGTGCA 15984

jen TGGATCATTTCCGTGTCTTGAAAGTGCTGATGATTGTGCA 16000

cor TGGATCATTTCCGTGTCTTGAAAGTGCTGATGATTGTGCA 16000

man TGGATCATTTCCGTGTCTTGAAAGTGCTGATGATTGTGCA 16000

uni TGGATCATTTCCGTGTCTTGAAAGTGCTGATGATTGTGCA 16000

tall TGGATCATTTCCGTGTCTTGAAAGTGCTGATGATTGTGCA 16000

quil TGGATCATTTCCGTGTCTTGAAAGTGCTGATGATTGTGCA 15966

meri TGGATCATTTCCGTGTCTTGAAAGTGCTGATGATTGTGCA 16122

ref TGGATCATTTCCGTGTCTTGAAAGTGCTGATGATTGTGCA 16248

Consensus tggatcatttccgtgtcttgaaagtgctgatgattgtgca

kal GAAATATCTTTTAAGTTATTCCTTGTTTGCCATTTAGCAT 16040

yor GAAATATCTTTTAAGTTATTCCTTGTTTGCCATTTAGCAT 16024

jen GAAATATCTTTTAAGTTATTCCTTGTTTGCCATTTAGCAT 16040

cor GAAATATCTTTTAAGTTATTCCTTGTTTGCCATTTAGCAT 16040

man GAAATATCTTTTAAGTTATTCCTTGTTTGCCATTTAGCAT 16040

uni GAAATATCTTTTAAGTTATTCCTTGTTTGCCATTTAGCAT 16040

tall GAAATATCTTTTAAGTTATTCCTTGTTTGCCATTTAGCAT 16040

quil GAAATATCTTTTAAGTTATTCCTTGTTTGCCATTTAGCAT 16006

meri GAAATATCTTTTAAGTTATTCCTTGTTTGCCATTTAGCAT 16162

ref GAAATATCTTTTAAGTTATTCCTTGTTTGCCATTTAGCAT 16288

Consensus gaaatatcttttaagttattccttgtttgccatttagcat

kal ATAATTTCCTCATGTTTTGTTGGCCACTCATTTTTTTTAG 16080

yor ATAATTTCCTCATGTTTTGTTGGCCACTCATTTTTTTTAG 16064

jen ATAATTTCCTCATGTTTTGTTGGCCACTCATTTTTTTTAG 16080

cor ATAATTTCCTCATGTTTTGTTGGCCACTCATTTTTTTTAG 16080

man ATAATTTCCTCATGTTTTGTTGGCCACTCATTTTTTTTAG 16080

uni ATAATTTCCTCATGTTTTGTTGGCCACTCATTTTTTTTAG 16080

tall ATAATTTCCTCATGTTTTGTTGGCCACTCATTTTTTTTAG 16080

quil ATAATTTCCTCATGTTTTGTTGGCCACTCATTTTTTTTAG 16046

meri ATAATTTCCTCATGTTTTGTTGGCCACTCATTTTTTTTAG 16202

ref ATAATTTCCTCATGTTTTGTTGGCCACTCATTTTTTTTAG 16328

Consensus ataatttcctcatgttttgttggccactcattttttttag

kal AGAGTATTAGTTTGTCATGCAGTATTTAACCTTTAACACT 16120

yor AGAGTATTAGTTTGTCATGCAGTATTTAACCTTTAACACT 16104

jen AGAGTATTAGTTTGTCATGCAGTATTTAACCTTTAACACT 16120

cor AGAGTATTAGTTTGTCATGCAGTATTTAACCTTTAACACT 16120

man AGAGTATTAGTTTGTCATGCAGTATTTAACCTTTAACACT 16120

uni AGAGTATTAGTTTGTCATGCAGTATTTAACCTTTAACACT 16120

tall AGAGTATTAGTTTGTCATGCAGTATTTAACCTTTAACACT 16120

quil AGAGTATTAGTTTGTCATGCAGTATTTAACCTTTAACACT 16086

meri AGAGTATTAGTTTGTCATGCAGTATTTAACCTTTAACACT 16242

ref AGAGTATTAGTTTGTCATGCAGTATTTAACCTTTAACACT 16368

Consensus agagtattagtttgtcatgcagtatttaacctttaacact

kal TGGTTTCGCACGCGACTGATATATGGTTTATTTTCCTTCA 16160

yor TGGTTTCGCACGCGACTGATATATGGTTTATTTTCCTTCA 16144

jen TGGTTTCGCACGCGACTGATATATGGTTTATTTTCCTTCA 16160

cor TGGTTTCGCACGCGACTGATATATGGTTTATTTTCCTTCA 16160

man TGGTTTCGCACGCGACTGATATATGGTTTATTTTCCTTCA 16160

uni TGGTTTCGCACGCGACTGATATATGGTTTATTTTCCTTCA 16160

tall TGGTTTCGCACGCGACTGATATATGGTTTATTTTCCTTCA 16160

quil TGGTTTCGCACGCGACTGATATATGGTTTATTTTCCTTCA 16126

meri TGGTTTCGCACGCGACTGATATATGGTTTATTTTCCTTCA 16282

ref TGGTTTCGCACGCGACTGATATATGGTTTATTTTCCTTCA 16408

Consensus tggtttcgcacgcgactgatatatggtttattttccttca

kal ACTTGAAGCAGCATATAGAGATGGTCATCTTCAATCCAGT 16200

yor ACTTGAAGCAGCATATAGAGATGGTCATCTTCAATCCAGT 16184

jen ACTTGAAGCAGCATATAGAGATGGTCATCTTCAATCCAGT 16200

cor ACTTGAAGCAGCATATAGAGATGGTCATCTTCAATCCAGT 16200

man ACTTGAAGCAGCATATAGAGATGGTCATCTTCAATCCAGT 16200

uni ACTTGAAGCAGCATATAGAGATGGTCATCTTCAATCCAGT 16200

tall ACTTGAAGCAGCATATAGAGATGGTCATCTTCAATCCAGT 16200

quil ACTTGAAGCAGCATATAGAGATGGTCATCTTCAATCCAGT 16166

meri ACTTGAAGCAGCATATAGAGATGGTCATCTTCAATCCAGT 16322

ref ACTTGAAGCAGCATATAGAGATGGTCATCTTCAATCCAGT 16448

Consensus acttgaagcagcatatagagatggtcatcttcaatccagt

kal CTTGAATGATGCCGAACATGCTCTAGACAAAGGTTGTAGC 16240

yor CTTGAATGATGCCGAACATGCTCTAGACAAAGGTTGTAGC 16224

jen CTTGAATGATGCCGAACATGCTCTAGACAAAGGTTGTAGC 16240

cor CTTGAATGATGCCGAACATGCTCTAGACAAAGGTTGTAGC 16240

man CTTGAATGATGCCGAACATGCTCTAGACAAAGGTTGTAGC 16240

uni CTTGAATGATGCCGAACATGCTCTAGACAAAGGTTGTAGC 16240

tall CTTGAATGATGCCGAACATGCTCTAGACAAAGGTTGTAGC 16240

quil CTTGAATGATGCCGAACATGCTCTAGACAAAGGTTGTAGC 16206

meri CTTGAATGATGCCGAACATGCTCTAGACAAAGGTTGTAGC 16362

ref CTTGAATGATGCCGAACATGCTCTAGACAAAGGTTGTAGC 16488

Consensus cttgaatgatgccgaacatgctctagacaaaggttgtagc

kal AGTATCGGTTCAAATATAATCATAAAGGAGGAAGGAAAAG 16280

yor AGTATCGGTTCAAATATAATCATAAAGGAGGAAGGAAAAG 16264

jen AGTATCGGTTCAAATATAATCATAAAGGAGGAAGGAAAAG 16280

cor AGTATCGGTTCAAATATAATCATAAAGGAGGAAGGAAAAG 16280

man AGTATCGGTTCAAATATAATCATAAAGGAGGAAGGAAAAG 16280

uni AGTATCGGTTCAAATATAATCATAAAGGAGGAAGGAAAAG 16280

tall AGTATCGGTTCAAATATAATCATAAAGGAGGAAGGAAAAG 16280

quil AGTATCGGTTCAAATATAATCATAAAGGAGGAAGGAAAAG 16246

meri AGTATCGGTTCAAATATAATCATAAAGGAGGAAGGAAAAG 16402

ref AGTATCGGTTCAAATATAATCATAAAGGAGGAAGGAAAAG 16528

Consensus agtatcggttcaaatataatcataaaggaggaaggaaaag

kal AACCCTGTGGAACTCCAGAAAATTCTAGAACCTCAGAACT 16320

yor AACCCTGTGGAACTCCAGAAAATTCTAGAACCTCAGAACT 16304

jen AACCCTGTGGAACTCCAGAAAATTCTAGAACCTCAGAACT 16320

cor AACCCTGTGGAACTCCAGAAAATTCTAGAACCTCAGAACT 16320

man AACCCTGTGGAACTCCAGAAAATTCTAGAACCTCAGAACT 16320

uni AACCCTGTGGAACTCCAGAAAATTCTAGAACCTCAGAACT 16320

tall AACCCTGTGGAACTCCAGAAAATTCTAGAACCTCAGAACT 16320

quil AACCCTGTGGAACTCCAGAAAATTCTAGAACCTCAGAACT 16286

meri AACCCTGTGGAACTCCAGAAAATTCTAGAACCTCAGAACT 16442

ref AACCCTGTGGAACTCCAGAAAATTCTAGAACCTCAGAACT 16568

Consensus aaccctgtggaactccagaaaattctagaacctcagaact

kal TCTTTTGGAGCTTCATGGAAAGAGTTCAAATGATCCTGAA 16360

yor TCTTTTGGAGCTTCATGGAAAGAGTTCAAATGATCCTGAA 16344

jen TCTTTTGGAGCTTCATGGAAAGAGTTCAAATGATCCTGAA 16360

cor TCTTTTGGAGCTTCATGGAAAGAGTTCAAATGATCCTGAA 16360

man TCTTTTGGAGCTTCATGGAAAGAGTTCAAATGATCCTGAA 16360

uni TCTTTTGGAGCTTCATGGAAAGAaTTCgAATGATCCTGAA 16360

tall TCTTTTGGAGCTTCATGGAAAGAGTTCAAATGATCCTGAA 16360

quil TCTTTTGGAGCTTCATGGAAAGAGTTCAAATGATCCTGAA 16326

meri TCTTTTGGAGCTTCATGGAAAGAGTTCAAATGATCCTGAA 16482

ref TCTTTTGGAGCTTCATGGAAAGAGTTCAAATGATCCTGAA 16608

Consensus tcttttggagcttcatggaaaga ttc aatgatcctgaa

kal AAGGAAGAAGGGAAAGAACTTCGTGGAACATCAGAAGATT 16400

yor AAGGAAGAAGGGAAAGAACTTCGTGGAACATCAGAAGATT 16384

jen AAGGAAGAAGGGAAAGAACTTCGTGGAACATCAGAAGATT 16400

cor AAGGAAGAAGGGAAAGAACTTCGTGGAACATCAGAAGATT 16400

man AAGGAAGAAGGGAAAGAACTTCGTGGAACATCAGAAGATT 16400

uni AAGGAAGAAGGaAAcaAACactaTGGAACtTCAGAAaATT 16400

tall AAGGAAGAAGGGAAAGAACTTCGTGGAACATCAGAAGATT 16400

quil AAGGAAGAAGGGAAAGAACTTCGTGGAACATCAGAAGATT 16366

meri AAGGAAGAAGGGAAAGAACTTCGTGGAACATCAGAAGATT 16522

ref AAGGAAGAAGGGAAAGAACTTCGTGGAACATCAGAAGATT 16648

Consensus aaggaagaagg aa aac tggaac tcagaa att

kal CCAGAAGCTCAGAACTTCTTTTGGAGCTTCATGGAAAGAG 16440

yor CCAGAAGCTCAGAACTTCTTTTGGAGCTTCATGGAAAGAG 16424

jen CCAGAAGCTCAGAACTTCTTTTGGAGCTTCATGGAAAGAG 16440

cor CCAGAAGCTCAGAACTTCTTTTGGAGCTTCATGGAAAGAG 16440

man CCAGAAGCTCAGAACTTCTTTTGGAGCTTCATGGAAAGAG 16440

uni gtAGAAtgTCAGAACTTCTTaTGGAGCTTCATGGAAAGAG 16440

tall CCAGAAGCTCAGAACTTCTTTTGGAGCTTCATGGAAAGAG 16440

quil CCAGAAGCTCAGAACTTCTTTTGGAGCTTCATGGAAAGAG 16406

meri CCAGAAGCTCAGAACTTCTTTTGGAGCTTCATGGAAAGAG 16562

ref CCAGAAGCTCAGAACTTCTTTTGGAGCTTCATGGAAAGAG 16688

Consensus agaa tcagaacttctt tggagcttcatggaaagag

kal ATTAAATGATGCTGAAAAGATTCAGGTCGATAAATCTCTA 16480

yor ATTAAATGATGCTGAAAAGATTCAGGTCGATAAATCTCTA 16464

jen ATTAAATGATGCTGAAAAGATTCAGGTCGATAAATCTCTA 16480

cor ATTAAATGATGCTGAAAAGATTCAGGTCGATAAATCTCTA 16480

man ATTAAATGATGCTGAAAAGATTCAGGTCGATAAATCTCTA 16480

uni ATTAAATGATGCTGAAAAGATTCAGGTCGATAAATCTCTA 16480

tall ATTAAATGATGCTGAAAAGATTCAGGTCGATAAATCTCTA 16480

quil ATTAAATGATGCTGAAAAGATTCAGGTCGATAAATCTCTA 16446

meri ATTAAATGATGCTGAAAAGATTCAGGTCGATAAATCTCTA 16602

ref ATTAAATGATGCTGAAAAGATTCAGGTCGATAAATCTCTA 16728

Consensus attaaatgatgctgaaaagattcaggtcgataaatctcta

kal TTACTATTTTATTTTTAGTTTAAACAAGCACTTATTTTCT 16520

yor TTACTATTTTATTTTTAGTTTAAACAAGCACTTATTTTCT 16504

jen TTACTATTTTATTTTTAGTTTAAACAAGCACTTATTTTCT 16520

cor TTACTATTTTATTTTTAGTTTAAACAAGCACTTATTTTCT 16520

man TTACTATTTTATTTTTAGTTTAAACAAGCACTTATTTTCT 16520

uni TTACTATTTTATTTTTAGTTTAAACAAGCACTTATTTTCT 16520

tall TTACTATTTTATTTTTAGTTTAAACAAGCACTTATTTTCT 16520

quil TTACTATTTTATTTTTAGTTTAAACAAGCACTTATTTTCT 16486

meri TTACTATTTTATTTTTAGTTTAAACAAGCACTTATTTTCT 16642

ref TTACTATTTTATTTTTAGTTTAAACAAGCACTTATTTTCT 16768

Consensus ttactattttatttttagtttaaacaagcacttattttct

kal CTTCCTGCTTGTCTTACCTTAATGTATTAATTTTAGAACT 16560

yor CTTCCTGCTTGTCTTACCTTAATGTATTAATTTTAGAACT 16544

jen CTTCCTGCTTGTCTTACCTTAATGTATTAATTTTAGAACT 16560

cor CTTCCTGCTTGTCTTACCTTAATGTATTAATTTTAGAACT 16560

man CTTCCTGCTTGTCTTACCTTAATGTATTAATTTTAGAACT 16560

uni CTTCCTGCTTGTCTTACCTTAATGTATTAATTTTAGAACT 16560

tall CTTCCTGCTTGTCTTACCTTAATGTATTAATTTTAGAACT 16560

quil CTTCCTGCTTGTCTTACCTTAATGTATTAATTTTAGAACT 16526

meri CTTCCTGCTTGTCTTACCTTAATGTATTAATTTTAGAACT 16682

ref CTTCCTGCTTGTCTTACCTTAATGTATTAATTTTAGAACT 16808

Consensus cttcctgcttgtcttaccttaatgtattaattttagaact

kal TGAGGTAAACGTATGCTTATTTTATGCGTATGTGCAATGG 16600

yor TGAGGTAAACGTATGCTTATTTTATGCGTATGTGCAATGG 16584

jen TGAGGTAAACGTATGCTTATTTTATGCGTATGTGCAATGG 16600

cor TGAGGTAAACGTATGCTTATTTTATGCGTATGTGCAATGG 16600

man TGAGGTAAACGTATGCTTATTTTATGCGTATGTGCAATGG 16600

uni TGAGGTAAACGTATGCTTATTTTATGCGTATGTGCAATGG 16600

tall TGAGGTAAACGTATGCTTATTTTATGCGTATGTGCAATGG 16600

quil TGAGGTAAACGTATGCTTATTTTATGCGTATGTGCAATGG 16566

meri TGAGGTAAACGTATGCTTATTTTATGCGTATGTGCAATGG 16722

ref TGAGGTAAACGTATGCTTATTTTATGCGTATGTGCAATGG 16848

Consensus tgaggtaaacgtatgcttattttatgcgtatgtgcaatgg

kal TTGCTTAACATGCCTACTAGCTGCACATGCAATTGCTTTT 16640

yor TTGCTTAACATGCCTACTAGCTGCACATGCAATTGCTTTT 16624

jen TTGCTTAACATGCCTACTAGCTGCACATGCAATTGCTTTT 16640

cor TTGCTTAACATGCCTACTAGCTGCACATGCAATTGCTTTT 16640

man TTGCTTAACATGCCTACTAGCTGCACATGCAATTGCTTTT 16640

uni TTGCTTAACATGCCTACTAGCTGCACATGCAATTGCTTTT 16640

tall TTGCTTAACATGCCTACTAGCTGCACATGCAATTGCTTTT 16640

quil TTGCTTAACATGCCTACTAGCTGCACATGCAATTGCTTTT 16606

meri TTGCTTAACATGCCTACTAGCTGCACATGCAATTGCTTTT 16762

ref TTGCTTAACATGCCTACTAGCTGCACATGCAATTGCTTTT 16888

Consensus ttgcttaacatgcctactagctgcacatgcaattgctttt

kal AATTATATATATTTAAAACATGATTGTTGTACAGTAATTT 16680

yor AATTATATATATTTAAAACATGATTGTTGTACAGTAATTT 16664

jen AATTATATATATTTAAAACATGATTGTTGTACAGTAATTT 16680

cor AATTATATATATTTAAAACATGATTGTTGTACAGTAATTT 16680

man AATTATATATATTTAAAACATGATTGTTGTACAGTAATTT 16680

uni AATTATATATATTTAAAACATGATTGTTGTACAGTAATTT 16680

tall AATTATATATATTTAAAACATGATTGTTGTACAGTAATTT 16680

quil AATTATATATATTTAAAACATGATTGTTGTACAGTAATTT 16646

meri AATTATATATATTTAAAACATGATTGTTGTACAGTAATTT 16802

ref AATTATATATATTTAAAACATGATTGTTGTACAGTAATTT 16928

Consensus aattatatatatttaaaacatgattgttgtacagtaattt

kal TATGCCTCTAGAACACATAGAACCTTGTTTTAGGTACTGA 16720

yor TATGCCTCTAGAACACATAGAACCTTGTTTTAGGTACTGA 16704

jen TATGCCTCTAGAACACATAGAACCTTGTTTTAGGTACTGA 16720

cor TATGCCTCTAGAACACATAGAACCTTGTTTTAGGTACTGA 16720

man TATGCCTCTAGAACACATAGAACCTTGTTTTAGGTACTGA 16720

uni TATGCCTCTAGAACACATAGAACCTTGTTTTAGGTACTGA 16720

tall TATGCCTCTAGAACACATAGAACCTTGTTTTAGGTACTGA 16720

quil TATGCCTCTAGAACACATAGAACCTTGTTTTAGGTACTGA 16686

meri TATGCCTCTAGAACACATAGAACCTTGTTTTAGGTACTGA 16842

ref TATGCCTCTAGAACACATAGAACCTTGTTTTAGGTACTGA 16968

Consensus tatgcctctagaacacatagaaccttgttttaggtactga

kal ATATGATATGATTTATATGGAGACACAATTGATCACTAAT 16760

yor ATATGATATGATTTATATGGAGACACAATTGATCACTAAT 16744

jen ATATGATATGATTTATATGGAGACACAATTGATCACTAAT 16760

cor ATATGATATGATTTATATGGAGACACAATTGATCACTAAT 16760

man ATATGATATGATTTATATGGAGACACAATTGATCACTAAT 16760

uni ATATGATATGATTTATATGGAGACACAATTGATCACTAAT 16760

tall ATATGATATGATTTATATGGAGACACAATTGATCACTAAT 16760

quil ATATGATATGATTTATATGGAGACACAATTGATCACTAAT 16726

meri ATATGATATGATTTATATGGAGACACAATTGATCACTAAT 16882

ref ATATGATATGATTTATATGGAGACACAATTGATCACTAAT 17008

Consensus atatgatatgatttatatggagacacaattgatcactaat

kal AATAGCATTTATGATAGGGTTGGCGATATCCATTTAAAGA 16800

yor AATAGCATTTATGATAGGGTTGGCGATATCCATTTAAAGA 16784

jen AATAGCATTTATGATAGGGTTGGCGATATCCATTTAAAGA 16800

cor AATAGCATTTATGATAGGGTTGGCGATATCCATTTAAAGA 16800

man AATAGCATTTATGATAGGGTTGGCGATATCCATTTAAAGA 16800

uni AATAGCATTTATGATAGGGTTGGCGATATCCATTTAAAGA 16800

tall AATAGCATTTATGATAGGGTTGGCGATATCCATTTAAAGA 16800

quil AATAGCATTTATGATAGGGTTGGCGATATCCATTTAAAGA 16766

meri AATAGCATTTATGATAGGGTTGGCGATATCCATTTAAAGA 16922

ref AATAGCATTTATGATAGGGTTGGCGATATCCATTTAAAGA 17048

Consensus aatagcatttatgatagggttggcgatatccatttaaaga

kal TTTTGGCATGTGCTGTAGACTTGCAGGCATAGATAAATAA 16840

yor TTTTGGCATGTGCTGTAGACTTGCAGGCATAGATAAATAA 16824

jen TTTTGGCATGTGCTGTAGACTTGCAGGCATAGATAAATAA 16840

cor TTTTGGCATGTGCTGTAGACTTGCAGGCATAGATAAATAA 16840

man TTTTGGCATGTGCTGTAGACTTGCAGGCATAGATAAATAA 16840

uni TTTTGGCATGTGCTGTAGACTTGCAGGCATAGATAAATAA 16840

tall TTTTGGCATGTGCTGTAGACTTGCAGGCATAGATAAATAA 16840

quil TTTTGGCATGTGCTGTAGACTTGCAGGCATAGATAAATAA 16806

meri TTTTGGCATGTGCTGTAGACTTGCAGGCATAGATAAATAA 16962

ref TTTTGGCATGTGCTGTAGACTTGCAGGCATAGATAAATAA 17088

Consensus ttttggcatgtgctgtagacttgcaggcatagataaataa

kal GCCTAGCACATGATTCTCTGCTCTTGGTTTTAGGGTAGGC 16880

yor GCCTAGCACATGATTCTCTGCTCTTGGTTTTAGGGTAGGC 16864

jen GCCTAGCACATGATTCTCTGCTCTTGGTTTTAGGGTAGGC 16880

cor GCCTAGCACATGATTCTCTGCTCTTGGTTTTAGGGTAGGC 16880

man GCCTAGCACATGATTCTCTGCTCTTGGTTTTAGGGTAGGC 16880

uni GCCTAGCACATGATTCTCTGCTCTTGGTTTTAGGGTAGGC 16880

tall GCCTAGCACATGATTCTCTGCTCTTGGTTTTAGGGTAGGC 16880

quil GCCTAGCACATGATTCTCTGCTCTTGGTTTTAGGGTAGGC 16846

meri GCCTAGCACATGATTCTCTGCTCTTGGTTTTAGGGTAGGC 17002

ref GCCTAGCACATGATTCTCTGCTCTTGGTTTTAGGGTAGGC 17128

Consensus gcctagcacatgattctctgctcttggttttagggtaggc

kal CTCTCATAGTGTTACATTGAGTCATATTCATTTCCTCAAT 16920

yor CTCTCATAGTGTTACATTGAGTCATATTCATTTCCTCAAT 16904

jen CTCTCATAGTGTTACATTGAGTCATATTCATTTCCTCAAT 16920

cor CTCTCATAGTGTTACATTGAGTCATATTCATTTCCTCAAT 16920

man CTCTCATAGTGTTACATTGAGTCATATTCATTTCCTCAAT 16920

uni CTCTCATAGTGTTACATTGAGTCATATTCATTTCCTCAAT 16920

tall CTCTCATAGTGTTACATTGAGTCATATTCATTTCCTCAAT 16920

quil CTCTCATAGTGTTACATTGAGTCATATTCATTTCCTCAAT 16886

meri CTCTCATAGTGTTACATTGAGTCATATTCATTTCCTCAAT 17042

ref CTCTCATAGTGTTACATTGAGTCATATTCATTTCCTCAAT 17168

Consensus ctctcatagtgttacattgagtcatattcatttcctcaat

kal TCTGTCTGTTATTTCTTTTCCTTCTGTTTTGAATTTCAAC 16960

yor TCTGTCTGTTATTTCTTTTCCTTCTGTTTTGAATTTCAAC 16944

jen TCTGTCTGTTATTTCTTTTCCTTCTGTTTTGAATTTCAAC 16960

cor TCTGTCTGTTATTTCTTTTCCTTCTGTTTTGAATTTCAAC 16960

man TCTGTCTGTTATTTCTTTTCCTTCTGTTTTGAATTTCAAC 16960

uni TCTGTCTGTTATTTCTTTTCCTTCTGTTTTGAATTTCAAC 16960

tall TCTGTCTGTTATTTCTTTTCCTTCTGTTTTGAATTTCAAC 16960

quil TCTGTCTGTTATTTCTTTTCCTTCTGTTTTGAATTTCAAC 16926

meri TCTGTCTGTTATTTCTTTTCCTTCTGTTTTGAATTTCAAC 17082

ref TCTGTCTGTTATTTCTTTTCCTTCTGTTTTGAATTTCAAC 17208

Consensus tctgtctgttatttcttttccttctgttttgaatttcaac

kal AAATCCTGAACATGCAAGTCTGGACAGAATAATACAGTCT 17000

yor AAATCCTGAACATGCAAGTCTGGACAGAATAATACAGTCT 16984

jen AAATCCTGAACATGCAAGTCTGGACAGAATAATACAGTCT 17000

cor AAATCCTGAACATGCAAGTCTGGACAGAATAATACAGTCT 17000

man AAATCCTGAACATGCAAGTCTGGACAGAATAATACAGTCT 17000

uni AAATCCTGAACATGCAAGTCTGGACAGAATAATACAGTCT 17000

tall AAATCCTGAACATGCAAGTCTGGACAGAATAATACAGTCT 17000

quil AAATCCTGAACATGCAAGTCTGGACAGAATAATACAGTCT 16966

meri AAATCCTGAACATGCAAGTCTGGACAGAATAATACAGTCT 17122

ref AAATCCTGAACATGCAAGTCTGGACAGAATAATACAGTCT 17248

Consensus aaatcctgaacatgcaagtctggacagaataatacagtct

kal ATTCCATCAGCTCTTTGGTTTTCCTGATCTTACATTCATT 17040

yor ATTCCATCAGCTCTTTGGTTTTCCTGATCTTACATTCATT 17024

jen ATTCCATCAGCTCTTTGGTTTTCCTGATCTTACATTCATT 17040

cor ATTCCATCAGCTCTTTGGTTTTCCTGATCTTACATTCATT 17040

man ATTCCATCAGCTCTTTGGTTTTCCTGATCTTACATTCATT 17040

uni ATTCCATCAGCTCTTTGGTTTTCCTGATCTTACATTCATT 17040

tall ATTCCATCAGCTCTTTGGTTTTCCTGATCTTACATTCATT 17040

quil ATTCCATCAGCTCTTTGGTTTTCCTGATCTTACATTCATT 17006

meri ATTCCATCAGCTCTTTGGTTTTCCTGATCTTACATTCATT 17162

ref ATTCCATCAGCTCTTTGGTTTTCCTGATCTTACATTCATT 17288

Consensus attccatcagctctttggttttcctgatcttacattcatt

kal CAGTTATATATACACAATGAGTGAAAAGTTTAAGGGAGAC 17080

yor CAGTTATATATACACAATGAGTGAAAAGTTTAAGGGAGAC 17064

jen CAGTTATATATACACAATGAGTGAAAAGTTTAAGGGAGAC 17080

cor CAGTTATATATACACAATGAGTGAAAAGTTTAAGGGAGAC 17080

man CAGTTATATATACACAATGAGTGAAAAGTTTAAGGGAGAC 17080

uni CAGTTATATATACACAATGAGTGAAAAGTTTAAGGGAGAC 17080

tall CAGTTATATATACACAATGAGTGAAAAGTTTAAGGGAGAC 17080

quil CAGTTATATATACACAATGAGTGAAAAGTTTAAGGGAGAC 17046

meri CAGTTATATATACACAATGAGTGAAAAGTTTAAGGGAGAC 17202

ref CAGTTATATATACACAATGAGTGAAAAGTTTAAGGGAGAC 17328

Consensus cagttatatatacacaatgagtgaaaagtttaagggagac

kal AGTCATATACTGGCCCCGACTGTACGAACCCGTGTTTAGC 17120

yor AGTCATATACTGGCCCCGACTGTACGAACCCGTGTTTAGC 17104

jen AGTCATATACTGGCCCCGACTGTACGAACCCGTGTTTAGC 17120

cor AGTCATATACTGGCCCCGACTGTACGAACCCGTGTTTAGC 17120

man AGTCATATACTGGCCCCGACTGTACGAACCCGTGTTTAGC 17120

uni AGTCATATACTGGCCCCGACTGTACGAACCCGTGTTTAGC 17120

tall AGTCATATACTGGCCCCGACTGTACGAACCCGTGTTTAGC 17120

quil AGTCATATACTGGCCCCGACTGTACGAACCCGTGTTTAGC 17086

meri AGTCATATACTGGCCCCGACTGTACGAACCCGTGTTTAGC 17242

ref AGTCATATACTGGCCCCGACTGTACGAACCCGTGTTTAGC 17368

Consensus agtcatatactggccccgactgtacgaacccgtgtttagc

kal ATTGAAACCTAAGGAAAATAGAGTAGATAAAGGCCAAAGT 17160

yor ATTGAAACCTAAGGAAAATAGAGTAGATAAAGGCCAAAGT 17144

jen ATTGAAACCTAAGGAAAATAGAGTAGATAAAGGCCAAAGT 17160

cor ATTGAAACCTAAGGAAAATAGAGTAGATAAAGGCCAAAGT 17160

man ATTGAAACCTAAGGAAAATAGAGTAGATAAAGGCCAAAGT 17160

uni ATTGAAACCTAAGGAAAATAGAGTAGATAAAGGCCAAAGT 17160

tall ATTGAAACCTAAGGAAAATAGAGTAGATAAAGGCCAAAGT 17160

quil ATTGAAACCTAAGGAAAATAGAGTAGATAAAGGCCAAAGT 17126

meri ATTGAAACCTAAGGAAAATAGAGTAGATAAAGGCCAAAGT 17282

ref ATTGAAACCTAAGGAAAATAGAGTAGATAAAGGCCAAAGT 17408

Consensus attgaaacctaaggaaaatagagtagataaaggccaaagt

kal GATAGCAGGTCTCTAACAGGTCTCTAACTTTGCATTTAGC 17200

yor GATAGCAGGTCTCTAACAGGTCTCTAACTTTGCATTTAGC 17184

jen GATAGCAGGTCTCTAACAGGTCTCTAACTTTGCATTTAGC 17200

cor GATAGCAGGTCTCTAACAGGTCTCTAACTTTGCATTTAGC 17200

man GATAGCAGGTCTCTAACAGGTCTCTAACTTTGCATTTAGC 17200

uni GATAGCAGGTCTCTAACAGGTCTCTAACTTTGCATTTAGC 17200

tall GATAGCAGGTCTCTAACAGGTCTCTAACTTTGCATTTAGC 17200

quil GATAGCAGGTCTCTAACAGGTCTCTAACTTTGCATTTAGC 17166

meri GATAGCAGGTCTCTAACAGGTCTCTAACTTTGCATTTAGC 17322

ref GATAGCAGGTCTCTAACAGGTCTCTAACTTTGCATTTAGC 17448

Consensus gatagcaggtctctaacaggtctctaactttgcatttagc

kal TGCATGATAAATTTGAGGTAAGGATCAAGGGCCATACCAT 17240

yor TGCATGATAAATTTGAGGTAAGGATCAAGGGCCATACCAT 17224

jen TGCATGATAAATTTGAGGTAAGGATCAAGGGCCATACCAT 17240

cor TGCATGATAAATTTGAGGTAAGGATCAAGGGCCATACCAT 17240

man TGCATGATAAATTTGAGGTAAGGATCAAGGGCCATACCAT 17240

uni TGCATGATAAATTTGAGGTAAGGATCAAGGGCCATACCAT 17240

tall TGCATGATAAATTTGAGGTAAGGATCAAGGGCCATACCAT 17240

quil TGCATGATAAATTTGAGGTAAGGATCAAGGGCCATACCAT 17206

meri TGCATGATAAATTTGAGGTAAGGATCAAGGGCCATACCAT 17362

ref TGCATGATAAATTTGAGGTAAGGATCAAGGGCCATACCAT 17488

Consensus tgcatgataaatttgaggtaaggatcaagggccataccat

kal TGTGGAGAGTACTATTTATACCTTCTTAGGTAACACCTTG 17280

yor TGTGGAGAGTACTATTTATACCTTCTTAGGTAACACCTTG 17264

jen TGTGGAGAGTACTATTTATACCTTCTTAGGTAACACCTTG 17280

cor TGTGGAGAGTACTATTTATACCTTCTTAGGTAACACCTTG 17280

man TGTGGAGAGTACTATTTATACCTTCTTAGGTAACACCTTG 17280

uni TGTGGAGAGTACTATTTATACCTTCTTAGGTAACACCTTG 17280

tall TGTGGAGAGTACTATTTATACCTTCTTAGGTAACACCTTG 17280

quil TGTGGAGAGTACTATTTATACCTTCTTAGGTAACACCTTG 17246

meri TGTGGAGAGTACTATTTATACCTTCTTAGGTAACACCTTG 17402

ref TGTGGAGAGTACTATTTATACCTTCTTAGGTAACACCTTG 17528

Consensus tgtggagagtactatttataccttcttaggtaacaccttg

kal CCTTTTATACTGTACATGATGCCTTGAATCTGTTGTATTG 17320

yor CCTTTTATACTGTACATGATGCCTTGAATCTGTTGTATTG 17304

jen CCTTTTATACTGTACATGATGCCTTGAATCTGTTGTATTG 17320

cor CCTTTTATACTGTACATGATGCCTTGAATCTGTTGTATTG 17320

man CCTTTTATACTGTACATGATGCCTTGAATCTGTTGTATTG 17320

uni CCTTTTATACTGTACATGATGCCTTGAATCTGTTGTATTG 17320

tall CCTTTTATACTGTACATGATGCCTTGAATCTGTTGTATTG 17320

quil CCTTTTATACTGTACATGATGCCTTGAATCTGTTGTATTG 17286

meri CCTTTTATACTGTACATGATGCCTTGAATCTGTTGTATTG 17442

ref CCTTTTATACTGTACATGATGCCTTGAATCTGTTGTATTG 17568

Consensus ccttttatactgtacatgatgccttgaatctgttgtattg

kal ATTTTCCTTGCCAAGCTTGTCCCAGCCAGTGGACCTGCTA 17360

yor ATTTTCCTTGCCAAGCTTGTCCCAGCCAGTGGACCTGCTA 17344

jen ATTTTCCTTGCCAAGCTTGTCCCAGCCAGTGGACCTGCTA 17360

cor ATTTTCCTTGCCAAGCTTGTCCCAGCCAGTGGACCTGCTA 17360

man ATTTTCCTTGCCAAGCTTGTCCCAGCCAGTGGACCTGCTA 17360

uni ATTTTCCTTGCCAAGCTTGTCCCAGCCAGTGGACCTGCTA 17360

tall ATTTTCCTTGCCAAGCTTGTCCCAGCCAGTGGACCTGCTA 17360

quil ATTTTCCTTGCCAAGCTTGTCCCAGCCAGTGGACCTGCTA 17326

meri ATTTTCCTTGCCAAGCTTGTCCCAGCCAGTGGACCTGCTA 17482

ref ATTTTCCTTGCCAAGCTTGTCCCAGCCAGTGGACCTGCTA 17608

Consensus attttccttgccaagcttgtcccagccagtggacctgcta

kal CTGGTCCTGACTATATTTTTCTACTTTTTTATAGGAACTG 17400

yor CTGGTCCTGACTATATTTTTCTACTTTTTTATAGGAACTG 17384

jen CTGGTCCTGACTATATTTTTCTACTTTTTTATAGGAACTG 17400

cor CTGGTCCTGACTATATTTTTCTACTTTTTTATAGGAACTG 17400

man CTGGTCCTGACTATATTTTTCTACTTTTTTATAGGAACTG 17400

uni CTGGTCCTGACTATATTTTTCTACTTTTTTATAGGAACTG 17400

tall CTGGTCCTGACTATATTTTTCTACTTTTTTATAGGAACTG 17400

quil CTGGTCCTGACTATATTTTTCTACTTTTTTATAGGAACTG 17366

meri CTGGTCCTGACTATATTTTTCTACTTTTTTATAGGAACTG 17522

ref CTGGTCCTGACTATATTTTTCTACTTTTTTATAGGAACTG 17648

Consensus ctggtcctgactatatttttctacttttttataggaactg

kal GTCACAAAATCTCTTGTGACATGTCCTGGTTTGGGGTCTG 17440

yor GTCACAAAATCTCTTGTGACATGTCCTGGTTTGGGGTCTG 17424

jen GTCACAAAATCTCTTGTGACATGTCCTGGTTTGGGGTCTG 17440

cor GTCACAAAATCTCTTGTGACATGTCCTGGTTTGGGGTCTG 17440

man GTCACAAAATCTCTTGTGACATGTCCTGGTTTGGGGTCTG 17440

uni GTCACAAAATCTCTTGTGACATGTCCTGGTTTGGGGTCTG 17440

tall GTCACAAAATCTCTTGTGACATGTCCTGGTTTGGGGTCTG 17440

quil GTCACAAAATCTCTTGTGACATGTCCTGGTTTGGGGTCTG 17406

meri GTCACAAAATCTCTTGTGACATGTCCTGGTTTGGGGTCTG 17562

ref GTCACAAAATCTCTTGTGACATGTCCTGGTTTGGGGTCTG 17688

Consensus gtcacaaaatctcttgtgacatgtcctggtttggggtctg

kal TCAGCTGTGCCGCTGATCATTCTGAAGTGGTGAAATTATC 17480

yor TCAGCTGTGCCGCTGATCATTCTGAAGTGGTGAAATTATC 17464

jen TCAGCTGTGCCGCTGATCATTCTGAAGTGGTGAAATTATC 17480

cor TCAGCTGTGCCGCTGATCATTCTGAAGTGGTGAAATTATC 17480

man TCAGCTGTGCCGCTGATCATTCTGAAGTGGTGAAATTATC 17480

uni TCAGCTGTGCCGCTGATCATTCTGAAGTGGTGAAATTATC 17480

tall TCAGCTGTGCCGCTGATCATTCTGAAGTGGTGAAATTATC 17480

quil TCAGCTGTGCCGCTGATCATTCTGAAGTGGTGAAATTATC 17446

meri TCAGCTGTGCCGCTGATCATTCTGAAGTGGTGAAATTATC 17602

ref TCAGCTGTGCCGCTGATCATTCTGAAGTGGTGAAATTATC 17728

Consensus tcagctgtgccgctgatcattctgaagtggtgaaattatc

kal CGGAACAGCAAATAATATGCTTACCAAAAGTGAGGAGGTC 17520

yor CGGAACAGCAAATAATATGCTTACCAAAAGTGAGGAGGTC 17504

jen CGGAACAGCAAATAATATGCTTACCAAAAGTGAGGAGGTC 17520

cor CGGAACAGCAAATAATATGCTTACCAAAAGTGAGGAGGTC 17520

man CGGAACAGCAAATAATATGCTTACCAAAAGTGAGGAGGTC 17520

uni CGGAACAGCAAATAATATGCTTACCAAAAGTGAGGAGGTC 17520

tall CGGAACAGCAAATAATATGCTTACCAAAAGTGAGGAGGTC 17520

quil CGGAACAGCAAATAATATGCTTACCAAAAGTGAGGAGGTC 17486

meri CGGAACAGCAAATAATATGCTTACCAAAAGTGAGGAGGTC 17642

ref CGGAACAGCAAATAATATGCTTACCAAAAGTGAGGAGGTC 17768

Consensus cggaacagcaaataatatgcttaccaaaagtgaggaggtc

kal AGAAGCCAACTCATTGCAATTGGCGCCAAGGGGAGGAAAA 17560

yor AGAAGCCAACTCATTGCAATTGGCGCCAAGGGGAGGAAAA 17544

jen AGAAGCCAACTCATTGCAATTGGCGCCAAGGGGAGGAAAA 17560

cor AGAAGCCAACTCATTGCAATTGGCGCCAAGGGGAGGAAAA 17560

man AGAAGCCAACTCATTGCAATTGGCGCCAAGGGGAGGAAAA 17560

uni AGAAGCCAACTCATTGCAATTGGCGCCAAGGGGAGGAAAA 17560

tall AGAAGCCAACTCATTGCAATTGGCGCCAAGGGGAGGAAAA 17560

quil AGAAGCCAACTCATTGCAATTGGCGCCAAGGGGAGGAAAA 17526

meri AGAAGCCAACTCATTGCAATTGGCGCCAAGGGGAGGAAAA 17682

ref AGAAGCCAACTCATTGCAATTGGCGCCAAGGGGAGGAAAA 17808

Consensus agaagccaactcattgcaattggcgccaaggggaggaaaa

kal TGTATCTTTCTTCCAGATTAAGTGCTTCACAATTTGAAAA 17600

yor TGTATCTTTCTTCCAGATTAAGTGCTTCACAATTTGAAAA 17584

jen TGTATCTTTCTTCCAGATTAAGTGCTTCACAATTTGAAAA 17600

cor TGTATCTTTCTTCCAGATTAAGTGCTTCACAATTTGAAAA 17600

man TGTATCTTTCTTCCAGATTAAGTGCTTCACAATTTGAAAA 17600

uni TGTATCTTTCTTCCAGATTAAGTGCTTCACAATTTGAAAA 17600

tall TGTATCTTTCTTCCAGATTAAGTGCTTCACAATTTGAAAA 17600

quil TGTATCTTTCTTCCAGATTAAGTGCTTCACAATTTGAAAA 17566

meri TGTATCTTTCTTCCAGATTAAGTGCTTCACAATTTGAAAA 17722

ref TGTATCTTTCTTCCAGATTAAGTGCTTCACAATTTGAAAA 17848

Consensus tgtatctttcttccagattaagtgcttcacaatttgaaaa

kal TTATGATCTAGACATAAAAAATTGCGATCTTGCTCGTAAA 17640

yor TTATGATCTAGACATAAAAAATTGCGATCTTGCTCGTAAA 17624

jen TTATGATCTAGACATAAAAAATTGCGATCTTGCTCGTAAA 17640

cor TTATGATCTAGACATAAAAAATTGCGATCTTGCTCGTAAA 17640

man TTATGATCTAGACATAAAAAATTGCGATCTTGCTCGTAAA 17640

uni TTATGATCTAGACATAAAAAATTGCGATCTTGCTCGTAAA 17640

tall TTATGATCTAGACATAAAAAATTGCGATCTTGCTCGTAAA 17640

quil TTATGATCTAGACATAAAAAATTGCGATCTTGCTCGTAAA 17606

meri TTATGATCTAGACATAAAAAATTGCGATCTTGCTCGTAAA 17762

ref TTATGATCTAGACATAAAAAATTGCGATCTTGCTCGTAAA 17888

Consensus ttatgatctagacataaaaaattgcgatcttgctcgtaaa

kal CCTGCACGAAGAGCTTGCAAGAAAGGTTCCAAGGTCGCTC 17680

yor CCTGCACGAAGAGCTTGCAAGAAAGGTTCCAAGGTCGCTC 17664

jen CCTGCACGAAGAGCTTGCAAGAAAGGTTCCAAGGTCGCTC 17680

cor CCTGCACGAAGAGCTTGCAAGAAAGGTTCCAAGGTCGCTC 17680

man CCTGCACGAAGAGCTTGCAAGAAAGGTTCCAAGGTCGCTC 17680

uni CCTGCACGAAGAGCTTGCAAGAAAGGTTCCAAGGTCGCTC 17680

tall CCTGCACGAAGAGCTTGCAAGAAAGGTTCCAAGGTCGCTC 17680

quil CCTGCACGAAGAGCTTGCAAGAAAGGTTCCAAGGTCGCTC 17646

meri CCTGCACGAAGAGCTTGCAAGAAAGGTTCCAAGGTCGCTC 17802

ref CCTGCACGAAGAGCTTGCAAGAAAGGTTCCAAGGTCGCTC 17928

Consensus cctgcacgaagagcttgcaagaaaggttccaaggtcgctc

kal CAGATGAGGATCTGGACTCTATGGAGTTAGCTCTACAAGT 17720

yor CAGATGAGGATCTGGACTCTATGGAGTTAGCTCTACAAGT 17704

jen CAGATGAGGATCTGGACTCTATGGAGTTAGCTCTACAAGT 17720

cor CAGATGAGGATCTGGACTCTATGGAGTTAGCTCTACAAGT 17720

man CAGATGAGGATCTGGACTCTATGGAGTTAGCTCTACAAGT 17720

uni CAGATGAGGATCTGGACTCTATGGAGTTAGCTCTACAAGT 17720

tall CAGATGAGGATCTGGACTCTATGGAGTTAGCTCTACAAGT 17720

quil CAGATGAGGATCTGGACTCTATGGAGTTAGCTCTACAAGT 17686

meri CAGATGAGGATCTGGACTCTATGGAGTTAGCTCTACAAGT 17842

ref CAGATGAGGATCTGGACTCTATGGAGTTAGCTCTACAAGT 17968

Consensus cagatgaggatctggactctatggagttagctctacaagt

kal TGTTTACCCTCAAGAATTGTGTCTTGCTGATACTGAATCG 17760

yor TGTTTACCCTCAAGAATTGTGTCTTGCTGATACTGAATCG 17744

jen TGTTTACCCTCAAGAATTGTGTCTTGCTGATACTGAATCG 17760

cor TGTTTACCCTCAAGAATTGTGTCTTGCTGATACTGAATCG 17760

man TGTTTACCCTCAAGAATTGTGTCTTGCTGATACTGAATCG 17760

uni TGTTTACCCTCAAGAATTGTGTCTTGCTGATACTGAATCG 17760

tall TGTTTACCCTCAAGAATTGTGTCTTGCTGATACTGAATCG 17760

quil TGTTTACCCTCAAGAATTGTGTCTTGCTGATACTGAATCG 17726

meri TGTTTACCCTCAAGAATTGTGTCTTGCTGATACTGAATCG 17882

ref TGTTTACCCTCAAGAATTGTGTCTTGCTGATACTGAATCG 18008

Consensus tgtttaccctcaagaattgtgtcttgctgatactgaatcg

kal TGCTCCTTTGAAGGAAGTTATGGTGATAAGTCCCAAGATA 17800

yor TGCTCCTTTGAAGGAAGTTATGGTGATAAGTCCCAAGATA 17784

jen TGCTCCTTTGAAGGAAGTTATGGTGATAAGTCCCAAGATA 17800

cor TGCTCCTTTGAAGGAAGTTATGGTGATAAGTCCCAAGATA 17800

man TGCTCCTTTGAAGGAAGTTATGGTGATAAGTCCCAAGATA 17800

uni TGCTCCTTTGAAGGAAGTTATGGTGATAAGTCCCAAGATA 17800

tall TGCTCCTTTGAAGGAAGTTATGGTGATAAGTCCCAAGATA 17800

quil TGCTCCTTTGAAGGAAGTTATGGTGATAAGTCCCAAGATA 17766

meri TGCTCCTTTGAAGGAAGTTATGGTGATAAGTCCCAAGATA 17922

ref TGCTCCTTTGAAGGAAGTTATGGTGATAAGTCCCAAGATA 18048

Consensus tgctcctttgaaggaagttatggtgataagtcccaagata

kal CCAGACTCATTCATGCTGAAAATTCTGCATTGATTTCACT 17840

yor CCAGACTtATTCATGCTGAAAATTCTGCATTGATTTCACT 17824

jen CCAGACTCATTCATGCTGAAAATTCTGCATTGATTTCACT 17840

cor CCAGACTCATTCATGCTGAAAATTCTGCATTGATTTCACT 17840

man CCAGACTCATTCATGCTGAAAATTCTGCATTGATTTCACT 17840

uni CCAGACTCATTCATGCTGAAAATTCTGCATTGATTTCACT 17840

tall CCAGACTCATTCATGCTGAAAATTCTGCATTGATTTCACT 17840

quil CCAGACTCATTCATGCTGAAAATTCTGCATTGATTTCACT 17806

meri CCAGACTCATTCATGCTGAAAATTCTGCATTGATTTCACT 17962

ref CCAGACTtATTCATGCTGAAAATTCTGCATTGATTTCACT 18088

Consensus ccagact attcatgctgaaaattctgcattgatttcact

kal GTTAAAGAAGCCAACCCAGAGTGCCCTGTTTCCTGGGATA 17880

yor GTTAAAGAAGCCAACCCAGAGTGCCCTGTTTCCTGGGATA 17864

jen GTTAAAGAAGCCAACCCAGAGTGCCCTGTTTCCTGGGATA 17880

cor GTTAAAGAAGCCAACCCAGAGTGCCCTGTTTCCTGGGATA 17880

man GTTAAAGAAGCCAACCCAGAGTGCCCTGTTTCCTGGGATA 17880

uni GTTAAAGAAGCCAACCCAGAGTGCCCTGTTTCCTGGGATA 17880

tall GTTAAAGAAGCCAACCCAGAGTGCCCTGTTTCCTGGGATA 17880

quil GTTAAAGAAGCCAACCCAGAGTGCCCTGTTTCCTGGGATA 17846

meri GTTAAAGAAGCCAACCCAGAGTGCCCTGTTTCCTGGGATA 18002

ref GTTAAAGAAGCCAACCCAGAGTGCCCTGTTTCCTGGGATA 18128

Consensus gttaaagaagccaacccagagtgccctgtttcctgggata

kal CAGCTGATAGATGAAGAGAAACAACCACCACAAGGCGTTA 17920

yor CAGCTGATAGATGAAGAGAAACAACCACCACAAGGCGTTA 17904

jen CAGCTGATAGATGAAGAGAAACAACCACCACAAGGCGTTA 17920

cor CAGCTGATAGATGAAGAGAAACAACCACCACAAGGCGTTA 17920

man CAGCTGATAGATGAAGAGAAACAACCACCACAAGGCGTTA 17920

uni CAGCTGATAGATGAAGAGAAACAACCACCACAAGGCGTTA 17920

tall CAGCTGATAGATGAAGAGAAACAACCACCACAAGGCGTTA 17920

quil CAGCTGATAGATGAAGAGAAACAACCACCACAAGGCGTTA 17886

meri CAGCTGATAGATGAAGAGAAACAACCACCACAAGGCGTTA 18042

ref CAGCTGATAGATGAAGAGAAACAACCACCACAAGGCGTTA 18168

Consensus cagctgatagatgaagagaaacaaccaccacaaggcgtta

kal AATCCAAAATTGTTGCAAAAAAGTCCAATTTGAGCTTTCC 17960

yor AATCCAAAATTGTTGCAAAAAAGTCCAATTTGAGCTTTCC 17944

jen AATCCAAAATTGTTGCAAAAAAGTCCAATTTGAGCTTTCC 17960

cor AATCCAAAATTGTTGCAAAAAAGTCCAATTTGAGCTTTCC 17960

man AATCCAAAATTGTTGCAAAAAAGTCCAATTTGAGCTTTCC 17960

uni AATCCAAAATTGTTGCAAAAAAGTCCAATTTGAGCTTTCC 17960

tall AATCCAAAATTGTTGCAAAAAAGTCCAATTTGAGCTTTCC 17960

quil AATCCAAAATTGTTGCAAAAAAGTCCAATTTGAGCTTTCC 17926

meri AATCCAAAATTGTTGCAAAAAAGTCCAATTTGAGCTTTCC 18082

ref AATCCAAAATTGTTGCAAAAAAGTCCAATTTGAGCTTTCC 18208

Consensus aatccaaaattgttgcaaaaaagtccaatttgagctttcc

kal TTCAAAACTAAAATCTCAGGGAAAGAGGAAAGCGGATTCA 18000

yor TTCAAAACTAAAATCTCAGGGAAAGAGGAAAGCGGATTCA 17984

jen TTCAAAACTAAAATCTCAGGGAAAGAGGAAAGCGGATTCA 18000

cor TTCAAAACTAAAATCTCAGGGAAAGAGGAAAGCGGATTCA 18000

man TTCAAAACTAAAATCTCAGGGAAAGAGGAAAGCGGATTCA 18000

uni TTCAAAACTAAAATCTCAGGGAAAGAGGAAAGCGGATTCA 18000

tall TTCAAAACTAAAATCTCAGGGAAAGAGGAAAGCGGATTCA 18000

quil TTCAAAACTAAAATCTCAGGGAAAGAGGAAAGCGGATTCA 17966

meri TTCAAAACTAAAATCTCAGGGAAAGAGGAAAGCGGATTCA 18122

ref TTCAAAACTAAAATCTCAGGGAAAGAGGAAAGCGGATTCA 18248

Consensus ttcaaaactaaaatctcagggaaagaggaaagcggattca

kal GAGGCATTTTCTGACATAGAAGCATATGACAGTCCTACAG 18040

yor GAGGCATTTTCTGACATAGAAGCATATGACAGTCCTACAG 18024

jen GAGGCATTTTCTGACATAGAAGCATATGACAGTCCTACAG 18040

cor GAGGCATTTTCTGACATAGAAGCATATGACAGTCCTACAG 18040

man GAGGCATTTTCTGACATAGAAGCATATGACAGTCCTACAG 18040

uni GAGGCATTTTCTGACATAGAAGCATATGACAGTCCTACAG 18040

tall GAGGCATTTTCTGACATAGAAGCATATGACAGTCCTACAG 18040

quil GAGGCATTTTCTGACATAGAAGCATATGACAGTCCTACAG 18006

meri GAGGCATTTTCTGACATAGAAGCATATGACAGTCCTACAG 18162

ref GAGGCATTTTCTGACATAGAAGCATATGACAGTCCTACAG 18288

Consensus gaggcattttctgacatagaagcatatgacagtcctacag

kal AAGTTGATCCAAATACACGCATTGTTGTATCAACCAAGTG 18080

yor AAGTTGATCCAAATACACGCATTGTTGTATCAACCAAGTG 18064

jen AAGTTGATCCAAATACACGCATTGTTGTATCAACCAAGTG 18080

cor AAGTTGATCCAAATACACGCATTGTTGTATCAACCAAGTG 18080

man AAGTTGATCCAAATACACGCATTGTTGTATCAACCAAGTG 18080

uni AAGTTGATCCAAATACACGCATTGTTGTATCAACCAAGTG 18080

tall AAGTTGATCCAAATACACGCATTGTTGTATCAACCAAGTG 18080

quil AAGTTGATCCAAATACACGCATTGTTGTATCAACCAAGTG 18046

meri AAGTTGATCCAAATACACGCATTGTTGTATCAACCAAGTG 18202

ref AAGTTGATCCAAATACACGCATTGTTGTATCAACCAAGTG 18328

Consensus aagttgatccaaatacacgcattgttgtatcaaccaagtg

kal GAAGCAGGCCAGTACTGGTACTGCCAGTTTAAATGAGTCC 18120

yor GAAGCAGGCCAGTACTGGTACTGCCAGTTTAAATGAGTCC 18104

jen GAAGCAGGCCAGTACTGGTACTGCCAGTTTAAATGAGTCC 18120

cor GAAGCAGGCCAGTACTGGTACTGCCAGTTTAAATGAGTCC 18120

man GAAGCAGGCCAGTACTGGTACTGCCAGTTTAAATGAGTCC 18120

uni GAAGCAGGCCAGTACTGGTACTGCCAGTTTAAATGAGTCC 18120

tall GAAGCAGGCCAGTACTGGTACTGCCAGTTTAAATGAGTCC 18120

quil GAAGCAGGCCAGTACTGGTACTGCCAGTTTAAATGAGTCC 18086

meri GAAGCAGGCCAGTACTGGTACTGCCAGTTTAAATGAGTCC 18242

ref GAAGCAGGCCAGTACTGGTACTGCCAGTTTAAATGAGTCC 18368

Consensus gaagcaggccagtactggtactgccagtttaaatgagtcc

kal AAGATTACTGAAAGAGCTGTTCAGCCTGTCCCAGATGAAA 18160

yor AAGATTACTGAAAGAGCTGTTCAGCCTGTCCCAGATGAAA 18144

jen AAGATTACTGAAAGAGCTGTTCAGCCTGTCCCAGATGAAA 18160

cor AAGATTACTGAAAGAGCTGTTCAGCCTGTCCCAGATGAAA 18160

man AAGATTACTGAAAGAGCTGTTCAGCCTGTCCCAGATGAAA 18160

uni AAGATTACTGAAAGAGCTGTTCAGCCTGTCCCAGATGAAA 18160

tall AAGATTACTGAAAGAGCTGTTCAGCCTGTCCCAGATGAAA 18160

quil AAGATTACTGAAAGAGCTGTTCAGCCTGTCCCAGATGAAA 18126

meri AAGATTACTGAAAGAGCTGTTCAGCCTGTCCCAGATGAAA 18282

ref AAGATTACTGAAAGAGCTGTTCAGCCTGTCCCAGATGAAA 18408

Consensus aagattactgaaagagctgttcagcctgtcccagatgaaa

kal CTGAATGTCGTGCTATTGTTCCTTACATCATTGAAGCACA 18200

yor CTGAATGTCGTGCTATTGTTCCTTACATCATTGAAGCACA 18184

jen CTGAATGTCGTGCTATTGTTCCTTACATCATTGAAGCACA 18200

cor CTGAATGTCGTGCTATTGTTCCTTACATCATTGAAGCACA 18200

man CTGAATGTCGTGCTATTGTTCCTTACATCATTGAAGCACA 18200

uni CTGAATGTCGTGCTATTGTTCCTTACATCATTGAAGCACA 18200

tall CTGAATGTCGTGCTATTGTTCCTTACATCATTGAAGCACA 18200

quil CTGAATGTCGTGCTATTGTTCCTTACATCATTGAAGCACA 18166

meri CTGAATGTCGTGCTATTGTTCCTTACATCATTGAAGCACA 18322

ref CTGAATGTCGTGCTATTGTTCCTTACATCATTGAAGCACA 18448

Consensus ctgaatgtcgtgctattgttccttacatcattgaagcaca

kal TGACAATCCTACAGAAGTTGATCCAAGTACATTGTTAGTA 18240

yor TGACAATCCTACAGAAGTTGATCCAAGTACATTGTTAGTA 18224

jen TGACAATCCTACAGAAGTTGATCCAAGTACATTGTTAGTA 18240

cor TGACAATCCTACAGAAGTTGATCCAAGTACATTGTTAGTA 18240

man TGACAATCCTACAGAAGTTGATCCAAGTACATTGTTAGTA 18240

uni TGACAATCCTACAGAAGTTGATCCAAGTACATTGTTAGTA 18240

tall TGACAATCCTACAGAAGTTGATCCAAGTACATTGTTAGTA 18240

quil TGACAATCCTACAGAAGTTGATCCAAGTACATTGTTAGTA 18206

meri TGACAATCCTACAGAAGTTGATCCAAGTACATTGTTAGTA 18362

ref TGACAATCCTACAGAAGTTGATCCAAGTACATTGTTAGTA 18488

Consensus tgacaatcctacagaagttgatccaagtacattgttagta

kal GCATCAACGAAGAGGGAGTGGGAATCTAAAACCAGCAGTG 18280

yor GCATCAACGAAGAGGGAGTGGGAATCTAAAACCAGCAGTG 18264

jen GCATCAACGAAGAGGGAGTGGGAATCTAAAACCAGCAGTG 18280

cor GCATCAACGAAGAGGGAGTGGGAATCTAAAACCAGCAGTG 18280

man GCATCAACGAAGAGGGAGTGGGAATCTAAAACCAGCAGTG 18280

uni GCATCAACGAAGAGGGAGTGGGAATCTAAAACCAGCAGTG 18280

tall GCATCAACGAAGAGGGAGTGGGAATCTAAAACCAGCAGTG 18280

quil GCATCAACGAAGAGGGAGTGGGAATCTAAAACCAGCAGTG 18246

meri GCATCAACGAAGAGGGAGTGGGAATCTAAAACCAGCAGTG 18402

ref GCATCAACGAAGAGGGAGTGGGAATCTAAAACCAGCAGTG 18528

Consensus gcatcaacgaagagggagtgggaatctaaaaccagcagtg

kal GTACTGCCAGTTTAAATGAGTCCATGAATGGGAATATTAC 18320

yor GTACTGCCAGTTTAAATGAGTCCATGAATGGGAATATTAC 18304

jen GTACTGCCAGTTTAAATGAGTCCATGAATGGGAATATTAC 18320

cor GTACTGCCAGTTTAAATGAGTCCATGAATGGGAATATTAC 18320

man GTACTGCCAGTTTAAATGAGTCCATGAATGGGAATATTAC 18320

uni GTACTGCCAGTTTAAATGAGTCCATGAATGGGAATATTAC 18320

tall GTACTGCCAGTTTAAATGAGTCCATGAATGGGAATATTAC 18320

quil GTACTGCCAGTTTAAATGAGTCCATGAATGGGAATATTAC 18286

meri GTACTGCCAGTTTAAATGAGTCCATGAATGGGAATATTAC 18442

ref GTACTGCCAGTTTAAATGAGTCCATGAATGGGAATATTAC 18568

Consensus gtactgccagtttaaatgagtccatgaatgggaatattac

kal TAAAAGAGCGGTTCAACCTGGCCCAGATGGAAGTGAATGC 18360

yor TAAAAGAGCGGTTCAACCTGGCCCAGATGGAAGTGAATGC 18344

jen TAAAAGAGCGGTTCAACCTGGCCCAGATGGAAGTGAATGC 18360

cor TAAAAGAGCGGTTCAACCTGGCCCAGATGGAAGTGAATGC 18360

man TAAAAGAGCGGTTCAACCTGGCCCAGATGGAAGTGAATGC 18360

uni TAAAAGAGCGGTTCAACCTGGCCCAGATGGAAGTGAATGC 18360

tall TAAAAGAGCGGTTCAACCTGGCCCAGATGGAAGTGAATGC 18360

quil TAAAAGAGCGGTTCAACCTGGCCCAGATGGAAGTGAATGC 18326

meri TAAAAGAGCGGTTCAACCTGGCCCAGATGGAAGTGAATGC 18482

ref TAAAAGAGCGGTTCAACCTGGCCCAGATGGAAGTGAATGC 18608

Consensus taaaagagcggttcaacctggcccagatggaagtgaatgc

kal CGTGCTATTGTTCCTTACATCAGTGAAGCACGCGACAATC 18400

yor CGTGCTATTGTTCCTTACATCAGTGAAGCACGCGACAATC 18384

jen CGTGCTATTGTTCCTTACATCAGTGAAGCACGCGACAATC 18400

cor CGTGCTATTGTTCCTTACATCAGTGAAGCACGCGACAATC 18400

man CGTGCTATTGTTCCTTACATCAGTGAAGCACGCGACAATC 18400

uni CGTGCTATTGTTCCTTACATCAGTGAAGCACGCGACAATC 18400

tall CGTGCTATTGTTCCTTACATCAGTGAAGCACGCGACAATC 18400

quil CGTGCTATTGTTCCTTACATCAGTGAAGCACGCGACAATC 18366

meri CGTGCTATTGTTCCTTACATCAGTGAAGCACGCGACAATC 18522

ref CGTGCTATTGTTCCTTACATCAGTGAAGCACGCGACAATC 18648

Consensus cgtgctattgttccttacatcagtgaagcacgcgacaatc

kal CTACAGACGTTGATCCAAGTAAAATGTTAATAGTATCAAC 18440

yor CTACAGACGTTGATCCAAGTAAAATGTTAATAGTATCAAC 18424

jen CTACAGACGTTGATCCAAGTAAAATGTTAATAGTATCAAC 18440

cor CTACAGACGTTGATCCAAGTAAAATGTTAATAGTATCAAC 18440

man CTACAGACGTTGATCCAAGTAAAATGTTAATAGTATCAAC 18440

uni CTACAGACGTTGATCCAAGTAAAATGTTAATAGTATCAAC 18440

tall CTACAGACGTTGATCCAAGTAAAATGTTAATAGTATCAAC 18440

quil CTACAGACGTTGATCCAAGTAAAATGTTAATAGTATCAAC 18406

meri CTACAGACGTTGATCCAAGTAAAATGTTAATAGTATCAAC 18562

ref CTACAGACGTTGATCCAAGTAAAATGTTAATAGTATCAAC 18688

Consensus ctacagacgttgatccaagtaaaatgttaatagtatcaac

kal CAAGAGGCGGTGGAAATCTAAAACCAGTACTGTTACTACC 18480

yor CAAGAGGCGGTGGAAATCTAAAACCAGTACTGTTACTACC 18464

jen CAAGAGGCGGTGGAAATCTAAAACCAGTACTGTTACTACC 18480

cor CAAGAGGCGGTGGAAATCTAAAACCAGTACTGTTACTACC 18480

man CAAGAGGCGGTGGAAATCTAAAACCAGTACTGTTACTACC 18480

uni CAAGAGGCGGTGGAAATCTAAAACCAGTACTGTTACTACC 18480

tall CAAGAGGCGGTGGAAATCTAAAACCAGTACTGTTACTACC 18480

quil CAAGAGGCGGTGGAAATCTAAAACCAGTACTGTTACTACC 18446

meri CAAGAGGCGGTGGAAATCTAAAACCAGTACTGTTACTACC 18602

ref CAAGAGGCGGTGGAAATCTAAAACCAGTACTGTTACTACC 18728

Consensus caagaggcggtggaaatctaaaaccagtactgttactacc

kal ATTTTAAGCGAGTCTATGAACAGTAATATTACTAAAAGAG 18520

yor ATTTTAAGCGAGTCTATGAACAGTAATATTACTAAAAGAG 18504

jen ATTTTAAGCGAGTCTATGAACAGTAATATTACTAAAAGAG 18520

cor ATTTTAAGCGAGTCTATGAACAGTAATATTACTAAAAGAG 18520

man ATTTTAAGCGAGTCTATGAACAGTAATATTACTAAAAGAG 18520

uni ATTTTAAGCGAGTCTATGAACAGTAATATTACTAAAAGAG 18520

tall ATTTTAAGCGAGTCTATGAACAGTAATATTACTAAAAGAG 18520

quil ATTTTAAGCGAGTCTATGAACAGTAATATTACTAAAAGAG 18486

meri ATTTTAAGCGAGTCTATGAACAGTAATATTACTAAAAGAG 18642

ref ATTTTAAGCGAGTCTATGAACAGTAATATTACTAAAAGAG 18768

Consensus attttaagcgagtctatgaacagtaatattactaaaagag

kal CTGTTCACCCTGGCCTGGATGGAACTGAAGGTTGTGCTAT 18560

yor CTGTTCACCCTGGCCTGGATGGAACTGAAGGTTGTGCTAT 18544

jen CTGTTCACCCTGGCCTGGATGGAACTGAAGGTTGTGCTAT 18560

cor CTGTTCACCCTGGCCTGGATGGAACTGAAGGTTGTGCTAT 18560

man CTGTTCACCCTGGCCTGGATGGAACTGAAGGTTGTGCTAT 18560

uni CTGTTCACCCTGGCCTGGATGGAACTGAAGGTTGTGCTAT 18560

tall CTGTTCACCCTGGCCTGGATGGAACTGAAGGTTGTGCTAT 18560

quil CTGTTCACCCTGGCCTGGATGGAACTGAAGGTTGTGCTAT 18526

meri CTGTTCACCCTGGCCTGGATGGAACTGAAGGTTGTGCTAT 18682

ref CTGTTCACCCTGGCCTGGATGGAACTGAAGGTTGTGCTAT 18808

Consensus ctgttcaccctggcctggatggaactgaaggttgtgctat

kal TGTTCCTTACATTAGTGAAGCACATGGCAAACCTACAAAA 18600

yor TGTTCCTTACATTAGTGAAGCACATGGCAAACCTACAAAA 18584

jen TGTTCCTTACATTAGTGAAGCACATGGCAAACCTACAAAA 18600

cor TGTTCCTTACATTAGTGAAGCACATGGCAAACCTACAAAA 18600

man TGTTCCTTACATTAGTGAAGCACATGGCAAACCTACAAAA 18600

uni TGTTCCTTACATTAGTGAAGCACATGGCAAACCTACAAAA 18600

tall TGTTCCTTACATTAGTGAAGCACATGGCAAACCTACAAAA 18600

quil TGTTCCTTACATTAGTGAAGCACATGGCAAACCTACAAAA 18566

meri TGTTCCTTACATTAGTGAAGCACATGGCAAACCTACAAAA 18722

ref TGTTCCTTACATTAGTGAAGCACATGGCAAACCTACAAAA 18848

Consensus tgttccttacattagtgaagcacatggcaaacctacaaaa

kal GTTGATCCAAGTACATCCATAGTTGCATCATCTAAGAGAC 18640

yor GTTGATCCAAGTACATCCATAGTTGCATCATCTAAGAGAC 18624

jen GTTGATCCAAGTACATCCATAGTTGCATCATCTAAGAGAC 18640

cor GTTGATCCAAGTACATCCATAGTTGCATCATCTAAGAGAC 18640

man GTTGATCCAAGTACATCCATAGTTGCATCATCTAAGAGAC 18640

uni GTTGATCCAAGTACATCCATAGTTGCATCATCTAAGAGAC 18640

tall GTTGATCCAAGTACATCCATAGTTGCATCATCTAAGAGAC 18640

quil GTTGATCCAAGTACATCCATAGTTGCATCATCTAAGAGAC 18606

meri GTTGATCCAAGTACATCCATAGTTGCATCATCTAAGAGAC 18762

ref GTTGATCCAAGTACATCCATAGTTGCATCATCTAAGAGAC 18888

Consensus gttgatccaagtacatccatagttgcatcatctaagagac

kal AGTGCAAATCTAAAACCAGTACTGGCACTGCCAATTTAAA 18680

yor AGTGCAAATCTAAAACCAGTACTGGCACTGCCAATTTAAA 18664

jen AGTGCAAATCTAAAACCAGTACTGGCACTGCCAATTTAAA 18680

cor AGTGCAAATCTAAAACCAGTACTGGCACTGCCAATTTAAA 18680

man AGTGCAAATCTAAAACCAGTACTGGCACTGCCAATTTAAA 18680

uni AGTGCAAATCTAAAACCAGTACTGGCACTGCCAATTTAAA 18680

tall AGTGCAAATCTAAAACCAGTACTGGCACTGCCAATTTAAA 18680

quil AGTGCAAATCTAAAACCAGTACTGGCACTGCCAATTTAAA 18646

meri AGTGCAAATCTAAAACCAGTACTGGCACTGCCAATTTAAA 18802

ref AGTGCAAATCTAAAACCAGTACTGGCACTGCCAATTTAAA 18928

Consensus agtgcaaatctaaaaccagtactggcactgccaatttaaa

kal TGAGCCTATGAACAGGAAGACTACCAAAAGAGCAGAGCTT 18720

yor TGAGCCTATGAACAGGAAGACTACCAAAAGAGCAGAGCTT 18704

jen TGAGCCTATGAACAGGAAGACTACCAAAAGAGCAGAGCTT 18720

cor TGAGCCTATGAACAGGAAGACTACCAAAAGAGCAGAGCTT 18720

man TGAGCCTATGAACAGGAAGACTACCAAAAGAGCAGAGCTT 18720

uni TGAGCCTATGAACAGGAAGACTACCAAAAGAGCAGAGCTT 18720

tall TGAGCCTATGAACAGGAAGACTACCAAAAGAGCAGAGCTT 18720

quil TGAGCCTATGAACAGGAAGACTACCAAAAGAGCAGAGCTT 18686

meri TGAGCCTATGAACAGGAAGACTACCAAAAGAGCAGAGCTT 18842

ref TGAGCCTATGAACAGGAAGACTACCAAAAGAGCAGAGCTT 18968

Consensus tgagcctatgaacaggaagactaccaaaagagcagagctt

kal GGCTTGGATGGAACTGAAAGTCATTCAGTTGTTCCTTACA 18760

yor GGCTTGGATGGAACTGAAAGTCATTCAGTTGTTCCTTACA 18744

jen GGCTTGGATGGAACTGAAAGTCATTCAGTTGTTCCTTACA 18760

cor GGCTTGGATGGAACTGAAAGTCATTCAGTTGTTCCTTACA 18760

man GGCTTGGATGGAACTGAAAGTCATTCAGTTGTTCCTTACA 18760

uni GGCTTGGATGGAACTGAAAGTCATTCAGTTGTTCCTTACA 18760

tall GGCTTGGATGGAACTGAAAGTCATTCAGTTGTTCCTTACA 18760

quil GGCTTGGATGGAACTGAAAGTCATTCAGTTGTTCCTTACA 18726

meri GGCTTGGATGGAACTGAAAGTCATTCAGTTGTTCCTTACA 18882

ref GGCTTGGATGGAACTGAAAGTCATTCAGTTGTTCCTTACA 19008

Consensus ggcttggatggaactgaaagtcattcagttgttccttaca

kal ATAGTGAATTTTCCGAGTTACAAAAGAGAAGACTTTCAAA 18800

yor ATAGTGAATTTTCCGAGTTACAAAAGAGAAGACTTTCAAA 18784

jen ATAGTGAATTTTCCGAGTTACAAAAGAGAAGACTTTCAAA 18800

cor ATAGTGAATTTTCCGAGTTACAAAAGAGAAGACTTTCAAA 18800

man ATAGTGAATTTTCCGAGTTACAAAAGAGAAGACTTTCAAA 18800

uni ATAGTGAATTTTCCGAGTTACAAAAGAGAAGACTTTCAAA 18800

tall ATAGTGAATTTTCCGAGTTACAAAAGAGAAGACTTTCAAA 18800

quil ATAGTGAATTTTCCGAGTTACAAAAGAGAAGACTTTCAAA 18766

meri ATAGTGAATTTTCCGAGTTACAAAAGAGAAGACTTTCAAA 18922

ref ATAGTGAATTTTCCGAGTTACAAAAGAGAAGACTTTCAAA 19048

Consensus atagtgaattttccgagttacaaaagagaagactttcaaa

kal GTGGCCAATCACTGCGGAAATAGAAAATTCTACTGTTAAC 18840

yor GTGGCCAATCACTGCGGAAATAGAAAATTCTACTGTTAAC 18824

jen GTGGCCAATCACTGCGGAAATAGAAAATTCTACTGTTAAC 18840

cor GTGGCCAATCACTGCGGAAATAGAAAATTCTACTGTTAAC 18840

man GTGGCCAATCACTGCGGAAATAGAAAATTCTACTGTTAAC 18840

uni GTGGCCAATCACTGCGGAAATAGAAAATTCTACTGTTAAC 18840

tall GTGGCCAATCACTGCGGAAATAGAAAATTCTACTGTTAAC 18840

quil GTGGCCAATCACTGCGGAAATAGAAAATTCTACTGTTAAC 18806

meri GTGGCCAATCACTGCGGAAATAGAAAATTCTACTGTTAAC 18962

ref GTGGCCAATCACTGCGGAAATAGAAAATTCTACTGTTAAC 19088

Consensus gtggccaatcactgcggaaatagaaaattctactgttaac

kal TTGGACTCACCAAATCCAGATAGGGTTTCAATGGACAATG 18880

yor TTGGACTCACCAAATCCAGATAGGGTTTCAATGGACAATG 18864

jen TTGGACTCACCAAATCCAGATAGGGTTTCAATGGACAATG 18880

cor TTGGACTCACCAAATCCAGATAGGGTTTCAATGGACAATG 18880

man TTGGACTCACCAAATCCAGATAGGGTTTCAATGGACAATG 18880

uni TTGGACTCACCAAATCCAGATAGGGTTTCAATGGACAATG 18880

tall TTGGACTCACCAAATCCAGATAGGGTTTCAATGGACAATG 18880

quil TTGGACTCACCAAATCCAGATAGGGTTTCAATGGACAATG 18846

meri TTGGACTCACCAAATCCAGATAGGGTTTCAATGGACAATG 19002

ref TTGGACTCACCAAATCCAGATAGGGTTTCAATGGACAATG 19128

Consensus ttggactcaccaaatccagatagggtttcaatggacaatg

kal GAAGCCAGGTAGATCTTCATATTGTTGAGTCTTATTCCTT 18920

yor GAAGCCAGGTAGATCTTCATATTGTTGAGTCTTATTCCTT 18904

jen GAAGCCAGGTAGATCTTCATATTGTTGAGTCTTATTCCTT 18920

cor GAAGCCAGGTAGATCTTCATATTGTTGAGTCTTATTCCTT 18920

man GAAGCCAGGTAGATCTTCATATTGTTGAGTCTTATTCCTT 18920

uni GAAGCCAGGTAGATCTTCATATTGTTGAGTCTTATTCCTT 18920

tall GAAGCCAGGTAGATCTTCATATTGTTGAGTCTTATTCCTT 18920

quil GAAGCCAGGTAGATCTTCATATTGTTGAGTCTTATTCCTT 18886

meri GAAGCCAGGTAGATCTTCATATTGTTGAGTCTTATTCCTT 19042

ref GAAGCCAGGTAGATCTTCATATTGTTGAGTCTTATTCCTT 19168

Consensus gaagccaggtagatcttcatattgttgagtcttattcctt

kal GGAGGATTCCCATAATGAAACCACAGCTTCTCAGCTTATT 18960

yor GGAGGATTCCCATAATGAAACCACAGCTTCTCAGCTTATT 18944

jen GGAGGATTCCCATAATGAAACCACAGCTTCTCAGCTTATT 18960

cor GGAGGATTCCCATAATGAAACCACAGCTTCTCAGCTTATT 18960

man GGAGGATTCCCATAATGAAACCACAGCTTCTCAGCTTATT 18960

uni GGAGGATTCCCATAATGAAACCACAGCTTCTCAGCTTATT 18960

tall GGAGGATTCCCATAATGAAACCACAGCTTCTCAGCTTATT 18960

quil GGAGGATTCCCATAATGAAACCACAGCTTCTCAGCTTATT 18926

meri GGAGGATTCCCATAATGAAACCACAGCTTCTCAGCTTATT 19082

ref GGAGGATTCCCATAATGAAACCACAGCTTCTCAGCTTATT 19208

Consensus ggaggattcccataatgaaaccacagcttctcagcttatt

kal ACCTTGGAGAAGCTGAATTTGCCTGCTTTGAGGGCCATGG 19000

yor ACCTTGGAGAAGCTGAATTTGCCTGCTTTGAGGGCCATGG 18984

jen ACCTTGGAGAAGCTGAATTTGCCTGCTTTGAGGGCCATGG 19000

cor ACCTTGGAGAAGCTGAATTTGCCTGCTTTGAGGGCCATGG 19000

man ACCTTGGAGAAGCTGAATTTGCCTGCTTTGAGGGCCATGG 19000

uni ACCTTGGAGAAGCTGAATTTGCCTGCTTTGAGGGCCATGG 19000

tall ACCTTGGAGAAGCTGAATTTGCCTGCTTTGAGGGCCATGG 19000

quil ACCTTGGAGAAGCTGAATTTGCCTGCTTTGAGGGCCATGG 18966

meri ACCTTGGAGAAGCTGAATTTGCCTGCTTTGAGGGCCATGG 19122

ref ACCTTGGAGAAGCTGAATTTGCCTGCTTTGAGGGCCATGG 19248

Consensus accttggagaagctgaatttgcctgctttgagggccatgg

kal CAAAGCAATACGACTTGAAAAAGTACTATAAGCTACGAAA 19040

yor CAAAGCAATACGACTTGAAAAAGTACTATAAGCTACGAAA 19024

jen CAAAGCAATACGACTTGAAAAAGTACTATAAGCTACGAAA 19040

cor CAAAGCAATACGACTTGAAAAAGTACTATAAGCTACGAAA 19040

man CAAAGCAATACGACTTGAAAAAGTACTATAAGCTACGAAA 19040

uni CAAAGCAATACGACTTGAAAAAGTACTATAAGCTACGAAA 19040

tall CAAAGCAATACGACTTGAAAAAGTACTATAAGCTACGAAA 19040

quil CAAAGCAATACGACTTGAAAAAGTACTATAAGCTACGAAA 19006

meri CAAAGCAATACGACTTGAAAAAGTACTATAAGCTACGAAA 19162

ref CAAAGCAATACGACTTGAAAAAGTACTATAAGCTACGAAA 19288

Consensus caaagcaatacgacttgaaaaagtactataagctacgaaa

kal AGCACAGCTGCTTCAACAATTAGTTGAACGGATGAGTAAC 19080

yor AGCACAGCTGCTTCAACAATTAGTTGAACGGATGAGTAAC 19064

jen AGCACAGCTGCTTCAACAATTAGTTGAACGGATGAGTAAC 19080

cor AGCACAGCTGCTTCAACAATTAGTTGAACGGATGAGTAAC 19080

man AGCACAGCTGCTTCAACAATTAGTTGAACGGATGAGTAAC 19080

uni AGCACAGCTGCTTCAACAATTAGTTGAACGGATGAGTAAC 19080

tall AGCACAGCTGCTTCAACAATTAGTTGAACGGATGAGTAAC 19080

quil AGCACAGCTGCTTCAACAATTAGTTGAACGGATGAGTAAC 19046

meri AGCACAGCTGCTTCAACAATTAGTTGAACGGATGAGTAAC 19202

ref AGCACAGCTGCTTCAACAATTAGTTGAACGGATGAGTAAC 19328

Consensus agcacagctgcttcaacaattagttgaacggatgagtaac

kal TGCTGAGCTTGCTATTGGGCTTAATGGTGGGTAGTGGATT 19120

yor TGCTGAGCTTGCTATTGGGCTTAATGGTGGGTAGTGGATT 19104

jen TGCTGAGCTTGCTATTGGGCTTAATGGTGGGTAGTGGATT 19120

cor TGCTGAGCTTGCTATTGGGCTTAATGGTGGGTAGTGGATT 19120

man TGCTGAGCTTGCTATTGGGCTTAATGGTGGGTAGTGGATT 19120

uni TGCTGAGCTTGCTATTGGGCTTAATGGTGGGTAGTGGATT 19120

tall TGCTGAGCTTGCTATTGGGCTTAATGGTGGGTAGTGGATT 19120

quil TGCTGAGCTTGCTATTGGGCTTAATGGTGGGTAGTGGATT 19086

meri TGCTGAGCTTGCTATTGGGCTTAATGGTGGGTAGTGGATT 19242

ref TGCTGAGCTTGCTATTGGGCTTAATGGTGGGTAGTGGATT 19368

Consensus tgctgagcttgctattgggcttaatggtgggtagtggatt

kal TATGCAGTGCTTTTTGGGGTATCTTTTTTGGTTTTGTTCA 19160

yor TATGCAGTGCTTTTTGGGGTATCTTTTTTGGTTTTGTTCA 19144

jen TATGCAGTGCTTTTTGGGGTATCTTTTTTGGTTTTGTTCA 19160

cor TATGCAGTGCTTTTTGGGGTATCTTTTTTGGTTTTGTTCA 19160

man TATGCAGTGCTTTTTGGGGTATCTTTTTTGGTTTTGTTCA 19160

uni TATGCAGTGCTTTTTGGGGTATCTTTTTTGGTTTTGTTCA 19160

tall TATGCAGTGCTTTTTGGGGTATCTTTTTTGGTTTTGTTCA 19160

quil TATGCAGTGCTTTTTGGGGTATCTTTTTTGGTTTTGTTCA 19126

meri TATGCAGTGCTTTTTGGGGTATCTTTTTTGGTTTTGTTCA 19282

ref TATGCAGTGCTTTTTGGGGTATCTTTTTTGGTTTTGTTCA 19408

Consensus tatgcagtgctttttggggtatcttttttggttttgttca

kal GTTCTTGCATGGAATTTGGTATAGGATGCTGACAGTTATG 19200

yor GTTCTTGCATGGAATTTGGTATAGGATGCTGACAGTTATG 19184

jen GTTCTTGCATGGAATTTGGTATAGGATGCTGACAGTTATG 19200

cor GTTCTTGCATGGAATTTGGTATAGGATGCTGACAGTTATG 19200

man GTTCTTGCATGGAATTTGGTATAGGATGCTGACAGTTATG 19200

uni GTTCTTGCATGGAATTTGGTATAGGATGCTGACAGTTATG 19200

tall GTTCTTGCATGGAATTTGGTATAGGATGCTGACAGTTATG 19200

quil GTTCTTGCATGGAATTTGGTATAGGATGCTGACAGTTATG 19166

meri GTTCTTGCATGGAATTTGGTATAGGATGCTGACAGTTATG 19322

ref GTTCTTGCATGGAATTTGGTATAGGATGCTGACAGTTATG 19448

Consensus gttcttgcatggaatttggtataggatgctgacagttatg

kal GATAATAGATAGGCAAACCAAAAGGGGGGTTTTGTGACAG 19240

yor GATAATAGATAGGCAAACCAAAAGGGGGGTTTTGTGACAG 19224

jen GATAATAGATAGGCAAACCAAAAGGGGGGTTTTGTGACAG 19240

cor GATAATAGATAGGCAAACCAAAAGGGGGGTTTTGTGACAG 19240

man GATAATAGATAGGCAAACCAAAAGGGGGGTTTTGTGACAG 19240

uni GATAATAGATAGGCAAACCAAAAGGGGGGTTTTGTGACAG 19240

tall GATAATAGATAGGCAAACCAAAAGGGGGGTTTTGTGACAG 19240

quil GATAATAGATAGGCAAACCAAAAGGGGGGTTTTGTGACAG 19206

meri GATAATAGATAGGCAAACCAAAAGGGGGGTTTTGTGACAG 19362

ref GATAATAGATAGGCAAACCAAAAGGGGGGTTTTGTGACAG 19488

Consensus gataatagataggcaaaccaaaaggggggttttgtgacag

kal GATATTGTACAGCCTATAGATAGTAGAATACTAGGGGTAC 19280

yor GATATTGTACAGCCTATAGATAGTAGAATACTAGGGGTAC 19264

jen GATATTGTACAGCCTATAGATAGTAGAATACTAGGGGTAC 19280

cor GATATTGTACAGCCTATAGATAGTAGAATACTAGGGGTAC 19280

man GATATTGTACAGCCTATAGATAGTAGAATACTAGGGGTAC 19280

uni GATATTGTACAGCCTATAGATAGTAGAATACTAGGGGTAC 19280

tall GATATTGTACAGCCTATAGATAGTAGAATACTAGGGGTAC 19280

quil GATATTGTACAGCCTATAGATAGTAGAATACTAGGGGTAC 19246

meri GATATTGTACAGCCTATAGATAGTAGAATACTAGGGGTAC 19402

ref GATATTGTACAGCCTATAGATAGTAGAATACTAGGGGTAC 19528

Consensus gatattgtacagcctatagatagtagaatactaggggtac

kal TTTTGTAAATGAAAGGAAGTATAGGTTTTCTTCCTTGTAA 19320

yor TTTTGTAAATGAAAGGAAGTATAGGTTTTCTTCCTTGTAA 19304

jen TTTTGTAAATGAAAGGAAGTATAGGTTTTCTTCCTTGTAA 19320

cor TTTTGTAAATGAAAGGAAGTATAGGTTTTCTTCCTTGTAA 19320

man TTTTGTAAATGAAAGGAAGTATAGGTTTTCTTCCTTGTAA 19320

uni TTTTGTAAATGAAAGGAAGTATAGGTTTTCTTCCTTGTAA 19320

tall TTTTGTAAATGAAAGGAAGTATAGGTTTTCTTCCTTGTAA 19320

quil TTTTGTAAATGAAAGGAAGTATAGGTTTTCTTCCTTGTAA 19286

meri TTTTGTAAATGAAAGGAAGTATAGGTTTTCTTCCTTGTAA 19442

ref TTTTGTAAATGAAAGGAAGTATAGGTTTTCTTCCTTGTAA 19568

Consensus ttttgtaaatgaaaggaagtataggttttcttccttgtaa

kal CTCTGGTTAGCCTTGGATGTTTCATGGGAAGACTCAGAAA 19360

yor CTCTGGTTAGCCTTGGATGTTTCATGGGAAGACTCAGAAA 19344

jen CTCTGGTTAGCCTTGGATGTTTCATGGGAAGACTCAGAAA 19360

cor CTCTGGTTAGCCTTGGATGTTTCATGGGAAGACTCAGAAA 19360

man CTCTGGTTAGCCTTGGATGTTTCATGGGAAGACTCAGAAA 19360

uni CTCTGGTTAGCCTTGGATGTTTCATGGGAAGACTCAGAAA 19360

tall CTCTGGTTAGCCTTGGATGTTTCATGGGAAGACTCAGAAA 19360

quil CTCTGGTTAGCCTTGGATGTTTCATGGGAAGACTCAGAAA 19326

meri CTCTGGTTAGCCTTGGATGTTTCATGGGAAGACTCAGAAA 19482

ref CTCTGGTTAGCCTTGGATGTTTCATGGGAAGACTCAGAAA 19608

Consensus ctctggttagccttggatgtttcatgggaagactcagaaa

kal CTTTGGTGATTACATTCTTGTGTTGCTGAGATCCATTATG 19400

yor CTTTGGTGATTACATTCTTGTGTTGCTGAGATCCATTATG 19384

jen CTTTGGTGATTACATTCTTGTGTTGCTGAGATCCATTATG 19400

cor CTTTGGTGATTACATTCTTGTGTTGCTGAGATCCATTATG 19400

man CTTTGGTGATTACATTCTTGTGTTGCTGAGATCCATTATG 19400

uni CTTTGGTGATTACATTCTTGTGTTGCTGAGATCCATTATG 19400

tall CTTTGGTGATTACATTCTTGTGTTGCTGAGATCCATTATG 19400

quil CTTTGGTGATTACATTCTTGTGTTGCTGAGATCCATTATG 19366

meri CTTTGGTGATTACATTCTTGTGTTGCTGAGATCCATTATG 19522

ref CTTTGGTGATTACATTCTTGTGTTGCTGAGATCCATTATG 19648

Consensus ctttggtgattacattcttgtgttgctgagatccattatg

kal GTGAATGATGATACATATTTTTAAATATGCGAAACAAAAA 19440

yor GTGAATGATGATACATATTTTTAAATATGCGAAACAAAAA 19424

jen GTGAATGATGATACATATTTTTAAATATGCGAAACAAAAA 19440

cor GTGAATGATGATACATATTTTTAAATATGCGAAACAAAAA 19440

man GTGAATGATGATACATATTTTTAAATATGCGAAACAAAAA 19440

uni GTGAATGATGATACATATTTTTAAATATGCGAAACAAAAA 19440

tall GTGAATGATGATACATATTTTTAAATATGCGAAACAAAAA 19440

quil GTGAATGATGATACATATTTTTAAATATGCGAAACAAAAA 19406

meri GTGAATGATGATACATATTTTTAAATATGCGAAACAAAAA 19562

ref GTGAATGATGATACATATTTTTAAATATGCGAAACAAAAA 19688

Consensus gtgaatgatgatacatatttttaaatatgcgaaacaaaaa

kal TCTCCGTCTAATGGCATATCACTTGCTATGCTAGATTGTG 19480

yor TCTCCGTCTAATGGCATATCACTTGCTATGCTAGATTGTG 19464

jen TCTCCGTCTAATGGCATATCACTTGCTATGCTAGATTGTG 19480

cor TCTCCGTCTAATGGCATATCACTTGCTATGCTAGATTGTG 19480

man TCTCCGTCTAATGGCATATCACTTGCTATGCTAGATTGTG 19480

uni TCTCCGTCTAATGGCATATCACTTGCTATGCTAGATTGTG 19480

tall TCTCCGTCTAATGGCATATCACTTGCTATGCTAGATTGTG 19480

quil TCTCCGTCTAATGGCATATCACTTGCTATGCTAGATTGTG 19446

meri TCTCCGTCTAATGGCATATCACTTGCTATGCTAGATTGTG 19602

ref TCTCCGTCTAATGGCATATCACTTGCTATGCTAGATTGTG 19728

Consensus tctccgtctaatggcatatcacttgctatgctagattgtg

kal TATGCTTCATGAACATGAAGAGCGATGAGATGTTTAACTT 19520

yor TATGCTTCATGAACATGAAGAGCGATGAGATGTTTAACTT 19504

jen TATGCTTCATGAACATGAAGAGCGATGAGATGTTTAACTT 19520

cor TATGCTTCATGAACATGAAGAGCGATGAGATGTTTAACTT 19520

man TATGCTTCATGAACATGAAGAGCGATGAGATGTTTAACTT 19520

uni TATGCTTCATGAACATGAAGAGCGATGAGATGTTTAACTT 19520

tall TATGCTTCATGAACATGAAGAGCGATGAGATGTTTAACTT 19520

quil TATGCTTCATGAACATGAAGAGCGATGAGATGTTTAACTT 19486

meri TATGCTTCATGAACATGAAGAGCGATGAGATGTTTAACTT 19642

ref TATGCTTCATGAACATGAAGAGCGATGAGATGTTTAACTT 19768

Consensus tatgcttcatgaacatgaagagcgatgagatgtttaactt

kal GGGTAGTTTTGTGTTCGGGATGACTATCATATATGTGGAA 19560

yor GGGTAGTTTTGTGTTCGGGATGACTATCATATATGTGGAA 19544

jen GGGTAGTTTTGTGTTCGGGATGACTATCATATATGTGGAA 19560

cor GGGTAGTTTTGTGTTCGGGATGACTATCATATATGTGGAA 19560

man GGGTAGTTTTGTGTTCGGGATGACTATCATATATGTGGAA 19560

uni GGGTAGTTTTGTGTTCGGGATGACTATCATATATGTGGAA 19560

tall GGGTAGTTTTGTGTTCGGGATGACTATCATATATGTGGAA 19560

quil GGGTAGTTTTGTGTTCGGGATGACTATCATATATGTGGAA 19526

meri GGGTAGTTTTGTGTTCGGGATGACTATCATATATGTGGAA 19682

ref GGGTAGTTTTGTGTTCGGGATGACTATCATATATGTGGAA 19808

Consensus gggtagttttgtgttcgggatgactatcatatatgtggaa

kal ATTCCATTTGTCCAAGTTCAATGTTGATTGCATGATGGTA 19600

yor ATTCCATTTGTCCAAGTTCAATGTTGATTGCATGATGGTA 19584

jen ATTCCATTTGTCCAAGTTCAATGTTGATTGCATGATGGTA 19600

cor ATTCCATTTGTCCAAGTTCAATGTTGATTGCATGATGGTA 19600

man ATTCCATTTGTCCAAGTTCAATGTTGATTGCATGATGGTA 19600

uni ATTCCATTTGTCCAAGTTCAATGTTGATTGCATGATGGTA 19600

tall ATTCCATTTGTCCAAGTTCAATGTTGATTGCATGATGGTA 19600

quil ATTCCATTTGTCCAAGTTCAATGTTGATTGCATGATGGTA 19566

meri ATTCCATTTGTCCAAGTTCAATGTTGATTGCATGATGGTA 19722

ref ATTCCATTTGTCCAAGTTCAATGTTGATTGCATGATGGTA 19848

Consensus attccatttgtccaagttcaatgttgattgcatgatggta

kal AACTCTCAAATATTAGCTTTTGAGTAAATTTCCAGCACCA 19640

yor AACTCTCAAATATTAGCTTTTGAGTAAATTTCCAGCACCA 19624

jen AACTCTCAAATATTAGCTTTTGAGTAAATTTCCAGCACCA 19640

cor AACTCTCAAATATTAGCTTTTGAGTAAATTTCCAGCACCA 19640

man AACTCTCAAATATTAGCTTTTGAGTAAATTTCCAGCACCA 19640

uni AACTCTCAAATATTAGCTTTTGAGTAAATTTCCAGCACCA 19640

tall AACTCTCAAATATTAGCTTTTGAGTAAATTTCCAGCACCA 19640

quil AACTCTCAAATATTAGCTTTTGAGTAAATTTCCAGCACCA 19606

meri AACTCTCAAATATTAGCTTTTGAGTAAATTTCCAGCACCA 19762

ref AACTCTCAAATATTAGCTTTTGAGTAAATTTCCAGCACCA 19888

Consensus aactctcaaatattagcttttgagtaaatttccagcacca

kal AGTTCTACTCTGTTTAGTTGATAAAGTCTAACATCATTTT 19680

yor AGTTCTACTCTGTTTAGTTGATAAAGTCTAACATCATTTT 19664

jen AGTTCTACTCTGTTTAGTTGATAAAGTCTAACATCATTTT 19680

cor AGTTCTACTCTGTTTAGTTGATAAAGTCTAACATCATTTT 19680

man AGTTCTACTCTGTTTAGTTGATAAAGTCTAACATCATTTT 19680

uni AGTTCTACTCTGTTTAGTTGATAAAGTCTAACATCATTTT 19680

tall AGTTCTACTCTGTTTAGTTGATAAAGTCTAACATCATTTT 19680

quil AGTTCTACTCTGTTTAGTTGATAAAGTCTAACATCATTTT 19646

meri AGTTCTACTCTGTTTAGTTGATAAAGTCTAACATCATTTT 19802

ref AGTTCTACTCTGTTTAGTTGATAAAGTCTAACATCATTTT 19928

Consensus agttctactctgtttagttgataaagtctaacatcatttt

kal TTTGCATAATTTACTACATCTAAATGATGTTATTGACCAT 19720

yor TTTGCATAATTTACTACATCTAAATGATGTTATTGACCAT 19704

jen TTTGCATAATTTACTACATCTAAATGATGTTATTGACCAT 19720

cor TTTGCATAATTTACTACATCTAAATGATGTTATTGACCAT 19720

man TTTGCATAATTTACTACATCTAAATGATGTTATTGACCAT 19720

uni TTTGCATAATTTACTACATCTAAATGATGTTATTGACCAT 19720

tall TTTGCATAATTTACTACATCTAAATGATGTTATTGACCAT 19720

quil TTTGCATAATTTACTACATCTAAATGATGTTATTGACCAT 19686

meri TTTGCATAATTTACTACATCTAAATGATGTTATTGACCAT 19842

ref TTTGCATAATTTACTACATCTAAATGATGTTATTGACCAT 19968

Consensus tttgcataatttactacatctaaatgatgttattgaccat

kal GTTGTAAGATTTCTCCTATGTGCACTGAACATTGCACAAT 19760

yor GTTGTAAGATTTCTCCTATGTGCACTGAACATTGCACAAT 19744

jen GTTGTAAGATTTCTCCTATGTGCACTGAACATTGCACAAT 19760

cor GTTGTAAGATTTCTCCTATGTGCACTGAACATTGCACAAT 19760

man GTTGTAAGATTTCTCCTATGTGCACTGAACATTGCACAAT 19760

uni GTTGTAAGATTTCTCCTATGTGCACTGAACATTGCACAAT 19760

tall GTTGTAAGATTTCTCCTATGTGCACTGAACATTGCACAAT 19760

quil GTTGTAAGATTTCTCCTATGTGCACTGAACATTGCACAAT 19726

meri GTTGTAAGATTTCTCCTATGTGCACTGAACATTGCACAAT 19882

ref GTTGTAAGATTTCTCCTATGTGCACTGAACATTGCACAAT 20008

Consensus gttgtaagatttctcctatgtgcactgaacattgcacaat

kal TTATCAAAGCTCAAATGCTAAACAAATAATTGTGTAGTAA 19800

yor TTATCAAAGCTCAAATGCTAAACAAATAATTGTGTAGTAA 19784

jen TTATCAAAGCTCAAATGCTAAACAAATAATTGTGTAGTAA 19800

cor TTATCAAAGCTCAAATGCTAAACAAATAATTGTGTAGTAA 19800

man TTATCAAAGCTCAAATGCTAAACAAATAATTGTGTAGTAA 19800

uni TTATCAAAGCTCAAATGCTAAACAAATAATTGTGTAGTAA 19800

tall TTATCAAAGCTCAAATGCTAAACAAATAATTGTGTAGTAA 19800

quil TTATCAAAGCTCAAATGCTAAACAAATAATTGTGTAGTAA 19766

meri TTATCAAAGCTCAAATGCTAAACAAATAATTGTGTAGTAA 19922

ref TTATCAAAGCTCAAATGCTAAACAAATAATTGTGTAGTAA 20048

Consensus ttatcaaagctcaaatgctaaacaaataattgtgtagtaa

kal ACTGGATATTCAAATGCATGCAGTATGTTCCATATTGCAT 19840

yor ACTGGATATTCAAATGCATGCAGTATGTTCCATATTGCAT 19824

jen ACTGGATATTCAAATGCATGCAGTATGTTCCATATTGCAT 19840

cor ACTGGATATTCAAATGCATGCAGTATGTTCCATATTGCAT 19840

man ACTGGATATTCAAATGCATGCAGTATGTTCCATATTGCAT 19840

uni ACTGGATATTCAAATGCATGCAGTATGTTCCATATTGCAT 19840

tall ACTGGATATTCAAATGCATGCAGTATGTTCCATATTGCAT 19840

quil ACTGGATATTCAAATGCATGCAGTATGTTCCATATTGCAT 19806

meri ACTGGATATTCAAATGCATGCAGTATGTTCCATATTGCAT 19962

ref ACTGGATATTCAAATGCATGCAGTATGTTCCATATTGCAT 20088

Consensus actggatattcaaatgcatgcagtatgttccatattgcat

kal TATAGAAATGTTAGTGAGGAATAGTTGAGCCGGTTAGGTG 19880

yor TATAGAAATGTTAGTGAGGAATAGTTGAGCCGGTTAGGTG 19864

jen TATAGAAATGTTAGTGAGGAATAGTTGAGCCGGTTAGGTG 19880

cor TATAGAAATGTTAGTGAGGAATAGTTGAGCCGGTTAGGTG 19880

man TATAGAAATGTTAGTGAGGAATAGTTGAGCCGGTTAGGTG 19880

uni TATAGAAATGTTAGTGAGGAATAGTTGAGCCGGTTAGGTG 19880

tall TATAGAAATGTTAGTGAGGAATAGTTGAGCCGGTTAGGTG 19880

quil TATAGAAATGTTAGTGAGGAATAGTTGAGCCGGTTAGGTG 19846

meri TATAGAAATGTTAGTGAGGAATAGTTGAGCCGGTTAGGTG 20002

ref TATAGAAATGTTAGTGAGGAATAGTTGAGCCGGTTAGGTG 20128

Consensus tatagaaatgttagtgaggaatagttgagccggttaggtg

kal ACTGTAATGATGCATTAACAGTACATGATACTTACACATT 19920

yor ACTGTAATGATGCATTAACAGTACATGATACTTACACATT 19904

jen ACTGTAATGATGCATTAACAGTACATGATACTTACACATT 19920

cor ACTGTAATGATGCATTAACAGTACATGATACTTACACATT 19920

man ACTGTAATGATGCATTAACAGTACATGATACTTACACATT 19920

uni ACTGTAATGATGCATTAACAGTACATGATACTTACACATT 19920

tall ACTGTAATGATGCATTAACAGTACATGATACTTACACATT 19920

quil ACTGTAATGATGCATTAACAGTACATGATACTTACACATT 19886

meri ACTGTAATGATGCATTAACAGTACATGATACTTACACATT 20042

ref ACTGTAATGATGCATTAACAGTACATGATACTTACACATT 20168

Consensus actgtaatgatgcattaacagtacatgatacttacacatt

kal TGTGTCAATGGTATCTGAAACTAGACAGGTTGTGATAGGA 19960

yor TGTGTCAATGGTATCTGAAACTAGACAGGTTGTGATAGGA 19944

jen TGTGTCAATGGTATCTGAAACTAGACAGGTTGTGATAGGA 19960

cor TGTGTCAATGGTATCTGAAACTAGACAGGTTGTGATAGGA 19960

man TGTGTCAATGGTATCTGAAACTAGACAGGTTGTGATAGGA 19960

uni TGTGTCAATGGTATCTGAAACTAGACAGGTTGTGATAGGA 19960

tall TGTGTCAATGGTATCTGAAACTAGACAGGTTGTGATAGGA 19960

quil TGTGTCAATGGTATCTGAAACTAGACAGGTTGTGATAGGA 19926

meri TGTGTCAATGGTATCTGAAACTAGACAGGTTGTGATAGGA 20082

ref TGTGTCAATGGTATCTGAAACTAGACAGGTTGTGATAGGA 20208

Consensus tgtgtcaatggtatctgaaactagacaggttgtgatagga

kal TCTTAAGGTGTGTCAGTTGTTATTAGACCTGTACCAAATG 20000

yor TCTTAAGGTGTGTCAGTTGTTATTAGACCTGTACCAAATG 19984

jen TCTTAAGGTGTGTCAGTTGTTATTAGACCTGTACCAAATG 20000

cor TCTTAAGGTGTGTCAGTTGTTATTAGACCTGTACCAAATG 20000

man TCTTAAGGTGTGTCAGTTGTTATTAGACCTGTACCAAATG 20000

uni TCTTAAGGTGTGTCAGTTGTTATTAGACCTGTACCAAATG 20000

tall TCTTAAGGTGTGTCAGTTGTTATTAGACCTGTACCAAATG 20000

quil TCTTAAGGTGTGTCAGTTGTTATTAGACCTGTACCAAATG 19966

meri TCTTAAGGTGTGTCAGTTGTTATTAGACCTGTACCAAATG 20122

ref TCTTAAGGTGTGTCAGTTGTTATTAGACCTGTACCAAATG 20248

Consensus tcttaaggtgtgtcagttgttattagacctgtaccaaatg

kal TTAGGCATAGCAGATGGGTGAATGTTGATCCCCATTGCAA 20040

yor TTAGGCATAGCAGATGGGTGAATGTTGATCCCCATTGCAA 20024

jen TTAGGCATAGCAGATGGGTGAATGTTGATCCCCATTGCAA 20040

cor TTAGGCATAGCAGATGGGTGAATGTTGATCCCCATTGCAA 20040

man TTAGGCATAGCAGATGGGTGAATGTTGATCCCCATTGCAA 20040

uni TTAGGCATAGCAGATGGGTGAATGTTGATCCCCATTGCAA 20040

tall TTAGGCATAGCAGATGGGTGAATGTTGATCCCCATTGCAA 20040

quil TTAGGCATAGCAGATGGGTGAATGTTGATCCCCATTGCAA 20006

meri TTAGGCATAGCAGATGGGTGAATGTTGATCCCCATTGCAA 20162

ref TTAGGCATAGCAGATGGGTGAATGTTGATCCCCATTGCAA 20288

Consensus ttaggcatagcagatgggtgaatgttgatccccattgcaa

kal CATGTGTTTGGGTTTGGAAGCATAGGTTCTACAAGTGCTG 20080

yor CATGTGTTTGGGTTTGGAAGCATAGGTTCTACAAGTGCTG 20064

jen CATGTGTTTGGGTTTGGAAGCATAGGTTCTACAAGTGCTG 20080

cor CATGTGTTTGGGTTTGGAAGCATAGGTTCTACAAGTGCTG 20080

man CATGTGTTTGGGTTTGGAAGCATAGGTTCTACAAGTGCTG 20080

uni CATGTGTTTGGGTTTGGAAGCATAGGTTCTACAAGTGCTG 20080

tall CATGTGTTTGGGTTTGGAAGCATAGGTTCTACAAGTGCTG 20080

quil CATGTGTTTGGGTTTGGAAGCATAGGTTCTACAAGTGCTG 20046

meri CATGTGTTTGGGTTTGGAAGCATAGGTTCTACAAGTGCTG 20202

ref CATGTGTTTGGGTTTGGAAGCATAGGTTCTACAAGTGCTG 20328

Consensus catgtgtttgggtttggaagcataggttctacaagtgctg

kal CATATCTTGGCACCTAAGTTCTGATTTATTTGGTGAAAGT 20120

yor CATATCTTGGCACCTAAGTTCTGATTTATTTGGTGAAAGT 20104

jen CATATCTTGGCACCTAAGTTCTGATTTATTTGGTGAAAGT 20120

cor CATATCTTGGCACCTAAGTTCTGATTTATTTGGTGAAAGT 20120

man CATATCTTGGCACCTAAGTTCTGATTTATTTGGTGAAAGT 20120

uni CATATCTTGGCACCTAAGTTCTGATTTATTTGGTGAAAGT 20120

tall CATATCTTGGCACCTAAGTTCTGATTTATTTGGTGAAAGT 20120

quil CATATCTTGGCACCTAAGTTCTGATTTATTTGGTGAAAGT 20086

meri CATATCTTGGCACCTAAGTTCTGATTTATTTGGTGAAAGT 20242

ref CATATCTTGGCACCTAAGTTCTGATTTATTTGGTGAAAGT 20368

Consensus catatcttggcacctaagttctgatttatttggtgaaagt

kal AAAGCCTCTTGGCAAGTGCATGAGGCTAGAACAGTAGAAC 20160

yor AAAGCCTCTTGGCAAGTGCATGAGGCTAGAACAGTAGAAC 20144

jen AAAGCCTCTTGGCAAGTGCATGAGGCTAGAACAGTAGAAC 20160

cor AAAGCCTCTTGGCAAGTGCATGAGGCTAGAACAGTAGAAC 20160

man AAAGCCTCTTGGCAAGTGCATGAGGCTAGAACAGTAGAAC 20160

uni AAAGCCTCTTGGCAAGTGCATGAGGCTAGAACAGTAGAAC 20160

tall AAAGCCTCTTGGCAAGTGCATGAGGCTAGAACAGTAGAAC 20160

quil AAAGCCTCTTGGCAAGTGCATGAGGCTAGAACAGTAGAAC 20126

meri AAAGCCTCTTGGCAAGTGCATGAGGCTAGAACAGTAGAAC 20282

ref AAAGCCTCTTGGCAAGTGCATGAGGCTAGAACAGTAGAAC 20408

Consensus aaagcctcttggcaagtgcatgaggctagaacagtagaac

kal TAGATCATCATATTGTTGAGTCTTATTCTTTGGTTGTCTT 20200

yor TAGATCATCATATTGTTGAGTCTTATTCTTTGGTTGTCTT 20184

jen TAGATCATCATATTGTTGAGTCTTATTCTTTGGTTGTCTT 20200

cor TAGATCATCATATTGTTGAGTCTTATTCTTTGGTTGTCTT 20200

man TAGATCATCATATTGTTGAGTCTTATTCTTTGGTTGTCTT 20200

uni TAGATCATCATATTGTTGAGTCTTATTCTTTGGTTGTCTT 20200

tall TAGATCATCATATTGTTGAGTCTTATTCTTTGGTTGTCTT 20200

quil TAGATCATCATATTGTTGAGTCTTATTCTTTGGTTGTCTT 20166

meri TAGATCATCATATTGTTGAGTCTTATTCTTTGGTTGTCTT 20322

ref TAGATCATCATATTGTTGAGTCTTATTCTTTGGTTGTCTT 20448

Consensus tagatcatcatattgttgagtcttattctttggttgtctt

kal TGTTTCTTCATCTCTCTTCTTACATTTATCATTAATATCT 20240

yor TGTTTCTTCATCTCTCTTCTTACATTTATCATTAATATCT 20224

jen TGTTTCTTCATCTCTCTTCTTACATTTATCATTAATATCT 20240

cor TGTTTCTTCATCTCTCTTCTTACATTTATCATTAATATCT 20240

man TGTTTCTTCATCTCTCTTCTTACATTTATCATTAATATCT 20240

uni TGTTTCTTCATCTCTCTTCTTACATTTATCATTAATATCT 20240

tall TGTTTCTTCATCTCTCTTCTTACATTTATCATTAATATCT 20240

quil TGTTTCTTCATCTCTCTTCTTACATTTATCATTAATATCT 20206

meri TGTTTCTTCATCTCTCTTCTTACATTTATCATTAATATCT 20362

ref TGTTTCTTCATCTCTCTTCTTACATTTATCATTAATATCT 20488

Consensus tgtttcttcatctctcttcttacatttatcattaatatct

kal ACCTCCTTCTACTCCATGTATGTATATTAATGGTGATGTT 20280

yor ACCTCCTTCTACTCCATGTATGTATATTAATGGTGATGTT 20264

jen ACCTCCTTCTACTCCATGTATGTATATTAATGGTGATGTT 20280

cor ACCTCCTTCTACTCCATGTATGTATATTAATGGTGATGTT 20280

man ACCTCCTTCTACTCCATGTATGTATATTAATGGTGATGTT 20280

uni ACCTCCTTCTACTCCATGTATGTATATTAATGGTGATGTT 20280

tall ACCTCCTTCTACTCCATGTATGTATATTAATGGTGATGTT 20280

quil ACCTCCTTCTACTCCATGTATGTATATTAATGGTGATGTT 20246

meri ACCTCCTTCTACTCCATGTATGTATATTAATGGTGATGTT 20402

ref ACCTCCTTCTACTCCATGTATGTATATTAATGGTGATGTT 20528

Consensus acctccttctactccatgtatgtatattaatggtgatgtt

kal CATTCACTAAGGATTGAAACCCATTCTACCACAATATTTT 20320

yor CATTCACTAAGGATTGAAACCCATTCTACCACAATATTTT 20304

jen CATTCACTAAGGATTGAAACCCATTCTACCACAATATTTT 20320

cor CATTCACTAAGGATTGAAACCCATTCTACCACAATATTTT 20320

man CATTCACTAAGGATTGAAACCCATTCTACCACAATATTTT 20320

uni CATTCACTAAGGATTGAAACCCATTCTACCACAATATTTT 20320

tall CATTCACTAAGGATTGAAACCCATTCTACCACAATATTTT 20320

quil CATTCACTAAGGATTGAAACCCATTCTACCACAATATTTT 20286

meri CATTCACTAAGGATTGAAACCCATTCTACCACAATATTTT 20442

ref CATTCACTAAGGATTGAAACCCATTCTACCACAATATTTT 20568

Consensus cattcactaaggattgaaacccattctaccacaatatttt

kal GTTTGCTTTATTACCAAGTTAATAGTAGAAAATAAATATC 20360

yor GTTTGCTTTATTACCAAGTTAATAGTAGAAAATAAATATC 20344

jen GTTTGCTTTATTACCAAGTTAATAGTAGAAAATAAATATC 20360

cor GTTTGCTTTATTACCAAGTTAATAGTAGAAAATAAATATC 20360

man GTTTGCTTTATTACCAAGTTAATAGTAGAAAATAAATATC 20360

uni GTTTGCTTTATTACCAAGTTAATAGTAGAAAATAAATATC 20360

tall GTTTGCTTTATTACCAAGTTAATAGTAGAAAATAAATATC 20360

quil GTTTGCTTTATTACCAAGTTAATAGTAGAAAATAAATATC 20326

meri GTTTGCTTTATTACCAAGTTAATAGTAGAAAATAAATATC 20482

ref GTTTGCTTTATTACCAAGTTAATAGTAGAAAATAAATATC 20608

Consensus gtttgctttattaccaagttaatagtagaaaataaatatc

kal TTCTTACTGTAAAGAACATGGTCTTAAAACAATGACTTTA 20400

yor TTCTTACTGTAAAGAACATGGTCTTAAAACAATGACTTTA 20384

jen TTCTTACTGTAAAGAACATGGTCTTAAAACAATGACTTTA 20400

cor TTCTTACTGTAAAGAACATGGTCTTAAAACAATGACTTTA 20400

man TTCTTACTGTAAAGAACATGGTCTTAAAACAATGACTTTA 20400

uni TTCTTACTGTAAAGAACATGGTCTTAAAACAATGACTTTA 20400

tall TTCTTACTGTAAAGAACATGGTCTTAAAACAATGACTTTA 20400

quil TTCTTACTGTAAAGAACATGGTCTTAAAACAATGACTTTA 20366

meri TTCTTACTGTAAAGAACATGGTCTTAAAACAATGACTTTA 20522

ref TTCTTACTGTAAAGAACATGGTCTTAAAACAATGACTTTA 20648

Consensus ttcttactgtaaagaacatggtcttaaaacaatgacttta

kal TAGTTAACCACTGAGTACACTTTTTGTAATCCTTACCCAA 20440

yor TAGTTAACCACTGAGTACACTTTTTGTAATCCTTACCCAA 20424

jen TAGTTAACCACTGAGTACACTTTTTGTAATCCTTACCCAA 20440

cor TAGTTAACCACTGAGTACACTTTTTGTAATCCTTACCCAA 20440

man TAGTTAACCACTGAGTACACTTTTTGTAATCCTTACCCAA 20440

uni TAGTTAACCACTGAGTACACTTTTTGTAATCCTTACCCAA 20440

tall TAGTTAACCACTGAGTACACTTTTTGTAATCCTTACCCAA 20440

quil TAGTTAACCACTGAGTACACTTTTTGTAATCCTTACCCAA 20406

meri TAGTTAACCACTGAGTACACTTTTTGTAATCCTTACCCAA 20562

ref TAGTTAACCACTGAGTACACTTTTTGTAATCCTTACCCAA 20688

Consensus tagttaaccactgagtacactttttgtaatccttacccaa

kal GATGCAATCTGTGAAGTGATCAACTCAAGCAATTGTGATA 20480

yor GATGCAATCTGTGAAGTGATCAACTCAAGCAATTGTGATA 20464

jen GATGCAATCTGTGAAGTGATCAACTCAAGCAATTGTGATA 20480

cor GATGCAATCTGTGAAGTGATCAACTCAAGCAATTGTGATA 20480

man GATGCAATCTGTGAAGTGATCAACTCAAGCAATTGTGATA 20480

uni GATGCAATCTGTGAAGTGATCAACTCAAGCAATTGTGATA 20480

tall GATGCAATCTGTGAAGTGATCAACTCAAGCAATTGTGATA 20480

quil GATGCAATCTGTGAAGTGATCAACTCAAGCAATTGTGATA 20446

meri GATGCAATCTGTGAAGTGATCAACTCAAGCAATTGTGATA 20602

ref GATGCAATCTGTGAAGTGATCAACTCAAGCAATTGTGATA 20728

Consensus gatgcaatctgtgaagtgatcaactcaagcaattgtgata

kal ACATGCTAAATCCAAGTACTTTATATAATGGTGAAAACAA 20520

yor ACATGCTAAATCCAAGTACTTTATATAATGGTGAAAACAA 20504

jen ACATGCTAAATCCAAGTACTTTATATAATGGTGAAAACAA 20520

cor ACATGCTAAATCCAAGTACTTTATATAATGGTGAAAACAA 20520

man ACATGCTAAATCCAAGTACTTTATATAATGGTGAAAACAA 20520

uni ACATGCTAAATCCAAGTACTTTATATAATGGTGAAAACAA 20520

tall ACATGCTAAATCCAAGTACTTTATATAATGGTGAAAACAA 20520

quil ACATGCTAAATCCAAGTACTTTATATAATGGTGAAAACAA 20486

meri ACATGCTAAATCCAAGTACTTTATATAATGGTGAAAACAA 20642

ref ACATGCTAAATCCAAGTACTTTATATAATGGTGAAAACAA 20768

Consensus acatgctaaatccaagtactttatataatggtgaaaacaa

kal TGTGCACAGGTTTTTAGATGTAGATATCTATATATGTACT 20560

yor TGTGCACAGGTTTTTAGATGTAGATATCTATATATGTACT 20544

jen TGTGCACAGGTTTTTAGATGTAGATATCTATATATGTACT 20560

cor TGTGCACAGGTTTTTAGATGTAGATATCTATATATGTACT 20560

man TGTGCACAGGTTTTTAGATGTAGATATCTATATATGTACT 20560

uni TGTGCACAGGTTTTTAGATGTAGATATCTATATATGTACT 20560

tall TGTGCACAGGTTTTTAGATGTAGATATCTATATATGTACT 20560

quil TGTGCACAGGTTTTTAGATGTAGATATCTATATATGTACT 20526

meri TGTGCACAGGTTTTTAGATGTAGATATCTATATATGTACT 20682

ref TGTGCACAGGTTTTTAGATGTAGATATCTATATATGTACT 20808

Consensus tgtgcacaggtttttagatgtagatatctatatatgtact

kal TAGCTTTAGTATTGCTCAAGCACACTCACTAGCACACATA 20600

yor TAGCTTTAGTATTGCTCAAGCACACTCACTAGCACACATA 20584

jen TAGCTTTAGTATTGCTCAAGCACACTCACTAGCACACATA 20600

cor TAGCTTTAGTATTGCTCAAGCACACTCACTAGCACACATA 20600

man TAGCTTTAGTATTGCTCAAGCACACTCACTAGCACACATA 20600

uni TAGCTTTAGTATTGCTCAAGCACACTCACTAGCACACATA 20600

tall TAGCTTTAGTATTGCTCAAGCACACTCACTAGCACACATA 20600

quil TAGCTTTAGTATTGCTCAAGCACACTCACTAGCACACATA 20566

meri TAGCTTTAGTATTGCTCAAGCACACTCACTAGCACACATA 20722

ref TAGCTTTAGTATTGCTCAAGCACACTCACTAGCACACATA 20848

Consensus tagctttagtattgctcaagcacactcactagcacacata

kal TGATACCCCAGTTGGAGGACACAGCCAGTAGAAGAAACAG 20640

yor TGATACCCCAGTTGGAGGACACAGCCAGTAGAAGAAACAG 20624

jen TGATACCCCAGTTGGAGGACACAGCCAGTAGAAGAAACAG 20640

cor TGATACCCCAGTTGGAGGACACAGCCAGTAGAAGAAACAG 20640

man TGATACCCCAGTTGGAGGACACAGCCAGTAGAAGAAACAG 20640

uni TGATACCCCAGTTGGAGGACACAGCCAGTAGAAGAAACAG 20640

tall TGATACCCCAGTTGGAGGACACAGCCAGTAGAAGAAACAG 20640

quil TGATACCCCAGTTGGAGGACACAGCCAGTAGAAGAAACAG 20606

meri TGATACCCCAGTTGGAGGACACAGCCAGTAGAAGAAACAG 20762

ref TGATACCCCAGTTGGAGGACACAGCCAGTAGAAGAAACAG 20888

Consensus tgataccccagttggaggacacagccagtagaagaaacag

kal CATGCATGGACACAGTTCAGTTCAGTTCAGATGCACATTC 20680

yor CATGCATGGACACAGTTCAGTTCAGTTCAGATGCACATTC 20664

jen CATGCATGGACACAGTTCAGTTCAGTTCAGATGCACATTC 20680

cor CATGCATGGACACAGTTCAGTTCAGTTCAGATGCACATTC 20680

man CATGCATGGACACAGTTCAGTTCAGTTCAGATGCACATTC 20680

uni CATGCATGGACACAGTTCAGTTCAGTTCAGATGCACATTC 20680

tall CATGCATGGACACAGTTCAGTTCAGTTCAGATGCACATTC 20680

quil CATGCATGGACACAGTTCAGTTCAGTTCAGATGCACATTC 20646

meri CATGCATGGACACAGTTCAGTTCAGTTCAGATGCACATTC 20802

ref CATGCATGGACACAGTTCAGTTCAGTTCAGATGCACATTC 20928

Consensus catgcatggacacagttcagttcagttcagatgcacattc

kal TACTGTCTCAACAAAAATAAACTACAACTTTAAATTTTAA 20720

yor TACTGTCTCAACAAAAATAAACTACAACTTTAAATTTTAA 20704

jen TACTGTCTCAACAAAAATAAACTACAACTTTAAATTTTAA 20720

cor TACTGTCTCAACAAAAATAAACTACAACTTTAAATTTTAA 20720

man TACTGTCTCAACAAAAATAAACTACAACTTTAAATTTTAA 20720

uni TACTGTCTCAACAAAAATAAACTACAACTTTAAATTTTAA 20720

tall TACTGTCTCAACAAAAATAAACTACAACTTTAAATTTTAA 20720

quil TACTGTCTCAACAAAAATAAACTACAACTTTAAATTTTAA 20686

meri TACTGTCTCAACAAAAATAAACTACAACTTTAAATTTTAA 20842

ref TACTGTCTCAACAAAAATAAACTACAACTTTAAATTTTAA 20968

Consensus tactgtctcaacaaaaataaactacaactttaaattttaa

kal CAGGAAATAACAATTTGATGGGTGAATGCATATCCCAAAT 20760

yor CAGGAAATAACAATTTGATGGGTGAATGCATATCCCAAAT 20744

jen CAGGAAATAACAATTTGATGGGTGAATGCATATCCCAAAT 20760

cor CAGGAAATAACAATTTGATGGGTGAATGCATATCCCAAAT 20760

man CAGGAAATAACAATTTGATGGGTGAATGCATATCCCAAAT 20760

uni CAGGAAATAACAATTTGATGGGTGAATGCATATCCCAAAT 20760

tall CAGGAAATAACAATTTGATGGGTGAATGCATATCCCAAAT 20760

quil CAGGAAATAACAATTTGATGGGTGAATGCATATCCCAAAT 20726

meri CAGGAAATAACAATTTGATGGGTGAATGCATATCCCAAAT 20882

ref CAGGAAATAACAATTTGATGGGTGAATGCATATCCCAAAT 21008

Consensus caggaaataacaatttgatgggtgaatgcatatcccaaat

kal GAGTAAAAACAATATGAAAGGTGAGGACTAGCTCATATAC 20800

yor GAGTAAAAACAATATGAAAGGTGAGGACTAGCTCATATAC 20784

jen GAGTAAAAACAATATGAAAGGTGAGGACTAGCTCATATAC 20800

cor GAGTAAAAACAATATGAAAGGTGAGGACTAGCTCATATAC 20800

man GAGTAAAAACAATATGAAAGGTGAGGACTAGCTCATATAC 20800

uni GAGTAAAAACAATATGAAAGGTGAGGACTAGCTCATATAC 20800

tall GAGTAAAAACAATATGAAAGGTGAGGACTAGCTCATATAC 20800

quil GAGTAAAAACAATATGAAAGGTGAGGACTAGCTCATATAC 20766

meri GAGTAAAAACAATATGAAAGGTGAGGACTAGCTCATATAC 20922

ref GAGTAAAAACAATATGAAAGGTGAGGACTAGCTCATATAC 21048

Consensus gagtaaaaacaatatgaaaggtgaggactagctcatatac

kal AATATTAGCTGATATATTCTGTGTCACAATCTCTTCTTGC 20840

yor AATATTAGCTGATATATTCTGTGTCACAATCTCTTCTTGC 20824

jen AATATTAGCTGATATATTCTGTGTCACAATCTCTTCTTGC 20840

cor AATATTAGCTGATATATTCTGTGTCACAATCTCTTCTTGC 20840

man AATATTAGCTGATATATTCTGTGTCACAATCTCTTCTTGC 20840

uni AATATTAGCTGATATATTCTGTGTCACAATCTCTTCTTGC 20840

tall AATATTAGCTGATATATTCTGTGTCACAATCTCTTCTTGC 20840

quil AATATTAGCTGATATATTCTGTGTCACAATCTCTTCTTGC 20806

meri AATATTAGCTGATATATTCTGTGTCACAATCTCTTCTTGC 20962

ref AATATTAGCTGATATATTCTGTGTCACAATCTCTTCTTGC 21088

Consensus aatattagctgatatattctgtgtcacaatctcttcttgc

kal ATGTATACTCCCCCAACCTTACCAGTGTCCAATTCAAATA 20880

yor ATGTATACTCCCCCAACCTTACCAGTGTCCAATTCAAATA 20864

jen ATGTATACTCCCCCAACCTTACCAGTGTCCAATTCAAATA 20880

cor ATGTATACTCCCCCAACCTTACCAGTGTCCAATTCAAATA 20880

man ATGTATACTCCCCCAACCTTACCAGTGTCCAATTCAAATA 20880

uni ATGTATACTCCCCCAACCTTACCAGTGTCCAATTCAAATA 20880

tall ATGTATACTCCCCCAACCTTACCAGTGTCCAATTCAAATA 20880

quil ATGTATACTCCCCCAACCTTACCAGTGTCCAATTCAAATA 20846

meri ATGTATACTCCCCCAACCTTACCAGTGTCCAATTCAAATA 21002

ref ATGTATACTCCCCCAACCTTACCAGTGTCCAATTCAAATA 21128

Consensus atgtatactcccccaaccttaccagtgtccaattcaaata

kal AATATGCGCCGATTTCACCTTGGACAAACTAGAATGGTAA 20920

yor AATATGCGCCGATTTCACCTTGGACAAACTAGAATGGTAA 20904

jen AATATGCGCCGATTTCACCTTGGACAAACTAGAATGGTAA 20920

cor AATATGCGCCGATTTCACCTTGGACAAACTAGAATGGTAA 20920

man AATATGCGCCGATTTCACCTTGGACAAACTAGAATGGTAA 20920

uni AATATGCGCCGATTTCACCTTGGACAAACTAGAATGGTAA 20920

tall AATATGCGCCGATTTCACCTTGGACAAACTAGAATGGTAA 20920

quil AATATGCGCCGATTTCACCTTGGACAAACTAGAATGGTAA 20886

meri AATATGCaCCGATTTCACCTTGGACAAACTAGAATGGTAA 21042

ref AATATGCGCCGATTTCACCTTGGACAAACTAGAATGGTAA 21168

Consensus aatatgc ccgatttcaccttggacaaactagaatggtaa

kal AAATCTAACGACGTCAACGGCCAATAATTTACTTTGAAAC 20960

yor AAATCTAACGACGTCAACGGCCAATAATTTACTTTGAAAC 20944

jen AAATCTAACGACGTCAACGGCCAATAATTTACTTTGAAAC 20960

cor AAATCTAACGACGTCAACGGCCAATAATTTACTTTGAAAC 20960

man AAATCTAACGACGTCAACGGCCAATAATTTACTTTGAAAC 20960

uni AAATCTAACGACGTCAACGGCCAATAATTTACTTTGAAAC 20960

tall AAATCTAACGACGTCAACGGCCAATAATTTACTTTGAAAC 20960

quil AAATCTAACGACGTCAACGGCCAATAATTTACTTTGAAAC 20926

meri AAATCTAACGACGTCAACGGCCAATAATTTACTTTGAAAC 21082

ref AAATCTAACGACGTCAACGGCCAATAATTTACTTTGAAAC 21208

Consensus aaatctaacgacgtcaacggccaataatttactttgaaac

kal ACCACCTTCACCATGTACAACAATTCCTGATACAAGCAAG 21000

yor ACCACCTTCACCATGTACAACAATTCCTGATACAAGCAAG 20984

jen ACCACCTTCACCATGTACAACAATTCCTGATACAAGCAAG 21000

cor ACCACCTTCACCATGTACAACAATTCCTGATACAAGCAAG 21000

man ACCACCTTCACCATGTACAACAATTCCTGATACAAGCAAG 21000

uni ACCACCTTCACCATGTACAACAATTCCTGATACAAGCAAG 21000

tall ACCACCTTCACCATGTACAACAATTCCTGATACAAGCAAG 21000

quil ACCACCTTCACCATGTACAACAATTCCTGATACAAGCAAG 20966

meri ACCACCTTCACCATGTACAACAATTCCTGATACAAGCAAG 21122

ref ACCACCTTCACCATGTACAACAATTCCTGATACAAGCAAG 21248

Consensus accaccttcaccatgtacaacaattcctgatacaagcaag

kal ACTATGATTTAAAGTTATAAGTTGCTACTCGTAATCACCT 21040

yor ACTAcGATTTAAAGTTATAAGTTGCTACTCGTAATCACCT 21024

jen ACTATGATTTAAAGTTATAAGTTGCTACTCGTAATCACCT 21040

cor ACTATGATTTAAAGTTATAAGTTGCTACTCGTAATCACCT 21040

man ACTATGATTTAAAGTTATAAGTTGCTACTCGTAATCACCT 21040

uni ACTATGATTTAAAGTTATAAGTTGCTACTCGTAATCACCT 21040

tall ACTATGATTTAAAGTTATAAGTTGCTACTCGTAATCACCT 21040

quil ACTATGATTTAAAGTTATAAGTTGCTACTCGTAATCACCT 21006

meri ACTAcGATTTAAAGTTATAAGTTGCTACTCGTAATCACCT 21162

ref ACTAcGATTTAAAGTTATAAGTTGCTACTCGTAATCACCT 21288

Consensus acta gatttaaagttataagttgctactcgtaatcacct

kal CCATCTCATGAGTTTCGGATACATTAAATCTTCATGGTGT 21080

yor CCATCTCATGAGTTTCGGATACATTAAATCTTCATGGTGT 21064

jen CCATCTCATGAGTTTCGGATACATTAAATCTTCATGGTGT 21080

cor CCATCTCATGAGTTTCGGATACATTAAATCTTCATGGTGT 21080

man CCATCTCATGAGTTTCGGATACATTAAATCTTCATGGTGT 21080

uni CCATCTCATGAGTTTCGGATACATTAAATCTTCATGGTGT 21080

tall CCATCTCATGAGTTTCGGATACATTAAATCTTCATGGTGT 21080

quil CCATCTCATGAGTTTCGGATACATTAAATCTTCATGGTGT 21046

meri CCATCTCATGAGTTTCGGATACATTAAATCTTCATGGTGT 21202

ref CCATCTCATGAGTTTCGGATACATTAAATCTTCATGGTGT 21328

Consensus ccatctcatgagtttcggatacattaaatcttcatggtgt

kal AGATCATGACACAGAAAACTCTCCTCAGTTTTGCTTTATC 21120

yor AGATCATGACACAGAAAACTCTCCTCAGTTTTGCTTTATC 21104

jen AGATCATGACACAGAAAACTCTCCTCAGTTTTGCTTTATC 21120

cor AGATCATGACACAGAAAACTCTCCTCAGTTTTGCTTTATC 21120

man AGATCATGACACAGAAAACTCTCCTCAGTTTTGCTTTATC 21120

uni AGATCATGACACAGAAAACTCTCCTCAGTTTTGCTTTATC 21120

tall AGATCATGACACAGAAAACTCTCCTCAGTTTTGCTTTATC 21120

quil AGATCATGACACAGAAAACTCTCCTCAGTTTTGCTTTATC 21086

meri AGATCATGACACAGAAAACTCTCCTCAGTTTTGCTTTATC 21242

ref AGATCATGACACAGAAAACTCTCCTCAGTTTTGCTTTATC 21368

Consensus agatcatgacacagaaaactctcctcagttttgctttatc

kal TCTTGAAGCATTTCTACGACATTCTTCATTTTCGGTCGTT 21160

yor TCTTGAAGCATTTCTACGACATTCTTCATTTTCGGTCGTT 21144

jen TCTTGAAGCATTTCTACGACATTCTTCATTTTCGGTCGTT 21160

cor TCTTGAAGCATTTCTACGACATTCTTCATTTTCGGTCGTT 21160

man TCTTGAAGCATTTCTACGACATTCTTCATTTTCGGTCGTT 21160

uni TCTTGAAGCATTTCTACGACATTCTTCATTTTCGGTCGTT 21160

tall TCTTGAAGCATTTCTACGACATTCTTCATTTTCGGTCGTT 21160

quil TCTTGAAGCATTTCTACGACATTCTTCATTTTCGGTCGTT 21126

meri TCTTGAAGCATTTCTACGACATTCTTCATTTTCGGTCGTT 21282

ref TCTTGAAGCATTTCTACGACATTCTTCATTTTCGGTCGTT 21408

Consensus tcttgaagcatttctacgacattcttcattttcggtcgtt

kal TGGCAGGTGTGTTGTCAGTGCATAGCAATGCGACCTTAAG 21200

yor TGGCAGGTGTGTTGTCAGTGCATAGCAATGCGACCTTAAG 21184

jen TGGCAGGTGTGTTGTCAGTGCATAGCAATGCGACCTTAAG 21200

cor TGGCAGGTGTGTTGTCAGTGCATAGCAATGCGACCTTAAG 21200

man TGGCAGGTGTGTTGTCAGTGCATAGCAATGCGACCTTAAG 21200

uni TGGCAGGTGTGTTGTCAGTGCATAGCAATGCGACCTTAAG 21200

tall TGGCAGGTGTGTTGTCAGTGCATAGCAATGCGACCTTAAG 21200

quil TGGCAGGTGTGTTGTCAGTGCATAGCAATGCGACCTTAAG 21166

meri TGGCAGGTGTGTTGTCAGTGCATAGCAATGCaACCTTAAG 21322

ref TGGCAGGTGTGTTGTCAGTGCATAGCAATGCGACCTTAAG 21448

Consensus tggcaggtgtgttgtcagtgcatagcaatgc accttaag

kal AGCAGCAAGCATGTCCTTCCTCCAACCAAACGAAACTGTG 21240

yor AGCAGCAAGCATGTCCTTCCTCCAACCAAACGAAACTGTG 21224

jen AGCAGCAAGCATGTCCTTCCTCCAACCAAACGAAACTGTG 21240

cor AGCAGCAAGCATGTCCTTCCTCCAACCAAACGAAACTGTG 21240

man AGCAGCAAGCATGTCCTTCCTCCAACCAAACGAAACTGTG 21240

uni AGCAGCAAGCATGTCCTTCCTCCAACCAAACGAAACTGTG 21240

tall AGCAGCAAGCATGTCCTTCCTCCAACCAAACGAAACTGTG 21240

quil AGCAGCAAGCATGTCCTTCCTCCAACCAAACGAAACTGTG 21206

meri AGCAGCAAGCATGTCCTTCCTCCAACCAAACGAAACTGTG 21362

ref AGCAGCAAGCATGTCCTTCCTCCAACCAAACGAAACTGTG 21488

Consensus agcagcaagcatgtccttcctccaaccaaacgaaactgtg

kal CTAAGCCTTGCATCAAGTATCTGCTCTGGAGTTTCATCTC 21280

yor CTAAGCCTTGCATCAAGTATCTGCTCTGGAGTTTCATCTC 21264

jen CTAAGCCTTGCATCAAGTATCTGCTCTGGAGTTTCATCTC 21280

cor CTAAGCCTTGCATCAAGTATCTGCTCTGGAGTTTCATCTC 21280

man CTAAGCCTTGCATCAAGTATCTGCTCTGGAGTTTCATCTC 21280

uni CTAAGCCTTGCATCAAGTATCTGCTCTGGAGTTTCATCTC 21280

tall CTAAGCCTTGCATCAAGTATCTGCTCTGGAGTTTCATCTC 21280

quil CTAAGCCTTGCATCAAGTATCTGCTCTGGAGTTTCATCTC 21246

meri CTAAGCCTTGCATCAAGTATCTGCTCTGGAGTTTCATCTC 21402

ref CTAAGCCTTGCATCAAGTATCTGCTCTGGAGTTTCATCTC 21528

Consensus ctaagccttgcatcaagtatctgctctggagtttcatctc

kal TTATTGGAGCACTATGAACCCACTTCACCAAATCTATGCC 21320

yor TTATTGGAGCACTATGAACCCACTTCACCAAATCTATGCC 21304

jen TTATTGGAGCACTATGAACCCACTTCACCAAATCTATGCC 21320

cor TTATTGGAGCACTATGAACCCACTTCACCAAATCTATGCC 21320

man TTATTGGAGCACTATGAACCCACTTCACCAAATCTATGCC 21320

uni TTATTGGAGCACTATGAACCCACTTCACCAAATCTATGCC 21320

tall TTATTGGAGCACTATGAACCCACTTCACCAAATCTATGCC 21320

quil TTATTGGAGCACTATGAACCCACTTCACCAAATCTATGCC 21286

meri TTATTGGAGCACTATGAACCCACTTCACCAAATCTATGCC 21442

ref TTATTGGAGCACTATGAACCCACTTCACCAAATCTATGCC 21568

Consensus ttattggagcactatgaacccacttcaccaaatctatgcc

kal TTCACCAAATTCTTCATCAACTGGTTGACGAGTTGTAAGG 21360

yor TTCACCAAATTCTTCATCAACTGGTTGACGAGTTGTAAGG 21344

jen TTCACCAAATTCTTCATCAACTGGTTGACGAGTTGTAAGG 21360

cor TTCACCAAATTCTTCATCAACTGGTTGACGAGTTGTAAGG 21360

man TTCACCAAATTCTTCATCAACTGGTTGACGAGTTGTAAGG 21360

uni TTCACCAAATTCTTCATCAACTGGTTGACGAGTTGTAAGG 21360

tall TTCACCAAATTCTTCATCAACTGGTTGACGAGTTGTAAGG 21360

quil TTCACCAAATTCTTCATCAACTGGTTGACGAGTTGTAAGG 21326

meri TTCACCAAATTCTTCATCAACTGGTTGACGAGTTGTAAGG 21482

ref TTCACCAAATTCTTCATCAACTGGTTGACGAGTTGTAAGG 21608

Consensus ttcaccaaattcttcatcaactggttgacgagttgtaagg

kal ATTTCCAAAAGAACAACACCATAGCTATAAACATTTCCTG 21400

yor ATTTCCAAAAGAACAACACCATAGCTATAAACATTTCCTG 21384

jen ATTTCCAAAAGAACAACACCATAGCTATAAACATTTCCTG 21400

cor ATTTCCAAAAGAACAACACCATAGCTATAAACATTTCCTG 21400

man ATTTCCAAAAGAACAACACCATAGCTATAAACATTTCCTG 21400

uni ATTTCCAAAAGAACAACACCATAGCTATAAACATTTCCTG 21400

tall ATTTCCAAAAGAACAACACCATAGCTATAAACATTTCCTG 21400

quil ATTTCCAAAAGAACAACACCATAGCTATAAACATTTCCTG 21366

meri ATTTCCAAAAGAACAACACCATAGCTATAAACATTTCCTG 21522

ref ATTTCCAAAAGAACAACACCATAGCTATAAACATTTCCTG 21648

Consensus atttccaaaagaacaacaccatagctataaacatttcctg

kal GCGCTGTAACTTGCATTGTATATGCATATTCTGCATCACA 21440

yor GCGCTGTAACTTGCATTGTATATGCATATTCTGCATCACA 21424

jen GCGCTGTAACTTGCATTGTATATGCATATTCTGCATCACA 21440

cor GCGCTGTAACTTGCATTGTATATGCATATTCTGCATCACA 21440

man GCGCTGTAACTTGCATTGTATATGCATATTCTGCATCACA 21440

uni GCGCTGTAACTTGCATTGTATATGCATATTCTGCATCACA 21440

tall GCGCTGTAACTTGCATTGTATATGCATATTCTGCATCACA 21440

quil GCGCTGTAACTTGCATTGTATATGCATATTCTGCATCACA 21406

meri GCGCTGTAACTTGCATTGTATATGCATATTCTGCATCACA 21562

ref GCGCTGTAACTTGCATTGTATATGCATATTCTGCATCACA 21688

Consensus gcgctgtaacttgcattgtatatgcatattctgcatcaca

kal AAAGAAGTGTGACATGAAGCATGCAAAAGATGTTAACAGT 21480

yor AAAGAAGTGTGACATGAAGCATGCAAAAGATGTTAACAGT 21464

jen AAAGAAGTGTGACATGAAGCATGCAAAAGATGTTAACAGT 21480

cor AAAGAAGTGTGACATGAAGCATGCAAAAGATGTTAACAGT 21480

man AAAGAAGTGTGACATGAAGCATGCAAAAGATGTTAACAGT 21480

uni AAAGAAGTGTGACATGAAGCATGCAAAAGATGTTAACAGT 21480

tall AAAGAAGTGTGACATGAAGCATGCAAAAGATGTTAACAGT 21480

quil AAAGAAGTGTGACATGAAGCATGCAAAAGATGTTAACAGT 21446

meri AAAGAAGTGTGACATGAAGCATGCAAAAGATGTTAACAGT 21602

ref AAAGAAGTGTGACATGAAGCATGCAAAAGATGTTAACAGT 21728

Consensus aaagaagtgtgacatgaagcatgcaaaagatgttaacagt

kal AAAGAGGAAGAATAGATGAGAAATTGCATGCCAGAAAATG 21520

yor AAAGAGGAAGAATAGATGAGAAATTGCATGCCAGAAAATG 21504

jen AAAGAGGAAGAATAGATGAGAAATTGCATGCCAGAAAATG 21520

cor AAAGAGGAAGAATAGATGAGAAATTGCATGCCAGAAAATG 21520

man AAAGAGGAAGAATAGATGAGAAATTGCATGCCAGAAAATG 21520

uni AAAGAGGAAGAATAGATGAGAAATTGCATGCCAGAAAATG 21520

tall AAAGAGGAAGAATAGATGAGAAATTGCATGCCAGAAAATG 21520

quil AAAGAGGAAGAATAGATGAGAAATTGCATGCCAGAAAATG 21486

meri AAAGAGGAAGAATAGATGAGAAATTGCATGCCAGAAAATG 21642

ref AAAGAGGAAGAATAGATGAGAAATTGCATGCCAGAAAATG 21768

Consensus aaagaggaagaatagatgagaaattgcatgccagaaaatg

kal GTAAAAGTATACCTGGTGGTATGTAACCAAAGGAACCAGC 21560

yor GTAAAAGTATACCTGGTGGTATGTAACCAAAGGAACCAGC 21544

jen GTAAAAGTATACCTGGTGGTATGTAACCAAAGGAACCAGC 21560

cor GTAAAAGTATACCTGGTGGTATGTAACCAAAGGAACCAGC 21560

man GTAAAAGTATACCTGGTGGTATGTAACCAAAGGAACCAGC 21560

uni GTAAAAGTATACCTGGTGGTATGTAACCAAAGGAACCAGC 21560

tall GTAAAAGTATACCTGGTGGTATGTAACCAAAGGAACCAGC 21560

quil GTAAAAGTATACCTGGTGGTATGTAACCAAAGGAACCAGC 21526

meri GTAAAAGTATACCTGGTGGTATGTAACCAAAGGAACCAGC 21682

ref GTAAAAGTATACCTGGTGGTATGTAACCAAAGGAACCAGC 21808

Consensus gtaaaagtatacctggtggtatgtaaccaaaggaaccagc

kal AACTGCACTAATGCTTCCAGTACCTTTGGTTGGATCAAGA 21600

yor AACTGCACTAATGCTTCCAGTACCTTTGGTTGGATCAAGA 21584

jen AACTGCACTAATGCTTCCAGTACCTTTGGTTGGATCAAGA 21600

cor AACTGCACTAATGCTTCCAGTACCTTTGGTTGGATCAAGA 21600

man AACTGCACTAATGCTTCCAGTACCTTTGGTTGGATCAAGA 21600

uni AACTGCACTAATGCTTCCAGTACCTTTGGTTGGATCAAGA 21600

tall AACTGCACTAATGCTTCCAGTACCTTTGGTTGGATCAAGA 21600

quil AACTGCACTAATGCTTCCAGTACCTTTGGTTGGATCAAGA 21566

meri AACTGCACTAATGCTTCCAGTACCTTTGGTTGGATCAAGA 21722

ref AACTGCACTAATGCTTCCAGTACCTTTGGTTGGATCAAGA 21848

Consensus aactgcactaatgcttccagtacctttggttggatcaaga

kal AGTTTTGAAATCTCGATCTCCCCAAGCAGCGGCTTAAAAT 21640

yor AGTTTTGAAATCTCGATCTCCCCAAGCAGCGGCTTAAAAT 21624

jen AGTTTTGAAATCTCGATCTCCCCAAGCAGCGGCTTAAAAT 21640

cor AGTTTTGAAATCTCGATCTCCCCAAGCAGCGGCTTAAAAT 21640

man AGTTTTGAAATCTCGATCTCCCCAAGCAGCGGCTTAAAAT 21640

uni AGTTTTGAAATCTCGATCTCCCCAAGCAGCGGCTTAAAAT 21640

tall AGTTTTGAAATCTCGATCTCCCCAAGCAGCGGCTTAAAAT 21640

quil AGTTTTGAAATCTCGATCTCCCCAAGCAGCGGCTTAAAAT 21606

meri AGTTTTGAAATCTCGATCTCCCCAAGCAGCGGCTTAAAAT 21762

ref AGTTTTGAAATCTCGATCTCCCCAAGCAGCGGCTTAAAAT 21888

Consensus agttttgaaatctcgatctccccaagcagcggcttaaaat

kal TTGCATCCAAAAGAACATTTCCAGAAGAAATGTCAAGATG 21680

yor TTGCATCCAAAAGAACATTgCCAGAAGAAATGTCAAGATG 21664

jen TTGCATCCAAAAGAACATTTCCAGAAGAAATGTCAAGATG 21680

cor TTGCATCCAAAAGAACATTTCCAGAAGAAATGTCAAGATG 21680

man TTGCATCCAAAAGAACATTTCCAGAAGAAATGTCAAGATG 21680

uni TTGCATCCAAAAGAACATTTCCAGAAGAAATGTCAAGATG 21680

tall TTGCATCCAAAAGAACATTTCCAGAAGAAATGTCAAGATG 21680

quil TTGCATCCAAAAGAACATTTCCAGAAGAAATGTCAAGATG 21646

meri TTGCATCCAAAAGAACATTTCCAGAAGAAATGTCAAGATG 21802

ref TTGCATCCAAAAGAACATTgCCAGAAGAAATGTCAAGATG 21928

Consensus ttgcatccaaaagaacatt ccagaagaaatgtcaagatg

kal GATAATCGCCACATGATGAAGGAAAGCCAAACCTTCAGCC 21720

yor GATAATCGCCACATGATGAAGGAAAGCCAAACCTTCAGCC 21704

jen GATAATCGCCACATGATGAAGGAAAGCCAAACCTTCAGCC 21720

cor GATAATCGCCACATGATGAAGGAAAGCCAAACCTTCAGCC 21720

man GATAATCGCCACATGATGAAGGAAAGCCAAACCTTCAGCC 21720

uni GATAATCGCCACATGATGAAGGAAAGCCAAACCTTCAGCC 21720

tall GATAATCGCCACATGATGAAGGAAAGCCAAACCTTCAGCC 21720

quil GATAATCGCCACATGATGAAGGAAAGCCAAACCTTCAGCC 21686

meri GATAATCGCCACATGATGAAGGAAAGCCAAACCTTCAGCC 21842

ref GATAATCGCCACATGATGAAGGAAAGCCAAACCTTCAGCC 21968

Consensus gataatcgccacatgatgaaggaaagccaaaccttcagcc

kal AATCCAATGGCAATGGATAGCCTCTCAGGCCAGTCAGGCT 21760

yor AATCCAATGGCAATGGATAGCCTCTCAGGCCAGTCAGGCT 21744

jen AATCCAATGGCAATGGATAGCCTCTCAGGCCAGTCAGGCT 21760

cor AATCCAATGGCAATGGATAGCCTCTCAGGCCAGTCAGGCT 21760

man AATCCAATGGCAATGGATAGCCTCTCAGGCCAGTCAGGCT 21760

uni AATCCAATGGCAATGGATAGCCTCTCAGGCCAGTCAGGCT 21760

tall AATCCAATGGCAATGGATAGCCTCTCAGGCCAGTCAGGCT 21760

quil AATCCAATGGCAATGGATAGCCTCTCAGGCCAGTCAGGCT 21726

meri AATCCAATGGCAATGGATAGCCTCTCAGGCCAGTCAGGCT 21882

ref AATCCAATGGCAATGGATAGCCTCTCAGGCCAGTCAGGCT 22008

Consensus aatccaatggcaatggatagcctctcaggccagtcaggct

kal GATAATCAGGTTGTCTGGTAGATTCATGAAGAAGCTGAGC 21800

yor GATAATCAGGTTGTCTGGTAGATTCATGAAGAAGCTGAGC 21784

jen GATAATCAGGTTGTCTGGTAGATTCATGAAGAAGCTGAGC 21800

cor GATAATCAGGTTGTCTGGTAGATTCATGAAGAAGCTGAGC 21800

man GATAATCAGGTTGTCTGGTAGATTCATGAAGAAGCTGAGC 21800

uni GATAATCAGGTTGTCTGGTAGATTCATGAAGAAGCTGAGC 21800

tall GATAATCAGGTTGTCTGGTAGATTCATGAAGAAGCTGAGC 21800

quil GATAATCAGGTTGTCTGGTAGATTCATGAAGAAGCTGAGC 21766

meri GATAATCAGGTTGTCTGGTAGATTCATGAAGAAGCTGAGC 21922

ref GATAATCAGGTTGTCTGGTAGATTCATGAAGAAGCTGAGC 22048

Consensus gataatcaggttgtctggtagattcatgaagaagctgagc

kal CAATGTTCCATCGGGAAAATAATGATGCAAGAGAAGAGCA 21840

yor CAATGTTCCATCGGGAAAATAATGATGCAAGAGAAGAGCA 21824

jen CAATGTTCCATCGGGAAAATAATGATGCAAGAGAAGAGCA 21840

cor CAATGTTCCATCGGGAAAATAATGATGCAAGAGAAGAGCA 21840

man CAATGTTCCATCGGGAAAATAATGATGCAAGAGAAGAGCA 21840

uni CAATGTTCCATCGGGAAAATAATGATGCAAGAGAAGAGCA 21840

tall CAATGTTCCATCGGGAAAATAATGATGCAAGAGAAGAGCA 21840

quil CAATGTTCCATCGGGAAAATAATGATGCAAGAGAAGAGCA 21806

meri CAATGTTCCATCGGGAAAATAATGATGCAAGAGAAGAGCA 21962

ref CAATGTTCCATCGGGAAAATAATGATGCAAGAGAAGAGCA 22088

Consensus caatgttccatcgggaaaataatgatgcaagagaagagca

kal ACATCTTCATAAATAACGTAGCCAATGGGTTGCACTATAT 21880

yor ACATCTTCATAAATAACGTAGCCAATGGGTTGCACTATAT 21864

jen ACATCTTCATAAATAACGTAGCCAATGGGTTGCACTATAT 21880

cor ACATCTTCATAAATAACGTAGCCAATGGGTTGCACTATAT 21880

man ACATCTTCATAAATAACGTAGCCAATGGGTTGCACTATAT 21880

uni ACATCTTCATAAATAACGTAGCCAATGGGTTGCACTATAT 21880

tall ACATCTTCATAAATAACGTAGCCAATGGGTTGCACTATAT 21880

quil ACATCTTCATAAATAACGTAGCCAATGGGTTGCACTATAT 21846

meri ACATCTTCATAAATAACGTAGCCAATGGGTTGCACTATAT 22002

ref ACATCTTCATAAATAACGTAGCCAATGGGTTGCACTATAT 22128

Consensus acatcttcataaataacgtagccaatgggttgcactatat

kal TAGCATGAGAAACTTTTCCAAGCCTTTCAAGCTCTCTAAT 21920

yor TAGCATGAGAAACTTTTCCAAGCCTTTCAAGCTCTCTAAT 21904

jen TAGCATGAGAAACTTTTCCAAGCCTTTCAAGCTCTCTAAT 21920

cor TAGCATGAGAAACTTTTCCAAGCCTTTCAAGCTCTCTAAT 21920

man TAGCATGAGAAACTTTTCCAAGCCTTTCAAGCTCTCTAAT 21920

uni TAGCATGAGAAACTTTTCCAAGCCTTTCAAGCTCTCTAAT 21920

tall TAGCATGAGAAACTTTTCCAAGCCTTTCAAGCTCTCTAAT 21920

quil TAGCATGAGAAACTTTTCCAAGCCTTTCAAGCTCTCTAAT 21886

meri TAGCATGAGAAACTTTTCCAAGCCTTTCAAGCTCTCTAAT 22042

ref TAGCATGAGAAACTTTTCCAAGCCTTTCAAGCTCTCTAAT 22168

Consensus tagcatgagaaacttttccaagcctttcaagctctctaat

kal CATCTTGTTCTGGTGCTGGATTATTGTCTTGTCTATGGAC 21960

yor CATCTTGTTCTGGTGCTGGATTATTGTCTTGTCTATGGAC 21944

jen CATCTTGTTCTGGTGCTGGATTATTGTCTTGTCTATGGAC 21960

cor CATCTTGTTCTGGTGCTGGATTATTGTCTTGTCTATGGAC 21960

man CATCTTGTTCTGGTGCTGGATTATTGTCTTGTCTATGGAC 21960

uni CATCTTGTTCTGGTGCTGGATTATTGTCTTGTCTATGGAC 21960

tall CATCTTGTTCTGGTGCTGGATTATTGTCTTGTCTATGGAC 21960

quil CATCTTGTTCTGGTGCTGGATTATTGTCTTGTCTATGGAC 21926

meri CATCTTGTTCTGGTGCTGGATTATTGTCTTGTCTATGGAC 22082

ref CATCTTGTTCTGGTGCTGGATTATTGTCTTGTCTATGGAC 22208

Consensus catcttgttctggtgctggattattgtcttgtctatggac

kal TTCAATCTCCTTACAGATAAGACCATCCCAGAAGGCATGA 22000

yor TTCAATCTCCTTACAGATAAGACCATCCCAGAAGGCATGA 21984

jen TTCAATCTCCTTACAGATAAGACCATCCCAGAAGGCATGA 22000

cor TTCAATCTCCTTACAGATAAGACCATCCCtGAAGGCATGA 22000

man TTCAATCTCCTTACAGATAAGACCATCCCAGAAGGCATGA 22000

uni TTCAATCTCCTTACAGATAAGACCATCCCAGAAGGCATGA 22000

tall TTCAATCTCCTTACAGATAAGACCATCCCAGAAGGCATGA 22000

quil TTCAATCTCCTTACAGATAAGACCATCCCAGAAGGCATGA 21966

meri TTCAATCTCCTTACAGATAAGACCATCCCAGAAGGCATGA 22122

ref TTCAATCTCCTTACAGATAAGACCATCCCAGAAGGCATGA 22248

Consensus ttcaatctccttacagataagaccatccc gaaggcatga

kal CTGCTTTATAAACTGTGCTGAAAGTTCCGCTGCTGACTTT 22040

yor CTGCTTTATAAACTGTGCTGAAAGTTCCGCTGCTGACTTT 22024

jen CTGCTTTATAAACTGTGCTGAAAGTTCCGCTGCTGACTTT 22040

cor CTGCTTTATAAACTGTGCTGAAAGTTCCGCTGCTGACTTT 22040

man CTGCTTTATAAACTGTGCTGAAAGTTCCGCTGCTGACTTT 22040

uni CTGCTTTATAAACTGTGCTGAAAGTTCCGCTGCTGACTTT 22040

tall CTGCTTTATAAACTGTGCTGAAAGTTCCGCTGCTGACTTT 22040

quil CTGCTTTATAAACTGTGCTGAAAGTTCCGCTGCTGACTTT 22006

meri CTGCTTTATAAACTGTGCTGAAAGTTCCGCTGCTGACTTT 22162

ref CTGCTTTATAAACTGTGCTGAAAGTTCCGCTGCTGACTTT 22288

Consensus ctgctttataaactgtgctgaaagttccgctgctgacttt

kal ATTAGAGTCTTTAAGAGTTGCTTTAACAACGGCATCAAGG 22080

yor ATTAGAGTCTTTAAGAGTTGCTTTAACAACGGCATCAAGG 22064

jen ATTAGAGTCTTTAAGAGTTGCTTTAACAACGGCATCAAGG 22080

cor ATTAGAGTCTTTAAGAGTTGCTTTAACAACGGCATCAAGG 22080

man ATTAGAGTCTTTAAGAGTTGCTTTAACAACGGCATCAAGG 22080

uni ATTAGAGTCTTTAAGAGTTGCTTTAACAACGGCATCAAGG 22080

tall ATTAGAGTCTTTAAGAGTTGCTTTAACAACGGCATCAAGG 22080

quil ATTAGAGTCTTTAAGAGTTGCTTTAACAACGGCATCAAGG 22046

meri ATTAGAGTCTTTAAGAGTTGCTTTAACAACGGCATCAAGG 22202

ref ATTAGAGTCTTTAAGAGTTGCTTTAACAACGGCATCAAGG 22328

Consensus attagagtctttaagagttgctttaacaacggcatcaagg

kal TCTATCGCTTGTCTTAGATTATCAACAAAAACACCCCCTG 22120

yor TCTATCGCTTGTCTTAGATTATCAACAAAAACACCCCCTG 22104

jen TCTATCGCTTGTCTTAGATTATCAACAAAAACACCCCCTG 22120

cor TCTATCGCTTGTCTTAGATTATCAACAAAAACACCCCCTG 22120

man TCTATCGCTTGTCTTAGATTATCAACAAAAACACCCCCTG 22120

uni TCTATCGCTTGTCTTAGATTATCAACAAAAACACCCCCTG 22120

tall TCTATCGCTTGTCTTAGATTATCAACAAAAACACCCCCTG 22120

quil TCTATCGCTTGTCTTAGATTATCAACAAAAACACCCCCTG 22086

meri TCTATCGCTTGTCTTAGATTATCAACAAAAACACCCCCTG 22242

ref TCTATCGCTTGTCTTAGATTATCAACAAAAACACCCCCTG 22368

Consensus tctatcgcttgtcttagattatcaacaaaaacaccccctg

kal CTATTACAGTTGGTTTGTCATTGGCTCCGTCATCAACAAT 22160

yor CTATTACAGTTGGTTTGTCATTGGCTCCGTCATCAACAAT 22144

jen CTATTACAGTTGGTTTGTCATTGGCTCCGTCATCAACAAT 22160

cor CTATTACAGTTGGTTTGTCATTGGCTCCGTCATCAACAAT 22160

man CTATTACAGTTGGTTTGTCATTGGCTCCGTCATCAACAAT 22160

uni CTATTACAGTTGGTTTGTCATTGGCTCCGTCATCAACAAT 22160

tall CTATTACAGTTGGTTTGTCATTGGCTCCGTCATCAACAAT 22160

quil CTATTACAGTTGGTTTGTCATTGGCTCCGTCATCAACAAT 22126

meri CTATTACAGTTGGTTTGTCATTGGCTCCGTCATCAACAAT 22282

ref CTATTACAGTTGGTTTGTCATTGGCTCCGTCATCAACAAT 22408

Consensus ctattacagttggtttgtcattggctccgtcatcaacaat

kal TCTGTCGGCCTCTTTGGCTACTTTTTCTTGCCTTTCTCTG 22200

yor TCTGTCGGCCTCTTTGGCTACTTTTTCTTGCCTTTCTCTG 22184

jen TCTGTCGGCCTCTTTGGCTACTTTTTCTTGCCTTTCTCTG 22200

cor TCTGTCGGCCTCTTTGGCTACTTTTTCTTGCCTTTCTCTG 22200

man TCTGTCGGCCTCTTTGGCTACTTTTTCTTGCCTTTCTCTG 22200

uni TCTGTCGGCCTCTTTGGCTACTTTTTCTTGCCTTTCTCTG 22200

tall TCTGTCGGCCTCTTTGGCTACTTTTTCTTGCCTTTCTCTG 22200

quil TCTGTCGGCCTCTTTGGCTACTTTTTCTTGCCTTTCTCTG 22166

meri TCTGTCGGCCTCTTTGGCTACTTTTTCTTGCCTTTCTCTG 22322

ref TCTGTCGGCCTCTTTGGCTACTTTTTCTTGCCTTTCTCTG 22448

Consensus tctgtcggcctctttggctactttttcttgcctttctctg

kal ATCATAAACAGCAAGACGACTACAGTCACTGACATAAAAA 22240

yor ATCATAAACAGCAAGACGACTACAGTCACTGACATAAAAA 22224

jen ATCATAAACAGCAAGACGACTACAGTCACTGACATAAAAA 22240

cor ATCATAAACAGCAAGACGACTACAGTCACTGACATAAAAA 22240

man ATCATAAACAGCAAGACGACTACAGTCACTGACATAAAAA 22240

uni ATCATAAACAGCAAGACGACTACAGTCACTGACATAAAAA 22240

tall ATCATAAACAGCAAGACGACTACAGTCACTGACATAAAAA 22240

quil ATCATAAACAGCAAGACGACTACAGTCACTGACATAAAAA 22206

meri ATCATAAACAGCAAGACGACTACAGTCACTGACATAAAAA 22362

ref ATCATAAACAGCAAGACGACTACAGTCACTGACATAAAAA 22488

Consensus atcataaacagcaagacgactacagtcactgacataaaaa

kal CTGCCAAACCAGAACCAATTACGGCCAGTATGATTCTATA 22280

yor CTGCCAAACCAGAACCAATTACGGCCAGTATGATTCTATA 22264

jen CTGCCAAACCAGAACCAATTACGGCCAGTATGATTCTATA 22280

cor CTGCCAAACCAGAACCAATTACGGCCAGTATGATTCTATA 22280

man CTGCCAAACCAGAACCAATTACGGCCAGTATGATTCTATA 22280

uni CTGCCAAACCAGAACCAATTACGGCCAGTATGATTCTATA 22280

tall CTGCCAAACCAGAACCAATTACGGCCAGTATGATTCTATA 22280

quil CTGCCAAACCAGAACCAATTACGGCCAGTATGATTCTATA 22246

meri CTGCCAAACCAGAACCAATTACGGCCAGTATGATTCTATA 22402

ref CTGCCAAACCAGAACCAATTACGGCCAGTATGATTCTATA 22528

Consensus ctgccaaaccagaaccaattacggccagtatgattctata

kal TGAAACCCTGTGATGGTAATTCTTGTGCTCATCATAAATA 22320

yor TGAAACCCTGTGATGGTAATTCTTGTGCTCATCATAAATA 22304

jen TGAAACCCTGTGATGGTAATTCTTGTGCTCATCATAAATA 22320

cor TGAAACCCTGTGATGGTAATTCTTGTGCTCATCATAAATA 22320

man TGAAACCCTGTGATGGTAATTCTTGTGCTCATCATAAATA 22320

uni TGAAACCCTGTGATGGTAATTCTTGTGCTCATCATAAATA 22320

tall TGAAACCCTGTGATGGTAATTCTTGTGCTCATCATAAATA 22320

quil TGAAACCCTGTGATGGTAATTCTTGTGCTCATCATAAATA 22286

meri TGAAACCCTGTGATGGTAATTCTTGTGCTCATCATAAATA 22442

ref TGAAACCCTGTGATGGTAATTCTTGTGCTCATCATAAATA 22568

Consensus tgaaaccctgtgatggtaattcttgtgctcatcataaata

kal TCACCACATGAAGAGTTTAATGGCTCTCCACAAAGCCCCT 22360

yor TCACCACATGAAGAGTTTAATGGCTCTCCACAAAGCCCCT 22344

jen TCACCACATGAAGAGTTTAATGGCTCTCCACAAAGCCCCT 22360

cor TCACCACATGAAGAGTTTAATGGCTCTCCACAAAGCCCCT 22360

man TCACCACATGAAGAGTTTAATGGCTCTCCACAAAGCCCCT 22360

uni TCACCACATGAAGAGTTTAATGGCTCTCCACAAAGCCCCT 22360

tall TCACCACATGAAGAGTTTAATGGCTCTCCACAAAGCCCCT 22360

quil TCACCACATGAAGAGTTTAATGGCTCTCCACAAAGCCCCT 22326

meri TCACCACATGAAGAGTTTAATGGCTCTCCACAAAGCCCCT 22482

ref TCACCACATGAAGAGTTTAATGGCTCTCCACAAAGCCCCT 22608

Consensus tcaccacatgaagagtttaatggctctccacaaagcccct

kal TGTTGCCCAAAAAACTGGAACTAGGACACTTCTGGAATGG 22400

yor TGTTGCCCAAAAAACTGGAACTAGGACACTTCTGGAATGG 22384

jen TGTTGCCCAAAAAACTGGAACTAGGACACTTCTGGAATGG 22400

cor TGTTGCCCAAAAAACTGGAACTAGGACACTTCTGGAATGG 22400

man TGTTGCCCAAAAAACTGGAACTAGGACACTTCTGGAATGG 22400

uni TGTTGCCCAAAAAACTGGAACTAGGACACTTCTGGAATGG 22400

tall TGTTGCCCAAAAAACTGGAACTAGGACACTTCTGGAATGG 22400

quil TGTTGCCCAAAAAACTGGAACTAGGACACTTCTGGAATGG 22366

meri TGTTGCCCAAAAAACTGGAACTAGGACACTTCTGGAATGG 22522

ref TGTTGCCCAAAAAACTGGAACTAGGACACTTCTGGAATGG 22648

Consensus tgttgcccaaaaaactggaactaggacacttctggaatgg

kal TACAAATGTTGGTACAGGACCTCCGAAAAGATTATTTGAG 22440

yor TACAAATGTTGGTACAGGACCTCCGAAAAGATTATTTGAG 22424

jen TACAAATGTTGGTACAGGACCTCCGAAAAGATTATTTGAG 22440

cor TACAAATGTTGGTACAGGACCTCCGAAAAGATTATTTGAG 22440

man TACAAATGTTGGTACAGGACCTCCGAAAAGATTATTTGAG 22440

uni TACAAATGTTGGTACAGGACCTCCGAAAAGATTATTTGAG 22440

tall TACAAATGTTGGTACAGGACCTCCGAAAAGATTATTTGAG 22440

quil TACAAATGTTGGTACAGGACCTCCGAAAAGATTATTTGAG 22406

meri TACAAATGTTGGTACAGGACCTCCGAAAAGATTATTTGAG 22562

ref TACAAATGTTGGTACAGGACCTCCGAAAAGATTATTTGAG 22688

Consensus tacaaatgttggtacaggacctccgaaaagattatttgag

kal AAGTTAACCTCAATCAAGCTCAACATTCCCTTAAGCTCAT 22480

yor AAGTTAACCTCAATCAAGCTCAACATTCCCTTAAGCTCAT 22464

jen AAGTTAACCTCAATCAAGCTCAACATTCCCTTAAGCTCAT 22480

cor AAGTTAACCTCAATCAAGCTCAACATTCCCTTAAGCTCAT 22480

man AAGTTAACCTCAATCAAGCTCAACATTCCCTTAAGCTCAT 22480

uni AAGTTAACCTCAATCAAGCTCAACATTCCCTTAAGCTCAT 22480

tall AAGTTAACCTCAATCAAGCTCAACATTCCCTTAAGCTCAT 22480

quil AAGTTAACCTCAATCAAGCTCAACATTCCCTTAAGCTCAT 22446

meri AAGTTAACCTCAATCAAGCTCAACATTCCCTTAAGCTCAT 22602

ref AAGTTAACCTCAATCAAGCTCAACATTCCCTTAAGCTCAT 22728

Consensus aagttaacctcaatcaagctcaacattcccttaagctcat

kal CTGGGATATTACCTGAGAGCCGATTGTTTGAGACATCGAG 22520

yor CTGGGATATTACCTGAGAGCCGATTGTTTGAGACATCGAG 22504

jen CTGGGATATTACCTGAGAGCCGATTGTTTGAGACATCGAG 22520

cor CTGGGATATTACCTGAGAGCCGATTGTTTGAGACATCGAG 22520

man CTGGGATATTACCTGAGAGCCGATTGTTTGAGACATCGAG 22520

uni CTGGGATATTACCTGAGAGCCGATTGTTTGAGACATCGAG 22520

tall CTGGGATATTACCTGAGAGCCGATTGTTTGAGACATCGAG 22520

quil CTGGGATATTACCTGAGAGCCGATTGTTTGAGACATCGAG 22486

meri CTGGGATATTACCTGAGAGCCGATTGTTTGAGACATCGAG 22642

ref CTGGGATATTACCTGAGAGCCGATTGTTTGAGACATCGAG 22768

Consensus ctgggatattacctgagagccgattgtttgagacatcgag

kal AGAAACAAGTTTGTCCAGTCTTCCTAATTCGGGAGGCAAT 22560

yor AGAAACAAGTTTGTCCAGTCTTCCTAATTCGGGAGGCAAT 22544

jen AGAAACAAGTTTGTCCAGTCTTCCTAATTCGGGAGGCAAT 22560

cor AGAAACAAGTTTGTCCAGTCTTCCTAATTCGGGAGGCAAT 22560

man AGAAACAAGTTTGTCCAGTCTTCCTAATTCGGGAGGCAAT 22560

uni AGAAACAAGTTTGTCCAGTCTTCCTAATTCGGGAGGCAAT 22560

tall AGAAACAAGTTTGTCCAGTCTTCCTAATTCGGGAGGCAAT 22560

quil AGAAACAAGTTTGTCCAGTCTTCCTAATTCGGGAGGCAAT 22526

meri AGAAACAAGTTTGTCCAGTCTTCCTAATTCGGGAGGCAAT 22682

ref AGAAACAAGTTTGTCCAGTCTTCCTAATTCGGGAGGCAAT 22808

Consensus agaaacaagtttgtccagtcttcctaattcgggaggcaat

kal GGTCCATGAAGATGATTGAAGCTCAAATTTAAAGCTATCT 22600

yor GGTCCATGAAGATGATTGAAGCTCAAATTTAAAGCTATCT 22584

jen GGTCCATGAAGATGATTGAAGCTCAAATTTAAAGCTATCT 22600

cor GGTCCATGAAGATGATTGAAGCTCAAATTTAAAGCTATCT 22600

man GGTCCATGAAGATGATTGAAGCTCAAATTTAAAGCTATCT 22600

uni GGTCCATGAAGATGATTGAAGCTCAAATTTAAAGCTATCT 22600

tall GGTCCATGAAGATGATTGAAGCTCAAATTTAAAGCTATCT 22600

quil GGTCCATGAAGATGATTGAAGCTCAAATTTAAAGCTATCT 22566

meri GGTCCATGAAGATGATTGAAGCTCAAATTTAAAGCTATCT 22722

ref GGTCCATGAAGATGATTGAAGCTCAAATTTAAAGCTATCT 22848

Consensus ggtccatgaagatgattgaagctcaaatttaaagctatct

kal GTAAGTTCCGGATATGGCCAATCTCAGGAGGGATAGTTCC 22640

yor GTAAGTTCCGGATATGGCCAATCTCAGGAGGGATAGTTCC 22624

jen GTAAGTTCCGGATATGGCCAATCTCAGGAGGGATAGTTCC 22640

cor GTAAGTTCCGGATATGGCCAATCTCAGGAGGGATAGTTCC 22640

man GTAAGTTCCGGATATGGCCAATCTCAGGAGGGATAGTTCC 22640

uni GTAAGTTCCGGATATGGCCAATCTCAGGAGGGATAGTTCC 22640

tall GTAAGTTCCGGATATGGCCAATCTCAGGAGGGATAGTTCC 22640

quil GTAAGTTCCGGATATGGCCAATCTCAGGAGGGATAGTTCC 22606

meri GTAAGTTCCGGATATGGCCAATCTCAGGAGGGATAGTTCC 22762

ref GTAAGTTCCGGATATGGCCAATCTCAGGAGGGATAGTTCC 22888

Consensus gtaagttccggatatggccaatctcaggagggatagttcc

kal AGACAAATAATTACTGCCCAATTGTAACTCAAGAAGTTTT 22680

yor AGACAAATAATTACTGCCCAATTGTAACTCAAGAAGTTTT 22664

jen AGACAAATAATTACTGCCCAATTGTAACTCAAGAAGTTTT 22680

cor AGACAAATAATTACTGCCCAATTGTAACTCAAGAAGTTTT 22680

man AGACAAATAATTACTGCCCAATTGTAACTCAAGAAGTTTT 22680

uni AGACAAATAATTACTGCCCAATTGTAACTCAAGAAGTTTT 22680

tall AGACAAATAATTACTGCCCAATTGTAACTCAAGAAGTTTT 22680

quil AGACAAATAATTACTGCCCAATTGTAACTCAAGAAGTTTT 22646

meri AGACAAATAATTACTGCCCAATTGTAACTCAAGAAGTTTT 22802

ref AGACAAATAATTACTGCCCAATTGTAACTCAAGAAGTTTT 22928

Consensus agacaaataattactgcccaattgtaactcaagaagtttt

kal GCACAACTTCCAATTTCATGAGGGATCTCTCCTCTCATGG 22720

yor GCACAACTTCCAATTTCATGAGGGATCTCTCCTCTCATGG 22704

jen GCACAACTTCCAATTTCATGAGGGATCTCTCCTCTCATGG 22720

cor GCACAACTTCCAATTTCATGAGGGATCTCTCCTCTCATGG 22720

man GCACAACTTCCAATTTCATGAGGGATCTCTCCTCTCATGG 22720

uni GCACAACTTCCAATTTCATGAGGGATCTCTCCTCTCATGG 22720

tall GCACAACTTCCAATTTCATGAGGGATCTCTCCTCTCATGG 22720

quil GCACAACTTCCAATTTCATGAGGGATCTCTCCTCTCATGG 22686

meri GCACAACTTCCAATTTCATGAGGGATCTCTCCTCTCATGG 22842

ref GCACAACTTCCAATTTCATGAGGGATCTCTCCTCTCATGG 22968

Consensus gcacaacttccaatttcatgagggatctctcctctcatgg

kal AATTTTGATCCAATAGCAAGTACTGCAACCGAGTGACACT 22760

yor AATTTTGATCCAATAGCAAGTACTGCAACCGAGTGACACT 22744

jen AATTTTGATCCAATAGCAAGTACTGCAACCGAGTGACACT 22760

cor AATTTTGATCCAATAGCAAGTACTGCAACCGAGTGACACT 22760

man AATTTTGATCCAATAGCAAGTACTGCAACCGAGTGACACT 22760

uni AATTTTGATCCAATAGCAAGTACTGCAACCGAGTGACACT 22760

tall AATTTTGATCCAATAGCAAGTACTGCAACCGAGTGACACT 22760

quil AATTTTGATCCAATAGCAAGTACTGCAACCGAGTGACACT 22726

meri AATTTTGATCCAATAGCAAGTACTGCAACCGAGTGACACT 22882

ref AATTTTGATCCAATAGCAAGTACTGCAACCGAGTGACACT 23008

Consensus aattttgatccaatagcaagtactgcaaccgagtgacact

kal GCAAATTTCGTTTGGTATTGTACCATTGAATCTGTTGTTG 22800

yor GCAAATTTCGTTTGGTATTGTACCATTGAATCTGTTGTTG 22784

jen GCAAATTTCGTTTGGTATTGTACCATTGAATCTGTTGTTG 22800

cor GCAAATTTCGTTTGGTATTGTACCATTGAATCTGTTGTTG 22800

man GCAAATTTCGTTTGGTATTGTACCATTGAATCTGTTGTTG 22800

uni GCAAATTTCGTTTGGTATTGTACCATTGAATCTGTTGTTG 22800

tall GCAAATTTCGTTTGGTATTGTACCATTGAATCTGTTGTTG 22800

quil GCAAATTTCGTTTGGTATTGTACCATTGAATCTGTTGTTG 22766

meri GCAAATTTCGTTTGGTATTGTACCATTGAATCTGTTGTTG 22922

ref GCAAATTTCGTTTGGTATTGTACCATTGAATCTGTTGTTG 23048

Consensus gcaaatttcgtttggtattgtaccattgaatctgttgttg

kal CTAATATCAAGCTTGTTAAGATTTTTGCAGCCCAGAATCG 22840

yor CTAATATCAAGCTTGTTAAGATTTTTGCAGCCCAGAATCG 22824

jen CTAATATCAAGCTTGTTAAGATTTTTGCAGCCCAGAATCG 22840

cor CTAATATCAAGCTTGTTAAGATTTTTGCAGCCCAGAATCG 22840

man CTAATATCAAGCTTGTTAAGATTTTTGCAGCCCAGAATCG 22840

uni CTAATATCAAGCTTGTTAAGATTTTTGCAGCCCAGAATCG 22840

tall CTAATATCAAGCTTGTTAAGATTTTTGCAGCCCAGAATCG 22840

quil CTAATATCAAGCTTGTTAAGATTTTTGCAGCCCAGAATCG 22806

meri CTAATATCAAGCTTGTTAAGATTTTTGCAGCCCAGAATCG 22962

ref CTAATATCAAGCTTGTTAAGATTTTTGCAGCCCAGAATCG 23088

Consensus ctaatatcaagcttgttaagatttttgcagcccagaatcg

kal AGTTTGGAATATCACCGAAGAGACTATTTCCAGAAAGGAT 22880

yor AGTTTGGAATATCACCGAAGAGACTATTTCCAGAAAGGAT 22864

jen AGTTTGGAATATCACCGAAGAGACTATTTCCAGAAAGGAT 22880

cor AGTTTGGAATATCACCGAAGAGACTATTTCCAGAAAGGAT 22880

man AGTTTGGAATATCACCGAAGAGACTATTTCCAGAAAGGAT 22880

uni AGTTTGGAATATCACCGAAGAGACTATTTCCAGAAAGGAT 22880

tall AGTTTGGAATATCACCGAAGAGACTATTTCCAGAAAGGAT 22880

quil AGTTTGGAATATCACCGAAGAGACTATTTCCAGAAAGGAT 22846

meri AGTTTGGAATATCACCGAAGAGACTATTTCCAGAAAGGAT 23002

ref AGTTTGGAATATCACCGAAGAGACTATTTCCAGAAAGGAT 23128

Consensus agtttggaatatcaccgaagagactatttccagaaaggat

kal CAACTCCTGTAGGTTCATAAGCTTTCCAAATTCTGGTGGA 22920

yor CAACTCCTGTAGGTTCATAAGCTTTCCAAATTCTGGTGGA 22904

jen CAACTCCTGTAGGTTCATAAGCTTTCCAAATTCTGGTGGA 22920

cor CAACTCCTGTAGGTTCATAAGCTTTCCAAATTCTGGTGGA 22920

man CAACTCCTGTAGGTTCATAAGCTTTCCAAATTCTGGTGGA 22920

uni CAACTCCTGTAGGTTCATAAGCTTTCCAAATTCTGGTGGA 22920

tall CAACTCCTGTAGGTTCATAAGCTTTCCAAATTCTGGTGGA 22920

quil CAACTCCTGTAGGTTCATAAGCTTTCCAAATTCTGGTGGA 22886

meri CAACTCCTGTAGGTTCATAAGCTTTCCAAATTCTGGTGGA 23042

ref CAACTCCTGTAGGTTCATAAGCTTTCCAAATTCTGGTGGA 23168

Consensus caactcctgtaggttcataagctttccaaattctggtgga

kal ATTATACCAGTAAATCCATTTTCAGCTAAATTCAGGAGAG 22960

yor ATTATACCAGTAAATCCATTTTCAGCTAAATTCAGGAGAG 22944

jen ATTATACCAGTAAATCCATTTTCAGCTAAATTCAGGAGAG 22960

cor ATTATACCAGTAAATCCATTTTCAGCTAAATTCAGGAGAG 22960

man ATTATACCAGTAAATCCATTTTCAGCTAAATTCAGGAGAG 22960

uni ATTATACCAGTAAATCCATTTTCAGCTAAATTCAGGAGAG 22960

tall ATTATACCAGTAAATCCATTTTCAGCTAAATTCAGGAGAG 22960

quil ATTATACCAGTAAATCCATTTTCAGCTAAATTCAGGAGAG 22926

meri ATTATACCAGTAAATCCATTTTCAGCTAAATTCAGGAGAG 23082

ref ATTATACCAGTAAATCCATTTTCAGCTAAATTCAGGAGAG 23208

Consensus attataccagtaaatccattttcagctaaattcaggagag

kal TCAGATTAGAACACCGAGAAAATTCTGACACTATCTCACC 23000

yor TCAGATTAGAACACCGAGAAAATTCTGACACTATCTCACC 22984

jen TCAGATTAGAACACCGAGAAAATTCTGACACTATCTCACC 23000

cor TCAGATTAGAACACCGAGAAAATTCTGACACTATCTCACC 23000

man TCAGATTAGAACACCGAGAAAATTCTGACACTATCTCACC 23000

uni TCAGATTAGAACACCGAGAAAATTCTGACACTATCTCACC 23000

tall TCAGATTAGAACACCGAGAAAATTCTGACACTATCTCACC 23000

quil TCAGATTAGAACACCGAGAAAATTCTGACACTATCTCACC 22966

meri TCAGATTAGAACACCGAGAAAATTCTGACACTATCTCACC 23122

ref TCAGATTAGAACACCGAGAAAATTCTGACACTATCTCACC 23248

Consensus tcagattagaacaccgagaaaattctgacactatctcacc

kal AGAAAGGTTGTTATTGTCGGCTTCAAAGTAGGTTAGGCCA 23040

yor AGAAAGGTTGTTATTGTCGGCTTCAAAGTAGGTTAGGCCA 23024

jen AGAAAGGTTGTTATTGTCGGCTTCAAAGTAGGTTAGGCCA 23040

cor AGAAAGGTTGTTATTGTCGGCTTCAAAGTAGGTTAGGCCA 23040

man AGAAAGGTTGTTATTGTCGGCTTCAAAGTAGGTTAGGCCA 23040

uni AGAAAGGTTGTTATTGTCGGCTTCAAAGTAGGTTAGGCCA 23040

tall AGAAAGGTTGTTATTGTCGGCTTCAAAGTAGGTTAGGCCA 23040

quil AGAAAGGTTGTTATTGTCGGCTTCAAAGTAGGTTAGGCCA 23006

meri AGAAAGGTTGTTATTGTCGGCTTCAAAGTAGGTTAGGCCA 23162

ref AGAAAGGTTGTTATTGTCGGCTTCAAAGTAGGTTAGGCCA 23288

Consensus agaaaggttgttattgtcggcttcaaagtaggttaggcca

kal CTAAGATTTCCAATCGTCTTAGGAATACTACCCACTAGAT 23080

yor CTAAGATTTCCAATCGTCTTAGGAATACTACCCACTAGAT 23064

jen CTAAGATTTCCAATCGTCTTAGGAATACTACCCACTAGAT 23080

cor CTAAGATTTCCAATCGTCTTAGGAATACTACCCACTAGAT 23080

man CTAAGATTTCCAATCGTCTTAGGAATACTACCCACTAGAT 23080

uni CTAAGATTTCCAATCGTCTTAGGAATACTACCCACTAGAT 23080

tall CTAAGATTTCCAATCGTCTTAGGAATACTACCCACTAGAT 23080

quil CTAAGATTTCCAATCGTCTTAGGAATACTACCCACTAGAT 23046

meri CTAAGATTTCCAATCGTCTTAGGAATACTACCCACTAGAT 23202

ref CTAAGATTTCCAATCGTCTTAGGAATACTACCCACTAGAT 23328

Consensus ctaagatttccaatcgtcttaggaatactacccactagat

kal AATTGTTGCCAATTCGAACATTGGAAAGGGCTCGACAATT 23120

yor AATTGTTGCCAATTCGAACATTGGAAAGGGCTCGACAATT 23104

jen AATTGTTGCCAATTCGAACATTGGAAAGGGCTCGACAATT 23120

cor AATTGTTGCCAATTCGAACATTGGAAAGGGCTCGACAATT 23120

man AATTGTTGCCAATTCGAACATTGGAAAGGGCTCGACAATT 23120

uni AATTGTTGCCAATTCGAACATTGGAAAGGGCTCGACAATT 23120

tall AATTGTTGCCAATTCGAACATTGGAAAGGGCTCGACAATT 23120

quil AATTGTTGCCAATTCGAACATTGGAAAGGGCTCGACAATT 23086

meri AATTGTTGCCAATTCGAACATTGGAAAGGGCTCGACAATT 23242

ref AATTGTTGCCAATTCGAACATTGGAAAGGGCTCGACAATT 23368

Consensus aattgttgccaattcgaacattggaaagggctcgacaatt

kal CCCAATTTCATCAGGAAGAACACCACTAAAATTATTCTGG 23160

yor CCCAATTTCATCAGGAAGAACACCACTAAAATTATTCTGG 23144

jen CCCAATTTCATCAGGAAGAACACCACTAAAATTATTCTGG 23160

cor CCCAATTTCATCAGGAAGAACACCACTAAAATTATTCTGG 23160

man CCCAATTTCATCAGGAAGAACACCACTAAAATTATTCTGG 23160

uni CCCAATTTCATCAGGAAGAACACCACTAAAATTATTCTGG 23160

tall CCCAATTTCATCAGGAAGAACACCACTAAAATTATTCTGG 23160

quil CCCAATTTCATCAGGAAGAACACCACTAAAATTATTCTGG 23126

meri CCCAATTTCATCAGGAAGAACACCACTAAAATTATTCTGG 23282

ref CCCAATTTCATCAGGAAGAACACCACTAAAATTATTCTGG 23408

Consensus cccaatttcatcaggaagaacaccactaaaattattctgg

kal GTGAGAACCAGAATTTCCAACTTCCCTGGAGCAAAAATGC 23200

yor GTGAGAACCAGAATTTCCAACTTCCCTGGAGCAAAAAcGC 23184

jen GTGAGAACCAGAATTTCCAACTTCCCTGGAGCAAAAATGC 23200

cor GTGAGAACCAGAATTTCCAACTTCCCTGcAGCAAAAATaC 23200

man GTGAGAACCAGAATTTCCAACTTCCCTGcAGCAAAAATaC 23200

uni GTGAGAACCAGAATTTCCAACTTCCCTGGAGCAAAAATGC 23200

tall GTGAGAACCAGAATTTCCAACTTCCCTGGAGCAAAAATGC 23200

quil GTGAGAACCAGAATTTCCAACTTCCCTGGAGCAAAAATGC 23166

meri GTGAGAACCAGAATTTCCAACTTCCCTGGAGCAAAAAcGC 23322

ref GTGAGAACCAGAATTTCCAACTTCCCTGGAGCAAAAAcGC 23448

Consensus gtgagaaccagaatttccaacttccctg agcaaaaa c

kal TTGCTGGTATTGGACCTTCAAGCTGGTTAGAGTGCAGGTT 23240

yor TTGCTGGTATTGGACCTTCAAGCTGGTTAGAGTGCAGGTT 23224

jen TTGCTGGTATTGGACCTTCAAGCTGGTTAGAGTGCAGGTT 23240

cor TTGCTGGTATTGGACCTTCAAGCTGGTTAGAGTGCAGGTT 23240

man TTGCTGGTATTGGACCTTCAAGCTGGTTAGAGTGCAGGTT 23240

uni TTGCTGGTATTGGACCTTCAAGCTGGTTAGAGTGCAGGTT 23240

tall TTGCTGGTATTGGACCTTCAAGCTGGTTAGAGTGCAGGTT 23240

quil TTGCTGGTATTGGACCTTCAAGCTGGTTAGAGTGCAGGTT 23206

meri TTGCTGGTATTGGACCTTCAAGCTGGTTAGAGTGCAGGTT 23362

ref TTGCTGGTATTGGACCTTCAAGCTGGTTAGAGTGCAGGTT 23488

Consensus ttgctggtattggaccttcaagctggttagagtgcaggtt

kal AAGTATCCGAAGCTCAGAATTCATGCCGAGACTATCCGGA 23280

yor AAGTATCCGAAGCTCAGAATTCATGCCGAGACTATCCGGA 23264

jen AAGTATCCGAAGCTCAGAATTCATGCCGAGACTATCCGGA 23280

cor AAGTATCCGAAGCTCAGAATTCATGCCGAGACTATCCGGA 23280

man AAGTATttGAAGCTCAGAAaTCgacCCGAGACTATCCGGA 23280

uni AAGTATCCGAAGCTCAGAATTCATGCCGAGACTATCCGGA 23280

tall AAGTATCCGAAGCTCAGAATTCATGCCGAGACTATCCGGA 23280

quil AAGTATCCGAAGCTCAGAATTCATGCCGAGACTATCCGGA 23246

meri AAGTATCCGAAGCTCAGAATTCATGCCGAGACTATCCGGA 23402

ref AAGTATCCGAAGCTCAGAATTCATGCCGAGACTATCCGGA 23528

Consensus aagtat gaagctcagaa tc ccgagactatccgga

kal ATCTTACCACCTAAGCTATTCTCATAAGCAGTAAAAAGTC 23320

yor ATCTTACCACCTAAGCTATTCTCATAAGCAGTAAAAAGTC 23304

jen ATCTTACCACCTAAGCTATTCTCATAAGCAGTAAAAAGTC 23320

cor ATCTTACCACCTAAGCTATTCTCATAAGCAGTAAAAAGTC 23320

man ATCcaACCACCTAAGCTATTCTCATAAGCAGTAAAAAGTC 23320

uni ATCTTACCACCTAAGCTATTCTCATAAGCAGTAAAAAGTC 23320

tall ATCTTACCACCTAAGCTATTCTCATAAGCAGTAAAAAGTC 23320

quil ATCTTACCACCTAAGCTATTCTCATAAGCAGTAAAAAGTC 23286

meri ATCTTACCACCTAAGCTATTCTCATAAGCAGTAAAAAGTC 23442

ref ATCTTACCACCTAAGCTATTCTCATAAGCAGTAAAAAGTC 23568

Consensus atc accacctaagctattctcataagcagtaaaaagtc

kal TCAAATAGCTCAAGTTTCCCACCCAAGAAGGTATCAAACC 23360

yor TCAAATAGCTCAAGTTTCCCACCCAAGAAGGTATCAAACC 23344

jen TCAAATAGCTCAAGTTTCCCACCCAAGAAGGTATCAAACC 23360

cor TCAAATAGCTCAAGTTTCCCACCCAAGAAGGTATCAAACC 23360

man TCAAATAGCTCAAGTTTCCCACCCAAGAAGGTATCAAACC 23360

uni TCAAATAGCTCAAGTTTCCCACCCAAGAAGGTATCAAACC 23360

tall TCAAATAGCTCAAGTTTCCCACCCAAGAAGGTATCAAACC 23360

quil TCAAATAGCTCAAGTTTCCCACCCAAGAAGGTATCAAACC 23326

meri TCAAATAGCTCAAGTTTCCCACCCAAGAAGGTATCAAACC 23482

ref TCAAATAGCTCAAGTTTCCCACCCAAGAAGGTATCAAACC 23608

Consensus tcaaatagctcaagtttcccacccaagaaggtatcaaacc

kal ACTCAAATGATTACTAGAAATTTGAAAATCCTGCAATTCC 23400

yor ACTCAAATGATTACTAGAAATTTGAAAATCCTGCAATTCC 23384

jen ACTCAAATGATTACTAGAAATTTGAAAATCCTGCAATTCC 23400

cor ACTCAAATGATTACTAGAAATTTGAAAATCCTGCAATTCC 23400

man ACTCAAATGATTACTAGAAATTTGAAAATCCTGCAATTCC 23400

uni ACTCAAATGATTACTAGAAATTTGAAAATCCTGCAATTCC 23400

tall ACTCAAATGATTACTAGAAATTTGAAAATCCTGCAATTCC 23400

quil ACTCAAATGATTACTAGAAATTTGAAAATCCTGCAATTCC 23366

meri ACTCAAATGATTACTAGAAATTTGAAAATCCTGCAATTCC 23522

ref ACTCAAATGATTACTAGAAATTTGAAAATCCTGCAATTCC 23648

Consensus actcaaatgattactagaaatttgaaaatcctgcaattcc

kal TCTAGGCCTTGAAGTTCCATTGGTATCTCTCCCACAAGGA 23440

yor TCTAGGCCTTGAAGTTCCATTGGTATCTCTCCCACAAGGA 23424

jen TCTAGGCCTTGAAGTTCCATTGGTATCTCTCCCACAAGGA 23440

cor TCTAGGCCTTGAAGTTCCATTGGTATCTCTCCCACAAGGA 23440

man TCTAGGCCTTGAAGTTCCATTGGTATCTCTCCCACAAGGA 23440

uni TCTAGGCCTTGAAGTTCCATTGGTATCTCTCCCACAAGGA 23440

tall TCTAGGCCTTGAAGTTCCATTGGTATCTCTCCCACAAGGA 23440

quil TCTAGGCCTTGAAGTTCCATTGGTATCTCTCCCACAAGGA 23406

meri TCTAGGCCTTGAAGTTCCATTGGTATCTCTCCCACAAGGA 23562

ref TCTAGGCCTTGAAGTTCCATTGGTATCTCTCCCACAAGGA 23688

Consensus tctaggccttgaagttccattggtatctctcccacaagga

kal CATTATTGGAAAGGTTCAATGACTTGAGGCTTCTAAGGAC 23480

yor CATTATTGGAAAGGTTCAATGACTTGAGGCTTCTAAGGAC 23464

jen CATTATTGGAAAGGTTCAATGACTTGAGGCTTCTAAGGAC 23480

cor CATTATTGGAAAGGTTCAATGACTTGAGGCTTCTAAGGAC 23480

man CATTATTGGAAAGGTTCAATGACTTGAGGCTTCTAAGGAC 23480

uni CATTATTGGAAAGGTTCAATGACTTGAGGCTTCTAAGGAC 23480

tall CATTATTGGAAAGGTTCAATGACTTGAGGCTTCTAAGGAC 23480

quil CATTATTGGAAAGGTTCAATGACTTGAGGCTTCTAAGGAC 23446

meri CATTATTGGAAAGGTTCAATGACTTGAGGCTTCTAAGGAC 23602

ref CATTATTGGAAAGGTTCAATGACTTGAGGCTTCTAAGGAC 23728

Consensus cattattggaaaggttcaatgacttgaggcttctaaggac

kal ACCCAATTGTGGTGGAATTGACCCTCTAAACTTATTTGAA 23520

yor ACCCAATTGTGGTGGAATTGACCCTCTAAACTTATTTGAA 23504

jen ACCCAATTGTGGTGGAATTGACCCTCTAAACTTATTTGAA 23520

cor ACCCAATTGTGGTGGAATTGACCCTCTAAACTTATTTGAA 23520

man ACCCAATTGTGGTGGAATTGACCCTCTAAACTTATTTGAA 23520

uni ACCCAATTGTGGTGGAATTGACCCTCTAAACTTATTTGAA 23520

tall ACCCAATTGTGGTGGAATTGACCCTCTAAACTTATTTGAA 23520

quil ACCCAATTGTGGTGGAATTGACCCTCTAAACTTATTTGAA 23486

meri ACCCAATTGTGGTGGAATTGACCCTCTAAACTTATTTGAA 23642

ref ACCCAATTGTGGTGGAATTGACCCTCTAAACTTATTTGAA 23768

Consensus acccaattgtggtggaattgaccctctaaacttatttgaa

kal GACAAATCTAGAACTTCAAGATGAGATAAATTTCCAAAAG 23560

yor GACAAATCTAGAACTTCAAGATGAGATAAATTTCCAAAAG 23544

jen GACAAATCTAGAACTTCAAGATGAGATAAATTTCCAAAAG 23560

cor GACAAATCTAGAACTTCAAGATGAGATAAATTTCCAAAAG 23560

man GACAAATCTAGAACTTCAAGATGAGATAAATTTCCAAAAG 23560

uni GACAAATCTAGAACTTCAAGATGAGATAAATTTCCAAAAG 23560

tall GACAAATCTAGAACTTCAAGATGAGATAAATTTCCAAAAG 23560

quil GACAAATCTAGAACTTCAAGATGAGATAAATTTCCAAAAG 23526

meri GACAAATCTAGAACTTCAAGATGAGATAAATTTCCAAAAG 23682

ref GACAAATCTAGAACTTCAAGATGAGATAAATTTCCAAAAG 23808

Consensus gacaaatctagaacttcaagatgagataaatttccaaaag

kal CAGTAGGAATCAAACCATCAAAGTTATTGTTTGAAAGGTC 23600

yor CAGTAGGAATCAAACCATCAAAGTTATTGTTTGAAAGGTC 23584

jen CAGTAGGAATCAAACCATCAAAGTTATTGTTTGAAAGGTC 23600

cor CAGTAGGAATCAAACCATCAAAGTTATTGTTTGAAAGGTC 23600

man CAGTAGGAATCAAACCATCAAAGTTATTGTTTGAAAGGTC 23600

uni CAGTAGGAATCAAACCATCAAAGTTATTGTTTGAAAGGTC 23600

tall CAGTAGGAATCAAACCATCAAAGTTATTGTTTGAAAGGTC 23600

quil CAGTAGGAATCAAACCATCAAAGTTATTGTTTGAAAGGTC 23566

meri CAGTAGGAATCAAACCATCAAAGTTATTGTTTGAAAGGTC 23722

ref CAGTAGGAATCAAACCATCAAAGTTATTGTTTGAAAGGTC 23848

Consensus cagtaggaatcaaaccatcaaagttattgtttgaaaggtc

kal AAGCTTCTTCAAAGCTTTGAGCTCAGAAATTAAAGTTACA 23640

yor AAGCTTCTTCAAAGCTTTGAGCTCAGAAATTAAAGTTACA 23624

jen AAGCTTCTTCAAAGCTTTGAGCTCAGAAATTAAAGTTACA 23640

cor AAGCTTCTTCAAAGCTTTGAGCTCAGAAATTAAAGTTACA 23640

man AAGCTTCTTCAAAGCTTTGAGCTCAGAAATTAAAGTTACA 23640

uni AAGCTTCTTCAAAGCTTTGAGCTCAGAAATTAAAGTTACA 23640

tall AAGCTTCTTCAAAGCTTTGAGCTCAGAAATTAAAGTTACA 23640

quil AAGCTTCTTCAAAGCTTTGAGCTCAGAAATTAAAGTTACA 23606

meri AAGCTTCTTCAAAGCTTTGAGCTCAGAAATTAAAGTTACA 23762

ref AAGCTTCTTCAAAGCTTTGAGCTCAGAAATTAAAGTTACA 23888

Consensus aagcttcttcaaagctttgagctcagaaattaaagttaca

kal TTACCTCGAAGATTCCGGTGAGCTAGATCAATCTTCTCCA 23680

yor TTACCTCGAAGATTCCGGTGAGCTAGATCAATCTTCTCCA 23664

jen TTACCTCGAAGATTCCGGTGAGCTAGATCAATCTTCTCCA 23680

cor TTACCTCGAAGATTCCGGTGAGCTAGATCAATCTTCTCCA 23680

man TTACCTCGAAGATTCCGGTGAGCTAGATCAATCTTCTCCA 23680

uni TTACCTCGAAGATTCCGGTGAGCTAGATCAATCTTCTCCA 23680

tall TTACCTCGAAGATTCCGGTGAGCTAGATCAATCTTCTCCA 23680

quil TTACCTCGAAGATTCCGGTGAGCTAGATCAATCTTCTCCA 23646

meri TTACCTCGAAGATTCCGGTGAGCTAGATCAATCTTCTCCA 23802

ref TTACCTCGAAGATTCCGGTGAGCTAGATCAATCTTCTCCA 23928

Consensus ttacctcgaagattccggtgagctagatcaatcttctcca

kal CCATTGAGTGATTACCACATGTGATTCCTTGCCAAGTGCA 23720

yor CCATTGAGTGATTACCACATGTGATTCCTTGCCAAGTGCA 23704

jen CCATTGAGTGATTACCACATGTGATTCCTTGCCAAGTGCA 23720

cor CCATTGAGTGATTACCACATGTGATTCCTTGCCAAGTGCA 23720

man CCATTGAGTGATTACCACATGTGATTCCTTGCCAAGTGCA 23720

uni CCATTGAGTGATTACCACATGTGATTCCTTGCCAAGTGCA 23720

tall CCATTGAGTGATTACCACATGTGATTCCTTGCCAAGTGCA 23720

quil CCATTGAGTGATTACCACATGTGATTCCTTGCCAAGTGCA 23686

meri CCATTGAGTGATTACCACATGTGATTCCTTGCCAAGTGCA 23842

ref CCATTGAGTGATTACCACATGTGATTCCTTGCCAAGTGCA 23968

Consensus ccattgagtgattaccacatgtgattccttgccaagtgca

kal GTAATTTGAGGTGTTGCCATCACTCCATTCAGGGACTCTA 23760

yor GTAATTTGAGGTGTTGCCATCACTCCATTCAGGGACTCTA 23744

jen GTAATTTGAGGTGTTGCCATCACTCCATTCAGGGACTCTA 23760

cor GTAATTTGAGGTGTTGCCATCACTCCATTCAGGGACTCTA 23760

man GTAATTTGAGGTGTTGCCATCACTCCATTCAGGGACTCTA 23760

uni GTAATTTGAGGTGTTGCCATCACTCCATTCAGGGACTCTA 23760

tall GTAATTTGAGGTGTTGCCATCACTCCATTCAGGGACTCTA 23760

quil GTAATTTGAGGTGTTGCCATCACTCCATTCAGGGACTCTA 23726

meri GTAATTTGAGGTGTTGCCATCACTCCATTCAGGGACTCTA 23882

ref GTAATTTGAGGTGTTGCCATCACTCCATTCAGGGACTCTA 24008

Consensus gtaatttgaggtgttgccatcactccattcagggactcta

kal AGCTCTTGATTGATAGCAAGTAAAATAGCTTGGTCTTGAA 23800

yor AGCTCTTGATTGATAGCAAGTAAAATAGCTTGGTCTTGAA 23784

jen AGCTCTTGATTGATAGCAAGTAAAATAGCTTGGTCTTGAA 23800

cor AGCTCTTGATTGATAGCAAGTAAAATAGCTTGGTCTTGAA 23800

man AGCTCTTGATTGATAGCAAGTAAAATAGCTTGGTCTTGAA 23800

uni AGCTCTTGATTGATAGCAAGTAAAATAGCTTGGTCTTGAA 23800

tall AGCTCTTGATTGATAGCAAGTAAAATAGCTTGGTCTTGAA 23800

quil AGCTCTTGATTGATAGCAAGTAAAATAGCTTGGTCTTGAA 23766

meri AGCTCTTGATTGATAGCAAGTAAAATAGCTTGGTCTTGAA 23922

ref AGCTCTTGATTGATAGCAAGTAAAATAGCTTGGTCTTGAA 24048

Consensus agctcttgattgatagcaagtaaaatagcttggtcttgaa

kal GCTCAGCAGCACCTACAAGGTGAAAAATTGACACATACCA 23840

yor GCTCAGCAGCACCTACAAGGTGAAAAATTGACACATACCA 23824

jen GCTCAGCAGCACCTACAAGGTGAAAAATTGACACATACCA 23840

cor GCTCAGCAGCACCTACAAGGTGAAAAATTGACACATACCA 23840

man GCTCAGCAGCACCTACAAGGTGAAAAATTGACACATACCA 23840

uni GCTCAGCAGCACCTACAAGGTGAAAAATTGACACATACCA 23840

tall GCTCAGCAGCACCTACAAGGTGAAAAATTGACACATACCA 23840

quil GCTCAGCAGCACCTACAAGGTGAAAAATTGACACATACCA 23806

meri GCTCAGCAGCACCTACAAGGTGAAAAATTGACACATACCA 23962

ref GCTCAGCAGCACCTACAAGGTGAAAAATTGACACATACCA 24088

Consensus gctcagcagcacctacaaggtgaaaaattgacacatacca

kal AGCTAGCAGCAACAAAAAGCACAAAACTTCCATCACTTCA 23880

yor AGCTAGCAGCAACAAAAAGCACAAAACTTCCATCACTTCA 23864

jen AGCTAGCAGCAACAAAAAGCACAAAACTTCCATCACTTCA 23880

cor AGCTAGCAGCAACAAAAAGCACAAAACTTCCATCACTTCA 23880

man AGCTAGCAGCAACAAAAAGCACAAAACTTCCATCACTTCA 23880

uni AGCTAGCAGCAACAAAAAGCACAAAACTTCCATCACTTCA 23880

tall AGCTAGCAGCAACAAAAAGCACAAAACTTCCATCACTTCA 23880

quil AGCTAGCAGCAACAAAAAGCACAAAACTTCCATCACTTCA 23846

meri AGCTAGCAGCAACAAAAAGCACAAAACTTCCATCACTTCA 24002

ref AGCTAGCAGCAACAAAAAGCACAAAACTTCCATCACTTCA 24128

Consensus agctagcagcaacaaaaagcacaaaacttccatcacttca

kal CACTGTTCTATGCTGAAGTGAAGTGAAATGTTAGTGCTTC 23920

yor CACTGTTCTATGCTGAAGTGAAGTGAAATGTTAGTGCTTC 23904

jen CACTGTTCTATGCTGAAGTGAAGTGAAATGTTAGTGCTTC 23920

cor CACTGTTCTATGCTGAAGTGAAGTGAAATGTTAGTGCTTC 23920

man CACTGTTCTATGCTGAAGTGAAGTGAAATGTTAGTGCTTC 23920

uni CACTGTTCTATGCTGAAGTGAAGTGAAATGTTAGTGCTTC 23920

tall CACTGTTCTATGCTGAAGTGAAGTGAAATGTTAGTGCTTC 23920

quil CACTGTTCTATGCTGAAGTGAAGTGAAATGTTAGTGCTTC 23886

meri CACTGTTCTATGCTGAAGTGAAGTGAAATGTTAGTGCTTC 24042

ref CACTGTTCTATGCTGAAGTGAAGTGAAATGTTAGTGCTTC 24168

Consensus cactgttctatgctgaagtgaagtgaaatgttagtgcttc

kal CACTCCTCTCAACAGTGTTAGAGGCTCCACAACATCAACA 23960

yor CACTCCTCTCAACAGTGTTAGAGGCTCCACAACATCAACA 23944

jen CACTCCTCTCAACAGTGTTAGAGGCTCCACAACATCAACA 23960

cor CACTCCTCTCAACAGTGTTAGAGGCTCCACAACATCAACA 23960

man CACTCCTCTCAACAGTGTTAGAGGCTCCACAACATCAACA 23960

uni CACTCCTCTCAACAGTGTTAGAGGCTCCACAACATCAACA 23960

tall CACTCCTCTCAACAGTGTTAGAGGCTCCACAACATCAACA 23960

quil CACTCCTCTCAACAGTGTTAGAGGCTCCACAACATCAACA 23926

meri CACTCCTCTCAACAGTGTTAGAGGCTCCACAACATCAACA 24082

ref CACTCCTCTCAACAGTGTTAGAGGCTCCACAACATCAACA 24208

Consensus cactcctctcaacagtgttagaggctccacaacatcaaca

kal AGTTTTGCTTCTTTTCACTCAAAAAATAAAACTTTTCTTC 24000

yor AGTTTTGCTTCTTTTCACTCAAAAAATAAAACTTTTCTTC 23984

jen AGTTTTGCTTCTTTTCACTCAAAAAATAAAACTTTTCTTC 24000

cor AGTTTTGCTTCTTTTCACTCAAAAAATAAAACTTTTCTTC 24000

man AGTTTTGCTTCTTTTCACTCAAAAAATAAAACTTTTCTTC 24000

uni AGTTTTGCTTCTTTTCACTCAAAAAATAAAACTTTTCTTC 24000

tall AGTTTTGCTTCTTTTCACTCAAAAAATAAAACTTTTCTTC 24000

quil AGTTTTGCTTCTTTTCACTCAAAAAATAAAACTTTTCTTC 23966

meri AGTTTTGCTTCTTTTCACTCAAAAAATAAAACTTTTCTTC 24122

ref AGTTTTGCTTCTTTTCACTCAAAAAATAAAACTTTTCTTC 24248

Consensus agttttgcttcttttcactcaaaaaataaaacttttcttc

kal ACAATGAACTTCCCCCTTTTCTTTATTTTGCACAAGTGAG 24040

yor ACAATGAACTTCCCCCTTTTCTTTATTTTGCACAAGTGAG 24024

jen ACAATGAACTTCCCCCTTTTCTTTATTTTGCACAAGTGAG 24040

cor ACAATGAACTTCCCCCTTTTCTTTATTTTGCACAAGTGAG 24040

man ACAATGAACTTCCCCCTTTTCTTTATTTTGCACAAGTGAG 24040

uni ACAATGAACTTCCCCCTTTTCTTTATTTTGCACAAGTGAG 24040

tall ACAATGAACTTCCCCCTTTTCTTTATTTTGCACAAGTGAG 24040

quil ACAATGAACTTCCCCCTTTTCTTTATTTTGCACAAGTGAG 24006

meri ACAATGAACTTCCCCCTTTTCTTTATTTTGCACAAGTGAG 24162

ref ACAATGAACTTCCCCCTTTTCTTTATTTTGCACAAGTGAG 24288

Consensus acaatgaacttcccccttttctttattttgcacaagtgag

kal GATTTGGAAAGAGATTTTCCAATTAGGAACAAAGTTTCAC 24080

yor GATTTGGAAAGAGATTTTCCAATTAGGAACAAAGTTTCAC 24064

jen GATTTGGAAAGAGATTTTCCAATTAGGAACAAAGTTTCAC 24080

cor GATTTGGAAAGAGATTTTCCAATTAGGAACAAAGTTTCAC 24080

man GATTTGGAAAGAGATTTTCCAATTAGGAACAAAGTTTCAC 24080

uni GATTTGGAAAGAGATTTTCCAATTAGGAACAAAGTTTCAC 24080

tall GATTTGGAAAGAGATTTTCCAATTAGGAACAAAGTTTCAC 24080

quil GATTTGGAAAGAGATTTTCCAATTAGGAACAAAGTTTCAC 24046

meri GATTTGGAAAGAGATTTTCCAATTAGGAACAAAGTTTCAC 24202

ref GATTTGGAAAGAGATTTTCCAATTAGGAACAAAGTTTCAC 24328

Consensus gatttggaaagagattttccaattaggaacaaagtttcac

kal TTGCTTCAATGAAGATTCATAGCTTCAAATAGAGGCCCCT 24120

yor TTGCTTCAATGAAGATTCATAGCTTCAAATAGAGGCCCCT 24104

jen TTGCTTCAATGAAGATTCATAGCTTCAAATAGAGGCCCCT 24120

cor TTGCTTCAATGAAGATTCATAGCTTCAAATAGAGGCCCCT 24120

man TTGCTTCAATGAAGATTCATAGCTTCAAATAGAGGCCCCT 24120

uni TTGCTTCAATGAAGATTCATAGCTTCAAATAGAGGCCCCT 24120

tall TTGCTTCAATGAAGATTCATAGCTTCAAATAGAGGCCCCT 24120

quil TTGCTTCAATGAAGATTCATAGCTTCAAATAGAGGCCCCT 24086

meri TTGCTTCAATGAAGATTCATAGCTTCAAATAGAGGCCCCT 24242

ref TTGCTTCAATGAAGATTCATAGCTTCAAATAGAGGCCCCT 24368

Consensus ttgcttcaatgaagattcatagcttcaaatagaggcccct

kal GAAAATGCAGAAACCACAGATCACAAAACATATCAACATT 24160

yor GAAAATGCAGAAACCACAGATCACAAAACATATCAACATT 24144

jen GAAAATGCAGAAACCACAGATCACAAAACATATCAACATT 24160

cor GAAAATGCAGAAACCACAGATCACAAAACATATCAACATT 24160

man GAAAATGCAGAAACCACAGATCACAAAACATATCAACATT 24160

uni GAAAATGCAGAAACCACAGATCACAAAACATATCAACATT 24160

tall GAAAATGCAGAAACCACAGATCACAAAACATATCAACATT 24160

quil GAAAATGCAGAAACCACAGATCACAAAACATATCAACATT 24126

meri GAAAATGCAGAAACCACAGATCACAAAACATATCAACATT 24282

ref GAAAATGCAGAAACCACAGATCACAAAACATATCAACATT 24408

Consensus gaaaatgcagaaaccacagatcacaaaacatatcaacatt

kal TTGAAGAAAACCCATTTGAGATAATCTGAAATGAAAAAAA 24200

yor TTGAAGAAAACCCATTTGAGATAATCTGAAATGAAAAAAA 24184

jen TTGAAGAAAACCCATTTGAGATAATCTGAAATGAAAAAAA 24200

cor TTGAAGAAAACCCATTTGAGATAATCTGAAATGAAAAAAA 24200

man TTGAAGAAAACCCATTTGAGATAATCTGAAATGAAAAAAA 24200

uni TTGAAGAAAACCCATTTGAGATAATCTGAAATGAAAAAAA 24200

tall TTGAAGAAAACCCATTTGAGATAATCTGAAATGAAAAAAA 24200

quil TTGAAGAAAACCCATTTGAGATAATCTGAAATGAAAAAAA 24166

meri TTGAAGAAAACCCATTTGAGATAATCTGAAATGAAAAAAA 24322

ref TTGAAGAAAACCCATTTGAGATAATCTGAAATGAAAAAAA 24448

Consensus ttgaagaaaacccatttgagataatctgaaatgaaaaaaa

kal AAA..GTGCTGTGCTACTATTATAGACATAAAGTAAAGAT 24238

yor AAAaaGTGCTGTGCTACTATTATAGACATAAAGTAAAGAT 24224

jen AAA..GTGCTGTGCTACTATTATAGACATAAAGTAAAGAT 24238

cor AAA..GTGCTGTGCTACTATTATAGACATAAAGTAAAGAT 24238

man AAA..GTGCTGTGCTACTATTATAGACATAAAGTAAAGAT 24238

uni AAA..GTGCTGTGCTACTATTATAGACATAAAGTAAAGAT 24238

tall AAA..GTGCTGTGCTACTATTATAGACATAAAGTAAAGAT 24238

quil AAA..GTGCTGTGCTACTATTATAGACATAAAGTAAAGAT 24204

meri AAA..GTGCTGTGCTACTATTATAGACATAAAGTAAAGAT 24360

ref AAAa.GTGCTGTGCTACTATTATAGACATAAAGTAAAGAT 24487

Consensus aaa gtgctgtgctactattatagacataaagtaaagat

kal TTTTTTTAATAATAATAGAAAATGGGAACTACCCAAAAGT 24278

yor TTTTTTTAATAATAATAGAAAATGGGAACTACCCAAAAGT 24264

jen TTTTTTTAATAATAATAGAAAATGGGAACTACCCAAAAGT 24278

cor TTTTTTTAATAATAATAGAAAATGGGAACTACCCAAAAGT 24278

man TTTTTTTAATAATAATAGAAAATGGGAACTACCCAAAAGT 24278

uni TTTTTTTAATAATAATAGAAAATGGGAACTACCCAAAAGT 24278

tall TTTTTTTAATAATAATAGAAAATGGGAACTACCCAAAAGT 24278

quil TTTTTTTAATAATAATAGAAAATGGGAACTACCCAAAAGT 24244

meri TTTTTTTAATAATAATAGAAAATGGGAACTACCCAAAAGT 24400

ref TTTTTTTAATAATAATAGAAAATGGGAACTACCCAAAAGT 24527

Consensus tttttttaataataatagaaaatgggaactacccaaaagt

kal TTAAATTTATTTATTAAAAAGGGAAGATAAATAAAAAGAA 24318

yor TTAAATTTATTTATTAAAAAGGGAAGATAAATAAAAAGAA 24304

jen TTAAATTTATTTATTAAAAAGGGAAGATAAATAAAAAGAA 24318

cor TTAAATTTATTTATTAAAAAGGGAAGATAAATAAAAAGAA 24318

man TTAAATTTATTTATTAAAAAGGGAAGATAAATAAAAAGAA 24318

uni TTAAATTTATTTATTAAAAAGGGAAGATAAATAAAAAGAA 24318

tall TTAAATTTATTTATTAAAAAGGGAAGATAAATAAAAAGAA 24318

quil TTAAATTTATTTATTAAAAAGGGAAGATAAATAAAAAGAA 24284

meri TTAAATTTATTTATTAAAAAGGGAAGATAAATAAAAAGAA 24440

ref TTAAATTTATTTATTAAAAAGGGAAGATAAATAAAAAGAA 24567

Consensus ttaaatttatttattaaaaagggaagataaataaaaagaa

kal AGGAAGGGTGCATTATGAAGCATGGGTTGGTTACATATGA 24358

yor AGGAAGGGTGCATTATGAAGCATGGGTTGGTTACATATGA 24344

jen AGGAAGGGTGCATTATGAAGCATGGGTTGGTTACATATGA 24358

cor AGGAAGGGTGCATTATGAAGCATGGGTTGGTTACATATGA 24358

man AGGAAGGGTGCATTATGAAGCATGGGTTGGTTACATATGA 24358

uni AGGAAGGGTGCATTATGAAGCATGGGTTGGTTACATATGA 24358

tall AGGAAGGGTGCATTATGAAGCATGGGTTGGTTACATATGA 24358

quil AGGAAGGGTGCATTATGAAGCATGGGTTGGTTACATATGA 24324

meri AGGAAGGGTGCATTATGAAGCATGGGTTGGTTACATATGA 24480

ref AGGAAGGGTGCATTATGAAGCATGGGTTGGTTACATATGA 24607

Consensus aggaagggtgcattatgaagcatgggttggttacatatga

kal AAGTAAAACAAAAAGCATTGCATAATAGTTAGTAGAAGAA 24398

yor AAGTAAAACAAAAAGCATTGCATAATAGTTAGTAGAAGAA 24384

jen AAGTAAAACAAAAAGCATTGCATAATAGTTAGTAGAAGAA 24398

cor AAGTAAAACAAAAAGCATTGCATAATAGTTAGTAGAAGAA 24398

man AAGTAAAACAAAAAGCATTGCATAATAGTTAGTAGAAGAA 24398

uni AAGTAAAACAAAAAGCATTGCATAATAGTTAGTAGAAGAA 24398

tall AAGTAAAACAAAAAGCATTGCATAATAGTTAGTAGAAGAA 24398

quil AAGTAAAACAAAAAGCATTGCATAATAGTTAGTAGAAGAA 24364

meri AAGTAAAACAAAAAGCATTGCATAATAGTTAGTAGAAGAA 24520

ref AAGTAAAACAAAAAGCATTGCATAATAGTTAGTAGAAGAA 24647

Consensus aagtaaaacaaaaagcattgcataatagttagtagaagaa

kal GTGTAGAAGGAAGATAGAAAGTGACCTTAGTAAGGAATGA 24438

yor GTGTAGAAGGAAGATAGAAAGTGACCTTAGTAAGGAATGA 24424

jen GTGTAGAAGGAAGATAGAAAGTGACCTTAGTAAGGAATGA 24438

cor GTGTAGAAGGAAGATAGAAAGTGACCTTAGTAAGGAATGA 24438

man GTGTAGAAGGAAGATAGAAAGTGACCTTAGTAAGGAATGA 24438

uni GTGTAGAAGGAAGATAGAAAGTGACCTTAGTAAGGAATGA 24438

tall GTGTAGAAGGAAGATAGAAAGTGACCTTAGTAAGGAATGA 24438

quil GTGTAGAAGGAAGATAGAAAGTGACCTTAGTAAGGAATGA 24404

meri GTGTAGAAGGAAGATAGAAAGTGACCTTAGTAAGGAATGA 24560

ref GTGTAGAAGGAAGATAGAAAGTGACCTTAGTAAGGAATGA 24687

Consensus gtgtagaaggaagatagaaagtgaccttagtaaggaatga

kal AGAAAGAGTGATGAAAATGAATGAGAGATTGAAGGAACTA 24478

yor AGAAAGAGTGATGAAAATGAATGAGAGATTGAAGGAACTA 24464

jen AGAAAGAGTGATGAAAATGAATGAGAGATTGAAGGAACTA 24478

cor AGAAAGAGTGATGAAAATGAATGAGAGATTGAAGGAACTA 24478

man AGAAAGAGTGATGAAAATGAATGAGAGATTGAAGGAACTA 24478

uni AGAAAGAGTGATGAAAATGAATGAGAGATTGAAGGAACTA 24478

tall AGAAAGAGTGATGAAAATGAATGAGAGATTGAAGGAACTA 24478

quil AGAAAGAGTGATGAAAATGAATGAGAGATTGAAGGAACTA 24444

meri AGAAAGAGTGATGAAAATGAATGAGAGATTGAAGGAACTA 24600

ref AGAAAGAGTGATGAAAATGAATGAGAGATTGAAGGAACTA 24727

Consensus agaaagagtgatgaaaatgaatgagagattgaaggaacta

kal ATGCTCTTTGGTACAGACAAGGTTCACGGACCAGGTGTTT 24518

yor ATGCTCTTTGGTACAGACAAGGTTCACGGACCAGGTGTTT 24504

jen ATGCTCTTTGGTACAGACAAGGTTCACGGACCAGGTGTTT 24518

cor ATGCTCTTTGGTACAGACAAGGTTCACGGACCAGGTGTTT 24518

man ATGCTCTTTGGTACAGACAAGGTTCACGGACCAGGTGTTT 24518

uni ATGCTCTTTGGTACAGACAAGGTTCACGGACCAGGTGTTT 24518

tall ATGCTCTTTGGTACAGACAAGGTTCACGGACCAGGTGTTT 24518

quil ATGCTCTTTGGTACAGACAAGGTTCACGGACCAGGTGTTT 24484

meri ATGCTCTTTGGTACAGACAAGGTTCACGGACCAGGTGTTT 24640

ref ATGCTCTTTGGTACAGACAAGGTTCACGGACCAGGTGTTT 24767

Consensus atgctctttggtacagacaaggttcacggaccaggtgttt

kal TCATGTGTCACAAACACAGAGAGAGAGAGAGAGAGAGAGA 24558

yor TCATGTGTCACAAACACAGAGAGAGAGAGAGAGAGAGAGA 24544

jen TCATGTGTCACAAACACAGAGAGAGAGAGAGAGAGAGAGA 24558

cor TCATGTGTCACAAACACAGAGAGAGAGAGAGAGAGAGAGA 24558

man TCATGTGTCACAAACACAGAGAGAGAGAGAGAGAGAGAGA 24558

uni TCATGTGTCACAAACACAGAGAGAGAGAGAGAGAGAGAGA 24558

tall TCATGTGTCACAAACACAGAGAGAGAGAGAGAGAGAGAGA 24558

quil TCATGTGTCACAAACACAGAGAGAGAGAGAGAGAGAGAGA 24524

meri TCATGTGTCACAAACACAGAGAGAGAGAGAGAGAGAGAGA 24680

ref TCATGTGTCACAAACACAGAGAGAGAGAGAGAGAGAGAGA 24807

Consensus tcatgtgtcacaaacacagagagagagagagagagagaga

kal G..G........TTGAGAGAGATGAATTTTCCCAAGTAAG 24588

yor GagG........gTGAGAGAGATGAATTTTCCCAAGTAAG 24576

jen G..G........TTGAGAGAGATGAATTTTCCCAAGTAAG 24588

cor G..G........TTGAGAGAGATGAATTTTCCCAAGTAAG 24588

man G..G........TTGAGAGAGATGAATTTTCCCAAGTAAG 24588

uni G..G........TTGAGAGAGATGAATTTTCCCAAGTAAG 24588

tall G..G........TTGAGAGAGATGAATTTTCCCAAGTAAG 24588

quil G..G........TTGAGAGAGATGAATTTTCCCAAGTAAG 24554

meri Gaga..................TGAATTTTCCCAAGTAAG 24702

ref GagagagagaggTTGAGAGAGATGAATTTTCCCAAGTAAG 24847

Consensus g tgaattttcccaagtaag

kal CTGGGGTGGGTTGTTCACGAGAAATGGGCAGTGATGAGTG 24628

yor CTGGGGTGGGTTGTTCACGAGAAATGGGCAGTGATGAGTG 24616

jen CTGGGGTGGGTTGTTCACGAGAAATGGGCAGTGATGAGTG 24628

cor CTGGGGTGGGTTGTTCACGAGAAATGGGCAGTGATGAGTG 24628

man CTGGGGTGGGTTGTTCACGAGAAATGGGCAGTGATGAGTG 24628

uni CTGGGGTGGGTTGTTCACGAGAAATGGGCAGTGATGAGTG 24628

tall CTGGGGTGGGTTGTTCACGAGAAATGGGCAGTGATGAGTG 24628

quil CTGGGGTGGGTTGTTCACGAGAAATGGGCAGTGATGAGTG 24594

meri CTGGGGTGGGTTGTTCACGAGAAATGGGCAGTGATGAGTG 24742

ref CTGGGGTGGGTTGTTCACGAGAAATGGGCAGTGATGAGTG 24887

Consensus ctggggtgggttgttcacgagaaatgggcagtgatgagtg

kal AATGAAAGCTGCATAAAACTCCTGTCTCACTGCATGTGGG 24668

yor AATGAAAGCTGCATAAAACTCCTGTCTCACTGCATGTGGG 24656

jen AATGAAAGCTGCATAAAACTCCTGTCTCACTGCATGTGGG 24668

cor AATGAAAGCTGCATAAAACTCCTGTCTCACTGCATGTGGG 24668

man AATGAAAGCTGCATAAAACTCCTGTCTCACTGCATGTGGG 24668

uni AATGAAAGCTGCATAAAACTCCTGTCTCACTGCATGTGGG 24668

tall AATGAAAGCTGCATAAAACTCCTGTCTCACTGCATGTGGG 24668

quil AATGAAAGCTGCATAAAACTCCTGTCTCACTGCATGTGGG 24634

meri AATGAAAGCTGCATAAAACTCCTGTCTCACTGCATGTGGG 24782

ref AATGAAAGCTGCATAAAACTCCTGTCTCACTGCATGTGGG 24927

Consensus aatgaaagctgcataaaactcctgtctcactgcatgtggg

kal GCACACTCAAACATCAACTGTTTCAGCTTCTGAGTTGGAC 24708

yor GCACACTCAAACATCAACTGTTTCAGCTTCTGAGTTGGAC 24696

jen GCACACTCAAACATCAACTGTTTCAGCTTCTGAGTTGGAC 24708

cor GCACACTCAAACATCAACTGTTTCAGCTTCTGAGTTGGAC 24708

man GCACACTCAAACATCAACTGTTTCAGCTTCTGAGTTGGAC 24708

uni GCACACTCAAACATCAACTGTTTCAGCTTCTGAGTTGGAC 24708

tall GCACACTCAAACATCAACTGTTTCAGCTTCTGAGTTGGAC 24708

quil GCACACTCAAACATCAACTGTTTCAGCTTCTGAGTTGGAC 24674

meri GCACACTCAAACATCAACTGTTTCAGCTTCTGAGTTGGAC 24822

ref GCACACTCAAACATCAACTGTTTCAGCTTCTGAGTTGGAC 24967

Consensus gcacactcaaacatcaactgtttcagcttctgagttggac

kal CATAAACCTTAAAAGTGGGTGTATTCTCGATATAAAGATT 24748

yor CATAAACCTTAAAAGTccaTGTATTtTCGATATAAAaATT 24736

jen CATAAACCTTAAAAGTGGGTGTATTCTCGATATAAAGATT 24748

cor CATAAACCTTAAAAGTGGGTGTATTCTCGATATAAAGATT 24748

man CATAAACCTTAAAAGTGGGTGTATTCTCGATATAAAGATT 24748

uni CATAAACCTTAAAAGTGGGTGTATTCTCGATATAAAGATT 24748

tall CATAAACCTTAAAAGTGGGTGTATTCTCGATATAAAGATT 24748

quil CATAAACCTTAAAAGTGGGTGTATTCTCGATATAAAGATT 24714

meri CATAAACCTTAAAAGTctaTGTATTtTCGATATAAAaATT 24862

ref CATAAACCTTAAAAGTccaTGTATTtTCGATATAAAaATT 25007

Consensus cataaaccttaaaagt tgtatt tcgatataaa att

kal TATACACAATTAATTTATTATTCATCATTCAATTTTATAA 24788

yor TATACACAATcAATTTATTATTCgTCATT........... 24765

jen TATACACAATTAATTTATTATTCATCATTCAATTTTATAA 24788

cor TATACACAATTAATTTATTATTCATCATTCAATTTTATAA 24788

man TATACACAATTAATTTATTATTCATCATTCAATTTTATAA 24788

uni TATACACAATTAATTTATTATTCATCATTCAATTTTATAA 24788

tall TATACACAATTAATTTATTATTCATCATTCAATTTTATAA 24788

quil TATACACAATTAATTTATTATTCATCATTCAATTTTATAA 24754

meri TATACACAATcAATTTATTATTtgTCATT........... 24891

ref TATACACAATcAATTTATTATTCgTCATT........... 25036

Consensus tatacacaat aatttattatt tcatt

kal TTAAATATGTTAAATATAAATAATAGATGATGCAAGATAA 24828

yor .........TTAAATATgAATAATAGATGATGtAAGATgA 24796

jen TTAAATATGTTAAATATAAATAATAGATGATGCAAGATAA 24828

cor TTAAATATGTTAAATATAAATAATAGATGATGCAAGATAA 24828

man TTAAATATGTTAAATATAAATAATAGATGATGCAAGATAA 24828

uni TTAAATATGTTAAATATAAATAATAGATGATGCAAGATAA 24828

tall TTAAATATGTTAAATATAAATAATAGATGATGCAAGATAA 24828

quil TTAAATATGTTAAATATAAATAATAGATGATGCAAGATAA 24794

meri .........TTAAATATgAATAATAGATGATGtAAGATgA 24922

ref .........TTAAATATgAATAATAGATGATGtAAGATgA 25067

Consensus ttaaatat aataatagatgatg aagat a

kal GGTGAGTGAGATTAGTTAAAATTATATAGTTAAAGTTCTG 24868

yor GGTGAGTGAGATTAGTTAAAATTAgATAGTTAAAGTTCTG 24836

jen GGTGAGTGAGATTAGTTAAAATTATATAGTTAAAGTTCTG 24868

cor GGTGAGTGAGATTAGTTAAAATTATATAGTTAAAGTTCTG 24868

man GGTGAGTGAGATTAGTTAAAATTATATAGTTAAAGTTCTG 24868

uni GGTGAGTGAGATTAGTTAAAATTATATAGTTAAAGTTCTG 24868

tall GGTGAGTGAGATTAGTTAAAATTATATAGTTAAAGTTCTG 24868

quil GGTGAGTGAGATTAGTTAAAATTATATAGTTAAAGTTCTG 24834

meri GGTGAaTGAGATTAGTTAAAATTAgATAGTTAAAGTTCTG 24962

ref GGTGAGTGAGATTAGTTAAAATTAgATAGTTAAAGTTCTG 25107

Consensus ggtga tgagattagttaaaatta atagttaaagttctg

kal TAAATGTGAGTAATGTATCCGTCTATATTTAAATGTTTGA 24908

yor TAAATGTGAGTAgTGTATCtGTCTATATTTAAATGTTTaA 24876

jen TAAATGTGAGTAATGTATCCGTCTATATTTAAATGTTTGA 24908

cor TAAATGTGAGTAATGTATCCGTCTATATTTAAATGTTTGA 24908

man TAAATGTGAGTAATGTATCCGTCTATATTTAAATGTTTGA 24908

uni TAAATGTGAGTAATGTATCCGTCTATATTTAAATGTTTGA 24908

tall TAAATGTGAGTAATGTATCCGTCTATATTTAAATGTTTGA 24908

quil TAAATGTGAGTAATGTATCCGTCTATATTTAAATGTTTGA 24874

meri TAAATGTGAGTAATGTATCtGTCTATATTTAAATGTTTaA 25002

ref TAAATGTGAGTAgTGTATCtGTCTATATTTAAATGTTTaA 25147

Consensus taaatgtgagta tgtatc gtctatatttaaatgttt a

kal GTACTGTGTTTGTGAACTTTGAAAATAGAAACCCCAAGTG 24948

yor GTgCTGTGTTTGTGAACTTTGAAAATAGAAACCCCAAGTG 24916

jen GTACTGTGTTTGTGAACTTTGAAAATAGAAACCCCAAGTG 24948

cor GTACTGTGTTTGTGAACTTTGAAAATAGAAACCCCAAGTG 24948

man GTACTGTGTTTGTGAACTTTGAAAATAGAAACCCCAAGTG 24948

uni GTACTGTGTTTGTGAACTTTGAAAATAGAAACCCCAAGTG 24948

tall GTACTGTGTTTGTGAACTTTGAAAATAGAAACCCCAAGTG 24948

quil GTACTGTGTTTGTGAACTTTGAAAATAGAAACCCCAAGTG 24914

meri GTgCTGTGTTTGTGAACTTTGAAAATAGAAACCCCAAGTG 25042

ref GTgCTGTGTTTGTGAACTTTGAAAATAGAAACCCCAAGTG 25187

Consensus gt ctgtgtttgtgaactttgaaaatagaaaccccaagtg

kal AAAATGTAACTAGGGAAATGCATGTTTCACTGATAAAATA 24988

yor AAAATGTAAtTAaGGAAATGCATGTTTCACTcATAAAATA 24956

jen AAAATGTAACTAGGGAAATGCATGTTTCACTGATAAAATA 24988

cor AAAATGTAACTAGGGAAATGCATGTTTCACTGATAAAATA 24988

man AAAATGTAACTAGGGAAATGCATGTTTCACTGATAAAATA 24988

uni AAAATGTAACTAGGGAAATGCATGTTTCACTGATAAAATA 24988

tall AAAATGTAACTAGGGAAATGCATGTTTCACTGATAAAATA 24988

quil AAAATGTAACTAGGGAAATGCATGTTTCACTGATAAAATA 24954

meri AAAATGTAAtTAaGGAAATGCATGTTTCACTcATAAAATA 25082

ref AAAATGTAAtTAaGGAAATGCATGTTTCACTcATAAAATA 25227

Consensus aaaatgtaa ta ggaaatgcatgtttcact ataaaata

kal AAAAGTTTAGTTTTTTTTAAACTAAACATTGGATTAAGTA 25028

yor AtAAGTTTAGTTTTTTT.AAACTAAACATTGGATTAAGTA 24995

jen AAAAGTTTAGTTTTTTTTAAACTAAACATTGGATTAAGTA 25028

cor AAAAGTTTAGTTTTTTTTAAACTAAACATTGGATTAAGTA 25028

man AAAAGTTTAGTTTTTTTTAAACTAAACATTGGATTAAGTA 25028

uni AAAAGTTTAGTTTTTTTTAAACTAAACATTGGATTAAGTA 25028

tall AAAAGTTTAGTTTTTTTTAAACTAAACATTGGATTAAGTA 25028

quil AAAAGTTTAGTTTTTTTTAAACTAAACATTGGATTAAGTA 24994

meri AtAAGTTTAGTTTTTTTaAAACTAAACATTGGATTAAGTA 25122

ref AtAAGTTTAGTTTTTTTaAA.CTAAACATTGGATTAAGTA 25266

Consensus a aagtttagttttttt aa ctaaacattggattaagta

kal GGATTCAAATGCATGATAATCA.......GCTTATATCTT 25061

yor GGATTCAAATGCATGATAATCAttaatcaGCTTcTATCTT 25035

jen GGATTCAAATGCATGATAATCA.......GCTTATATCTT 25061

cor GGATTCAAATGCATGATAATCA.......GCTTATATCTT 25061

man GGATTCAAATGCATGATAATCA.......GCTTATATCTT 25061

uni GGATTCAAATGCATGATAATCA.......GCTTATATCTT 25061

tall GGATTCAAATGCATGATAATCA.......GCTTATATCTT 25061

quil GGATTCAAATGCATGATAATCA.......GCTTATATCTT 25027

meri GGATTCAAATGCATGATAATCAttaatcaGCTTcTATCTT 25162

ref GGATTCAAATGCATGATAATCAttaatcaGCTTcTATCTT 25306

Consensus ggattcaaatgcatgataatca gctt tatctt

kal TAATCATATGTTATTATTTTAATAAAATAAAAACAATAGC 25101

yor TAATCATATGTTATTATTTTAATAAAATAAAAACAATAaC 25075

jen TAATCATATGTTATTATTTTAATAAAATAAAAACAATAGC 25101

cor TAATCATATGTTATTATTTTAATAAAATAAAAACAATAGC 25101

man TAATCATATGTTATTATTTTAATAAAATAAAAACAATAGC 25101

uni TAATCATATGTTATTATTTTAATAAAATAAAAACAATAGC 25101

tall TAATCATATGTTATTATTTTAATAAAATAAAAACAATAGC 25101

quil TAATCATATGTTATTATTTTAATAAAATAAAAACAATAGC 25067

meri TAATCATATGTTATTATTTTAATAAAATAAAAACAATAaC 25202

ref TAATCATATGTTATTATTTTAATAAAATAAAAACAATAaC 25346

Consensus taatcatatgttattattttaataaaataaaaacaata c

kal TAAATAAGCAAAGAGAAAGGAGGGAAAAGTGGGGCCTAAT 25141

yor TAAATAAGCAAAGAGAAAGGAGGGAAA.GTGGGGCCTAAT 25114

jen TAAATAAGCAAAGAGAAAGGAGGGAAAAGTGGGGCCTAAT 25141

cor TAAATAAGCAAAGAGAAAGGAGGGAAAAGTGGGGCCTAAT 25141

man TAAATAAGCAAAGAGAAAGGAGGGAAAAGTGGGGCCTAAT 25141

uni TAAATAAGCAAAGAGAAAGGAGGGAAAAGTGGGGCCTAAT 25141

tall TAAATAAGCAAAGAGAAAGGAGGGAAAAGTGGGGCCTAAT 25141

quil TAAATAAGCAAAGAGAAAGGAGGGAAAAGTGGGGCCTAAT 25107

meri TAAATAAGCAAAGAGAAAGGAGGGAAA.GTGGGGCCTAAT 25241

ref TAAATAAGCAAAGAGAAAGGAGGGAAA.GTGGGGCCTAAT 25385

Consensus taaataagcaaagagaaaggagggaaa gtggggcctaat

kal GAGAAATAAATTTGCAATGGCCATAAAGCATATTGTGAAG 25181

yor GAGAAATgAATTTGCAATGGCCATAAAGCATATTGTGAAG 25154

jen GAGAAATAAATTTGCAATGGCCATAAAGCATATTGTGAAG 25181

cor GAGAAATAAATTTGCAATGGCCATAAAGCATATTGTGAAG 25181

man GAGAAATAAATTTGCAATGGCCATAAAGCATATTGTGAAG 25181

uni GAGAAATAAATTTGCAATGGCCATAAAGCATATTGTGAAG 25181

tall GAGAAATAAATTTGCAATGGCCATAAAGCATATTGTGAAG 25181

quil GAGAAATAAATTTGCAATGGCCATAAAGCATATTGTGAAG 25147

meri GAGAAATgAATTTGCAATGGCCATAAAGCATATTGTGAAG 25281

ref GAGAAATgAATTTGCAATGGCCATAAAGCATATTGTGAAG 25425

Consensus gagaaat aatttgcaatggccataaagcatattgtgaag

kal ATTATCATTGATTTGAGGAGGCAATAAAGTTGTATTGCAA 25221

yor ATTATCATTGATTTGAGGAGGCAATAAAGTTGTATTGCAA 25194

jen ATTATCATTGATTTGAGGAGGCAATAAAGTTGTATTGCAA 25221

cor ATTATCATTGATTTGAGGAGGCAATAAAGTTGTATTGCAA 25221

man ATTATCATTGATTTGAGGAGGCAATAAAGTTGTATTGCAA 25221

uni ATTATCATTGATTTGAGGAGGCAATAAAGTTGTATTGCAA 25221

tall ATTATCATTGATTTGAGGAGGCAATAAAGTTGTATTGCAA 25221

quil ATTATCATTGATTTGAGGAGGCAATAAAGTTGTATTGCAA 25187

meri ATTATCATTGATTTGAGGAGGCAATAAAGTTGTATTGCAA 25321

ref ATTATCATTGATTTGAGGAGGCAATAAAGTTGTATTGCAA 25465

Consensus attatcattgatttgaggaggcaataaagttgtattgcaa

kal ATCTCACAATACCTAATCCTAGGATTCCTTTCACATGGGC 25261

yor ATCTCACAATACCTAATCCTAGGATTCCTTTCACATGGGC 25234

jen ATCTCACAATACCTAATCCTAGGATTCCTTTCACATGGGC 25261

cor ATCTCACAATACCTAATCCTAGGATTCCTTTCACATGGGC 25261

man ATCTCACAATACCTAATCCTAGGATTCCTTTCACATGGGC 25261

uni ATCTCACAATACCTAATCCTAGGATTCCTTTCACATGGGC 25261

tall ATCTCACAATACCTAATCCTAGGATTCCTTTCACATGGGC 25261

quil ATCTCACAATACCTAATCCTAGGATTCCTTTCACATGGGC 25227

meri ATCTCACAATACCTAATCCTAGGATTCCTTTCACATGGGC 25361

ref ATCTCACAATACCTAATCCTAGGATTCCTTTCACATGGGC 25505

Consensus atctcacaatacctaatcctaggattcctttcacatgggc

kal TTATTTTAATATTGGGGCATTCATTATAAAAAACACATTT 25301

yor TTATTTTAATATTGGGGCATTtATTATAAAAAACACATTT 25274

jen TTATTTTAATATTGGGGCATTCATTATAAAAAACACATTT 25301

cor TTATTTTAATATTGGGGCATTCATTATAAAAAACACATTT 25301

man TTATTTTAATATTGGGGCATTCATTATAAAAAACACATTT 25301

uni TTATTTTAATATTGGGGCATTCATTATAAAAAACACATTT 25301

tall TTATTTTAATATTGGGGCATTCATTATAAAAAACACATTT 25301

quil TTATTTTAATATTGGGGCATTCATTATAAAAAACACATTT 25267

meri TTATTTTAATATTGGGGCATTtATTATAAAAAACACATTT 25401

ref TTATTTTAATATTGGGGCATTtATTATAAAAAACACATTT 25545

Consensus ttattttaatattggggcatt attataaaaaacacattt

kal AAATTTATTTGTTAAATAAATATATATCCAAACAAATTTT 25341

yor AAATTTATTTGTTAAATAAATATATATCCAAACAAATTTT 25314

jen AAATTTATTTGTTAAATAAATATATATCCAAACAAATTTT 25341

cor AAATTTATTTGTTAAATAAATATATATCCAAACAAATTTT 25341

man AAATTTATTTGTTAAATAAATATATATCCAAACAAATTTT 25341

uni AAATTTATTTGTTAAATAAATATATATCCAAACAAATTTT 25341

tall AAATTTATTTGTTAAATAAATATATATCCAAACAAATTTT 25341

quil AAATTTATTTGTTAAATAAATATATATCCAAACAAATTTT 25307

meri AAATTTATTTGTTAAATAAATATATATCCAAACAAATTTT 25441

ref AAATTTATTTGTTAAATAAATATATATCCAAACAAATTTT 25585

Consensus aaatttatttgttaaataaatatatatccaaacaaatttt

kal CTTGATTTTTGGAGGTGTCTATTTTTTTATTTGAGTGTAA 25381

yor CTTGATTTTTGGAGGTGTCTATTTTTTTATTTGAGTGTAA 25354

jen CTTGATTTTTGGAGGTGTCTATTTTTTTATTTGAGTGTAA 25381

cor CTTGATTTTTGGAGGTGTCTATTTTTTTATTTGAGTGTAA 25381

man CTTGATTTTTGGAGGTGTCTATTTTTTTATTTGAGTGTAA 25381

uni CTTGATTTTTGGAGGTGTCTATTTTTTTATTTGAGTGTAA 25381

tall CTTGATTTTTGGAGGTGTCTATTTTTTTATTTGAGTGTAA 25381

quil CTTGATTTTTGGAGGTGTCTATTTTTTTATTTGAGTGTAA 25347

meri CTTGATTTTTGGAGGTGTCTATTTTTTTATTTGAGTGTAA 25481

ref CTTGATTTTTGGAGGTGTCTATTTTTTTATTTGAGTGTAA 25625

Consensus cttgatttttggaggtgtctatttttttatttgagtgtaa

kal CATTTATGTACTAACAGTATAAAGATATATACACAATTAG 25421

yor CATcTATGTACTAACAGTATAAAGATATATACAtAgTcAa 25394

jen CATTTATGTACTAACAGTATAAAGATATATACACAATTAG 25421

cor CATTTATGTACTAACAGTATAAAGATATATACACAATTAG 25421

man CATTTATGTACTAACAGTATAAAGATATATACACAATTAG 25421

uni CATTTATGTACTAACAGTATAAAGATATATACACAATTAG 25421

tall CATTTATGTACTAACAGTATAAAGATATATACACAATTAG 25421

quil CATTTATGTACTAACAGTATAAAGATATATACACAATTAG 25387

meri CATcTATGTACTAACAGTATAAAGATATATACAtAgTcAa 25521

ref CATcTATGTACTAACAGTATAAAGATATATACAtAgTcAa 25665

Consensus cat tatgtactaacagtataaagatatataca a t a

kal TGCGTAATCTACAATTAATTTGTATTACTTTTATTTAAGA 25461

yor TGCGTAATCTAtAATTAATTTGTATTACTTTTATTTAAGA 25434

jen TGCGTAATCTACAATTAATTTGTATTACTTTTATTTAAGA 25461

cor TGCGTAATCTACAATTAATTTGTATTACTTTTATTTAAGA 25461

man TGCGTAATCTACAATTAATTTGTATTACTTTTATTTAAGA 25461

uni TGCGTAATCTACAATTAATTTGTATTACTTTTATTTAAGA 25461

tall TGCGTAATCTACAATTAATTTGTATTACTTTTATTTAAGA 25461

quil TGCGTAATCTACAATTAATTTGTATTACTTTTATTTAAGA 25427

meri TGCGTAATCTAtAATTAATTTGTATTACTTTTATTTAAGA 25561

ref TGCGTAATCTAtAATTAATTTGTATTACTTTTATTTAAGA 25705

Consensus tgcgtaatcta aattaatttgtattacttttatttaaga

kal TTCAACGATAATTTCTATTTAACTAGAACATACTACT... 25498

yor gTtAAtGATAATTTCTAcTTAACTAaAACATACTACTcca 25474

jen TTCAACGATAATTTCTATTTAACTAGAACATACTACT... 25498

cor TTCAACGATAATTTCTATTTAACTAGAACATACTACT... 25498

man TTCAACGATAATTTCTATTTAACTAGAACATACTACT... 25498

uni TTCAACGATAATTTCTATTTAACTAGAACATACTACT... 25498

tall TTCAACGATAATTTCTATTTAACTAGAACATACTACT... 25498

quil TTCAACGATAATTTCTATTTAACTAGAACATACTACT... 25464

meri gTtAAtGATAATTTCTAcTTAACTAaAACATACTACTcca 25601

ref gTtAAtGATAATTTCTAcTTAACTAaAACATACTACTcca 25745

Consensus t aa gataatttcta ttaacta aacatactact

kal .................ATGAATTTAGCCTCTCTAATCAT 25521

yor cactctttctagcatgcATGAATTTAGCCTCTCTAATCAT 25514

jen .................ATGAATTTAGCCTCTCTAATCAT 25521

cor .................ATGAATTTAGCCTCTCTAATCAT 25521

man .................ATGAATTTAGCCTCTCTAATCAT 25521

uni .................ATGAATTTAGCCTCTCTAATCAT 25521

tall .................ATGAATTTAGCCTCTCTAATCAT 25521

quil .................ATGAATTTAGCCTCTCTAATCAT 25487

meri cactctttctagcatgcATGAATTTAGCCTCTCTAATCAT 25641

ref cactctttctagcatgcATGAATTTAGCCTCTCTAATCAT 25785

Consensus atgaatttagcctctctaatcat

kal GAAAGGGAAATTATAACAAGTATGCATAGAAATGGATTTG 25561

yor GAAAaGGAAATTATAACAAGTATGCATAGAAATGGATTTG 25554

jen GAAAGGGAAATTATAACAAGTATGCATAGAAATGGATTTG 25561

cor GAAAGGGAAATTATAACAAGTATGCATAGAAATGGATTTG 25561

man GAAAGGGAAATTATAACAAGTATGCATAGAAATGGATTTG 25561

uni GAAAGGGAAATTATAACAAGTATGCATAGAAATGGATTTG 25561

tall GAAAGGGAAATTATAACAAGTATGCATAGAAATGGATTTG 25561

quil GAAAGGGAAATTATAACAAGTATGCATAGAAATGGATTTG 25527

meri GAAAaGGAAATTATAACAAGTATGCATAGAAATGGATTTG 25681

ref GAAAaGGAAATTATAACAAGTATGCATAGAAATGGATTTG 25825

Consensus gaaa ggaaattataacaagtatgcatagaaatggatttg

kal AGGTATTATCAGAAAGATAATTTGTAATACTCACTTAAAG 25601

yor AGGTATTATCAGAAAGATAATTTGTAATACTCACTTAAAG 25594

jen AGGTATTATCAGAAAGATAATTTGTAATACTCACTTAAAG 25601

cor AGGTATTATCAGAAAGATAATTTGTAATACTCACTTAAAG 25601

man AGGTATTATCAGAAAGATAATTTGTAATACTCACTTAAAG 25601

uni AGGTATTATCAGAAAGATAATTTGTAATACTCACTTAAAG 25601

tall AGGTATTATCAGAAAGATAATTTGTAATACTCACTTAAAG 25601

quil AGGTATTATCAGAAAGATAATTTGTAATACTCACTTAAAG 25567

meri AGGTATTATCAGAAAGATAATTTGTAATACTCACTTAAAG 25721

ref AGGTATTATCAGAAAGATAATTTGTAATACTCACTTAAAG 25865

Consensus aggtattatcagaaagataatttgtaatactcacttaaag

kal TCATAGTTTGGGTATATCATATAACTTCAATGGAACCATG 25641

yor TCATAGTTTGGGTATAcCATATAACTTCAATGGAACCATG 25634

jen TCATAGTTTGGGTATATCATATAACTTCAATGGAACCATG 25641

cor TCATAGTTTGGGTATATCATATAACTTCAATGGAACCATG 25641

man TCATAGTTTGGGTATATCATATAACTTCAATGGAACCATG 25641

uni TCATAGTTTGGGTATATCATATAACTTCAATGGAACCATG 25641

tall TCATAGTTTGGGTATATCATATAACTTCAATGGAACCATG 25641

quil TCATAGTTTGGGTATATCATATAACTTCAATGGAACCATG 25607

meri TCATAGTTTGGGTATAcCATATAACTTCAATGGAACCATG 25761

ref TCATAGTTTGGGTATAcCATATAACTTCAATGGAACCATG 25905

Consensus tcatagtttgggtata catataacttcaatggaaccatg

kal CTGATTGCATCACCAAGTTACAATGTCAATTATAAGGTGC 25681

yor CTGATTGCATCACCAAGTTACAATGTCAATTATAAGGTGC 25674

jen CTGATTGCATCACCAAGTTACAATGTCAATTATAAGGTGC 25681

cor CTGATTGCATCACCAAGTTACAATGTCAATTATAAGGTGC 25681

man CTGATTGCATCACCAAGTTACAATGTCAATTATAAGGTGC 25681

uni CTGATTGCATCACCAAGTTACAATGTCAATTATAAGGTGC 25681

tall CTGATTGCATCACCAAGTTACAATGTCAATTATAAGGTGC 25681

quil CTGATTGCATCACCAAGTTACAATGTCAATTATAAGGTGC 25647

meri CTGATTGCATCACCAAGTTACAATGTCAATTATAAGGTGC 25801

ref CTGATTGCATCACCAAGTTACAATGTCAATTATAAGGTGC 25945

Consensus ctgattgcatcaccaagttacaatgtcaattataaggtgc

kal TTAGATATATTTTAAGCTTAACTAATATATGAAGCTTCTA 25721

yor TTAGATgTATTTTAAGCTTAACTAATATATGAAGCTTCTA 25714

jen TTAGATATATTTTAAGCTTAACTAATATATGAAGCTTCTA 25721

cor TTAGATATATTTTAAGCTTAACTAATATATGAAGCTTCTA 25721

man TTAGATATATTTTAAGCTTAACTAATATATGAAGCTTCTA 25721

uni TTAGATATATTTTAAGCTTAACTAATATATGAAGCTTCTA 25721

tall TTAGATATATTTTAAGCTTAACTAATATATGAAGCTTCTA 25721

quil TTAGATATATTTTAAGCTTAACTAATATATGAAGCTTCTA 25687

meri TTAGATgTATTTTAAGCTTAACTAATATATGAAGCTTCTA 25841

ref TTAGATgTATTTTAAGCTTAACTAATATATGAAGCTTCTA 25985

Consensus ttagat tattttaagcttaactaatatatgaagcttcta

kal ATATCAATAAATGTTGACCCAATTCTTGAGAAATTGATCA 25761

yor ATATCAATAAATGTTGACCCAATTCTTaAGAAATTGATCA 25754

jen ATATCAATAAATGTTGACCCAATTCTTGAGAAATTGATCA 25761

cor ATATCAATAAATGTTGACCCAATTCTTGAGAAATTGATCA 25761

man ATATCAATAAATGTTGACCCAATTCTTGAGAAATTGATCA 25761

uni ATATCAATAAATGTTGACCCAATTCTTGAGAAATTGATCA 25761

tall ATATCAATAAATGTTGACCCAATTCTTGAGAAATTGATCA 25761

quil ATATCAATAAATGTTGACCCAATTCTTGAGAAATTGATCA 25727

meri ATATCAATAAATGTTGACCCAATTCTTaAGAAATTGATCA 25881

ref ATATCAATAAATGTTGACCCAATTCTTaAGAAATTGATCA 26025

Consensus atatcaataaatgttgacccaattctt agaaattgatca

kal ATAACACATAAGTTAAGGTTATAAATTTAATCGTGTTATT 25801

yor ATAACACATAAGTTAAGGTTATAAATTTAATtGTGTTgTc 25794

jen ATAACACATAAGTTAAGGTTATAAATTTAATCGTGTTATT 25801

cor ATAACACATAAGTTAAGGTTATAAATTTAATCGTGTTATT 25801

man ATAACACATAAGTTAAGGTTATAAATTTAATCGTGTTATT 25801

uni ATAACACATAAGTTAAGGTTATAAATTTAATCGTGTTATT 25801

tall ATAACACATAAGTTAAGGTTATAAATTTAATCGTGTTATT 25801

quil ATAACACATAAGTTAAGGTTATAAATTTAATCGTGTTATT 25767

meri ATAACACATAAGTTAAGGTTATAAATTTAATtGTGTTgTc 25921

ref ATAACACATAAGTTAAGGTTATAAATTTAATtGTGTTgTc 26065

Consensus ataacacataagttaaggttataaatttaat gtgtt t

kal AGCATATGAAACTCGTTGACGAATAATCTATAAATATATT 25841

yor AGCATATGgAACTCGTTGACGAATAATCTATAAATATATT 25834

jen AGCATATGAAACTCGTTGACGAATAATCTATAAATATATT 25841

cor AGCATATGAAACTCGTTGACGAATAATCTATAAATATATT 25841

man AGCATATGAAACTCGTTGACGAATAATCTATAAATATATT 25841

uni AGCATATGAAACTCGTTGACGAATAATCTATAAATATATT 25841

tall AGCATATGAAACTCGTTGACGAATAATCTATAAATATATT 25841

quil AGCATATGAAACTCGTTGACGAATAATCTATAAATATATT 25807

meri AGCATATGgAACTCGTTGACGAATAATCTATAAATATATT 25961

ref AGCATATGgAACTCGTTGACGAATAATCTATAAATATATT 26105

Consensus agcatatg aactcgttgacgaataatctataaatatatt

kal TGTAAGTGCTCATAAATAAATGAACCTAACCATATCCATA 25881

yor TGTAAGTGCTCgTAgATAAATGAACCTtACCATATtCATA 25874

jen TGTAAGTGCTCATAAATAAATGAACCTAACCATATCCATA 25881

cor TGTAAGTGCTCATAAATAAATGAACCTAACCATATCCATA 25881

man TGTAAGTGCTCATAAATAAATGAACCTAACCATATCCATA 25881

uni TGTAAGTGCTCATAAATAAATGAACCTAACCATATCCATA 25881

tall TGTAAGTGCTCATAAATAAATGAACCTAACCATATCCATA 25881

quil TGTAAGTGCTCATAAATAAATGAACCTAACCATATCCATA 25847

meri TGTAAGTGCTCgTAgATAAATGAACCTtACCATATtCATA 26001

ref TGTAAGTGCTCgTAgATAAATGAACCTtACCATATtCATA 26145

Consensus tgtaagtgctc ta ataaatgaacct accatat cata

kal TCGAGTTTATATAATAAAAAATGTCAACTATGAGGTATAT 25921

yor TtGAGTTTATAcAtT...............TGAGGTATAc 25899

jen TCGAGTTTATATAATAAAAAATGTCAACTATGAGGTATAT 25921

cor TCGAGTTTATATAATAAAAAATGTCAACTATGAGGTATAT 25921

man TCGAGTTTATATAATAAAAAATGTCAACTATGAGGTATAT 25921

uni TCGAGTTTATATAATAAAAAATGTCAACTATGAGGTATAT 25921

tall TCGAGTTTATATAATAAAAAATGTCAACTATGAGGTATAT 25921

quil TCGAGTTTATATAATAAAAAATGTCAACTATGAGGTATAT 25887

meri TtGAGTTTATAcAtT...............TGAGGTATAc 26026

ref TtGAGTTTATAcAtT...............TGAGGTATAc 26170

Consensus t gagtttata a t tgaggtata

kal GGATGTATTTCCTTACTATAATTAAACTTTATTTTCTTGA 25961

yor GGATGTATTTCCTTACTAcAATTAAACTT.ATTTTgTTGA 25938

jen GGATGTATTTCCTTACTATAATTAAACTTTATTTTCTTGA 25961

cor GGATGTATTTCCTTACTATAATTAAACTTTATTTTCTTGA 25961

man GGATGTATTTCCTTACTATAATTAAACTTTATTTTCTTGA 25961

uni GGATGTATTTCCTTACTATAATTAAACTTTATTTTCTTGA 25961

tall GGATGTATTTCCTTACTATAATTAAACTTTATTTTCTTGA 25961

quil GGATGTATTTCCTTACTATAATTAAACTTTATTTTCTTGA 25927

meri GGATGTATTTCCTTACTAcAATTAAACTT.ATTTTgTTGA 26065

ref GGATGTATTTCCTTACTAcAATTAAACTT.ATTTTgTTGA 26209

Consensus ggatgtatttccttacta aattaaactt atttt ttga

kal TAGGTTAGTGCATGATTGAGCCATCAAACTACCTAATTAG 26001

yor TAGGTTAGTGCATGATTGAGCCATCAAACTACCTAAgTAG 25978

jen TAGGTTAGTGCATGATTGAGCCATCAAACTACCTAATTAG 26001

cor TAGGTTAGTGCATGATTGAGCCATCAAACTACCTAATTAG 26001

man TAGGTTAGTGCATGATTGAGCCATCAAACTACCTAATTAG 26001

uni TAGGTTAGTGCATGATTGAGCCATCAAACTACCTAATTAG 26001

tall TAGGTTAGTGCATGATTGAGCCATCAAACTACCTAATTAG 26001

quil TAGGTTAGTGCATGATTGAGCCATCAAACTACCTAATTAG 25967

meri TAGGTTAGTGCATGATTGAGCCATCAAACTACCTAATTAG 26105

ref TAGGTTAGTGCATGATTGAGCCATCAAACTACCTAATTAG 26249

Consensus taggttagtgcatgattgagccatcaaactacctaa tag

kal TCTTCCCTATAGCTCTTGATTTCCACCCTCTCATTAAGCT 26041

yor TCTTCCCTATAGCTCTTGATTTCCACCCTCTCATTAAGCT 26018

jen TCTTCCCTATAGCTCTTGATTTCCACCCTCTCATTAAGCT 26041

cor TCTTCCCTATAGCTCTTGATTTCCACCCTCTCATTAAGCT 26041

man TCTTCCCTATAGCTCTTGATTTCCACCCTCTCATTAAGCT 26041

uni TCTTCCCTATAGCTCTTGATTTCCACCCTCTCATTAAGCT 26041

tall TCTTCCCTATAGCTCTTGATTTCCACCCTCTCATTAAGCT 26041

quil TCTTCCCTATAGCTCTTGATTTCCACCCTCTCATTAAGCT 26007

meri TCTTCCCTATAGCTCTTGATTTCCACCCTCTCATTAAGCT 26145

ref TCTTCCCTATAGCTCTTGATTTCCACCCTCTCATTAAGCT 26289

Consensus tcttccctatagctcttgatttccaccctctcattaagct

kal TAAGAGCAAAGGATATGATTATCTTTATTCTTGAAGAAGA 26081

yor TA.GAGCAAAGGATATGATTATCTTTATTCTTGAAGAAGA 26057

jen TAAGAGCAAAGGATATGATTATCTTTATTCTTGAAGAAGA 26081

cor TAAGAGCAAAGGATATGATTATCTTTATTCTTGAAGAAGA 26081

man TAAGAGCAAAGGATATGATTATCTTTATTCTTGAAGAAGA 26081

uni TAAGAGCAAAGGATATGATTATCTTTATTCTTGAAGAAGA 26081

tall TAAGAGCAAAGGATATGATTATCTTTATTCTTGAAGAAGA 26081

quil TAAGAGCAAAGGATATGATTATCTTTATTCTTGAAGAAGA 26047

meri TA.GAGCAAAGGATATGATTATCTTTATTCTTGAAGAAGA 26184

ref TA.GAGCAAAGGATATGATTATCTTTATTCTTGAAGAAGA 26328

Consensus ta gagcaaaggatatgattatctttattcttgaagaaga

kal CAACAAAATATACATAACTCTCATAGTTTATTGGACTCTA 26121

yor CAACAAAATATACATAACTCTCATAGTTTATTGGACTCTA 26097

jen CAACAAAATATACATAACTCTCATAGTTTATTGGACTCTA 26121

cor CAACAAAATATACATAACTCTCATAGTTTATTGGACTCTA 26121

man CAACAAAATATACATAACTCTCATAGTTTATTGGACTCTA 26121

uni CAACAAAATATACATAACTCTCATAGTTTATTGGACTCTA 26121

tall CAACAAAATATACATAACTCTCATAGTTTATTGGACTCTA 26121

quil CAACAAAATATACATAACTCTCATAGTTTATTGGACTCTA 26087

meri CAACAAAATATACATAACTCTCATAGTTTATTGGACTCTA 26224

ref CAACAAAATATACATAACTCTCATAGTTTATTGGACTCTA 26368

Consensus caacaaaatatacataactctcatagtttattggactcta

kal TGCACTATAGGGAAATCCAAATCCGTTAAATGGGGTGAAT 26161

yor TGCACTATAGGGAAATCCAAATCCGTTAAATGGGGTGAAT 26137

jen TGCACTATAGGGAAATCCAAATCCGTTAAATGGGGTGAAT 26161

cor TGCACTATAGGGAAATCCAAATCCGTTAAATGGGGTGAAT 26161

man TGCACTATAGGGAAATCCAAATCCGTTAAATGGGGTGAAT 26161

uni TGCACTATAGGGAAATCCAAATCCGTTAAATGGGGTGAAT 26161

tall TGCACTATAGGGAAATCCAAATCCGTTAAATGGGGTGAAT 26161

quil TGCACTATAGGGAAATCCAAATCCGTTAAATGGGGTGAAT 26127

meri TGCACTATAGGGAAATCCAAATCCGTTAAATGGGGTGAAT 26264

ref TGCACTATAGGGAAATCCAAATCCGTTAAATGGGGTGAAT 26408

Consensus tgcactatagggaaatccaaatccgttaaatggggtgaat

kal AAAAGTGGCATTATTTTAAGTTATACCTTCTCTATCTATG 26201

yor AAAAGTGGCATTATTTTAAGTTATACCTTCTCTATCTATG 26177

jen AAAAGTGGCATTATTTTAAGTTATACCTTCTCTATCTATG 26201

cor AAAAGTGGCATTATTTTAAGTTATACCTTCTCTATCTATG 26201

man AAAAGTGGCATTATTTTAAGTTATACCTTCTCTATCTATG 26201

uni AAAAGTGGCATTATTTTAAGTTATACCTTCTCTATCTATG 26201

tall AAAAGTGGCATTATTTTAAGTTATACCTTCTCTATCTATG 26201

quil AAAAGTGGCATTATTTTAAGTTATACCTTCTCTATCTATG 26167

meri AAAAGTGGCATTATTTTAAGTTATACCTTCTCTATCTATG 26304

ref AAAAGTGGCATTATTTTAAGTTATACCTTCTCTATCTATG 26448

Consensus aaaagtggcattattttaagttataccttctctatctatg

kal TGTGATTTTTTAAAATCATATTTAATACATCATCACATTA 26241

yor TGTGATTTTTTAAAATCATATTTAATAtATCATCACAT.. 26215

jen TGTGATTTTTTAAAATCATATTTAATACATCATCACATTA 26241

cor TGTGATTTTTTAAAATCATATTTAATACATCATCACATTA 26241

man TGTGATTTTTTAAAATCATATTTAATACATCATCACATTA 26241

uni TGTGATTTTTTAAAATCATATTTAATACATCATCACATTA 26241

tall TGTGATTTTTTAAAATCATATTTAATACATCATCACATTA 26241

quil TGTGATTTTTTAAAATCATATTTAATACATCATCACATTA 26207

meri TGTGATTTTTTAAAATCATATTTAATAtATCATCACATTA 26344

ref TGTGATTTTTTAAAATCATATTTAATAtATCATCACATTA 26488

Consensus tgtgattttttaaaatcatatttaata atcatcacat

kal ACTAGGTAAAACATTTACTACTACTAATGGTATTTTCTCA 26281

yor .......AAAACATTTACTACTACTAATGaTATcTTtTCA 26248

jen ACTAGGTAAAACATTTACTACTACTAATGGTATTTTCTCA 26281

cor ACTAGGTAAAACATTTACTACTACTAATGGTATTTTCTCA 26281

man ACTAGGTAAAACATTTACTACTACTAATGGTATTTTCTCA 26281

uni ACTAGGTAAAACATTTACTACTACTAATGGTATTTTCTCA 26281

tall ACTAGGTAAAACATTTACTACTACTAATGGTATTTTCTCA 26281

quil ACTAGGTAAAACATTTACTACTACTAATGGTATTTTCTCA 26247

meri ACTAcGTAAAACATTTACTACTACTAATGGTATTTTCTCA 26384

ref ACTAcGTAAAACATTTACTACTACTAATGGTATTTTCTCA 26528

Consensus aaaacatttactactactaatg tat tt tca

kal TCTTTTTGGATAATGAACTAAAAAAATTTGAGGTTCGTGA 26321

yor TCTTTTTGGATAATGAACTAAtt..tTTTtgGGTTCGTGA 26286

jen TCTTTTTGGATAATGAACTAAAAAAATTTGAGGTTCGTGA 26321

cor TCTTTTTGGATAATGAACTAAAAAAATTTGAGGTTCGTGA 26321

man TCTTTTTGGATAATGAACTAAAAAAATTTGAGGTTCGTGA 26321

uni TCTTTTTGGATAATGAACTAAAAAAATTTGAGGTTCGTGA 26321

tall TCTTTTTGGATAATGAACTAAAAAAATTTGAGGTTCGTGA 26321

quil TCTTTTTGGATAATGAACTAAAAAAATTTGAGGTTCGTGA 26287

meri TCTTTTTGGATAATGAACTAAAAAAtTTTGAGGTTCGTGA 26424

ref TCTTTTTGGATAATGAACTAAAAAAtTTTGAGGTTCGTGA 26568

Consensus tctttttggataatgaactaa ttt ggttcgtga

kal CTATTTCTCTCAACAAGTGTCACTCTCAGAAATCGAACCC 26361

yor CTgcccCTCTCAACAAGTaTCACTCTCAGgAATtGAACtC 26326

jen CTATTTCTCTCAACAAGTGTCACTCTCAGAAATCGAACCC 26361

cor CTATTTCTCTCAACAAGTGTCACTCTCAGAAATCGAACCC 26361

man CTATTTCTCTCAACAAGTGTCACTCTCAGAAATCGAACCC 26361

uni CTATTTCTCTCAACAAGTGTCACTCTCAGAAATCGAACCC 26361

tall CTATTTCTCTCAACAAGTGTCACTCTCAGAAATCGAACCC 26361

quil CTATTTCTCTCAACAAGTGTCACTCTCAGAAATCGAACCC 26327

meri CTAccTCTtTCAACAAGTGTaACTCTCAGAAATCGAACtC 26464

ref CTAccTCTtTCAACAAGTGTaACTCTCAGAAATCGAACtC 26608

Consensus ct ct tcaacaagt t actctcag aat gaac c

kal TCGACCTTGAAGATATAATAAAGTTTTACACCACTAAACT 26401

yor TCGACCTTaAAGATtTAATgAAGTTTTACACCACTAAACT 26366

jen TCGACCTTGAAGATATAATAAAGTTTTACACCACTAAACT 26401

cor TCGACCTTGAAGATATAATAAAGTTTTACACCACTAAACT 26401

man TCGACCTTGAAGATATAATAAAGTTTTACACCACTAAACT 26401

uni TCGACCTTGAAGATATAATAAAGTTTTACACCACTAAACT 26401

tall TCGACCTTGAAGATATAATAAAGTTTTACACCACTAAACT 26401

quil TCGACCTTGAAGATATAATAAAGTTTTACACCACTAAACT 26367

meri TCGACCTTagAGATATAATAAAGTTTTACACCACTAAACT 26504

ref TCGACCTTagAGATATAATAAAGTTTTACACCACTAAACT 26648

Consensus tcgacctt agat taat aagttttacaccactaaact

kal AACGTT.TGTTAGTTGCTACTC.....CAGAAAAACCACA 26435

yor AACGcTaTGTTAGTTaCTACTCatgttCAGAAAAACCACA 26406

jen AACGTT.TGTTAGTTGCTACTC.....CAGAAAAACCACA 26435

cor AACGTT.TGTTAGTTGCTACTC.....CAGAAAAACCACA 26435

man AACGTT.TGTTAGTTGCTACTC.....CAGAAAAACCACA 26435

uni AACGTT.TGTTAGTTGCTACTC.....CAGAAAAACCACA 26435

tall AACGTT.TGTTAGTTGCTACTC.....CAGAAAAACCACA 26435

quil AACGTT.TGTTAGTTGCTACTC.....CAGAAAAACCACA 26401

meri AACGcTtTaTTAGTTaCTACTCatgttCAGAAAAACCACA 26544

ref AACGcTtTaTTAGTTaCTACTCatgttCAGAAAAACCACA 26688

Consensus aacg t t ttagtt ctactc cagaaaaaccaca

kal ATATAATTAGTGTTGAAAAGCATACTTTTAGAAAGCATAT 26475

yor ATATAATTAGTGTTaAAAAGCATACTTTTAGAAAGCATAT 26446

jen ATATAATTAGTGTTGAAAAGCATACTTTTAGAAAGCATAT 26475

cor ATATAATTAGTGTTGAAAAGCATACTTTTAGAAAGCATAT 26475

man ATATAATTAGTGTTGAAAAGCATACTTTTAGAAAGCATAT 26475

uni ATATAATTAGTGTTGAAAAGCATACTTTTAGAAAGCATAT 26475

tall ATATAATTAGTGTTGAAAAGCATACTTTTAGAAAGCATAT 26475

quil ATATAATTAGTGTTGAAAAGCATACTTTTAGAAAGCATAT 26441

meri ATATAATTAGTGTTGAAAAGCATACTTTTAGAAAGCATAT 26584

ref ATATAATTAGTGTTGAAAAGCATACTTTTAGAAAGCATAT 26728

Consensus atataattagtgtt aaaagcatacttttagaaagcatat

kal TTTGTCCTTTAATTGTTCCATATGTGTTATCATTGCTTCC 26515

yor TTTGTCCTTTAATTGTTCCATATGTGTTATCATTGCTTCC 26486

jen TTTGTCCTTTAATTGTTCCATATGTGTTATCATTGCTTCC 26515

cor TTTGTCCTTTAATTGTTCCATATGTGTTATCATTGCTTCC 26515

man TTTGTCCTTTAATTGTTCCATATGTGTTATCATTGCTTCC 26515

uni TTTGTCCTTTAATTGTTCCATATGTGTTATCATTGCTTCC 26515

tall TTTGTCCTTTAATTGTTCCATATGTGTTATCATTGCTTCC 26515

quil TTTGTCCTTTAATTGTTCCATATGTGTTATCATTGCTTCC 26481

meri TTTGTCCTTTAATTGTTCCATATGTGTTATCATTGCTTCC 26624

ref TTTGTCCTTTAATTGTTCCATATGTGTTATCATTGCTTCC 26768

Consensus tttgtcctttaattgttccatatgtgttatcattgcttcc

kal ATTTATTGTAACACAAGATACTAGTATATACTTTATGGTA 26555

yor ATTTATTGTAACACAAGATACTAGTATATACTTTATGGTA 26526

jen ATTTATTGTAACACAAGATACTAGTATATACTTTATGGTA 26555

cor ATTTATTGTAACACAAGATACTAGTATATACTTTATGGTA 26555

man ATTTATTGTAACACAAGATACTAGTATATACTTTATGGTA 26555

uni ATTTATTGTAACACAAGATACTAGTATATACTTTATGGTA 26555

tall ATTTATTGTAACACAAGATACTAGTATATACTTTATGGTA 26555

quil ATTTATTGTAACACAAGATACTAGTATATACTTTATGGTA 26521

meri ATTTATTGTAACACAAGATACTAGTATATACTTTATGGTA 26664

ref ATTTATTGTAACACAAGATACTAGTATATACTTTATGGTA 26808

Consensus atttattgtaacacaagatactagtatatactttatggta

kal GGTGTTGTATTATAATATGAATGAATTACACAAAAGTTTT 26595

yor GGTGTTGTATTATAATATGAATGAATTACACAAAAGTTTT 26566

jen GGTGTTGTATTATAATATGAATGAATTACACAAAAGTTTT 26595

cor GGTGTTGTATTATAATATGAATGAATTACACAAAAGTTTT 26595

man GGTGTTGTATTATAATATGAATGAATTACACAAAAGTTTT 26595

uni GGTGTTGTATTATAATATGAATGAATTACACAAAAGTTTT 26595

tall GGTGTTGTATTATAATATGAATGAATTACACAAAAGTTTT 26595

quil GGTGTTGTATTATAATATGAATGAATTACACAAAAGTTTT 26561

meri GGTGTTGTATTATAATATGAATGAATTACACAAAAGTTTT 26704

ref GGTGTTGTATTATAATATGAATGAATTACACAAAAGTTTT 26848

Consensus ggtgttgtattataatatgaatgaattacacaaaagtttt

kal CTACAAAGTCAATTACAATGAGTATGGTTGTGGACCATTT 26635

yor CTACAAAGTCAATTACAATGAGTATGGTTGTGGACCATTT 26606

jen CTACAAAGTCAATTACAATGAGTATGGTTGTGGACCATTT 26635

cor CTACAAAGTCAATTACAATGAGTATGGTTGTGGACCATTT 26635

man CTACAAAGTCAATTACAATGAGTATGGTTGTGGACCATTT 26635

uni CTACAAAGTCAATTACAATGAGTATGGTTGTGGACCATTT 26635

tall CTACAAAGTCAATTACAATGAGTATGGTTGTGGACCATTT 26635

quil CTACAAAGTCAATTACAATGAGTATGGTTGTGGACCATTT 26601

meri CTACAAAGTCAATTACAATGAGTATGGTTGTGGACCATTT 26744

ref CTACAAAGTCAATTACAATGAGTATGGTTGTGGACCATTT 26888

Consensus ctacaaagtcaattacaatgagtatggttgtggaccattt

kal GCCTTCAAAGACAGCGTAGAAAGGGGAATGTGTTTTTGTT 26675

yor GCCTTCAAAGACAGCGTAGAAAGGGGAATGTGTTTTTGTT 26646

jen GCCTTCAAAGACAGCGTAGAAAGGGGAATGTGTTTTTGTT 26675

cor GCCTTCAAAGACAGCGTAGAAAGGGGAATGTGTTTTTGTT 26675

man GCCTTCAAAGACAGCGTAGAAAGGGGAATGTGTTTTTGTT 26675

uni GCCTTCAAAGACAGCGTAGAAAGGGGAATGTGTTTTTGTT 26675

tall GCCTTCAAAGACAGCGTAGAAAGGGGAATGTGTTTTTGTT 26675

quil GCCTTCAAAGACAGCGTAGAAAGGGGAATGTGTTTTTGTT 26641

meri GCCTTCAAAGACAGCGTAGAAAGGGGAATGTGTTTTTGTT 26784

ref GCCTTCAAAGACAGCGTAGAAAGGGGAATGTGTTTTTGTT 26928

Consensus gccttcaaagacagcgtagaaaggggaatgtgtttttgtt

kal TTCGGATGTATAATTTTAATAACTTCTTATATCACAAATC 26715

yor TTCGGATGTATAATTTTAATAACTTCTTATATCACAAATC 26686

jen TTCGGATGTATAATTTTAATAACTTCTTATATCACAAATC 26715

cor TTCGGATGTATAATTTTAATAACTTCTTATATCACAAATC 26715

man TTCGGATGTATAATTTTAATAACTTCTTATATCACAAATC 26715

uni TTCGGATGTATAATTTTAATAACTTCTTATATCACAAATC 26715

tall TTCGGATGTATAATTTTAATAACTTCTTATATCACAAATC 26715

quil TTCGGATGTATAATTTTAATAACTTCTTATATCACAAATC 26681

meri TTCGGATGTATAATTTTAATAACTTCTTATATCACAAATC 26824

ref TTCGGATGTATAATTTTAATAACTTCTTATATCACAAATC 26968

Consensus ttcggatgtataattttaataacttcttatatcacaaatc

kal AAAGTATTCTTATATTTCTATTATTTTAATTTTTTATCCA 26755

yor AAAGTATTCTTATATTTCTATTATTTTAATTTTTTATCCA 26726

jen AAAGTATTCTTATATTTCTATTATTTTAATTTTTTATCCA 26755

cor AAAGTATTCTTATATTTCTATTATTTTAATTTTTTATCCA 26755

man AAAGTATTCTTATATTTCTATTATTTTAATTTTTTATCCA 26755

uni AAAGTATTCTTATATTTCTATTATTTTAATTTTTTATCCA 26755

tall AAAGTATTCTTATATTTCTATTATTTTAATTTTTTATCCA 26755

quil AAAGTATTCTTATATTTCTATTATTTTAATTTTTTATCCA 26721

meri AAAGTATTaTTATATTTCTATTATTTTAATTTTTTATCCA 26864

ref AAAGTATTaTTATATTTCTATTATTTTAATTTTTTATCCA 27008

Consensus aaagtatt ttatatttctattattttaattttttatcca

kal TAAACAAATATCAAATAATTCATCTAGGAATCACTTTGCT 26795

yor TAAACAAATATCAAATAATTCATCTAGGAATCACTTTGCT 26766

jen TAAACAAATATCAAATAATTCATCTAGGAATCACTTTGCT 26795

cor TAAACAAATATCAAATAATTCATCTAGGAATCACTTTGCT 26795

man TAAACAAATATCAAATAATTCATCTAGGAATCACTTTGCT 26795

uni TAAACAAATATCAAATAATTCATCTAGGAATCACTTTGCT 26795

tall TAAACAAATATCAAATAATTCATCTAGGAATCACTTTGCT 26795

quil TAAACAAATATCAAATAATTCATCTAGGAATCACTTTGCT 26761

meri TAAACAAATATCAAATAATTCATCTAGGAATCACTTTGCT 26904

ref TAAACAAATATCAAATAATTCATCTAGGAATCACTTTGCT 27048

Consensus taaacaaatatcaaataattcatctaggaatcactttgct

kal TCTAAGTTATGATAACATCATAACATACTCTCAAAACTAA 26835

yor TCTAAGTTATGATAACATCATAACATACTCTCAAAACTAA 26806

jen TCTAAGTTATGATAACATCATAACATACTCTCAAAACTAA 26835

cor TCTAAGTTATGATAACATCATAACATACTCTCAAAACTAA 26835

man TCTAAGTTATGATAACATCATAACATACTCTCAAAACTAA 26835

uni TCTAAGTTATGATAACATCATAACATACTCTCAAAACTAA 26835

tall TCTAAGTTATGATAACATCATAACATACTCTCAAAACTAA 26835

quil TCTAAGTTATGATAACATCATAACATACTCTCAAAACTAA 26801

meri TgTAAGgTATGATAACATCATAACATACTCTCAAAACTAA 26944

ref TgTAAGgTATGATAACATCATAACATACTCTCAAAACTAA 27088

Consensus t taag tatgataacatcataacatactctcaaaactaa

kal TGTTGAAATTTCAGCACTTGAAAAAAAAATGTCACTCTTC 26875

yor TGTTGAAATTTCAGCACTTGAAAAAAAAATGTCACTCTTC 26846

jen TGTTGAAATTTCAGCACTTGAAAAAAAAATGTCACTCTTC 26875

cor TGTTGAAATTTCAGCACTTGAAAAAAAAATGTCACTCTTC 26875

man TGTTGAAATTTCAGCACTTGAAAAAAAAATGTCACTCTTC 26875

uni TGTTGAAATTTCAGCACTTGAAAAAAAAATGTCACTCTTC 26875

tall TGTTGAAATTTCAGCACTTGAAAAAAAAATGTCACTCTTC 26875

quil TGTTGAAATTTCAGCACTTGAAAAAAAAATGTCACTCTTC 26841

meri TGTTGAAATTTCAGCACTTGAAAAAAAA.TGTCACTCTTC 26983

ref TGTTGAAATTTCAGCACTTGAAAAAAAA.TGTCACTCTTC 27127

Consensus tgttgaaatttcagcacttgaaaaaaaa tgtcactcttc

kal TAACTTTATTTAAAAAGAAATCATCAATCCAATTCTTATA 26915

yor TAACTTTATTTAAAAAGAAATCATCAATCCAATTCTTATA 26886

jen TAACTTTATTTAAAAAGAAATCATCAATCCAATTCTTATA 26915

cor TAACTTTATTTAAAAAGAAATCATCAATCCAATTCTTATA 26915

man TAACTTTATTTAAAAAGAAATCATCAATCCAATTCTTATA 26915

uni TAACTTTATTTAAAAAGAAATCATCAATCCAATTCTTATA 26915

tall TAACTTTATTTAAAAAGAAATCATCAATCCAATTCTTATA 26915

quil TAACTTTATTTAAAAAGAAATCATCAATCCAATTCTTATA 26881

meri TAACTTTATTTtAAAAGAAATCATaAATCCAATTCTTATA 27023

ref TAACTTTATTTtAAAAGAAATCATaAATCCAATTCTTATA 27167

Consensus taactttattt aaaagaaatcat aatccaattcttata

kal TTTAACTTTGTCCTAAACATCATTTTCAATTGCTATCAAC 26955

yor TTTAACTTTGTCCTAAACATCATTTTCAATTGCTATCAAC 26926

jen TTTAACTTTGTCCTAAACATCATTTTCAATTGCTATCAAC 26955

cor TTTAACTTTGTCCTAAACATCATTTTCAATTGCTATCAAC 26955

man TTTAACTTTGTCCTAAACATCATTTTCAATTGCTATCAAC 26955

uni TTTAACTTTGTCCTAAACATCATTTTCAATTGCTATCAAC 26955

tall TTTAACTTTGTCCTAAACATCATTTTCAATTGCTATCAAC 26955

quil TTTAACTTTGTCCTAAACATCATTTTCAATTGCTATCAAC 26921

meri TTTAACTTTGTCCaAAACATCATTTTCAATTGCTATCAAC 27063

ref TTTAACTTTGTCCaAAACATCATTTTCAATTGCTATCAAC 27207

Consensus tttaactttgtcc aaacatcattttcaattgctatcaac

kal ACTAATTATTTAGTCTTT...................... 26973

yor ACTAATTATTTAGTCTTT...................... 26944

jen ACTAATTATTTAGTCTTT...................... 26973

cor ACTAATTATTTAGTCTTT...................... 26973

man ACTAATTATTTAGTCTTT...................... 26973

uni ACTAATTATTTAGTCTTT...................... 26973

tall ACTAATTATTTAGTCTTT...................... 26973

quil ACTAATTATTTAGTCTTT...................... 26939

meri ACTAATTATTTAGTCTTTctcctaacattatagaatgcaa 27103

ref ACTAATTATTTAGTCTTTctcctaacattatagaatgcaa 27247

Consensus actaattatttagtcttt

kal ........................................ 26973

yor ........................................ 26944

jen ........................................ 26973

cor ........................................ 26973

man ........................................ 26973

uni ........................................ 26973

tall ........................................ 26973

quil ........................................ 26939

meri gtatgacttcaataactttagtttagaactatagataatc 27143

ref gtatgacttcaataactttagtttagaactatagataatc 27287

Consensus

kal .....TTTCCATATGCAATATAAATAGGAATAGTCAATAA 27008

yor .....TTTCCATATGCAATATAAATAGGAATAGTCAATAA 26979

jen .....TTTCCATATGCAATATAAATAGGAATAGTCAATAA 27008

cor .....TTTCCATATGCAATATAAATAGGAATAGTCAATAA 27008

man .....TTTCCATATGCAATATAAATAGGAATAGTCAATAA 27008

uni .....TTTCCATATGCAATATAAATAGGAATAGTCAATAA 27008

tall .....TTTCCATATGCAATATAAATAGGAATAGTCAATAA 27008

quil .....TTTCCATATGCAATATAAATAGGAATAGTCAATAA 26974

meri atcttTTTCCATATGCAATATAAATAGGAATAGTCAATAA 27183

ref atcttTTTCCATATGCAATATAAATAGGAATAGTCAATAA 27327

Consensus tttccatatgcaatataaataggaatagtcaataa

kal AATTCAATATGAAATTAGTGTTAGGCAAACAATATATACA 27048

yor AATTCAATATGAAATTAGTGTTAGGCAAACAATATATACA 27019

jen AATTCAATATGAAATTAGTGTTAGGCAAACAATATATACA 27048

cor AATTCAATATGAAATTAGTGTTAGGCAAACAATATATACA 27048

man AATTCAATATGAAATTAGTGTTAGGCAAACAATATATACA 27048

uni AATTCAATATGAAATTAGTGTTAGGCAAACAATATATACA 27048

tall AATTCAATATGAAATTAGTGTTAGGCAAACAATATATACA 27048

quil AATTCAATATGAAATTAGTGTTAGGCAAACAATATATACA 27014

meri AATTCAATATGAAATTAGTGcTAGGCAAACAATATATACA 27223

ref AATTCAATATGAAATTAGTGcTAGGCAAACAATATATACA 27367

Consensus aattcaatatgaaattagtg taggcaaacaatatataca

kal TTTCAAAAACTTCAAGTAGTTTGAACTTGACACATAATTC 27088

yor TTTCAAAAACTTCAAGTAGTTTGAACTTGACACATAATTC 27059

jen TTTCAAAAACTTCAAGTAGTTTGAACTTGACACATAATTC 27088

cor TTTCAAAAACTTCAAGTAGTTTGAACTTGACACATAATTC 27088

man TTTCAAAAACTTCAAGTAGTTTGAACTTGACACATAATTC 27088

uni TTTCAAAAACTTCAAGTAGTTTGAACTTGACACATAATTC 27088

tall TTTCAAAAACTTCAAGTAGTTTGAACTTGACACATAATTC 27088

quil TTTCAAAAACTTCAAGTAGTTTGAACTTGACACATAATTC 27054

meri TTTCAAAAACTTCAAGTAGTTcGAACTTGACACATAATTC 27263

ref TTTCAAAAACTTCAAGTAGTTcGAACTTGACACATAATTC 27407

Consensus tttcaaaaacttcaagtagtt gaacttgacacataattc

kal ATTTTTATCGATATTAGATTGACTATTTTTCCTATTTATA 27128

yor ATTTTTATCGATATTAGATTGACTATTTTTCCTATTTATA 27099

jen ATTTTTATCGATATTAGATTGACTATTTTTCCTATTTATA 27128

cor ATTTTTATCGATATTAGATTGACTATTTTTCCTATTTATA 27128

man ATTTTTATCGATATTAGATTGACTATTTTTCCTATTTATA 27128

uni ATTTTTATCGATATTAGATTGACTATTTTTCCTATTTATA 27128

tall ATTTTTATCGATATTAGATTGACTATTTTTCCTATTTATA 27128

quil ATTTTTATCGATATTAGATTGACTATTTTTCCTATTTATA 27094

meri ATTTTTATCGATATTAGATTGACTATTTTTCCTATTTATA 27303

ref ATTTTTATCGATATTAGATTGACTATTTTTCCTATTTATA 27447

Consensus atttttatcgatattagattgactatttttcctatttata

kal CTTTCAAAATAACTATAACAAAACTTTATATTCATCATAT 27168

yor CTTTCAAAATAACTATAACAAAACTTTATATTCATCATAT 27139

jen CTTTCAAAATAACTATAACAAAACTTTATATTCATCATAT 27168

cor CTTTCAAAATAACTATAACAAAACTTTATATTCATCATAT 27168

man CTTTCAAAATAACTATAACAAAACTTTATATTCATCATAT 27168

uni CTTTCAAAATAACTATAACAAAACTTTATATTCATCATAT 27168

tall CTTTCAAAATAACTATAACAAAACTTTATATTCATCATAT 27168

quil CTTTCAAAATAACTATAACAAAACTTTATATTCATCATAT 27134

meri CTTTCAAAATAACTAcAACAAAACTTTATATTCATCATAT 27343

ref CTTTCAAAATAACTAcAACAAAACTTTATATTCATCATAT 27487

Consensus ctttcaaaataacta aacaaaactttatattcatcatat

kal CATCCAACGAAAACAACTTAAGGTTGCCCTAATGATAACT 27208

yor CATCCAACGAAAACAACTTAAGGTTGCCCTAATGATAACT 27179

jen CATCCAACGAAAACAACTTAAGGTTGCCCTAATGATAACT 27208

cor CATCCAACGAAAACAACTTAAGGTTGCCCTAATGATAACT 27208

man CATCCAACGAAAACAACTTAAGGTTGCCCTAATGATAACT 27208

uni CATCCAACGAAAACAACTTAAGGTTGCCCTAATGATAACT 27208

tall CATCCAACGAAAACAACTTAAGGTTGCCCTAATGATAACT 27208

quil CATCCAACGAAAACAACTTAAGGTTGCCCTAATGATAACT 27174

meri CATCCAACGAAAACAACTTAAaGTTGCCCTAATGATAACT 27383

ref CATCCAACGAAAACAACTTAAaGTTGCCCTAATGATAACT 27527

Consensus catccaacgaaaacaacttaa gttgccctaatgataact

kal ATGAACAAAATCATTCAGAATATAAAATTAATGACTAAAA 27248

yor ATGAACAAAATCATTCAGAATATAAAATTAATGACTAAAA 27219

jen ATGAACAAAATCATTCAGAATATAAAATTAATGACTAAAA 27248

cor ATGAACAAAATCATTCAGAATATAAAATTAATGACTAAAA 27248

man ATGAACAAAATCATTCAGAATATAAAATTAATGACTAAAA 27248

uni ATGAACAAAATCATTCAGAATATAAAATTAATGACTAAAA 27248

tall ATGAACAAAATCATTCAGAATATAAAATTAATGACTAAAA 27248

quil ATGAACAAAATCATTCAGAATATAAAATTAATGACTAAAA 27214

meri ATGAACAAAATCATTtAGAATATAAAATTAATGACTAAAA 27423

ref ATGAACAAAATCATTtAGAATATAAAATTAATGACTAAAA 27567

Consensus atgaacaaaatcatt agaatataaaattaatgactaaaa

kal ATAACTATAAAATATTGATATCTGAATTATAATTAGAAAA 27288

yor ATAACTATAAAATATTGATATCTGAATTATAATTAGAAAA 27259

jen ATAACTATAAAATATTGATATCTGAATTATAATTAGAAAA 27288

cor ATAACTATAAAATATTGATATCTGAATTATAATTAGAAAA 27288

man ATAACTATAAAATATTGATATCTGAATTATAATTAGAAAA 27288

uni ATAACTATAAAATATTGATATCTGAATTATAATTAGAAAA 27288

tall ATAACTATAAAATATTGATATCTGAATTATAATTAGAAAA 27288

quil ATAACTATAAAATATTGATATCTGAATTATAATTAGAAAA 27254

meri ATAACTATAAAATATTGAgATtTGAATTATAATTAGgAAA 27463

ref ATAACTATAAAATATTGAgATtTGAATTATAATTAGgAAA 27607

Consensus ataactataaaatattga at tgaattataattag aaa

kal ACTTGATTATCAGAACATCAACTTGAGCTTTGAGAAATAC 27328

yor ACTTGAcTATCAGAACATCAACTTGAGCTTTGAGAAATAC 27299

jen ACTTGATTATCAGAACATCAACTTGAGCTTTGAGAAATAC 27328

cor ACTTGATTATCAGAACATCAACTTGAGCTTTGAGAAATAC 27328

man ACTTGATTATCAGAACATCAACTTGAGCTTTGAGAAATAC 27328

uni ACTTGATTATCAGAACATCAACTTGAGCTTTGAGAAATAC 27328

tall ACTTGATTATCAGAACATCAACTTGAGCTTTGAGAAATAC 27328

quil ACTTGATTATCAGAACATCAACTTGAGCTTTGAGAAATAC 27294

meri ACTTGtcTATCAtAgCATCAAtTTGAGCTTTGAGAAggAC 27503

ref ACTTGtcTATCAtAgCATCAAtTTGAGCTTTGAGAAggAC 27647

Consensus acttg tatca a catcaa ttgagctttgagaa ac

kal TATTATAGAATTTAAATGGCGACCAAAACTCGAAAATTAA 27368

yor TATTATAGAATTTAAATGGCGACCAAAACTCGgAAATTAA 27339

jen TATTATAGAATTTAAATGGCGACCAAAACTCGAAAATTAA 27368

cor TATTATAGAATTTAAATGGCGACCAAAACTCGAAAATTAA 27368

man TATTATAGAATTTAAATGGCGACCAAAACTCGAAAATTAA 27368

uni TATTATAGAATTTAAATGGCGACCAAAACTCGAAAATTAA 27368

tall TATTATAGAATTTAAATGGCGACCAAAACTCGAAAATTAA 27368

quil TATTATAGAATTTAAATGGCGACCAAAACTCGAAAATTAA 27334

meri TATTATAGAATTTAAATaGCaACCAAAACTCGggAATTAA 27543

ref TATTATAGAATTTAAATaGCaACCAAAACTCGggAATTAA 27687

Consensus tattatagaatttaaat gc accaaaactcg aattaa

kal TCTAGTTTCAGTCAAGATAATCATCACTAAAAAAACTAAT 27408

yor TCTAGTTTCAGTCAAGATAATCATCACTAAAAAAACTAAT 27379

jen TCTAGTTTCAGTCAAGATAATCATCACTAAAAAAACTAAT 27408

cor TCTAGTTTCAGTCAAGATAATCATCACTAAAAAAACTAAT 27408

man TCTAGTTTCAGTCAAGATAATCATCACTAAAAAAACTAAT 27408

uni TCTAGTTTCAGTCAAGATAATCATCACTAAAAAAACTAAT 27408

tall TCTAGTTTCAGTCAAGATAATCATCACTAAAAAAACTAAT 27408

quil TCTAGTTTCAGTCAAGATAATCATCACTAAAAAAACTAAT 27374

meri TCTAGTTTCAGTCAAGATAATCATCACTAAAAAAACTAAT 27583

ref TCTAGTTTCAGTCAAGATAATCATCACTAAAAAAACTAAT 27727

Consensus tctagtttcagtcaagataatcatcactaaaaaaactaat

kal AATGTAAATCAAGAGCAAGGAATTGATCCAACTTAATTAC 27448

yor AATGTAAATCAAGAGCAAGGAATTGATCCAACTTAATTAC 27419

jen AATGTAAATCAAGAGCAAGGAATTGATCCAACTTAATTAC 27448

cor AATGTAAATCAAGAGCAAGGAATTGATCCAACTTAATTAC 27448

man AATGTAAATCAAGAGCAAGGAATTGATCCAACTTAATTAC 27448

uni AATGTAAATCAAGAGCAAGGAATTGATCCAACTTAATTAC 27448

tall AATGTAAATCAAGAGCAAGGAATTGATCCAACTTAATTAC 27448

quil AATGTAAATCAAGAGCAAGGAATTGATCCAACTTAATTAC 27414

meri AATGTAAATCAAGAGCAAGGAATTGATCCAACTTAATTAg 27623

ref AATGTAAATCAAGAGCAAGGAATTGATCCAACTTAATTAg 27767

Consensus aatgtaaatcaagagcaaggaattgatccaacttaatta

kal TCAATACTCTTCAATTCAACACTTAGGCATGTTATTGAGT 27488

yor TCAATACTCTTCAATTCAACACTTAGGCATGTTATTGAGT 27459

jen TCAATACTCTTCAATTCAACACTTAGGCATGTTATTGAGT 27488

cor TCAATACTCTTCAATTCAACACTTAGGCATGTTATTGAGT 27488

man TCAATACTCTTCAATTCAACACTTAGGCATGTTATTGAGT 27488

uni TCAATACTCTTCAATTCAACACTTAGGCATGTTATTGAGT 27488

tall TCAATACTCTTCAATTCAACACTTAGGCATGTTATTGAGT 27488

quil TCAATACTCTTCAATTCAACACTTAGGCATGTTATTGAGT 27454

meri TtAATACTCTTCAATTCAACACTTAGGCATGTTATTaAGT 27663

ref TtAATACTCTTCAATTCAACACTTAGGCATGTTATTaAGT 27807

Consensus t aatactcttcaattcaacacttaggcatgttatt agt

kal TAAATACTTGAAAAGGGAATTTTTCTACCTATCATGATGC 27528

yor TAAATACTTGAAAAGGGAATTTTTCTACCTATCATGATGC 27499

jen TAAATACTTGAAAAGGGAATTTTTCTACCTATCATGATGC 27528

cor TAAATACTTGAAAAGGGAATTTTTCTACCTATCATGATGC 27528

man TAAATACTTGAAAAGGGAATTTTTCTACCTATCATGATGC 27528
[truncated: 59,910 more chars]
